# Bayesian parametric models for survival prediction in medical applications

**DOI:** 10.1186/s12874-023-02059-4

**Published:** 2023-10-26

**Authors:** Iwan Paolucci, Yuan-Mao Lin, Jessica Albuquerque Marques Silva, Kristy K. Brock, Bruno C. Odisio

**Affiliations:** 1 Department of Interventional Radiology, The University of Texas MD Anderson Cancer Center, Houston, TX USA; 2 Department of Imaging Physics, The University of Texas MD Anderson Cancer Center, Houston, TX USA

**Keywords:** Bayesian, Neural network, Machine learning, Survival analysis, Survival outcome

## Abstract

**Background:**

Evidence-based treatment decisions in medicine are made founded on population-level evidence obtained during randomized clinical trials. In an era of personalized medicine, these decisions should be based on the predicted benefit of a treatment on a patient-level. Survival prediction models play a central role as they incorporate the time-to-event and censoring. In medical applications uncertainty is critical especially when treatments differ in their side effect profiles or costs. Additionally, models must be adapted to local populations without diminishing performance and often without the original training data available due to privacy concern. Both points are supported by Bayesian models—yet they are rarely used. The aim of this work is to evaluate Bayesian parametric survival models on public datasets including cardiology, infectious diseases, and oncology.

**Materials and methods:**

Bayesian parametric survival models based on the Exponential and Weibull distribution were implemented as a Python package. A linear combination and a neural network were used for predicting the parameters of the distributions. A superiority design was used to assess whether Bayesian models are better than commonly used models such as Cox Proportional Hazards, Random Survival Forest, and Neural Network-based Cox Proportional Hazards. In a secondary analysis, overfitting was compared between these models. An equivalence design was used to assess whether the prediction performance of Bayesian models after model updating using Bayes rule is equivalent to retraining on the full dataset.

**Results:**

In this study, we found that Bayesian parametric survival models perform as good as state-of-the art models while requiring less hyperparameters to be tuned and providing a measure of the uncertainty of the predictions. In addition, these models were less prone to overfitting. Furthermore, we show that updating these models using Bayes rule yields equivalent performance compared to models trained on combined original and new datasets.

**Conclusions:**

Bayesian parametric survival models are non-inferior to conventional survival models while requiring less hyperparameter tuning, being less prone to overfitting, and allowing model updating using Bayes rule. Further, the Bayesian models provide a measure of the uncertainty on the statistical inference, and, in particular, on the prediction.

**Supplementary Information:**

The online version contains supplementary material available at 10.1186/s12874-023-02059-4.

## Background

Many treatment decisions in medicine are made based on evidence from clinical trials, which evaluate whether an intervention benefits a certain population on average [1]. However, in a personalized medicine approach, such decisions would preferably be made based on predictions for an individual patient [2, 3]. Predictive models using machine learning (ML) could learn the patterns in patient characteristics for which one treatment works better than another. In contrast to other ML tasks in medical applications, such as radiological image segmentation or disease detection, the true result is often unknown because the patient may experience the event of interest many years after the treatment (e.g. disease recurrence or death). Survival models have been introduced for statistical analysis of such data and incorporate the time to an event in addition to the event itself [4].

Predictive models in medical applications have unique requirements, as the size of datasets is often limited because diseases are divided into many subcategories. For example, cancers are subclassified according to the stage, histological findings, and genetic mutations with the list of subclassifications continuously increasing [5]. Thus, the sample size for each subtype treated in a single health care center or region might be very small. Such datasets are especially prone to overfitting. Most ML algorithms use regularization to prevent overfitting, which requires more hyperparameters tuning during the training process [6]. Another challenge is retraining models due to changes in the patient population or treatment strategies [7]. When adapting a model from an older dataset or from another institution, it is often impossible to keep the original training data due to data privacy concerns. Some models can be updated using transfer learning techniques where the training process is initialized using the existing model [8]. However, this approach treats the existing parameters as random numbers, which ignores the amount of information that is in these parameters (e.g. 10 vs. 100 samples). It is also essential for physicians who base their clinical decisions on such predictions to have a measure of uncertainty about a prediction [9].

**Fig. 1:**
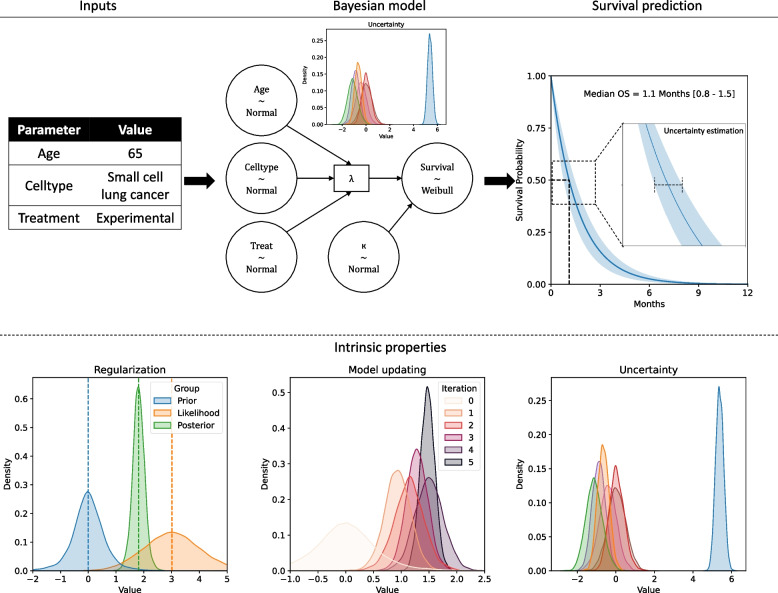
Overview of Bayesian approach to survival prediction. **a** data pipeline with tabular input, Bayesian model for survival times, and individualized survival prediction with uncertainty; **b** Intrinsic properties of Bayesian models that are essential in medical applications: Regularization through non-informative priors, model updating using Bayes rule through informative priors from previous model, estimation of uncertainty through full posterior probability distributions of each parameter

Bayesian models have intrinsic properties, such as regularization through non-informative priors, model updating using Bayes rule, and estimation of the uncertainty of the prediction, that would make them ideal for medical applications (Fig. 1). Yet, these types of models are rarely used for survival predictions and in medical applications in general. The aim of this work is to evaluate Bayesian parametric survival models with respect to prediction performance compared to other state-of-the-art models. We hypothesize that Bayesian survival models i)are superior to state-of-the art models (i.e. Cox Proportional Hazards, Random Survival Forest [10], DeepSurv [11]) with less overfitting and hyperparameter tuning; ii) have equivalent performance when re-trained on the whole dataset compared to updated using Bayes rule.

## Materials and methods

### Bayesian parametric survival models – Mathematical background

The Bayesian parametric survival models are composed of two basic building blocks. The first, is the underlying distribution by which the survival times *T*_*Survival*_ are distributed. In the second part, the parameters of the distribution are given by a function of the input data and the coefficients *θ*. Additionally, a prior is set to the coefficients *θ*_*i*_ which are estimated during the training phase.

1 $$\begin{array}{c}{\uptheta }_{i}\sim Normal \left({\mu }_{i},{\sigma }_{i}\right)\\ \begin{array}{c}\uplambda =f\left(X,\uptheta \right)\\ {T}_{Survival}\sim Dist\left(\uplambda \right)\end{array}\end{array}$$

#### Functions for distribution parameters

##### Linear function

The most basic predictor function is a linear combination of the predictors X and the coefficients θ. X_0_ is set to one such that θ_0_ represents the intercept. The result is exponentiated to ensure that the parameters are positive, which is required by the probability distributions of the survival time.

2 $$\lambda =f(X,\uptheta )=exp({\sum }_{i=0}^{n}{\uptheta }_{i}{x}_{i})$$

##### Neural network

For non-linear relationships, a feed forward neural network is used with L hidden layers consisting of the weights and biases denoted as W and b respectively (later combined as θ). X_1_ is set to the model input. The hyperbolic tangent function is used as activation function between the layers.

3 $${a}^{l}=tanh\left({W}^{l}\cdot {a}^{l-1}+{b}^{l}\right)$$

For the last layer, the result is exponentiated to ensure that the parameters are positive.

4 $$\uplambda =exp\left({W}^{L}\cdot {a}^{L-1}+{b}^{L}\right)$$

The number of outputs of the last layer equals the number of parameters of the survival time distribution (see next section).

#### Distributions of the survival time

The most common probability distributions for survival times are the Exponential and the Weibull distribution. Following, the mathematical description of the survival model based on these distributions are described.

##### Exponential model

The time to event follows an Exponential distribution which is parametrized by a rate parameter (λ) [12]. The parameter λ is predicted using one of the predictor functions using inputs X and coefficients θ:

5 $$\begin{array}{c}\lambda =f(X,\theta )\\ {T}_{Survival}\sim Exponential(\lambda )\end{array}$$

For censored observations, the true survival time t_true_ is larger than the time they were under observation t_observed_. This probability is given by the log complementary cumulative distribution function (CDF):

6 $$ln (P({t}_{true}>{t}_{observed})) =-\lambda t$$

The probability of an event at any point in time is the given by the CDF:

7 $${P}_{event}\left(t;\lambda ,\kappa \right)=1-{\mathrm{e}}^{-\uplambda t}$$

The survival function is then the complement of the CDF (1-CDF):

8 $${P}_{survival}\left(t;\lambda ,\kappa \right)=1-{P}_{event}\left(t;\lambda ,\kappa \right)={\mathrm{e}}^{-\lambda t}$$

As an intuition, the parameter λ represents rate of events with a higher value corresponding to shorter time-to-event.

##### Weibull model

In this model the time to event follows a Weibull distribution which is parametrized by a scale parameter (λ) and a shape parameter (κ) [12]. These parameters are predicted using one of the predictor functions using inputs X and coefficients θ_λ_ and θ_κ_.

9 $$\begin{array}{c}\lambda =f(X, {\theta }_{\uplambda })\\ \kappa =f(X, {\theta }_{\kappa })\\ {T}_{Survival}\sim Weibull\left(\lambda ,\kappa \right)\end{array}$$

As for the Exponential model, the survival probability for censored observations is given by the log complementary CDF:

10 $$ln (P({t}_{true}>{t}_{observed}))=-{(\lambda t)}^{\kappa }$$

The probability of an event at any point in time is the given by the CDF.

11 $${P}_{event}\left(t;\lambda ,\kappa \right)=1-{\mathrm{e}}^{-{(\lambda t)}^{\kappa }}$$

The survival function is then the complement of the CDF (1-CDF):

12 $${P}_{survival}\left(t;\lambda ,\kappa \right)=1-{P}_{event}\left(t;\lambda ,\kappa \right)={\mathrm{e}}^{-{(\lambda t)}^{\kappa }}$$

As an intuition, the parameter λ represents the timepoint when the probability of having experienced an event is 63.2%. The shape parameter κ represents whether the probability of an event increases (κ > 1), decreases (κ < 1) or stays constant (κ = 1) over time. With κ = 1 the model is equivalent to the Exponential model. The Weibull model has a setting that specifies whether the shape parameter κ is modeled ad intercept only (default setting in our implementation).

### Bayesian parametric survival models—training, predicting, and updating

**Fig. 2:**
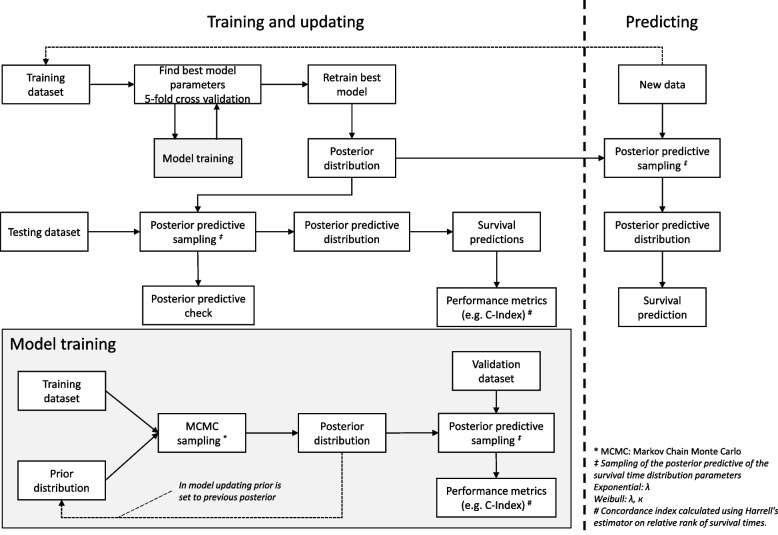
General workflow for training, updating, and testing of the Bayesian parametric survival models using fivefold cross validation for feature selection and hyperparameter tuning. Model training itself is detailed in the gray box

The proposed Bayesian parametric survival models are trained using a combined Bayesian model building and machine learning workflow incorporating specification of priors, sampling, model checking using posterior predictive checks, testing, and predicting using sampling of the posterior predictive distribution (Fig. 2). Details on the mathematical background and model training and updating are in the following sections.

#### Model training

The models were implemented using the probabilistic programming framework PyMC (v4.3.0) [13]. The priors for the coefficients of the model parameters were set to $\sim Normal(0, 1)$, representing non-informative priors. The models were fitted using Hamiltonian Monte Carlo using the No-U-Turn Sampler (NUTS) provided in the software package PyMC [14]. Three Bayesian parametric survival (BPS) models were used in this study; an exponential and a Weibull model with linear predictor (BPS Exp and BPS Wb respectively) and a Weibull model with a Neural Network predictor (BPS WbNN).

#### Model prediction

The predictions were performed using posterior predictive sampling to obtain the probability distribution of the parameters λ and κ (Weibull model only) with a new set of input data. These parameters are then used to estimate the survival function using the respective formulas of the complementary CDF. The survival function is evaluated at the mean ± standard deviation to provide a measure of uncertainty.

#### Model updating

Bayesian models provide a natural way to retrain with new data using Bayes rule. With this, the posterior probability of the model parameters of the old model is used as prior of the new model. When training a model from scratch, the priors are set to non-informative priors like $\sim Normal(0, 1)$. In the initial training this acts like a regularization. For model updating, informative priors can be used based on the posterior distribution of the old model. Once the prior is set to the “old” posterior distribution, the workflow for obtaining the likelihood and the “new” posterior during model updating is identical to the initial model training. For example, if the posterior distribution of *λ*_*param*_ has a mean of 2 and a standard deviation of 0.5, the prior would be set as such ${\lambda }_{param}\sim Normal(2, 0.5)$.

13 $$P\left(\theta |data\right)=\frac{P\left(\theta \right)\times P\left(data|\theta \right)}{P\left(data\right)}$$

P(θ |data) is the posterior probability of the parameters θ after observing the data. P(θ), is the prior probability before observing the data, which can be obtained from a previous model. P(data|θ) is the likelihood that the observed data was generated by a model with the parameters θ. P(data) is the probability of the data itself. Since the data has been observed, the probability is one and therefore the equation can be interpreted as follows.

14 $$Posterio\mathrm{r}\propto Prior\times Likelihood$$

If a model is trained on a very large sample size the posterior distributions can be very informative (e.g. small standard deviations, means far away from zero). Thus, it would require more samples during model updating to influence the new posterior distribution. Widening the standard deviation of the prior would make them less informative and therefore reduce the effect on the posterior in such cases.

### Evaluation

The evaluation uses data of two experiments and a case study. The first experiment compares the models using five different public datasets. The second compares the models after updating on the four larger datasets. Finally, a case study is presented on the Veteran dataset. The public datasets were: AIDS Clinical Trials Group (ACTG), German Breast Cancer Study (GBCS), Veteran lung cancer (Veteran), Worcester Heart Attack Study (WHAS), and primary biliary cirrhosis (PBC) [4, 15, 16]. Details are in section Datasets.

#### Experiment 1: comparison with other survival models

The models were compared against the Cox proportional hazards (CoxPH) model [17], Random survival forest (RSF) [10], and deep neural network CoxPH (DeepSurv) [11]. These models are easy-to-use and available in well-known and maintained open source packages. The CoxPH and RSF models were implemented using scikit-survival(v0.19.0) and the DeepSurv model using PyCox(v0.2.3) and PyTorch(v1.13.0). All models were applied to each of the 5 datasets (see Datasets).

All models were evaluated in common ML pipeline using train/test split, cross validation, feature selection and hyperparameter tuning using scikit-learn [18] (v1.1.3) and scikit-optimize (v0.9.0). The hyperparameters and the boundaries used during tuning are described in Table 1. All splits (train/test, cross validation) were performed at random. The following procedure was performed 47 times for each model and dataset combination (see Sample size estimation).

**Table 1:** Hyperparamters that were optimized during the training process of the different models in evaluation

Model	Hyperparameter	Description	Bounds
**Cox PH**	*alpha*	Regularization parameter	(10^–5^, 0.9)
	*k*	K-best features	[1, n_features]
**RSF**	*n_estimators*	Number of trees	[5,25]
	*tree_depth*	Max depth per tree	[3, 5, 7, 9]
	*k*	K-best features	[1, n_features]
**DeepSurv**	*hidden_units*	Nr of hidden units	(X, n_features)
	*lr*	Learning rate	(10^–5^, 10^–1^)
	*dropout*	Dropout	(0.1, 0.5)
	*k*	K-best features	[1, n_features]
**BPS Exp**	*priors_sd*	Standard deviation of priors	(10^–1^, 10^1^)
	*k*	K-best features	[1, n_features]
**BPS Wb**	*priors_sd*	Standard deviation of priors	(10^–1^, 10^1^)
	*k*	K-best features	[1, n_features]
**BPS Wb NN**	*n_hidden_layers*	Number of hidden layers	
	*priors_sd*	Standard deviation of priors	(10^–1^, 10^1^)
	*k*	K-best features	[1, n_features]

*CoxPH* Cox Proportional Hazards, *RSF* Random Survival Forest, *BPS* Bayesian Parametric Survival model, *Exp* Exponential, *Wb* Weibull, *NN* Neural Network

First, the dataset was split into training and test set using an 80%/20% split at random. On the training set, the continuous variables were normalized. Feature selection and hyperparameter tuning (Table 1) were performed using fivefold cross validation and Bayesian search. Features were ordered using mutual information with median survival, and k-best features were selected with k as a hyperparameter. The hyperparameters and features with the best average fivefold cross-validation performance were used to train the final model on the whole training data set. The final model was then evaluated against the test set using the concordance index (C-Index) as evaluation metric [19]. In addition, the difference in C-Index between the training and test set was used to estimate overfitting.

#### Experiment 2: model updating

**Fig. 3:**
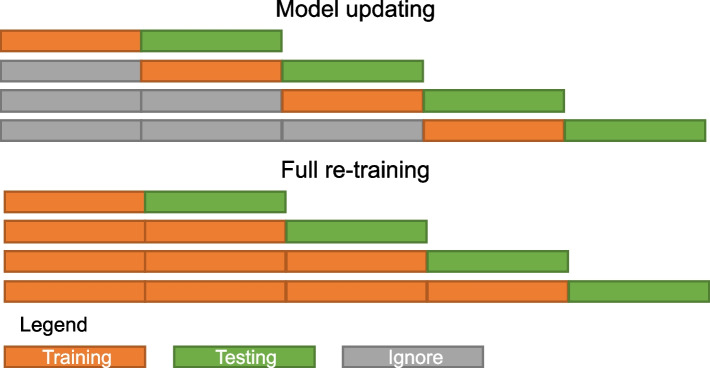
Use of data-partitions for model updating vs. full re-training. In model updating only the two most recent data partitions is used for training (orange) and testing (green). Previously used training data is ignored (grey). In full re-training, all previously used training data partitions are combined with the new training data (orange). The data partition for testing remains the same (green)

The Exponential, Weibull, and DeepSurv model were updated using two strategies – full retraining and model updating. Therefore, the dataset was split into partitions, representing data collected in different timeframes (Fig. 3). In full retraining, the training data from all the previous partitions is combined with the data of the current partition. In model updating, the training data from the previous partition is discarded and the model updated using the new partition only. For the Bayesian models the posterior distribution of the previous model was used as prior for the new model. For DeepSurv transfer learning was applied. The C-Index is used as a performance metric at each partition.

We further investigate the internals of the Bayesian models to see whether the model parameters are in agreement. Therefore, the posterior distributions of the parameters of the models were extracted after each partition. The probability density function (PDF) for each parameter was estimated using kernel density estimation. The overlap index (OVI) between the PDF of full training vs. model updating was computed after each partition [20].

For this experiment, the four datasets with > 200 samples were used. Datasets with > 1000 samples were split into 10 and the others into 6 partitions. Datasets with year of enrollment (WHAS) were split according to the year variable and the others by random. The experiment was repeated 74 times (see Sample size estimation).

#### Case studies on the Veterans dataset

To show the benefit of models with integrated uncertainty estimation, a series of cases of the Veteran dataset is presented. All six models (CoxPH, RSF, DeepSurv, BPS Exp, BPS WB, BPS Wb NN) were trained on the Veteran dataset. The variables *age*, *trt*, *celltype*, and interaction terms between *trt* and *celltype* are used. Survival predictions, along with the uncertainty (Bayesian models only), are presented for all six models.

### Datasets

Five public datasets for survival analysis were used to evaluate the models. The original and pre-processed (binarized and one-hot encoded) datasets are available on Zenodo (https://zenodo.org/record/7429722). Only complete cases were included, and no imputation was performed.

#### AIDS Clinical Trials Group Study (ACTG)

This is a dataset from the AIDS Clinical Trials Group (ACTG) study, a randomized-control trial of HIV patients 1151 observations and 16 variables [4]. Binary variables (*sex, hemophil*) were transformed to 0 and 1. Categorical variables (*strat2, txgrp, ivdrug, karnof, raceth*) were one-hot encoded. Continuous variables (*age, cd4, priorzdv*) were normalized.

#### German Breast Cancer Study data (GBCS)

This is a dataset from the German Breast Cancer Study (GBCS) study of patients with primary node positive breast cancer with 686 observations and 16 variables [4]. Binary variables (*menopause, hormone*) were transformed to 0 and 1. Categorical variables (*grade*) were one-hot encoded. Continuous variables (*age, size, nodes, prog_recp, estrg_recp*) were normalized.

#### Veteran lung cancer (Veteran)

This is a dataset from the a randomized-control trial of two treatments for lung cancer with 137 observations and 8 variables [15]. Due to the smaller sample size, this dataset was not used for the model updating experiment. Binary variables (*trt, prior*) were transformed to 0 and 1. Categorical variables (*celltype*) were one-hot encoded. Continuous variables (*age, karno, diagtime*) were normalized.

#### Worcester Heart attack study (WHAS)

This is a dataset from the Worcester Heart Attack Study (WHAS) of patients admitted for acute myocardial infarction with 481 observations and 14 variables [4]. Binary variables (*sex, sho, chf, miord*) were transformed to 0 and 1. Categorical variables (*mitype, yrgrp*) were one-hot encoded. Continuous variables (*age, ckp*) were normalized.

#### Primary Biliary Cirrhosis (PBC)

This is a dataset from a randomized-control trial of the drug D-penicillamine for primary biliary cirrhosis (PBC) of the liver with 418 observations and 20 variables [16]. Binary variables (*sex, trt, ascites, hepatomegaly, spiders*) were transformed to 0 and 1. Categorical variables (*edema, histologic*) were one-hot encoded. Continuous variables (*age, bilirubin, cholesterol, albumin, copper, alkphosphotase, sgot, triglycerides, platelet, prothrombin*) were normalized.

### Statistical methods

#### Sample size estimation

In the superiority analysis of the different survival models, we assume a 0.02 higher C-Index in the best performing models with a standard deviation of 0.025. Six models are compared with each other per dataset, resulting in 15 comparisons per dataset. Using Bonferroni correction, the significance level was adjusted to 0.05 / 15 = 0.0033. Thus, we estimated that a total of 47 samples per model/dataset combination are necessary to achieve a power of 0.8 at a significance level of 0.0033 to detect a 0.02 ± 0.025 difference.

For the equivalence analysis of two model updating strategies, we defined a range of practical equivalence of [-0.01, 0.01]. Three models are compared with each other per dataset, resulting in 3 comparisons per dataset. Using Bonferroni correction, the significance level was set to 0.05 / 3 = 0.016. Thus, we estimated that a total of 75 samples per model/dataset combination are necessary to achieve a power of 0.8 at a significance level of 0. 016.

The code for the sample size estimation is available in the Supplementary material (Sample size calculation.pdf).

#### Statistical analysis

Bayesian statistics was used in this analysis. Credible Intervals (CrI) were estimated using the high definition interval (HDI) of the posterior distribution. In the comparative analysis, significant outperformance/underperformance by a model was defined when the median performance of all other models lied outside the CrI of the best/worst performing model. Using Bonferroni correction, the HDI was set at 99.66% (1–0.05/15). In the model updating analysis, Bayesian equivalence testing was used to test whether the difference between Full training and model updating is within the region of practical equivalence (ROPE) of [-0.01, 0.01]. Using Bonferroni correction, the HDI was set at 98.33%(1–0.05/3). The two training methods were considered equivalent or non-equivalent if 100% or 0% of the CrI of the difference in performance was within the ROPE respectively. Otherwise, the result was considered inconclusive. Bayesian analysis was performed using rstanarm (v 2.21.3) and bayestestR (0.13.0). All plots were created using ggplot2 and ggpubr. All statistical analyses were performed in R and RStudio. Sample size estimation was performed using pwr (v 1.3.0) and TOSTER (v 0.4.2) [21]. The statistical analysis is available in the Supplementary material (Comparison of algorithms.pdf, Overlap of posterior distributions.pdf, Posterior distributions.pdf, Retraining.pdf).

## Results

### Comparison with state-of-the art models

Overall, none of the models consistently outperformed or underperformed. In all datasets, at least one model was within the CrI of the best performing model, and therefore no model outperformed. For the PBC dataset, the BPS Wb NN model performed significantly better than all other models. The DeepSurv and the BPS Wb NN model, two neural network-based models, were never the top performing model. However, the PM NN model was always within the CrI of the best performing model. Table 2 shows detailed results of all experiments.

**Table 2:** Results of the comparative experiments of six models on five public datasets. Per dataset, the best performing model (**bold**) and the models within the 95% CI of the best performing model (*italics*) are highlighted

**Characteristic**	**CoxPH**, *N* = 47^a^	**DeepSurv**, *N* = 47^a^	**BPS Exp**, *N* = 47^a^	**BPS Wb**, *N* = 47^a^	**BPS Wb NN**, *N* = 47^a^	**RSF**, *N* = 47^a^
**ACTG**						
C-Index	**0.754** [0.731—0.778]	0.714 [0.691—0.739]	*0.739* [0.715—0.763]	*0.745* [0.718—0.776]	0.748 [0.724—0.770]	0.729 [0.704—0.751]
**GBCS**						
C-Index	*0.736* [0.715—0.757]	*0.731* [0.711—0.751]	**0.737** [0.721—0.756]	*0.735* [0.719—0.751]	*0.735* [0.718—0.753]	**0.737** [0.718—0.756]
**PBC**						
C-Index	0.820 [0.801—0.838]	0.813 [0.796—0.829]	*0.825* [0.808—0.845]	0.824 [0.805—0.843]	**0.842** [0.825—0.858]	*0.832* [0.814—0.851]
**Veteran**						
C-Index	**0.709** [0.692—0.727]	*0.699* [0.674—0.724]	*0.700* [0.668—0.731]	*0.696* [0.665—0.729]	*0.704* [0.678—0.730]	*0.693* [0.671—0.715]
**WHAS**						
C-Index	*0.817* [0.790—0.843]	*0.825* [0.804—0.845]	*0.828* [0.806—0.850]	*0.832* [0.811—0.853]	**0.834** [0.814—0.853]	*0.821* [0.794—0.848]

^a^Mean [99.6% CrI]

**best performing model**

*within 95% CI of best performing model*

**Fig. 4:**
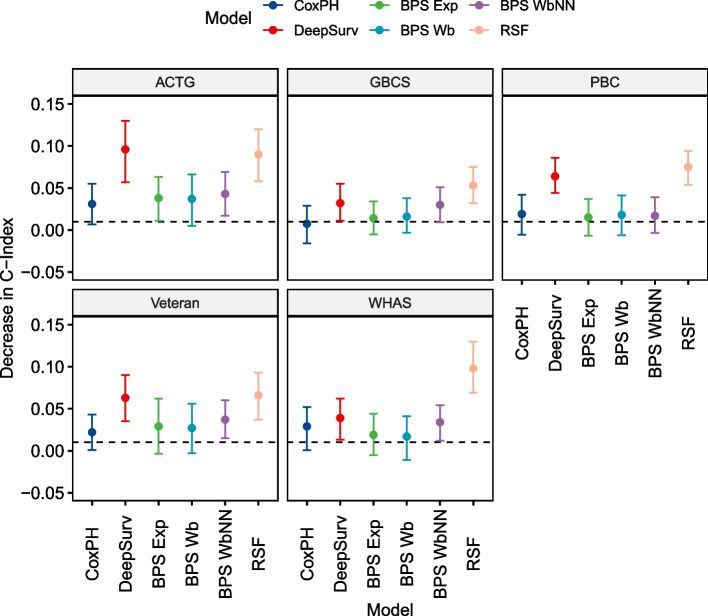
Overfitting of the models under evaluation shows that Weibull model had consistently small overfitting, whereas DeepSurv and RSF models showed consistently larger overfitting

When looking at overfitting, represented by the difference in C-Index between training and testing, it shows that the Cox PH model and the Weibull-based Bayesian models overfit the least. The Exponential and Neural Network-based model overfit more but are mostly within the CrI of the former. The DeepSurv and RSF model consistently show significantly more overfitting than the least overfitting model. Detailed results are available in Table 3 and Fig. 4.

**Table 3:** Results of the comparative experiments evaluating overfitting. Per dataset, the least overfitting model (**bold**) and the models within the 95% CI of the least overfitting model (*italics*) are highlighted

**Characteristic**	**CoxPH** *N* = 47^a^	**DeepSurv** *N* = 47^a^	**BPS Exp** *N* = 47^a^	**BPS Wb** *N* = 47^a^	**BPS WbNN** *N* = 47^a^	**RSF** *N* = 47^a^
**ACTG**						
C-Index	**0.031** [0.005—0.056]	0.096 [0.059—0.131]	*0.038* [0.011—0.063]	*0.037* [0.007—0.065]	*0.043* [0.015—0.074]	0.090 [0.058—0.122]
**GBCS**						
C-Index	**0.007** [-0.014—0.029]	0.032 [0.008—0.055]	*0.014* [-0.006—0.032]	*0.016* [-0.005—0.038]	0.030 [0.010—0.052]	0.053 [0.034—0.074]
**PBC**						
C-Index	*0.018* [-0.004—0.040]	0.064 [0.040—0.089]	*0.015* [-0.005—0.036]	*0.018* [-0.008—0.041]	**0.017** [-0.003—0.037]	0.074 [0.053—0.095]
**Veteran**						
C-Index	**0.022** [0.002—0.044]	0.063 [0.035—0.089]	*0.029* [-0.005—0.062]	*0.027* [-0.005—0.056]	*0.037* [0.014—0.060]	0.066 [0.034—0.097]
**WHAS**						
C-Index	*0.028* [0.004—0.051]	*0.039* [0.014—0.066]	*0.020* [-0.005—0.046]	**0.016** [-0.009—0.040]	*0.034* [0.012—0.057]	0.098 [0.071—0.125]

^a^Mean [95% CrI]

**least overfitting model**

*within 95% CI of least overfitting model*

### Partial re-training vs. full re-training

**Fig. 5:**
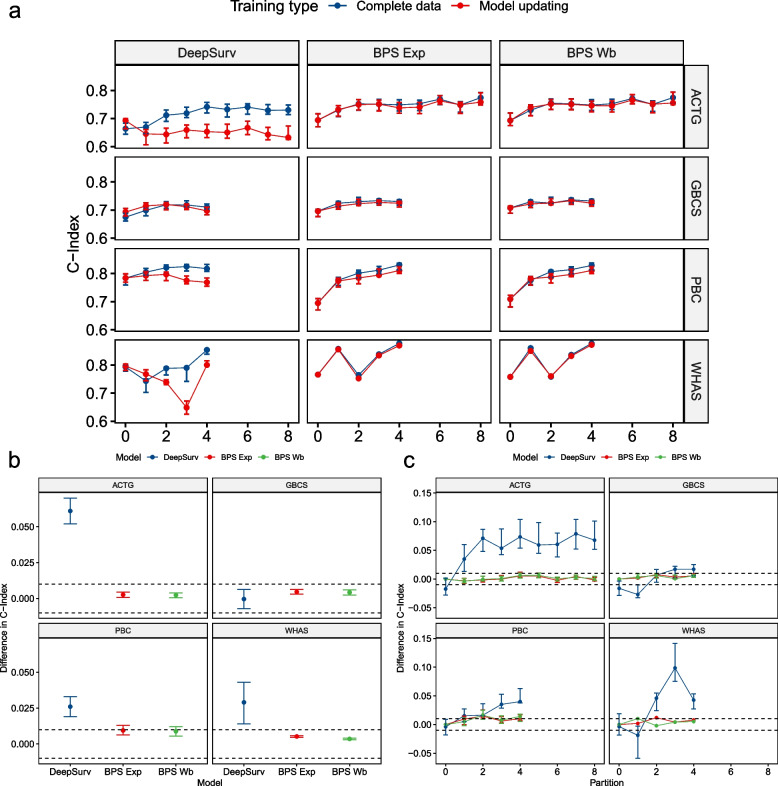
**a** Model performance after each partition, **b** Difference between full training and model updating after each partition. Dashed lines represent the region of practical equivalence [-0.01, 0.01], **c** Difference between full training and model updating. Dashed lines represent the region of practical equivalence [-0.01, 0.01]

In all 4 datasets, the performance of the updated Bayesian parametric models is similar to the model with full training and within the CrI at each partition (Fig. 5a). In the PBC dataset, the performance difference of the Bayesian models crosses the equivalence boundary and equivalence can neither be accepted nor rejected (Fig. 5 b). The performance of the DeepSurv model diverged and decrease for the ACTG, PBC, and WHAS dataset and is clearly outside the equivalence boundary. In the GBCS dataset, the performance difference is within the equivalence boundary on average but Fig. 5c show initial performance difference below the equivalence boundary followed by a difference above the equivalence boundary. Detailed results are presented in Table 4.

**Table 4:** Detailed results of model updating vs. full retraining

Model	Experiment	C-Index^a,b^	% in ROPE	H_Equivalence_
DeepSurv	ACTG	0.0610 [0.0520—0.0700]	0.0	Rejected
BPS Exp	ACTG	0.0026 [0.0007—0.0045]	100.0	Accepted
BPS Wb	ACTG	0.0023 [0.0006—0.0039]	100.0	Accepted
DeepSurv	GBCS	-0.0004 [-0.0072—0.0062]	100.0	Accepted
BPS Exp	GBCS	0.0046 [0.0030—0.0063]	100.0	Accepted
BPS Wb	GBCS	0.0042 [0.0023—0.0060]	100.0	Accepted
DeepSurv	PBC	0.0260 [0.0190—0.0330]	0.0	Rejected
BPS Exp	PBC	0.0094 [0.0063—0.0130]	68.1	Undecided
BPS Wb	PBC	0.0088 [0.0054—0.0120]	79.6	Undecided
DeepSurv	WHAS	0.0290 [0.0140—0.0430]	0.0	Rejected
BPS Exp	WHAS	0.0052 [0.0046—0.0057]	100.0	Accepted
BPS Wb	WHAS	0.0035 [0.0030—0.0041]	100.0	Accepted

^a^Median [98.3% CrI]

^b^Difference between full re-training and model updating

H_Equivalence_: Hypothesis that difference in model performance is *N* = 75

**Fig. 6:**
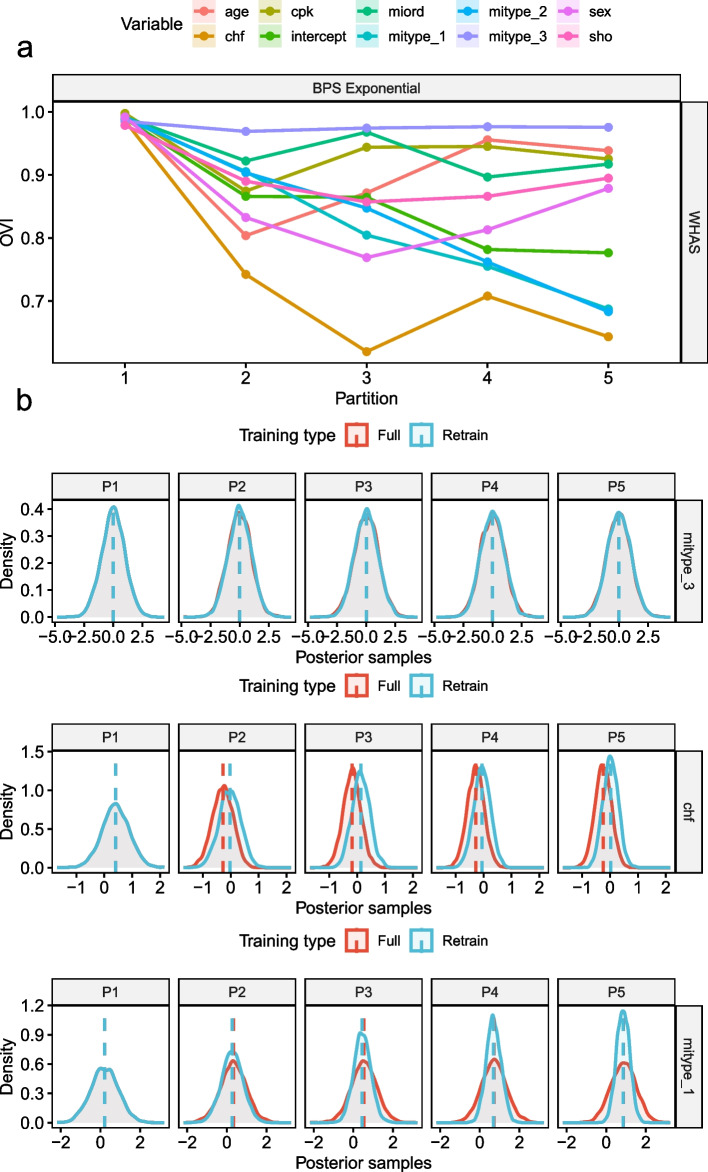
Overlap index of posterior distributions (**a**) with three different patterns of overlap between Full and partial re-training (**b**)

Figure 6 shows the posterior distributions and the OVI between full training and model updating of the BPS Exponential model trained on the WHAS dataset. Some parameters are in strong agreement between the training modes (> 0.9 OVI) whereas others disagree more and more over time. Of those who disagree in terms of lower OVI two different types of disagreement were observed. One type represents a shift in the median of the posterior PDF, which would indicate a change in feature importance. The second type represents higher certainty (smaller standard deviation) about the parameter in the model updating compared to full retraining. Despite these changes in the model parameters, the performance difference was less than 1% in terms of C-Index. Further examples are shown in the Supplementary materials.

### Case studies

**Fig. 7:**
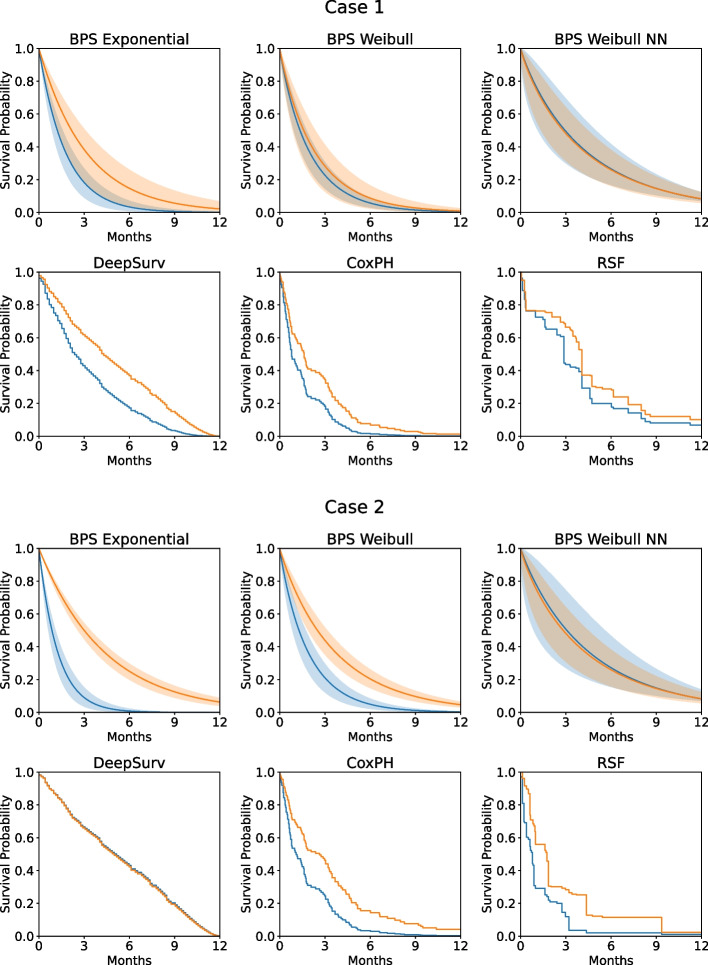
Predictions for case 1 using different survival models with treatment from the control arm or the experimental treatment. Predictions for case 2 using different survival models with treatment from the control arm or the experimental treatment

To show examples, all models were trained on the Veteran dataset. The first case is a 50-year-old male with lung adenocarcinoma (Fig. 7left). The second case is a 65-year-old male with small cell lung cancer (Fig. 7 right). In both cases, two survival predictions are shown; one for the standard-of-care treatment and one for the experimental treatment. In the first case, all models favor the experimental treatment. However, the Bayesian models show some uncertainty, where the standard-of-care treatment is within the uncertainty region of the experimental treatment. In the second case, all, except of the two Neural Network-based models, favor the experimental treatment. In this case, the Bayesian models have a much smaller uncertainty and thus predict a clear survival benefit for the experimental treatment.

## Discussion

In this study, we evaluated the performance of Bayesian parametric survival models compared to state-of-the art models. While these models were among the best performing, no model significantly outperformed all others. This emphasizes the importance of model selection per application. However, our results suggest that using a Bayesian parametric model would lead to good performance in all datasets. In addition, we demonstrate that Bayesian models can be updated as more data is collected without requiring the original training data by applying Bayes rule. The performance of these updated models was equivalent to the performance of models trained on all data.

The possibility of updating models without the original data and without sacrificing model performance has several practical advantages. First, privacy concerns often preclude data sharing among institutions. Second, the proposed models use Bayes rule to enable model updating which only requires the summary statistics of the original models used as priors to update the new model (e.g. in another institution). In this work, the performance between these two re-training approaches resulted in equivalent performance. To the best of our knowledge, this application of Bayesian models has not been studied so far.

In secondary analyses, we showed that the Bayesian models had less overfitting (performance decrease between training and testing) despite requiring less hyperparameter tuning for regularization. Regularization is achieved through non-informative priors. This is contrary to the belief that Bayesian models are more complex, which is mostly related to the lack of easy-to-use frameworks like scikit-learn. Previously reported non-parametric Bayesian models either did not provide code or use more complicated methods for model fitting [22, 23]. Even though the performance between training approaches was equivalent the model parameters diverged in some cases. We identified two distinct patterns of divergence: a difference in the mean or the standard deviation. While these divergences did not affect the performance in this study, it shows that attention should be paid to such effects before deploying a model. This is also stated by the US FDA in their good machine learning guidelines, that re-training risks must be managed [7].

This study has limitations. The datasets used are relatively small when compared to other AI studies. However, there are many scenarios where researchers initially are only able to collect a few hundred samples. Further, we did not compare the models in terms of computation time for training and predicting. In general, all models could be trained in < 10 min including hyperparameter optimization. Bayesian frameworks, such as PyMC, are undergoing heavy development with a focus on computation time. Computational libraries such as NumPy or JAX are getting faster through accessing GPUs and parallel computing optimizations. Therefore, a comparison in terms of computation performance would likely be outdated within a very short period of time.

In conclusion, Bayesian parametric survival models have similar performance as state-of-the art models while requiring less hyperparameter tuning and resulting in less overfitting. In addition, they allow for model updating using Bayes rule with equivalent performance compared to re-training using the full dataset. These properties of Bayesian parametric survival models would make them ideal for medical applications with their unique requirements due to the high stakes decisions and data privacy regulations.

### Supplementary Information

**Additional file 1.** Comparison of algorithms.

**Additional file 2.** Overlap of posterior distributions.

**Additional file 3.** Posterior distributions.

**Additional file 4.** Retraining.

**Additional file 5.** Sample size calculation.

**Additional file 6.** Overfitting.

## Data Availability

The data for this study is available in the GitHub repository and on Zenodo (https://zenodo.org/record/7429722). The code is publicly available in the following GitHub repository. https://github.com/ipa/pymc-survival.
