# Supplementary material for: Bayesian parametric models for survival prediction in medical applications
Source: BMC Med Res Methodol. 2023 Oct 26;23:250. doi: 10.1186/s12874-023-02059-4 (PMC10605790; doi:10.1186/s12874-023-02059-4)
Supplement: Supplementary file 1 — Additional file 1. Comparison of algorithms. [file 12874_2023_2059_MOESM1_ESM.pdf]

# Bayesian parametric models for survival prediction in medical applications

**Comparison of algorithms**

Iwan Paolucci, PhD

7/4/23

## Table of contents

**Aim** 1

**Setup** 2

**Load data** 3

Preprocess data . . . 3

**Results** 5

Graphical . . . 5

Tabular . . . 6

Bayesian . . . 7

Frequentist . . . 10

**Diagnostics** 10

Check distributionof C-Indexes . . . 10

## Aim

The aim of this experiment is to compare Bayesian parametric survival models from the pymc-survival package against CoxPH, Random Survival Forest (RSF) and DeepSurv models.

***Significant outperformance*** is defined when all other models are below the 95% CI of the best performing model.

## Setup

```r
library(ggplot2)
library(ggpubr)
library(gtsummary)
library(dplyr)
library(gt)
library(rstanarm)
library(parameters)
```

## Load data

```
data.raw <- read.csv('data/results_comparison.csv')
```

### Preprocess data

```
data <- data.raw %>%
  filter(model != 'pmsurv_weibull_linear_failed') %>%
  mutate(experiment = ifelse(experiment == 'data/aids/', 'data/aids', experiment),
         experiment_lbl = factor(experiment, labels = c("ACTG", "GBCS", "PBC", "Veteran",
         model_lbl = factor(model,
                            levels = c('cox', 'deepsurv', 'pmsurv_exponential', 'pmsurv_we
                            labels = c("CoxPH", "DeepSurv", "BPS Exp", "BPS Wb", "BPS WbNN
         # run_id = rep(seq(1, 100), times = nrow(.)/100)) %>%
  # filter(run_id <= 47)
```

```
data %>%
  select(model_lbl, model, experiment_lbl, cindex) %>%
  group_by(model_lbl, experiment_lbl) %>%
  summarise(
    n = n(),
    lbl = first(model),
  ) %>% as.data.frame()
```

```
`summarise()` has grouped output by 'model_lbl'. You can override using the
`.groups` argument.
```

```
   model_lbl experiment_lbl  n                  lbl
1      CoxPH           ACTG 47                  cox
2      CoxPH           GBCS 47                  cox
3      CoxPH            PBC 47                  cox
4      CoxPH        Veteran 47                  cox
5      CoxPH           WHAS 47                  cox
6   DeepSurv           ACTG 47             deepsurv
7   DeepSurv           GBCS 47             deepsurv
8   DeepSurv            PBC 47             deepsurv
9   DeepSurv        Veteran 47             deepsurv
10  DeepSurv           WHAS 47             deepsurv
```

| | | | | |
|---|---|---|---|---|
| 11 | BPS Exp | ACTG | 47 | pmsurv_exponential |
| 12 | BPS Exp | GBCS | 47 | pmsurv_exponential |
| 13 | BPS Exp | PBC | 47 | pmsurv_exponential |
| 14 | BPS Exp | Veteran | 47 | pmsurv_exponential |
| 15 | BPS Exp | WHAS | 47 | pmsurv_exponential |
| 16 | BPS Wb | ACTG | 47 | pmsurv_weibull_linear |
| 17 | BPS Wb | GBCS | 47 | pmsurv_weibull_linear |
| 18 | BPS Wb | PBC | 47 | pmsurv_weibull_linear |
| 19 | BPS Wb | Veteran | 47 | pmsurv_weibull_linear |
| 20 | BPS Wb | WHAS | 47 | pmsurv_weibull_linear |
| 21 | BPS WbNN | ACTG | 47 | pmsurv_weibull_nn |
| 22 | BPS WbNN | GBCS | 47 | pmsurv_weibull_nn |
| 23 | BPS WbNN | PBC | 47 | pmsurv_weibull_nn |
| 24 | BPS WbNN | Veteran | 47 | pmsurv_weibull_nn |
| 25 | BPS WbNN | WHAS | 47 | pmsurv_weibull_nn |
| 26 | RSF | ACTG | 47 | rsf |
| 27 | RSF | GBCS | 47 | rsf |
| 28 | RSF | PBC | 47 | rsf |
| 29 | RSF | Veteran | 47 | rsf |
| 30 | RSF | WHAS | 47 | rsf |

## Results

### Graphical

```
ggboxplot(data = data, x = 'model_lbl', y = 'cindex', fill = 'model_lbl', facet.by = 'expe
          ylab = 'C-Index', xlab = 'Model', palette = 'lancet') +
  theme(axis.text.x = element_text(angle = 90, vjust = 0.5))
```

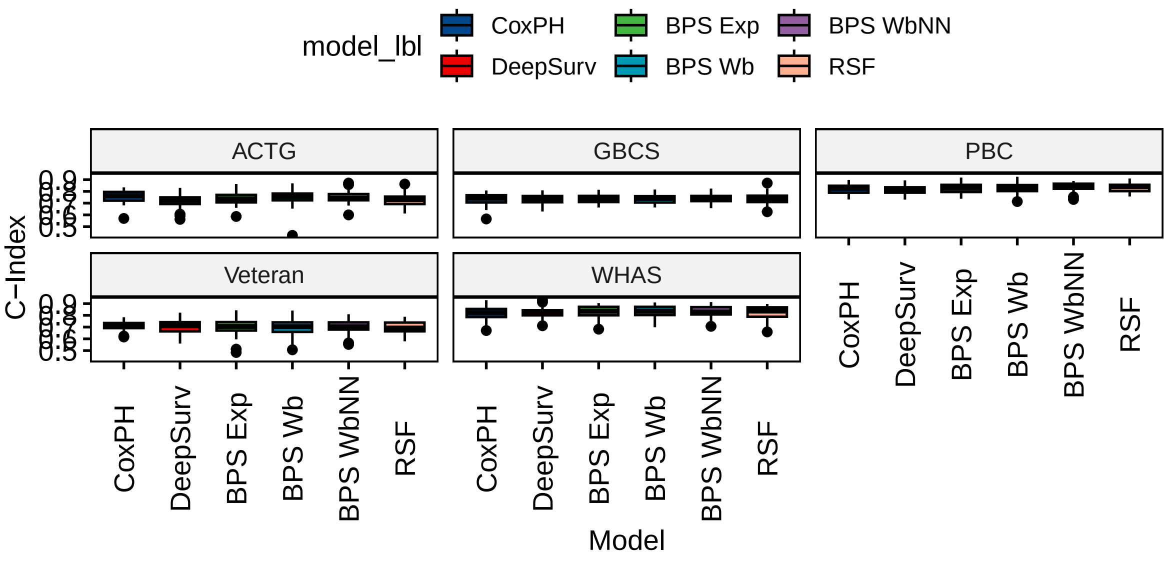

## Tabular

```
custom_mean_ci <- function(data, variable, ...) {
  mu <- mean(data[[variable]])
  margin <- qt(0.975, df=nrow(data) - 1) * sd(data[[variable]]) / sqrt(nrow(data))
  dplyr::tibble(
    mean = mu,
    conf.low = mu - margin,
    conf.high = mu + margin
  )
}

custom_median_ci <- function(data, variable, ...) {
  res <- wilcox.test(data$cindex, conf.int = TRUE)
  dplyr::tibble(
    mean = res$estimate,
    conf.low = res$conf.int[1],
    conf.high = res$conf.int[2]
  )
}

custom_mean_ci_bayes <- function(data, variable, ...) {
  model <- stan_glm(cindex ~ 1 ,
                    data = data ,
                    refresh = 0,
                    algorithm = 'sampling')

  model_hdi <- model_parameters(model, ci = 1-0.05/15)

  dplyr::tibble(
    mean = model_hdi$Median[1],
    conf.low = model_hdi$CI_low[1],
    conf.high = model_hdi$CI_high[1]
  )
}
```

### Bayesian

```
res.tbl <- data %>%
  select(model_lbl, experiment_lbl, cindex) %>%
  tbl_strata(strata = experiment_lbl,  ~.x %>%
    tbl_custom_summary(by = 'model_lbl',
                 type = list(
                   cindex ~ 'continuous'),
                 label = list(
                   cindex ~ 'C-Index'),
                 digits = everything() ~ 3,
                 stat_fns = ~ custom_mean_ci_bayes,
                 statistic = ~ "{mean} [{conf.low} - {conf.high}]"
                 ) ,
    .combine_with = 'tbl_stack'
  ) %>%
  modify_footnote(
    update = all_stat_cols() ~ "Mean [95% CI]"
  )

res.tbl
```

```
Table printed with `knitr::kable()`, not {gt}. Learn why at
https://www.danieldsjoberg.com/gtsummary/articles/rmarkdown.html
To suppress this message, include `message = FALSE` in code chunk header.
```

```
res.tbl %>% as_gt() %>% gtsave(filename = 'out/results_bayes.rtf')
```

```
comparisons <- data.frame('Model'=character(),
                          'Experiment'=character(),
                          'HDI_low'=double(),
                          'HDI_high'=double(),
                          'ROPE_percentage'=double(),
                          'ROPE_equivalence'=character())

for (idx_experiment in levels(data$experiment_lbl)){
  for (idx_model in levels(data$model_lbl)){
    model <- stan_glm(cindex ~ 1 ,
                 data = data %>%
                   filter(experiment_lbl == idx_experiment & model_lbl == idx_model),
```

Table 1: **?(caption)**

| **Group** | **Characteristic** | **CoxPH**, N = 47 | **DeepSurv**, N = 47 | **BPS Exp**, N = 47 | **BPS Wb**, N = 47 | **BPS WbNN**, N = 47 | **RSF**, N = 47 |
|---|---|---|---|---|---|---|---|
| ACTG | C-Index | 0.754 [0.731 - 0.778] | 0.714 [0.691 - 0.739] | 0.739 [0.715 - 0.763] | 0.745 [0.718 - 0.776] | 0.748 [0.724 - 0.770] | 0.729 [0.704 - 0.751] |
| GBCS | C-Index | 0.736 [0.715 - 0.757] | 0.731 [0.711 - 0.751] | 0.737 [0.721 - 0.756] | 0.735 [0.719 - 0.751] | 0.735 [0.718 - 0.753] | 0.737 [0.718 - 0.756] |
| PBC | C-Index | 0.820 [0.801 - 0.838] | 0.813 [0.796 - 0.829] | 0.825 [0.808 - 0.845] | 0.824 [0.805 - 0.843] | 0.842 [0.825 - 0.858] | 0.832 [0.814 - 0.851] |
| Veteran | C-Index | 0.709 [0.692 - 0.727] | 0.699 [0.674 - 0.724] | 0.700 [0.668 - 0.731] | 0.696 [0.665 - 0.729] | 0.704 [0.678 - 0.730] | 0.693 [0.671 - 0.715] |
| WHAS | C-Index | 0.817 [0.790 - 0.843] | 0.825 [0.804 - 0.845] | 0.828 [0.806 - 0.850] | 0.832 [0.811 - 0.853] | 0.834 [0.814 - 0.853] | 0.821 [0.794 - 0.848] |

```
                    refresh = 0,
                    algorithm = 'sampling')

      et <- equivalence_test(model,
                   range = c(-0.05, +0.05),
                   ci = 1 - 0.05 / 15
                   )
      comparisons <- rbind(comparisons, data.frame(
        'Model'=idx_model,
        'Experiment'=idx_experiment,
        'HDI_mean'=signif(model$coefficients['(Intercept)'], digits = 2),
        'HDI_low'=signif(et$HDI_low, digits = 2),
        'HDI_high'=signif(et$HDI_high, digits = 2),
        'ROPE_percentage'=round(et$ROPE_Percentage * 100, 1),
        'ROPE_equivalence'=et$ROPE_Equivalence
      ))
    }
  }
```

```
ggscatter(data = comparisons, x = 'Model', y = 'HDI_mean',
          facet.by = 'Experiment',
          color = 'Model', palette = 'lancet', ylab = 'C-Index') +
  #geom_hline(yintercept = c(0.01), color = 'black', linetype = 'dashed') +
  geom_errorbar(data = comparisons,
                aes(color = Model, ymin = HDI_low, ymax = HDI_high),
                width = 0.25) +
  theme(axis.text.x = element_text(angle = 90, vjust = 0.5))
```

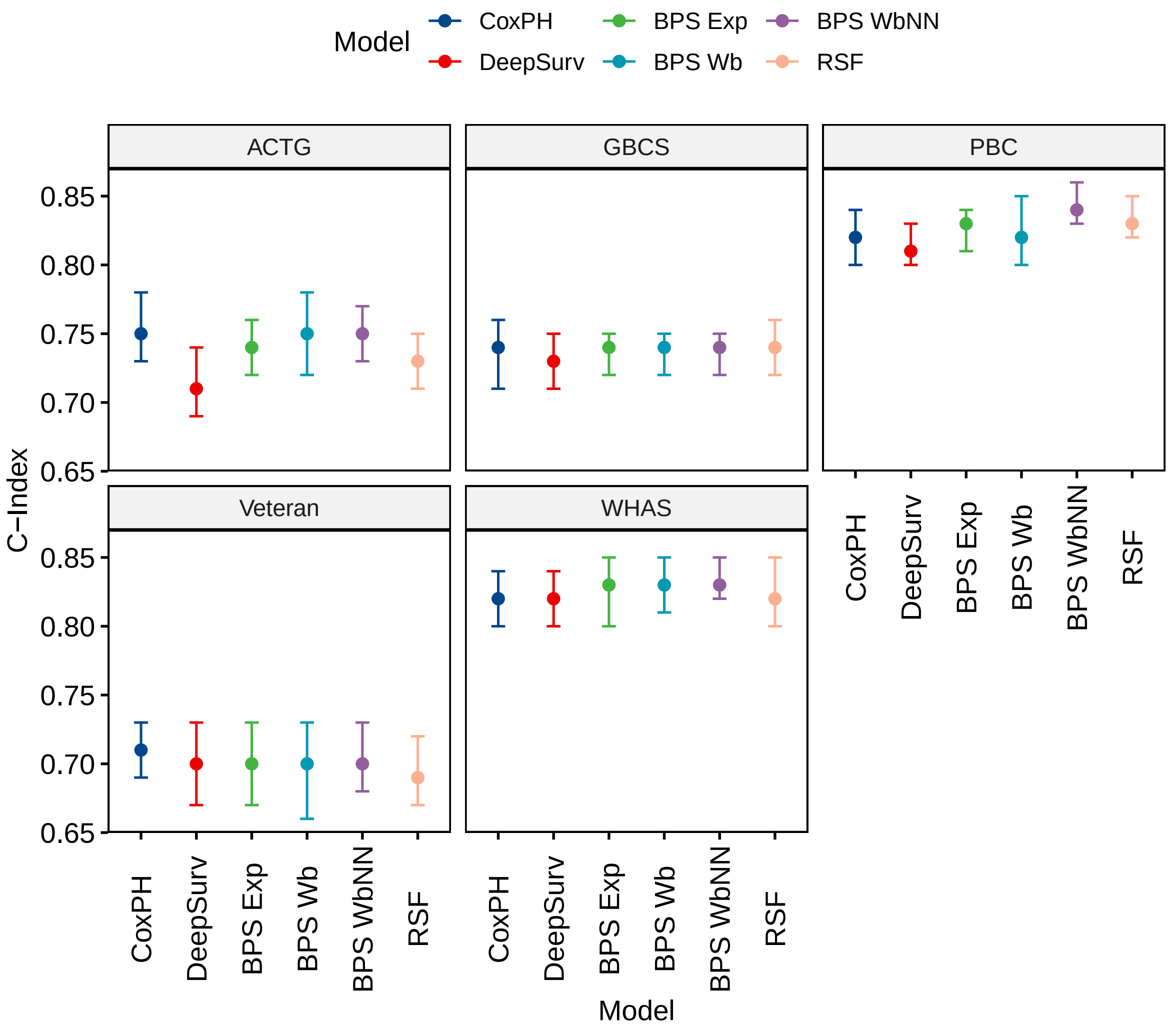

### Frequentist

```
res.tbl <- data %>%
  select(model_lbl, experiment_lbl, cindex) %>%
  tbl_strata(strata = experiment_lbl,  ~.x %>%
    tbl_custom_summary(by = 'model_lbl',
                type = list(
                  cindex ~ 'continuous'),
                label = list(
                  cindex ~ 'C-Index'),
                digits = everything() ~ 3,
                stat_fns = ~ custom_mean_ci,
                statistic = ~ "{mean} [{conf.low} - {conf.high}]"
                ) ,
    .combine_with = 'tbl_stack'
  ) %>%
  modify_footnote(
    update = all_stat_cols() ~ "Mean [95% CI]"
  )

res.tbl
```

```
Table printed with `knitr::kable()`, not {gt}. Learn why at
https://www.danieldsjoberg.com/gtsummary/articles/rmarkdown.html
To suppress this message, include `message = FALSE` in code chunk header.
```

```
res.tbl %>% as_gt() %>% gtsave(filename = 'out/results.rtf')
```

## Diagnostics

### Check distributionof C-Indexes

Compute Shapiro test for C-index on each experiment/model combination.

```
data %>%
  group_by(model_lbl, experiment_lbl) %>%
  summarise(n = n(),
            shap_test = round(shapiro.test(cindex)$p.value, 3),
```

Table 3: **?(caption)**

| **Group** | **Characteristic** | **CoxPH**, N = 47 | **DeepSurv**, N = 47 | **BPS Exp**, N = 47 | **BPS Wb**, N = 47 | **BPS WbNN**, N = 47 | **RSF**, N = 47 |
|---|---|---|---|---|---|---|---|
| ACTG | C-Index | 0.754 [0.739 - 0.769] | 0.715 [0.699 - 0.730] | 0.739 [0.724 - 0.755] | 0.745 [0.726 - 0.764] | 0.748 [0.733 - 0.762] | 0.729 [0.714 - 0.744] |
| GBCS | C-Index | 0.736 [0.722 - 0.749] | 0.731 [0.719 - 0.744] | 0.737 [0.726 - 0.748] | 0.735 [0.724 - 0.746] | 0.735 [0.725 - 0.746] | 0.737 [0.724 - 0.750] |
| PBC | C-Index | 0.820 [0.808 - 0.833] | 0.813 [0.801 - 0.824] | 0.825 [0.813 - 0.838] | 0.824 [0.811 - 0.837] | 0.842 [0.831 - 0.853] | 0.833 [0.821 - 0.844] |
| Veteran | C-Index | 0.709 [0.698 - 0.720] | 0.699 [0.682 - 0.717] | 0.700 [0.680 - 0.721] | 0.696 [0.675 - 0.717] | 0.704 [0.687 - 0.721] | 0.693 [0.678 - 0.707] |
| WHAS | C-Index | 0.817 [0.801 - 0.833] | 0.824 [0.811 - 0.838] | 0.828 [0.813 - 0.843] | 0.831 [0.817 - 0.846] | 0.834 [0.821 - 0.847] | 0.821 [0.804 - 0.838] |

```
is_normal = (shapiro.test(cindex)$p.value > 0.05)
)
```

```
`summarise()` has grouped output by 'model_lbl'. You can override using the
`.groups` argument.
```

```
# A tibble: 30 x 5
# Groups:   model_lbl [6]
   model_lbl experiment_lbl     n shap_test is_normal
   <fct>     <fct>          <int>     <dbl> <lgl>
 1 CoxPH     ACTG              47     0.009 FALSE
 2 CoxPH     GBCS              47     0.004 FALSE
 3 CoxPH     PBC               47     0.279 TRUE
 4 CoxPH     Veteran           47     0.275 TRUE
 5 CoxPH     WHAS              47     0.372 TRUE
 6 DeepSurv  ACTG              47     0.021 FALSE
 7 DeepSurv  GBCS              47     0.341 TRUE
 8 DeepSurv  PBC               47     0.76  TRUE
```

```
 9 DeepSurv  Veteran           47     0.485 TRUE
10 DeepSurv  WHAS              47     0.089 TRUE
# ... with 20 more rows
```

There are a few cases where the C-Indexes are not normally distributed.
