# Supplementary material for: Bayesian parametric models for survival prediction in medical applications
Source: BMC Med Res Methodol. 2023 Oct 26;23:250. doi: 10.1186/s12874-023-02059-4 (PMC10605790; doi:10.1186/s12874-023-02059-4)
Supplement: Supplementary file 2 — Additional file 2. Overlap of posterior distributions. [file 12874_2023_2059_MOESM2_ESM.pdf]

Bayesian parametric models for survival prediction in medical applications

Overlap of posterior distributions

Iwan Paolucci, PhD

7/3/23

Table of contents

Setup 2

Load data 2

Functions 3

WHAS 4

GBCS 5

PBC 7

ACTG 9

Setup

```r
library(Cairo)
library(ggplot2)
library(ggpubr)
library(ggsci)
library(dplyr)
```

```
Attaching package: 'dplyr'

The following objects are masked from 'package:stats':

    filter, lag

The following objects are masked from 'package:base':

    intersect, setdiff, setequal, union
```

```r
library(stringr)
```

```r
knitr::opts_chunk$set(dev.args = list(png = list(type = "cairo")),
                      fig.path='out/figs/ovi/', dev = c('pdf', 'png'), dpi = 300)
```

Load data

```r
load(file = 'data/ovi.rds')
```

```r
data$experiment_lbl <- toupper(data$experiment_lbl)

data$model_lbl <- factor(data$model_lbl, labels = c('BPS Exponential', 'BPS Weibull'))
```

Functions

```
plot_ovi <- function(.data, experiment_str, model_str){

  data.grouped <- .data %>%
    filter(experiment == experiment_str) %>%
    mutate(
      var_lbl = factor(str_sub(var, 8, -1)),
      experiment_lbl = toupper(experiment_lbl)
    ) %>%
    group_by(experiment_lbl, model_lbl, var_lbl, partition) %>%
    summarise(
      n = n(),
      median = median(ovi),
      median_lower = wilcox.test(ovi, conf.int = TRUE)$conf.int[1],
      median_upper = wilcox.test(ovi, conf.int = TRUE)$conf.int[2],
      model = first(model)
    )

  data_filtered <- data.grouped %>% filter(model == model_str)

  plt.all <- ggline(data = data_filtered,
                   x = 'partition', y = 'median', color = 'var_lbl', group = 'var_lbl',
              facet.by = c('experiment_lbl', 'model_lbl'),
              add.params = list(color = "var_lbl", size = 1, width = 0.1),
              palette = 'hls', size = 1, plot_type = 'b', alpha = 0.5) +
    geom_ribbon(data = data_filtered, aes(ymin = median_lower, ymax = median_upper,
                                          fill = var_lbl), alpha=0.2, outline.type = 'bot

  plt.all <- ggpar(plt.all, ylab = 'OVI', xlab = 'Partition', legend.title = "Variable")

  return (plt.all)
}
```

WHAS

```
data.whas <- data %>%
  filter(!var %in% c('lambda_yrgrp_1975 & 1978', 'lambda_yrgrp_1981 & 1984', 'lambda_yrgrp
```

```
plot_ovi(data.whas, 'whas', 'pm_exp')
```

```
`summarise()` has grouped output by 'experiment_lbl', 'model_lbl', 'var_lbl'.
You can override using the `.groups` argument.
```

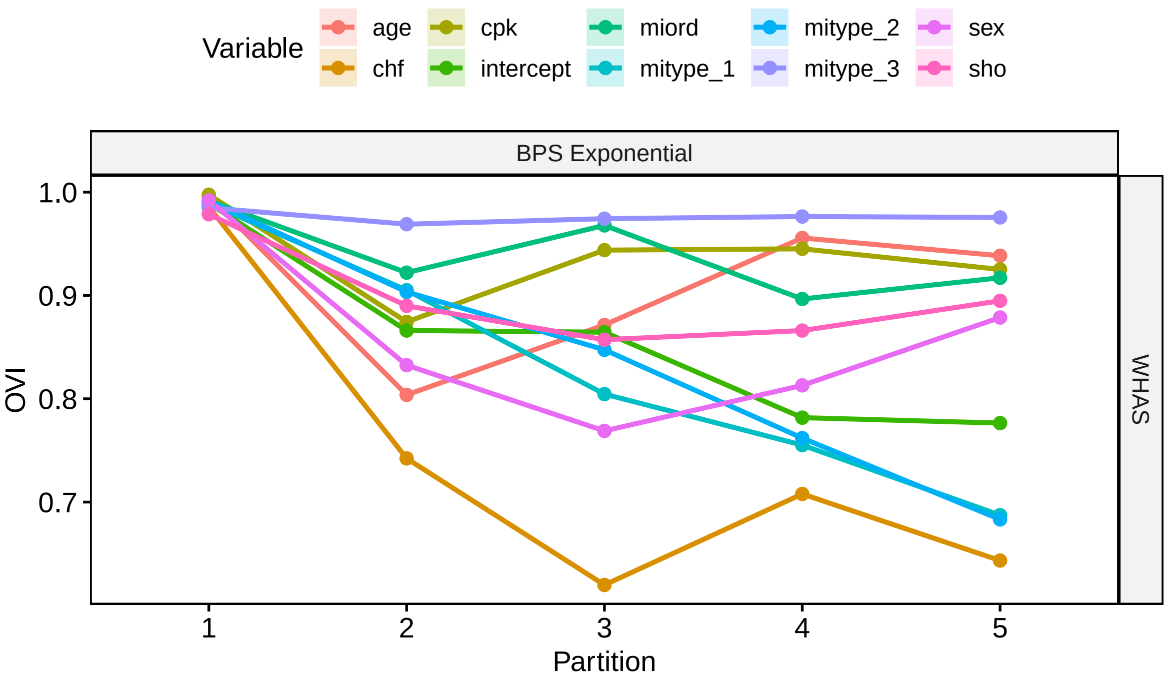


```
plot_ovi(data.whas, 'whas', 'pm_wb')
```

```
`summarise()` has grouped output by 'experiment_lbl', 'model_lbl', 'var_lbl'.
You can override using the `.groups` argument.
```

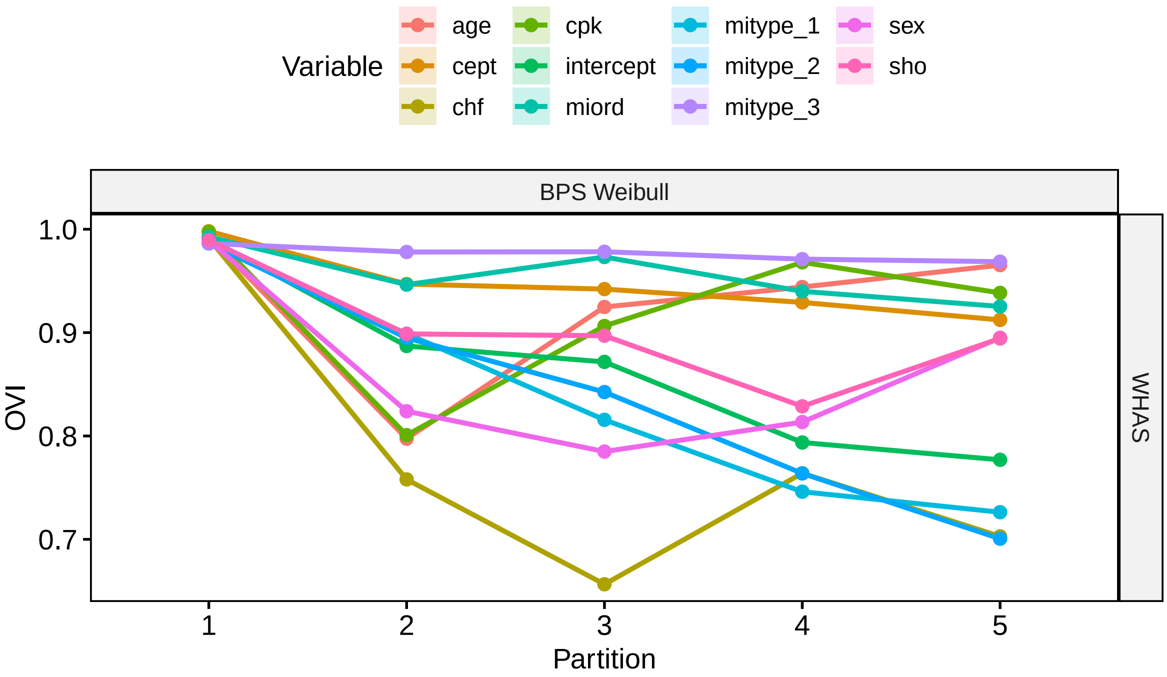

GBCS

```
plot_ovi(data, 'gbcs', 'pm_exp')
```

```
`summarise()` has grouped output by 'experiment_lbl', 'model_lbl', 'var_lbl'.
You can override using the `.groups` argument.
```

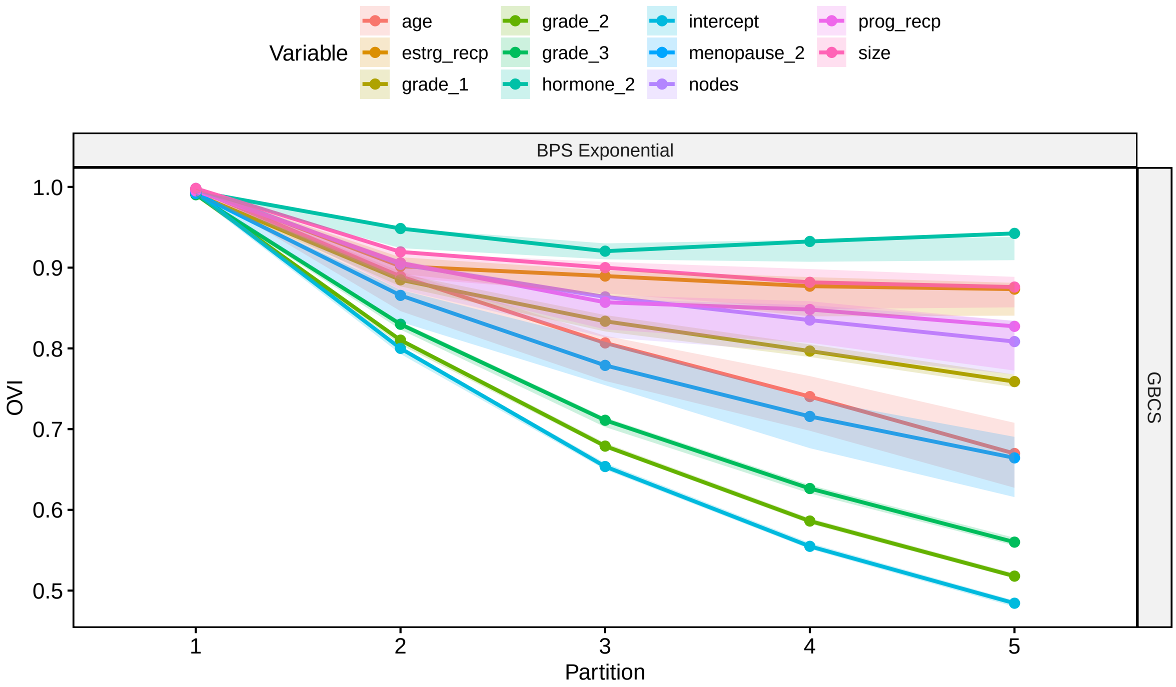


```
plot_ovi(data, 'gbcs', 'pm_wb')
```

```
`summarise()` has grouped output by 'experiment_lbl', 'model_lbl', 'var_lbl'.
You can override using the `.groups` argument.
```

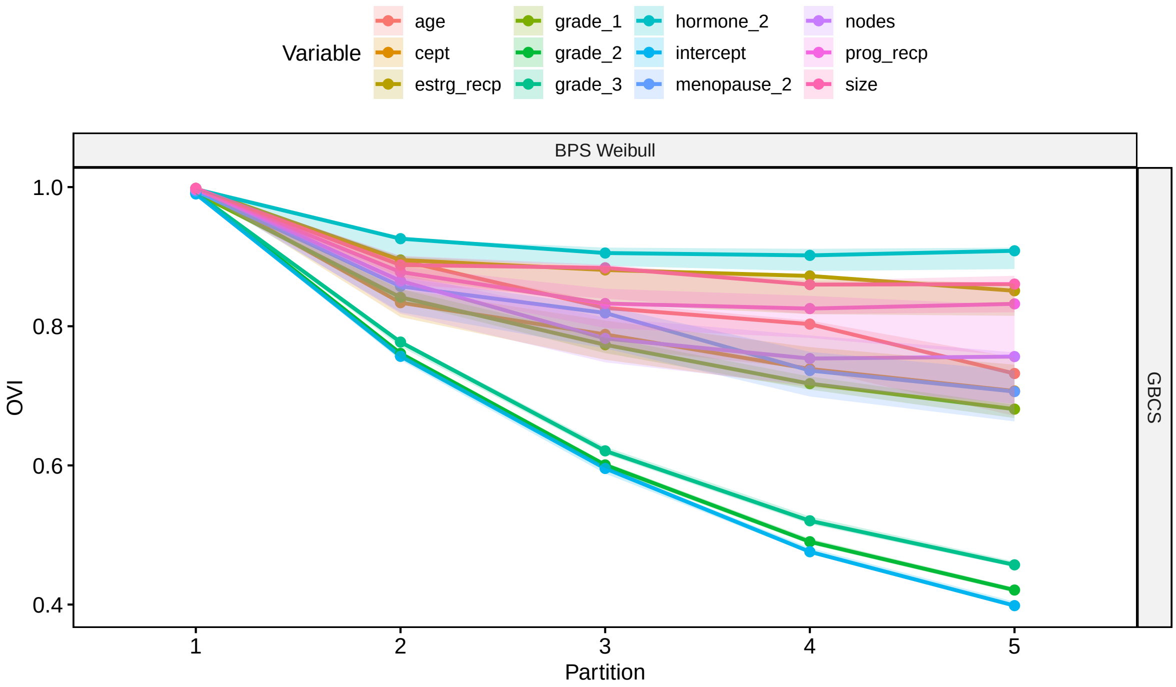

PBC

```
plot_ovi(data, 'pbc', 'pm_exp')
```

```
`summarise()` has grouped output by 'experiment_lbl', 'model_lbl', 'var_lbl'.
You can override using the `.groups` argument.
```

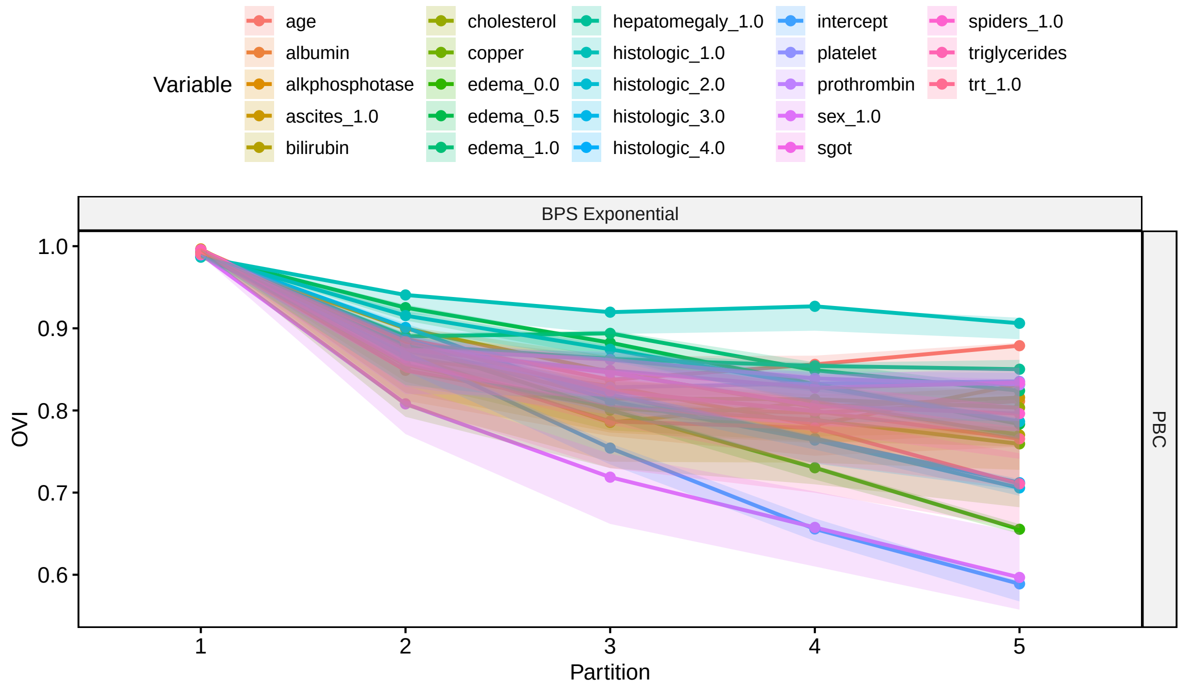


```
plot_ovi(data, 'pbc', 'pm_wb')
```

```
`summarise()` has grouped output by 'experiment_lbl', 'model_lbl', 'var_lbl'.
You can override using the `.groups` argument.
```

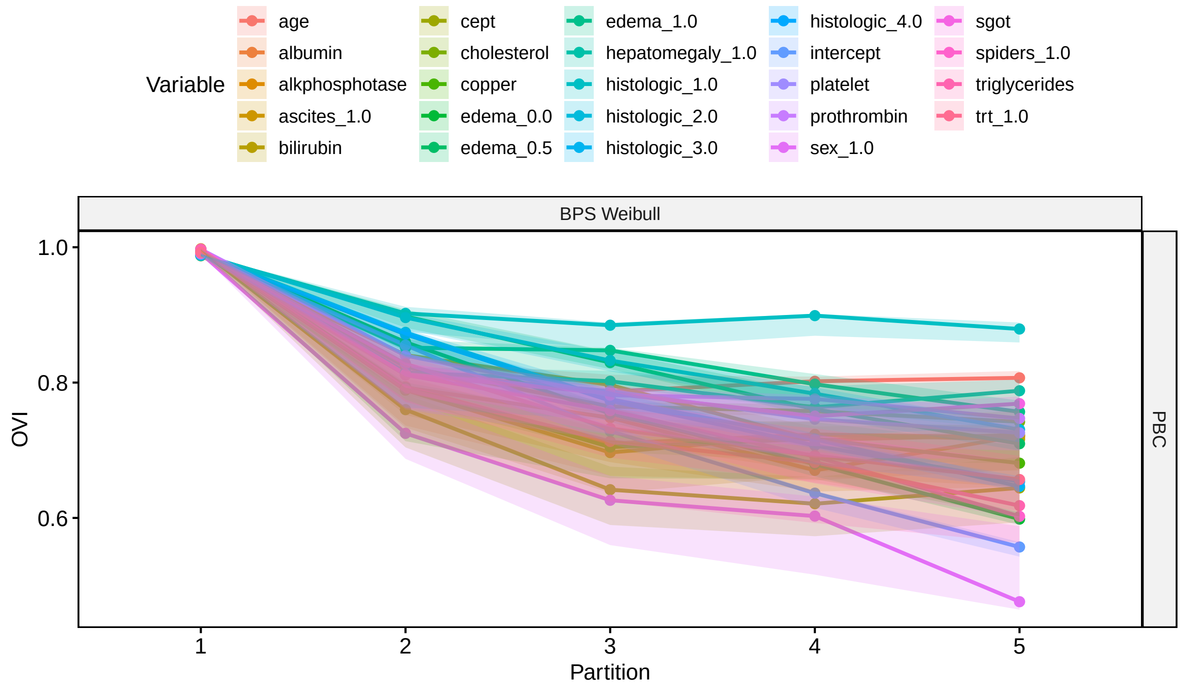

ACTG

```
plot_ovi(data, 'actg', 'pm_exp')
```

```
`summarise()` has grouped output by 'experiment_lbl', 'model_lbl', 'var_lbl'.
You can override using the `.groups` argument.
```

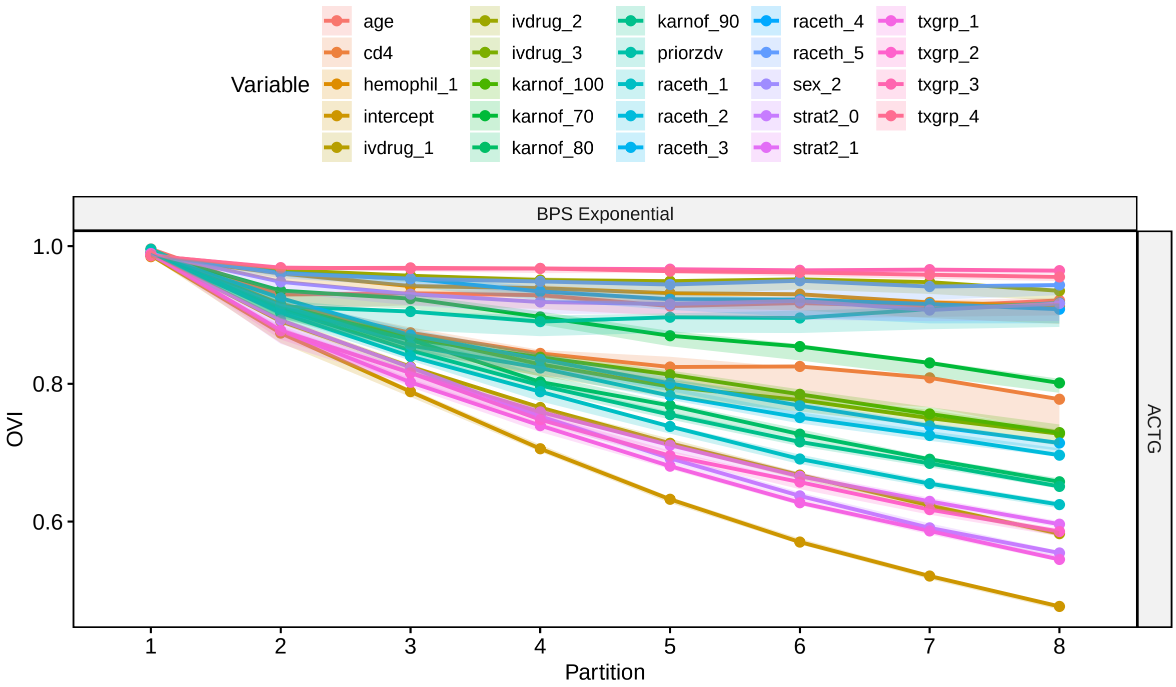


```r
plot_ovi(data, 'actg', 'pm_wb')
```

```
`summarise()` has grouped output by 'experiment_lbl', 'model_lbl', 'var_lbl'.
You can override using the `.groups` argument.
```

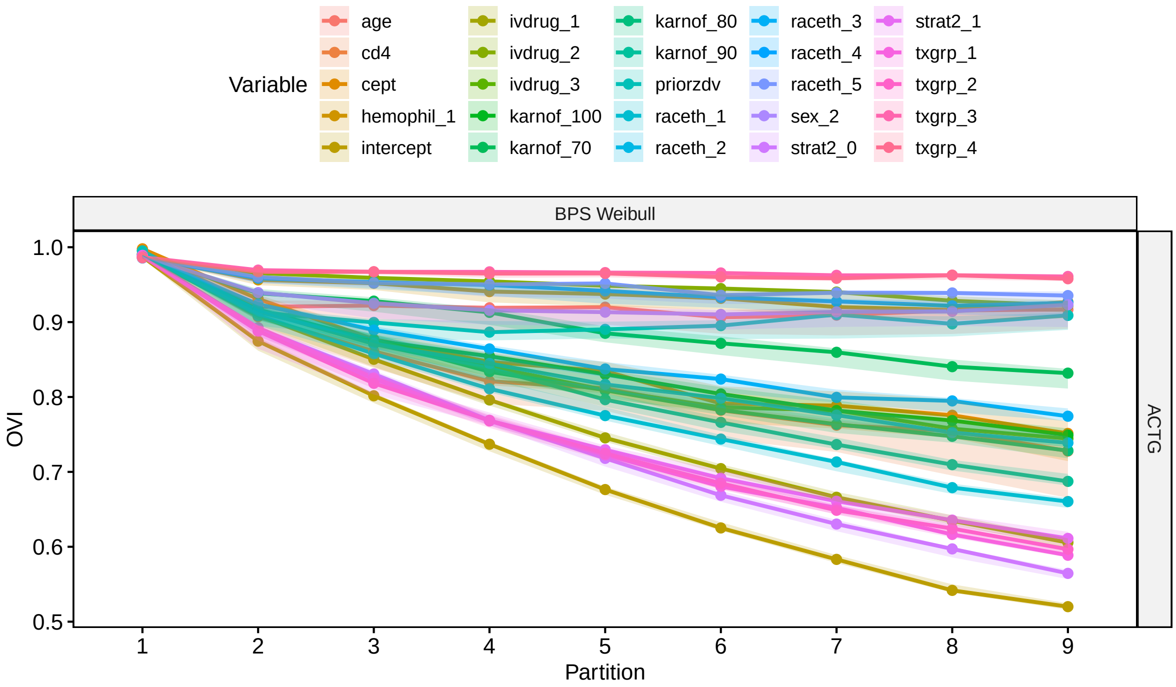

Variable
age
cd4
cept
hemophil_1
intercept
ivdrug_1
ivdrug_2
ivdrug_3
karnof_100
karnof_70
karnof_80
karnof_90
priorzdv
raceth_1
raceth_2
raceth_3
raceth_4
raceth_5
sex_2
strat2_0
strat2_1
txgrp_1
txgrp_2
txgrp_3
txgrp_4
BPS Weibull
ACTG
OVI
1.0
0.9
0.8
0.7
0.6
0.5
1
2
3
4
5
6
7
8
9
Partition
