# Supplementary material for: Bayesian parametric models for survival prediction in medical applications
Source: BMC Med Res Methodol. 2023 Oct 26;23:250. doi: 10.1186/s12874-023-02059-4 (PMC10605790; doi:10.1186/s12874-023-02059-4)
Supplement: Supplementary file 3 — Additional file 3. Posterior distributions. [file 12874_2023_2059_MOESM3_ESM.pdf]

Bayesian parametric models for survival prediction in medical applications

Visualization posterior distributions

Iwan Paolucci, PhD

12/2/22

Table of contents

Load data **2**

Exponential **4**
WHAS 4
GBCS 12
PBC 23
ACTG 47

Weibull **71**
WHAS 71
GBCS 83
PBC 95
ACTG 120

```r
library(Cairo)
library(ggplot2)
library(ggpubr)
library(ggsci)
library(dplyr)
```

```
Attaching package: 'dplyr'

The following objects are masked from 'package:stats':

    filter, lag
```

```
The following objects are masked from 'package:base':

    intersect, setdiff, setequal, union
```

```
library(stringr)
```

```
knitr::opts_chunk$set(dev.args = list(png = list(type = "cairo")),
                      fig.path='out/figs/posterior/', dev = c('pdf', 'png'), dpi = 300)
```

Load data

```
redownload <- FALSE

if (redownload){
  src_folder <- 'U:/src/pymc-survival/examples/experiments/results_retrain'
  file <- "posterior_5.csv"

  data.exp <- rbind(
    read.csv(paste(src_folder, 'pm_exp_gbcs', file, sep = '/')) %>% mutate(experiment = 'g
    read.csv(paste(src_folder, 'pm_exp_whas', file, sep = '/')) %>% mutate(experiment = 'w
    read.csv(paste(src_folder, 'pm_exp_pbc', file, sep = '/')) %>% mutate(experiment = 'pb
    read.csv(paste(src_folder, 'pm_exp_aids', file, sep = '/')) %>% mutate(experiment = 'a
  )


  data.wb <- rbind(
    read.csv(paste(src_folder, 'pm_wb_gbcs', file, sep = '/')) %>% mutate(experiment = 'gb
    read.csv(paste(src_folder, 'pm_wb_whas', file, sep = '/')) %>% mutate(experiment = 'wh
    read.csv(paste(src_folder, 'pm_wb_pbc', file, sep = '/')) %>% mutate(experiment = 'pbc
    read.csv(paste(src_folder, 'pm_wb_aids', file, sep = '/')) %>% mutate(experiment = 'ac
  )

  data <- rbind(data.exp, data.wb)

  data <- data %>%
    mutate(
      experiment_lbl = as.factor(experiment),
      model_lbl = as.factor(model)
    )
```

```
  save(data, file = 'data/posteriors.rds')
}
```

```
load('data/posteriors.rds')
```

```
data <- data %>%
  mutate(
    posterior_samples = posterior_sampled,
    posterior_type_lbl = factor(posterior_type, labels = c('Full', 'Retrain'))
  ) %>%
  select(-posterior, -posterior_sampled)

data <- data %>%
  filter((!var %in% c('lambda_yrgrp_1975 & 1978', 'lambda_yrgrp_1981 & 1984', 'lambda_yrgr
```

```
plot_density <- function(data, experiment_str, model_str, var_str){

  data.filter <- data %>%
    filter(experiment == experiment_str & model == model_str) %>%
    mutate(partition_lbl = factor(partition,
                                  labels = paste("P", unique(partition), sep = "")),
           var_lbl = factor(str_replace(var, 'lambda_', '')))
  #
  # data.filter$partition_lbl = factor(data.filter$partition,
  #                                    labels = paste("P", unique(data.filter$partition),

  p1 <- ggdensity(data = data.filter %>% filter(var == var_str) ,
          x = 'posterior_samples', y = 'density',
          add = 'median',
          color = 'partition_lbl', size = 1,
          fill = 'partition_lbl', alpha = 0.005,
          facet.by = c('var_lbl', 'posterior_type_lbl' ),
          ylab = 'Density', xlab = 'Posterior samples',
          palette = get_palette(c("orange", "red"), length(levels(data.filter$partition_lb
    labs(fill = "Partition", color = 'Partition')
  return (p1)
}

plot_density_2 <- function(data, experiment_str, model_str, var_str, add_normal=FALSE) {
```

```
  data.filter <- data %>%
    filter(experiment == experiment_str & model == model_str) %>%
  mutate(
    partition_lbl = factor(partition,
                           labels = paste("P", unique(partition), sep = "")),
    var_lbl = factor(str_replace(var, 'lambda_', ''))
  )

  p <- ggdensity(data = data.filter %>% filter(var == var_str) ,
            x = 'posterior_samples', y = 'density',
            add = 'median',
            color = 'posterior_type_lbl', size = 1,
            fill = 'posterior_type_lbl', alpha = 0.1,
            facet.by = c('var_lbl', 'partition_lbl' ),
            palette = 'npg',
            ylab = 'Density', xlab = 'Posterior samples') +
    labs(fill = "Training type", color = 'Training type')

  if (add_normal) {
    norm_dens <- data.frame(samples = rnorm(100))
    p <- p + stat_overlay_normal_density(
              data = norm_dens, aes(x = samples),
              linetype = "dashed" )
  }

  return(p)
}
```

Exponential

WHAS

```
whas_vars <- data %>% filter(experiment == 'whas' & model == 'pm_exp') %>% select(var) %>%

for(var_idx in unique(whas_vars$var)){
  p1 <- plot_density(data, 'whas', 'pm_exp', var_idx)
  plot(p1)

  p <- plot_density_2(data, 'whas', 'pm_exp', var_idx)
```

```
  plot(p)

}
```

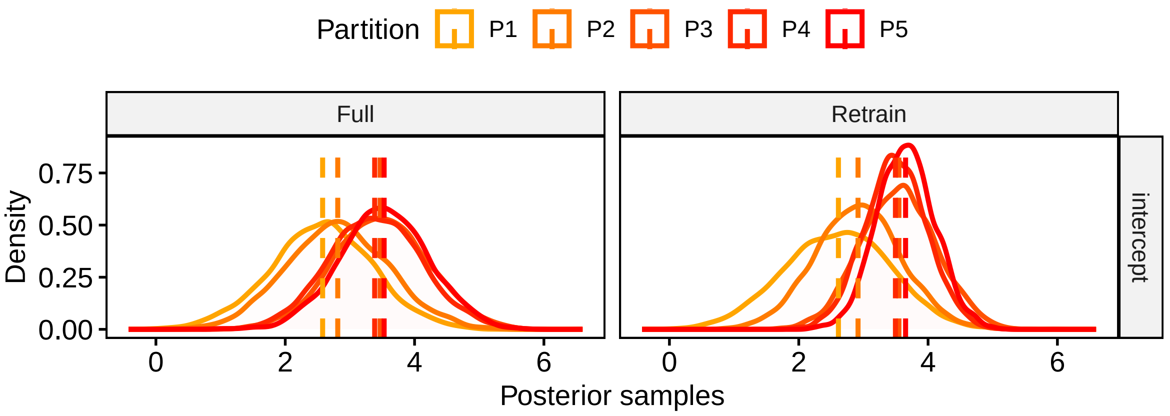

Partition P1 P2 P3 P4 P5
Full
Retrain
intercept
Density
0.75
0.50
0.25
0.00
0
2
4
6
Posterior samples

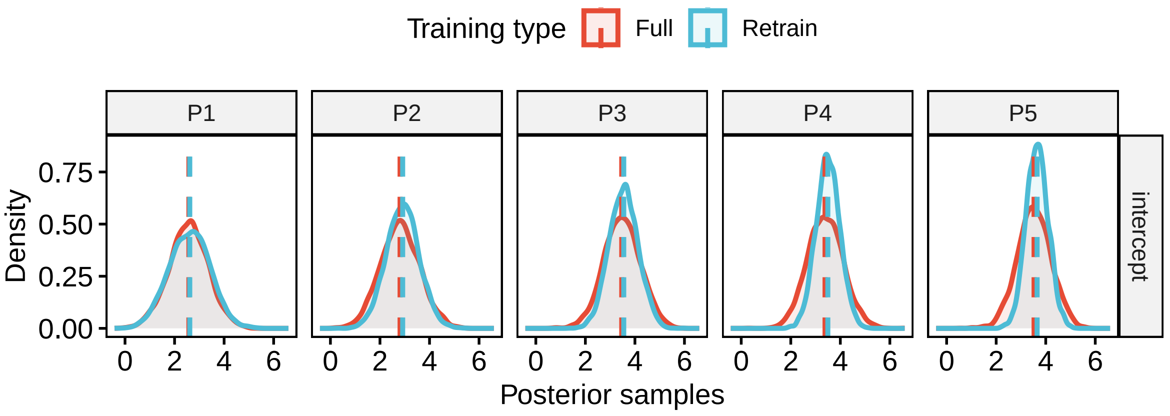

Training type Full Retrain
P1
P2
P3
P4
P5
intercept
Density
0.75
0.50
0.25
0.00
0
2
4
6
Posterior samples

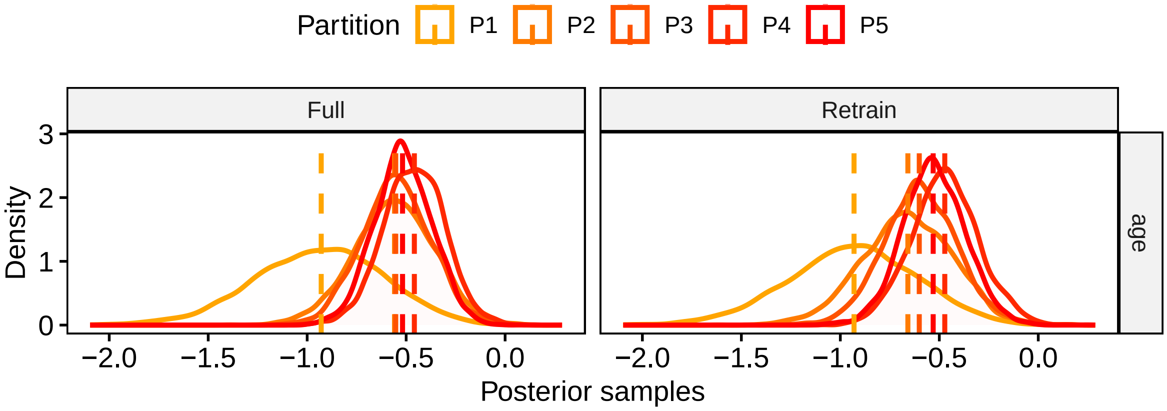
Partition
P1
P2
P3
P4
P5
Full
Retrain
age
Density
3
2
1
0
−2.0
−1.5
−1.0
−0.5
0.0
Posterior samples

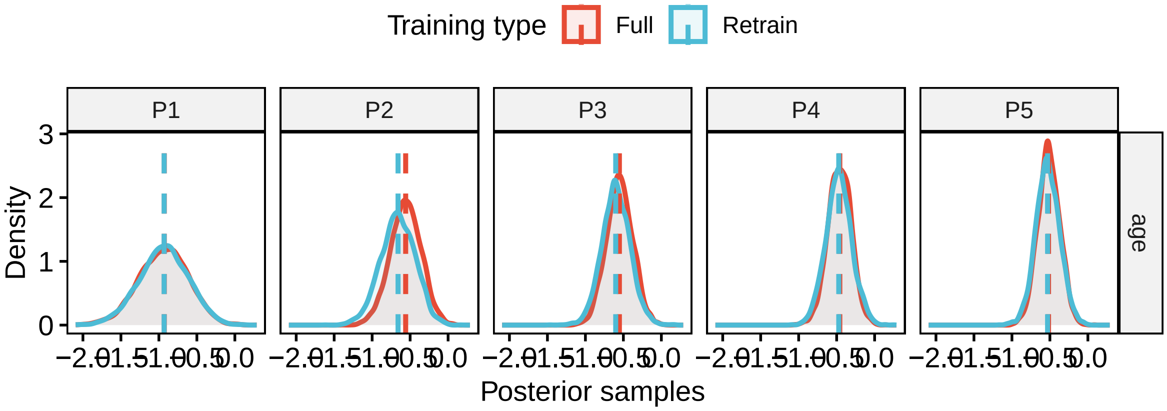
Training type
Full
Retrain
P1
P2
P3
P4
P5
age
Density
3
2
1
0
Posterior samples

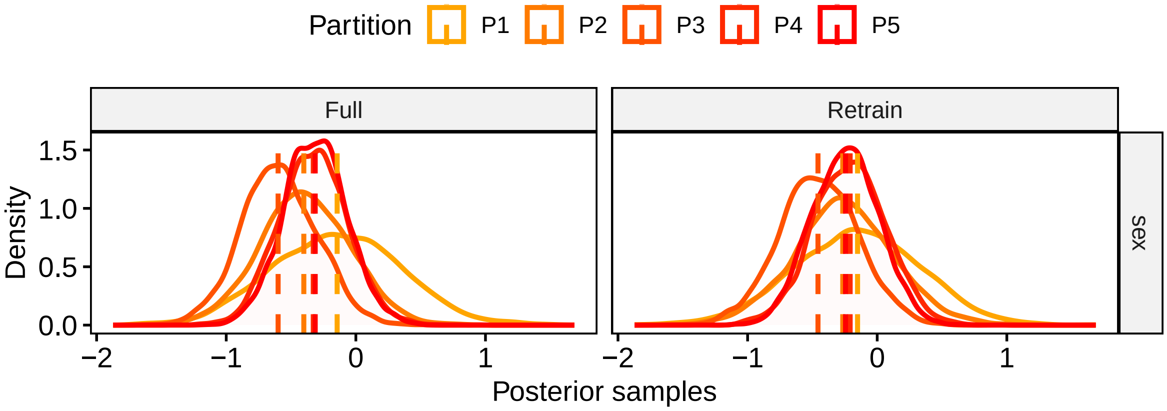
Partition
P1
P2
P3
P4
P5
Full
Retrain
sex
Density
1.5
1.0
0.5
0.0
−2
−1
0
1
Posterior samples

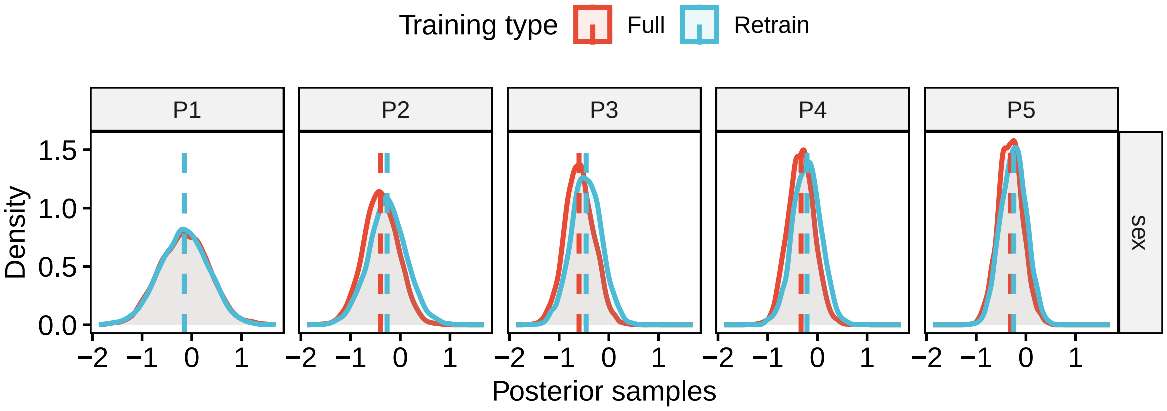
Training type
Full
Retrain
P1
P2
P3
P4
P5
sex
Density
1.5
1.0
0.5
0.0
−2 −1 0 1
Posterior samples

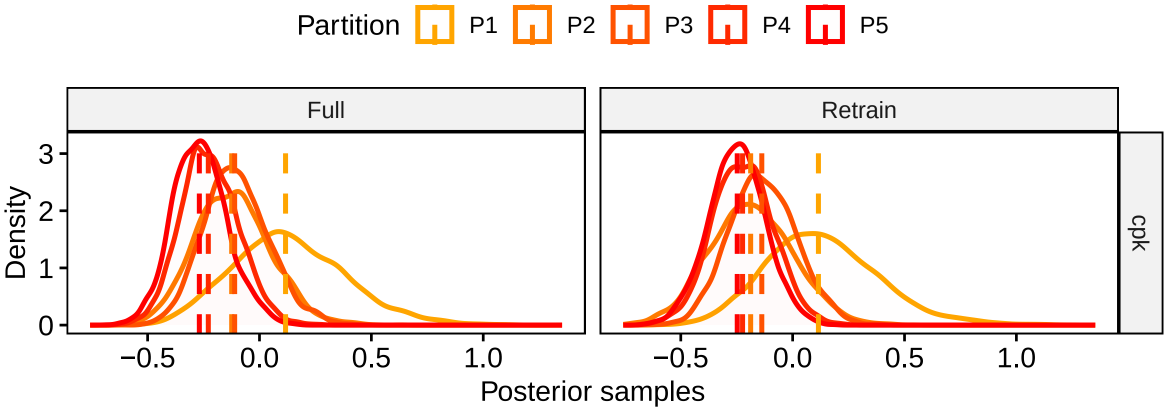
Partition
P1
P2
P3
P4
P5
Full
Retrain
cpk
Density
3
2
1
0
−0.5 0.0 0.5 1.0
Posterior samples

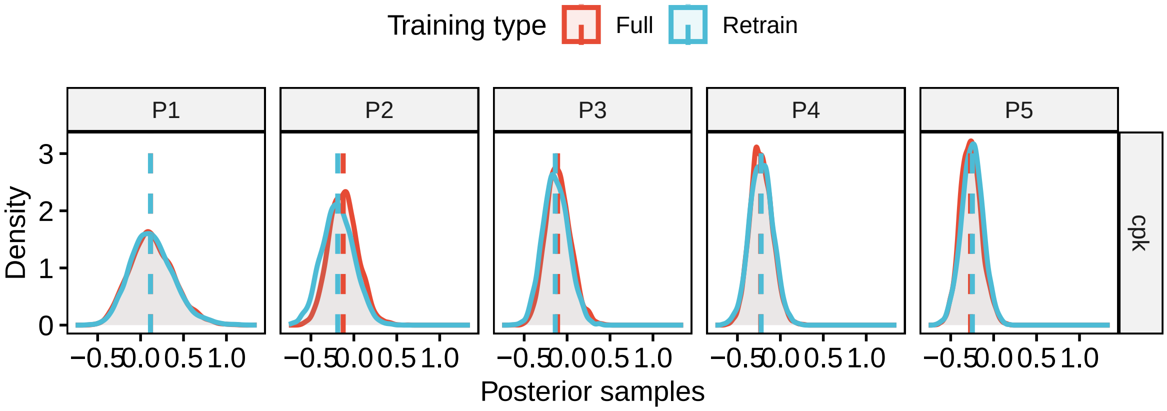
Training type
Full
Retrain
P1
P2
P3
P4
P5
cpk
Density
3
2
1
0
−0.5 0.0 0.5 1.0
Posterior samples

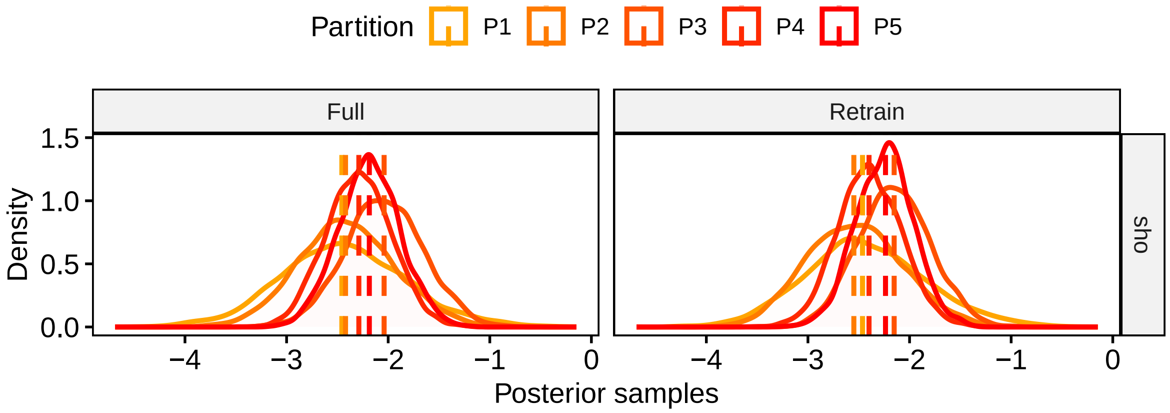
Partition
P1
P2
P3
P4
P5
Full
Retrain
sho
Density
Posterior samples

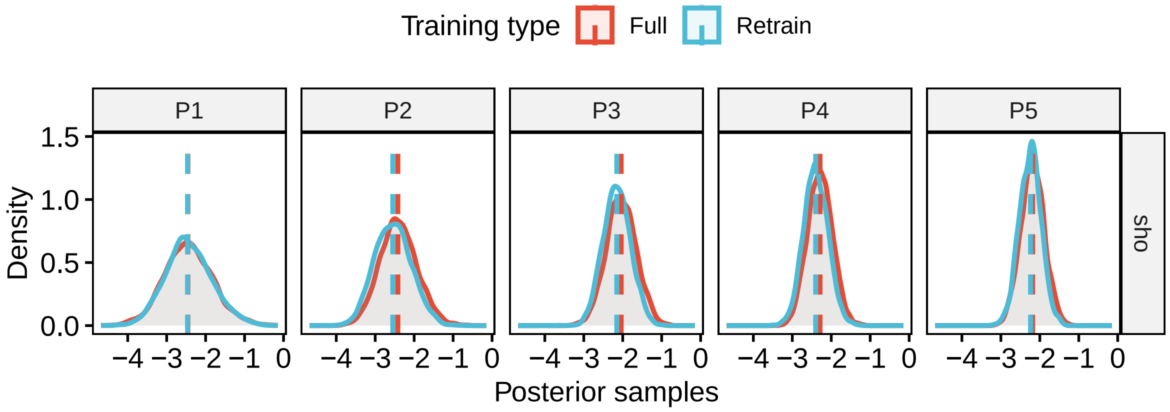
Training type
Full
Retrain
P1
P2
P3
P4
P5
sho
Density
Posterior samples

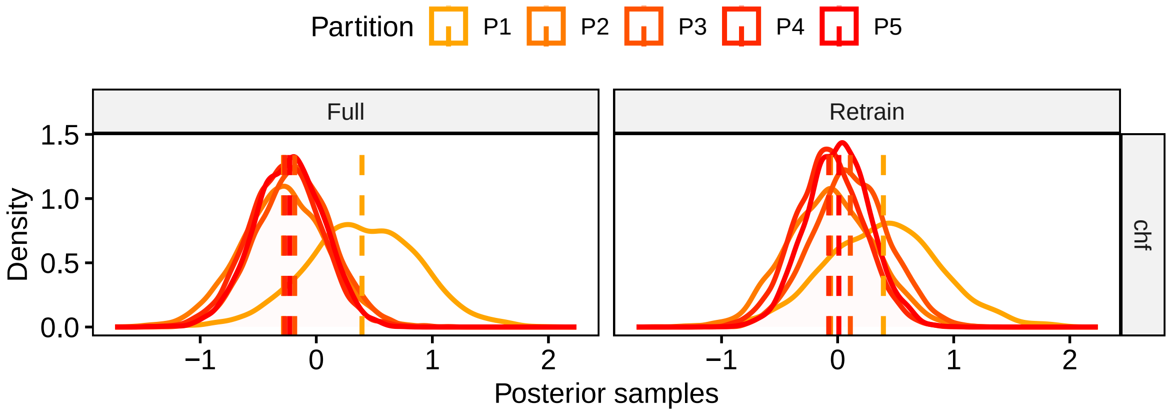
Partition
P1
P2
P3
P4
P5
Full
Retrain
chf
Density
Posterior samples

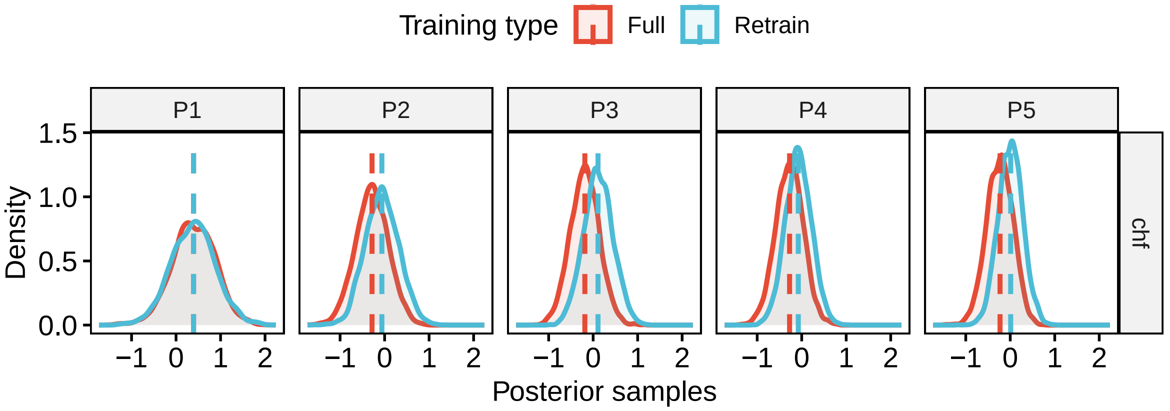
Training type
Full
Retrain
P1
P2
P3
P4
P5
chf
Density
1.5
1.0
0.5
0.0
−1
0
1
2
Posterior samples

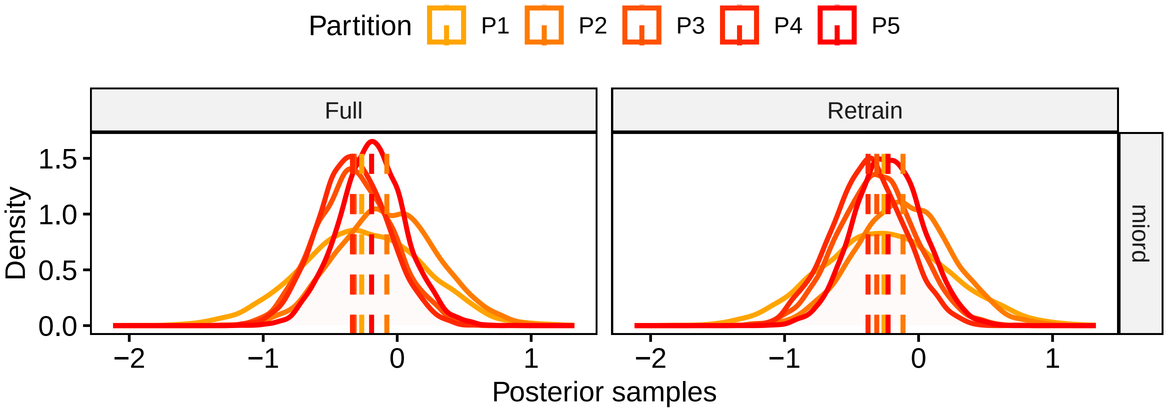
Partition
P1
P2
P3
P4
P5
Full
Retrain
miord
Density
1.5
1.0
0.5
0.0
−2
−1
0
1
Posterior samples

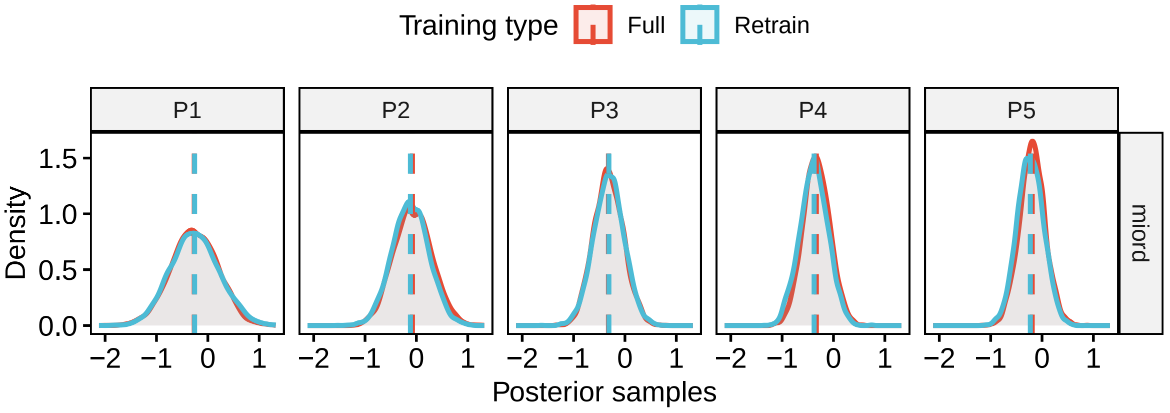
Training type
Full
Retrain
P1
P2
P3
P4
P5
miord
Density
1.5
1.0
0.5
0.0
−2
−1
0
1
Posterior samples

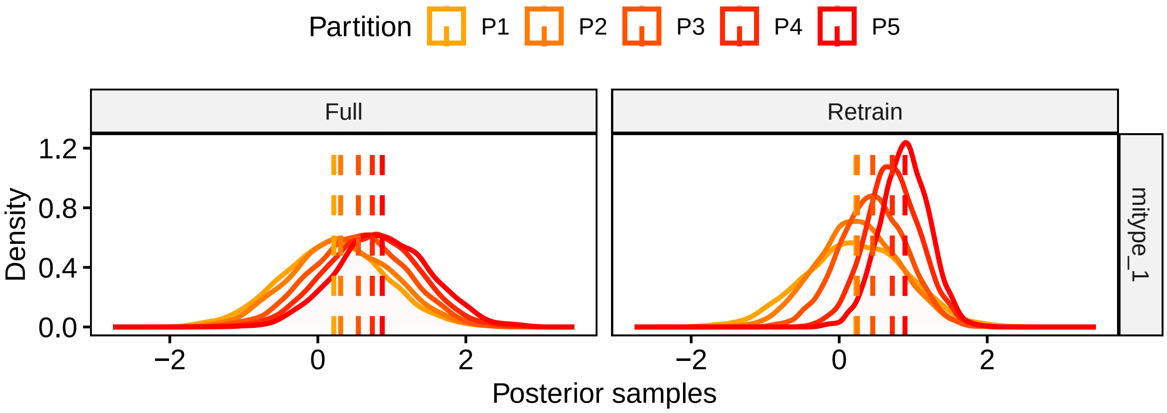
Partition
P1
P2
P3
P4
P5
Full
Retrain
mitype_1
Density
1.2
0.8
0.4
0.0
−2
0
2
Posterior samples

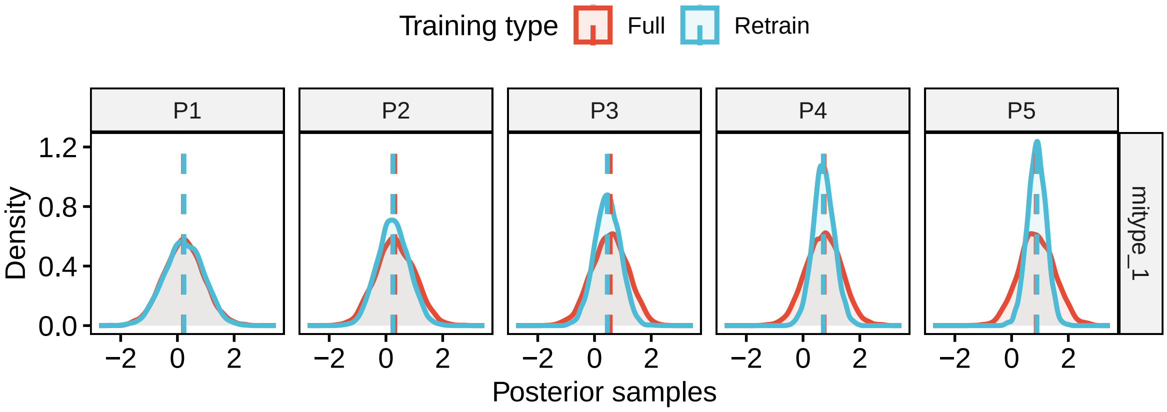
Training type
Full
Retrain
P1
P2
P3
P4
P5
mitype_1
Density
1.2
0.8
0.4
0.0
−2
0
2
Posterior samples

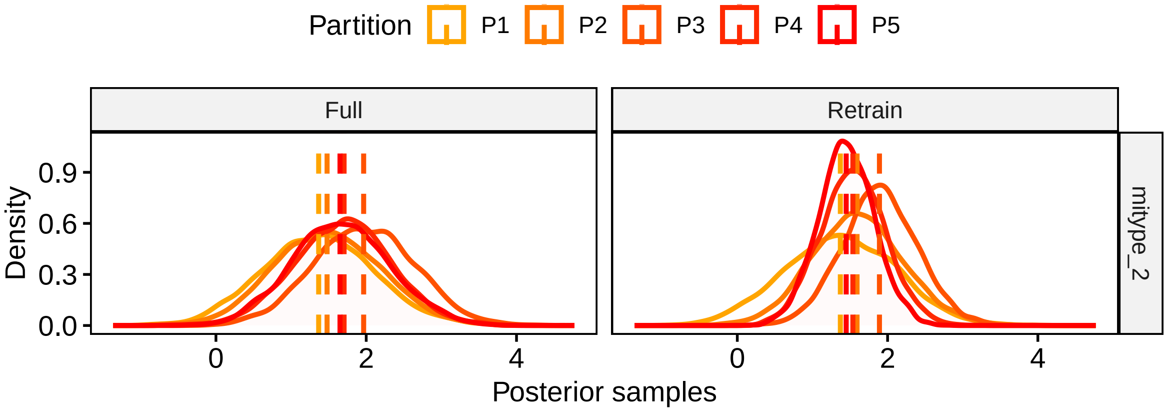
Partition
P1
P2
P3
P4
P5
Full
Retrain
mitype_2
Density
0.9
0.6
0.3
0.0
0
2
4
Posterior samples

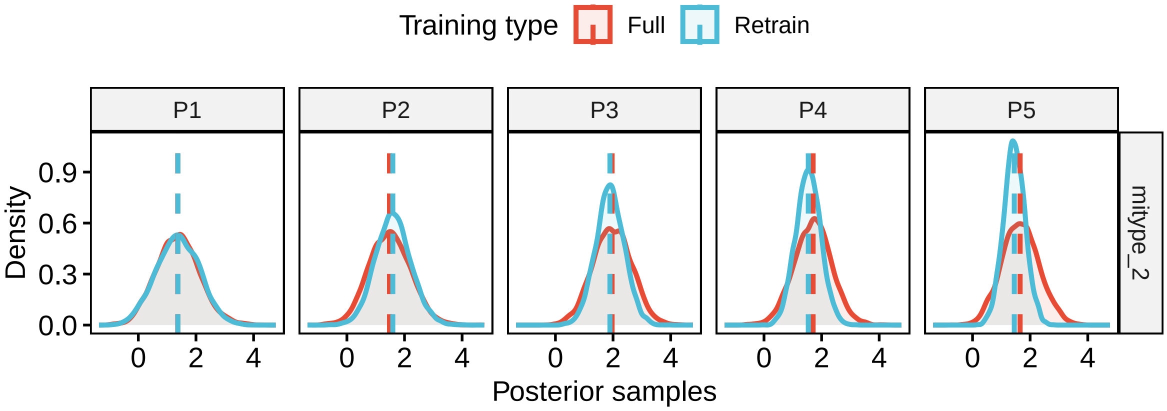
Training type
Full
Retrain
P1
P2
P3
P4
P5
Density
0.0
0.3
0.6
0.9
0
2
4
Posterior samples
mitype_2

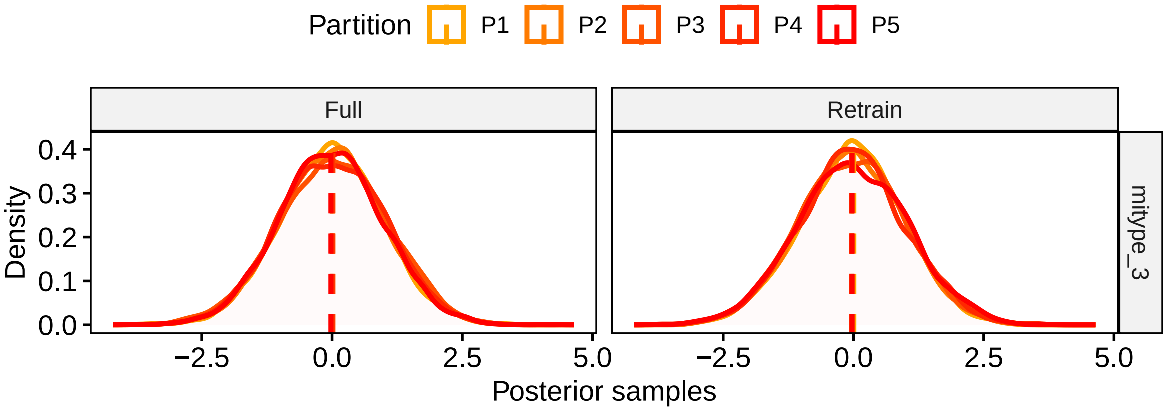
Partition
P1
P2
P3
P4
P5
Full
Retrain
Density
0.0
0.1
0.2
0.3
0.4
−2.5
0.0
2.5
5.0
Posterior samples
mitype_3

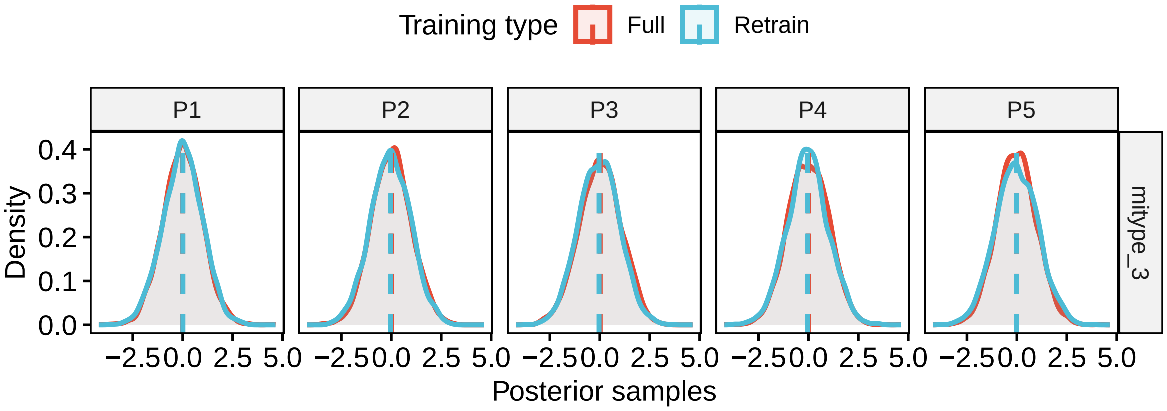
Training type
Full
Retrain
P1
P2
P3
P4
P5
Density
0.0
0.1
0.2
0.3
0.4
−2.5
0.0
2.5
5.0
Posterior samples
mitype_3

GBCS

```
gbcs_vars <- data %>% filter(experiment == 'gbcs' & model == 'pm_exp') %>% select(var) %>%

for(var_idx in unique(gbcs_vars$var)){
  p1 <- plot_density(data, 'gbcs', 'pm_exp', var_idx)
  plot(p1)

  p <- plot_density_2(data, 'gbcs', 'pm_exp', var_idx)
  plot(p)
}
```

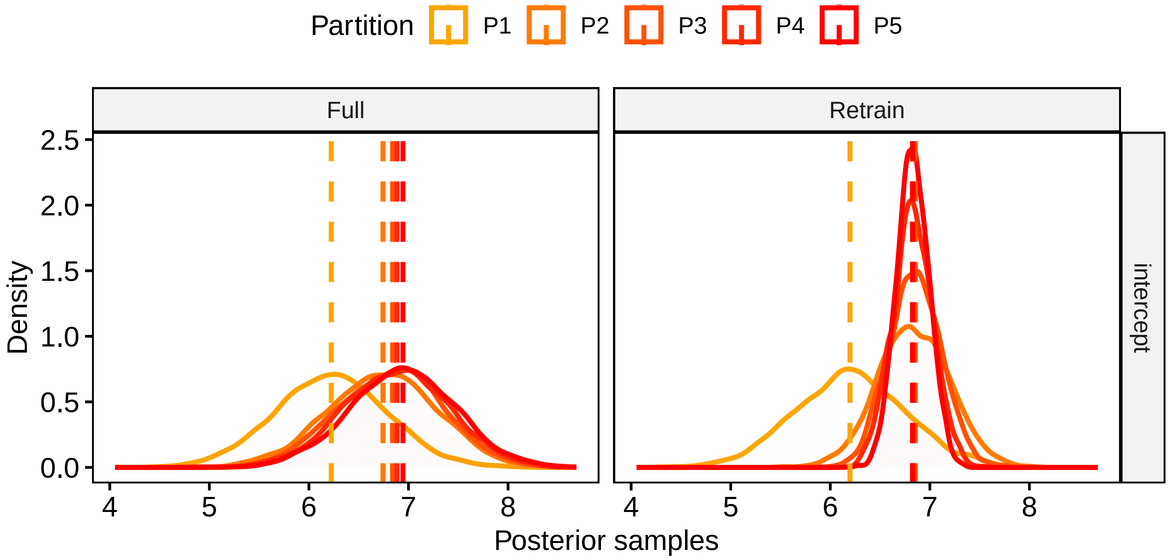

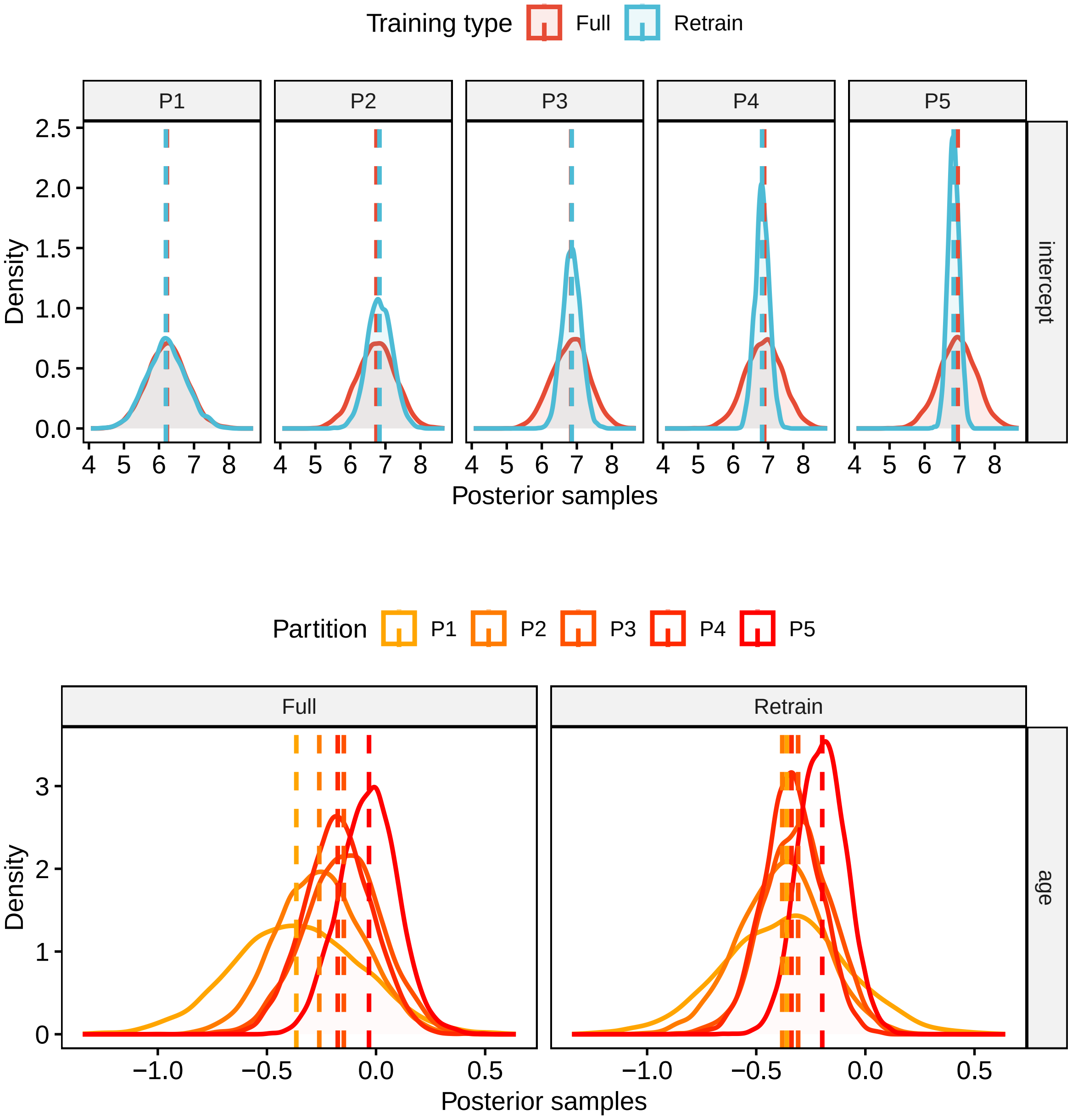
Training type
Full
Retrain
P1
P2
P3
P4
P5
intercept
Density
2.5
2.0
1.5
1.0
0.5
0.0
4 5 6 7 8
Posterior samples
Partition
P1
P2
P3
P4
P5
Full
Retrain
age
Density
3
2
1
0
−1.0
−0.5
0.0
0.5
Posterior samples

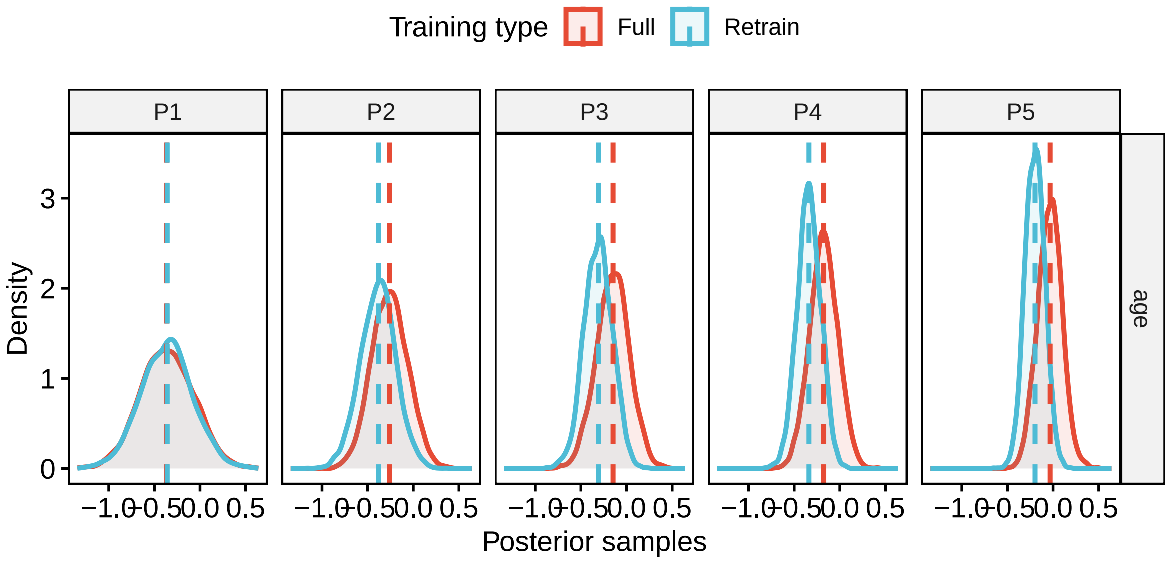
Training type
Full
Retrain
P1
P2
P3
P4
P5
age
Density
Posterior samples

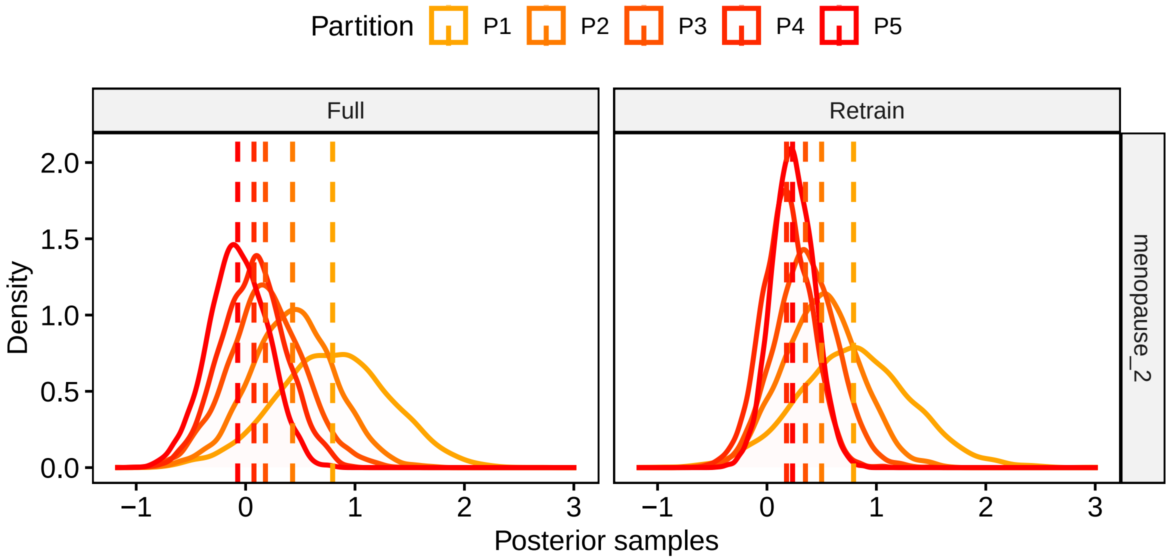
Partition
P1
P2
P3
P4
P5
Full
Retrain
menopause_2
Density
Posterior samples

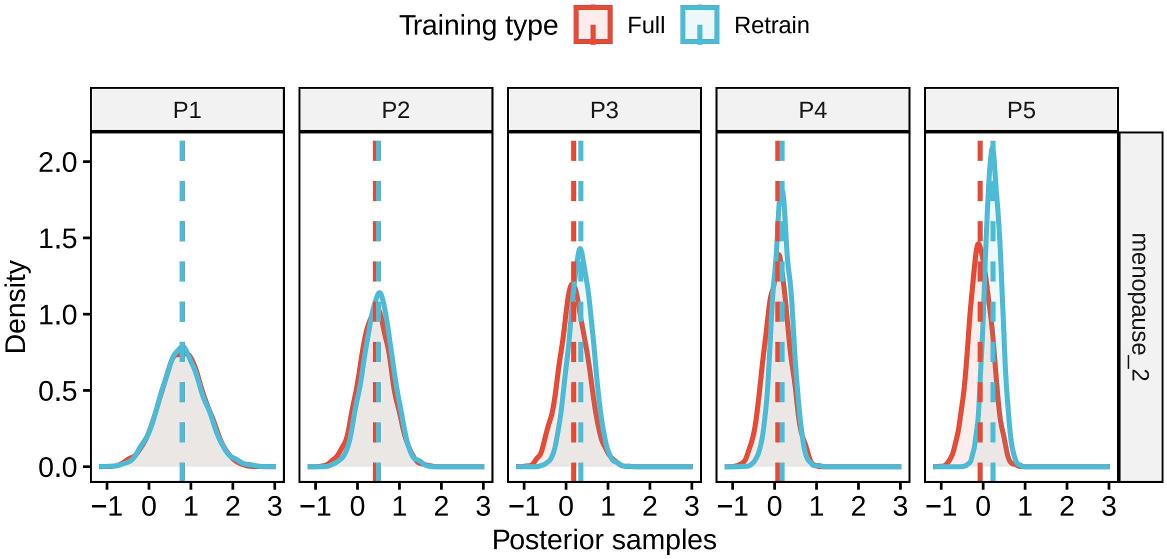

Training type
Full
Retrain
P1
P2
P3
P4
P5
menopause_2
Density
Posterior samples

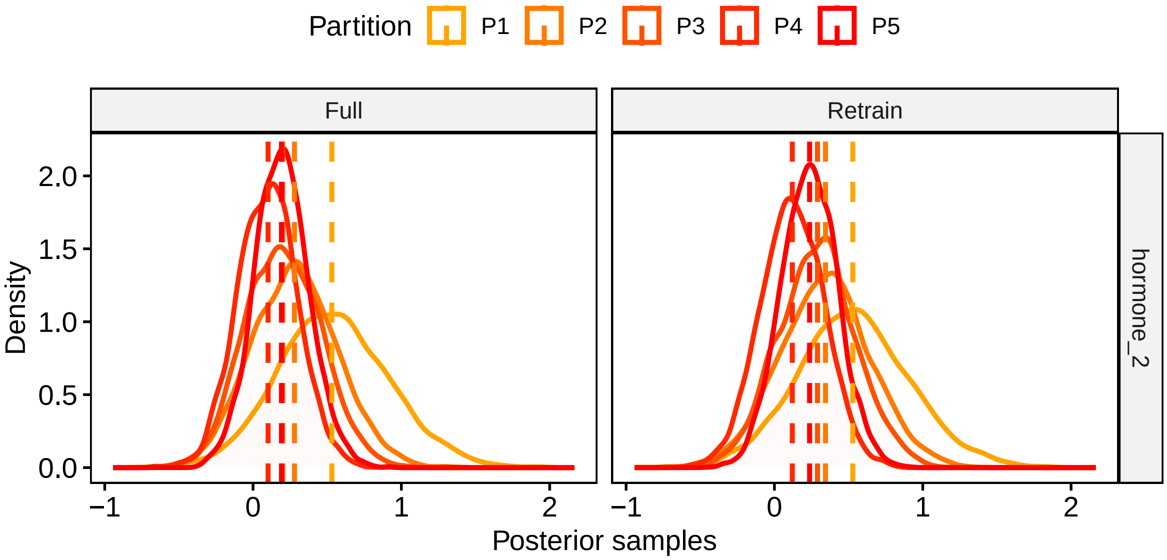

Partition
P1
P2
P3
P4
P5
Full
Retrain
hormone_2
Density
Posterior samples

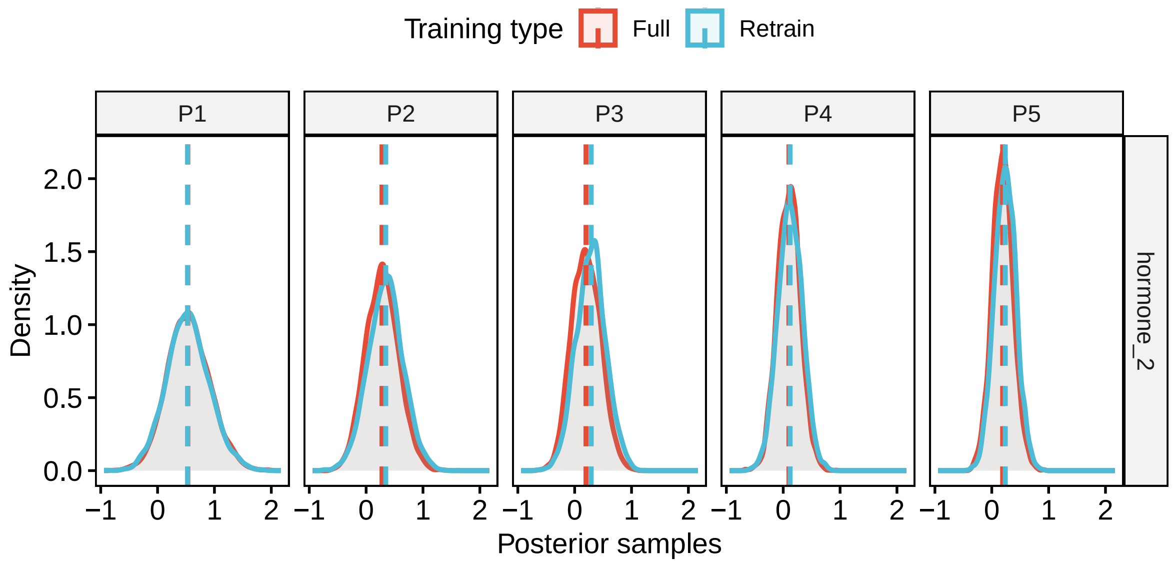

Training type
Full
Retrain
P1
P2
P3
P4
P5
hormone_2
Density
2.0
1.5
1.0
0.5
0.0
−1
0
1
2
Posterior samples

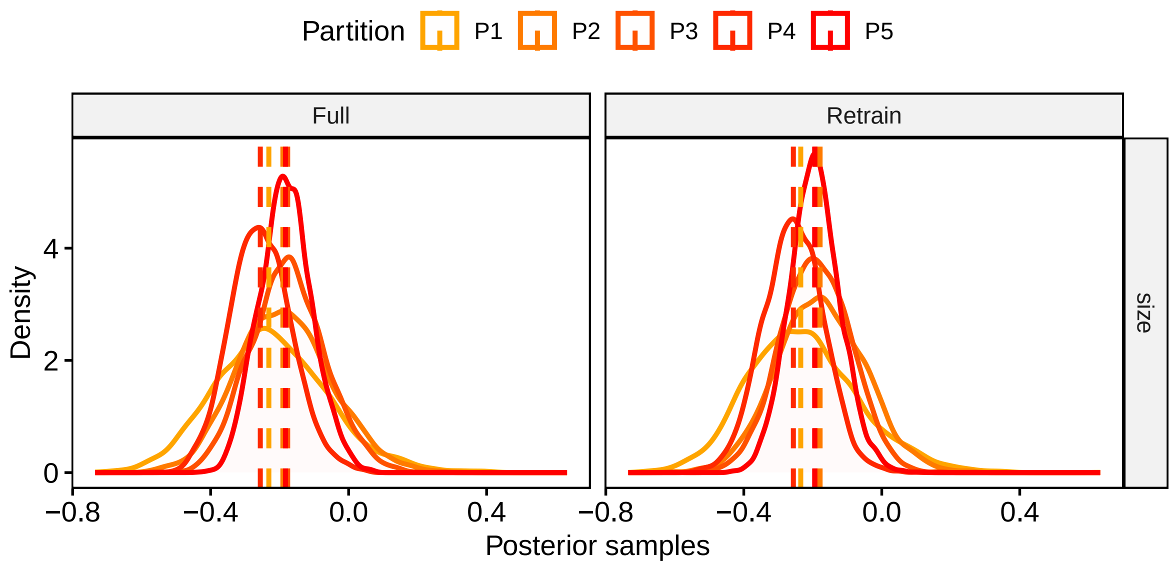

Partition
P1
P2
P3
P4
P5
Full
Retrain
size
Density
4
2
0
−0.8
−0.4
0.0
0.4
Posterior samples

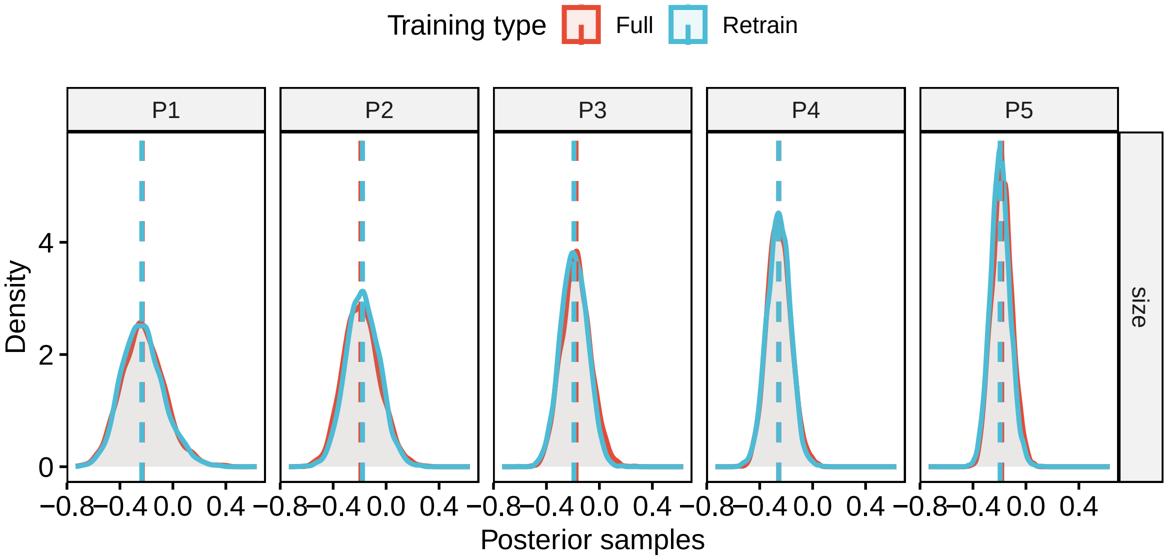
Training type
Full
Retrain
P1
P2
P3
P4
P5
size
Density
Posterior samples

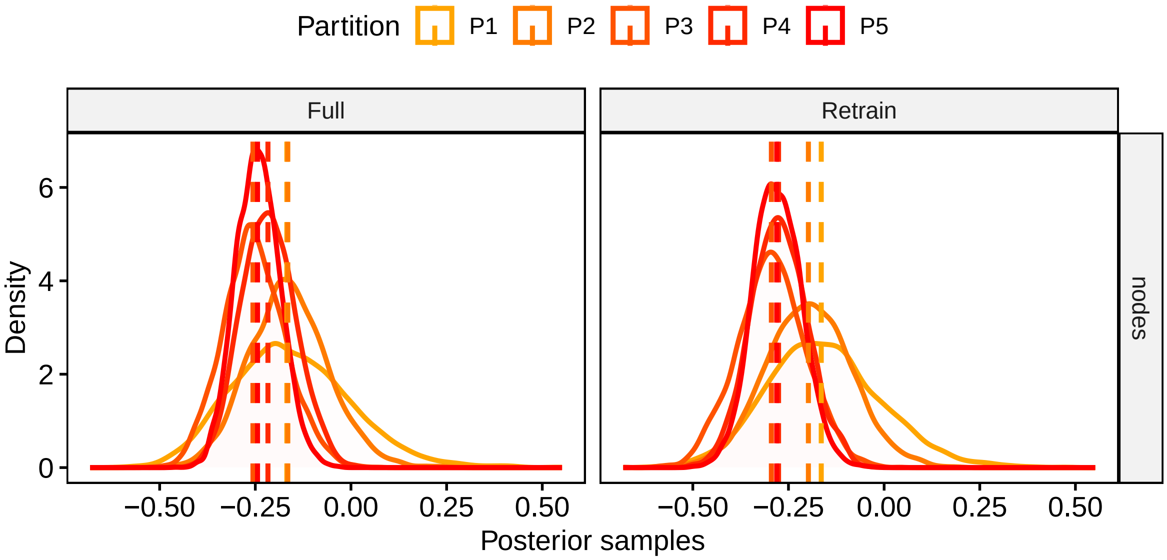
Partition
P1
P2
P3
P4
P5
Full
Retrain
nodes
Density
Posterior samples

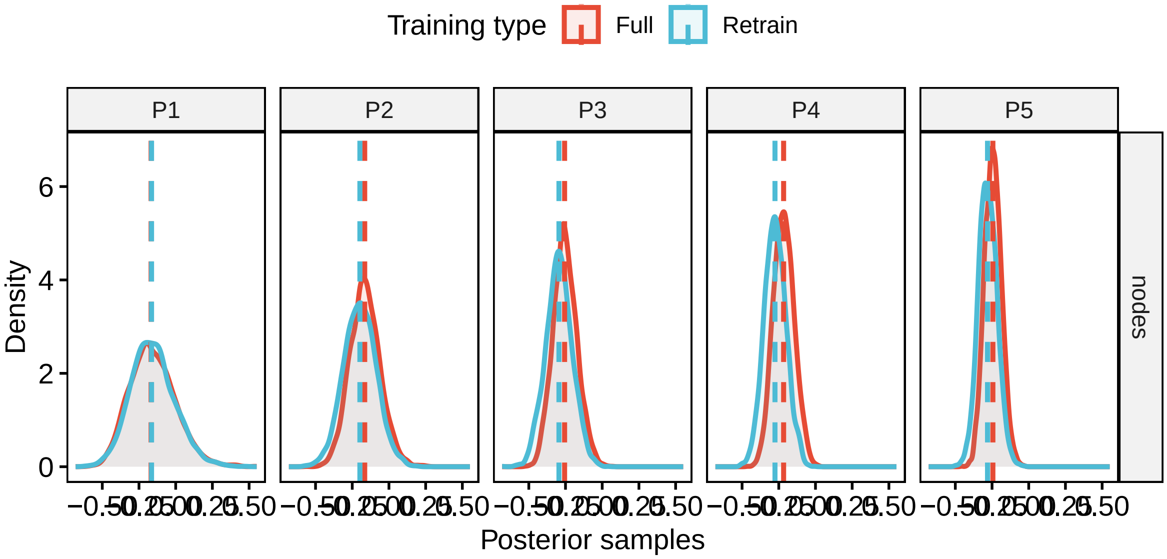
Training type
Full
Retrain
P1
P2
P3
P4
P5
nodes
Density
0
2
4
6
Posterior samples

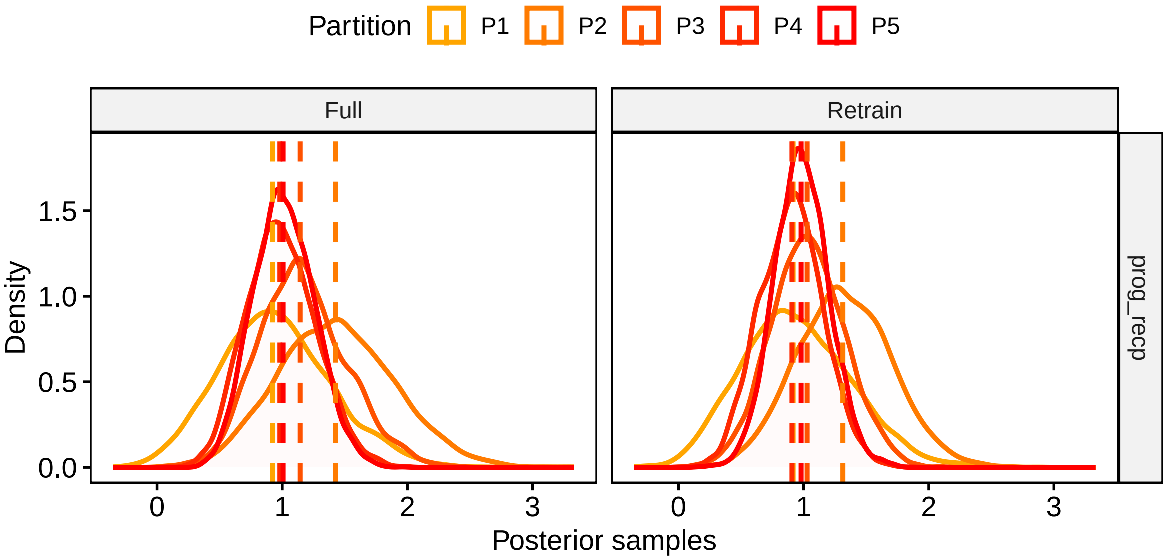
Partition
P1
P2
P3
P4
P5
Full
Retrain
prog_recp
Density
0.0
0.5
1.0
1.5
0
1
2
3
Posterior samples

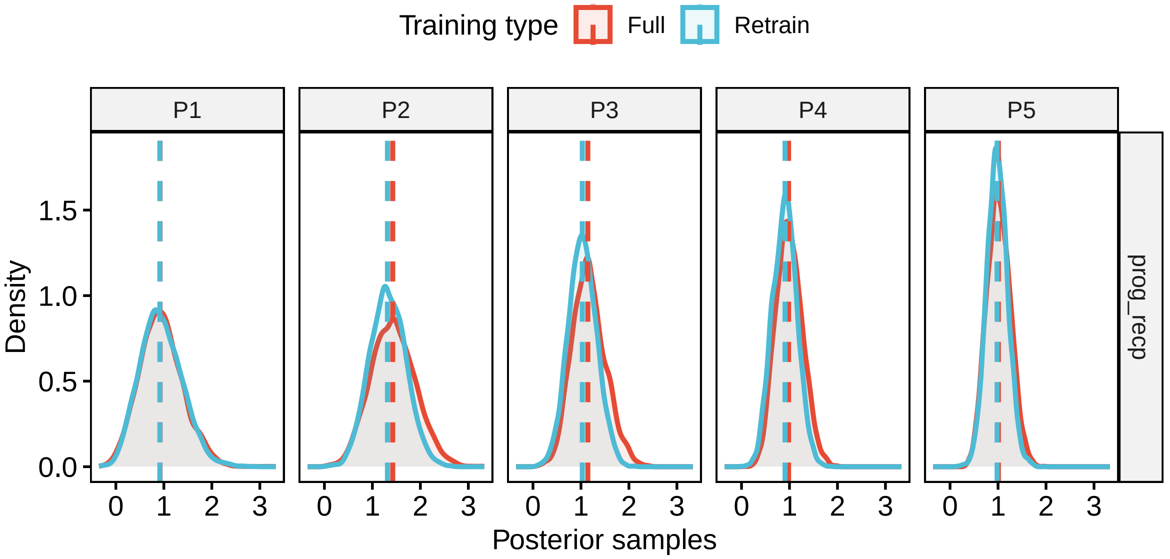
Training type
Full
Retrain
P1
P2
P3
P4
P5
prog_recp
Density
1.5
1.0
0.5
0.0
0
1
2
3
Posterior samples

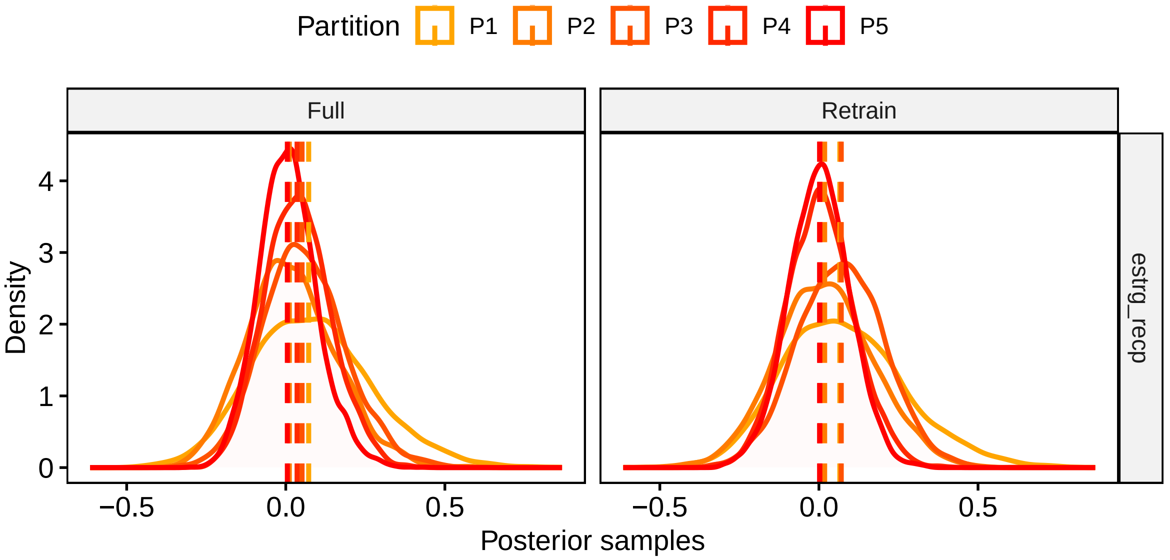
Partition
P1
P2
P3
P4
P5
Full
Retrain
estrg_recp
Density
4
3
2
1
0
−0.5
0.0
0.5
Posterior samples

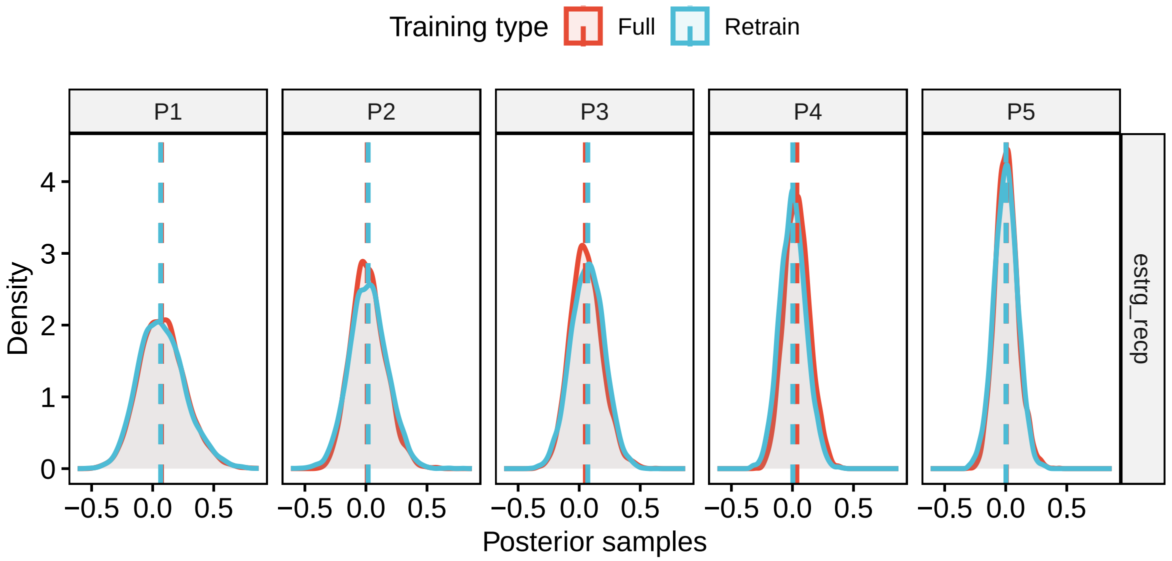
Training type
Full
Retrain
P1
P2
P3
P4
P5
estrg_recp
Density
Posterior samples

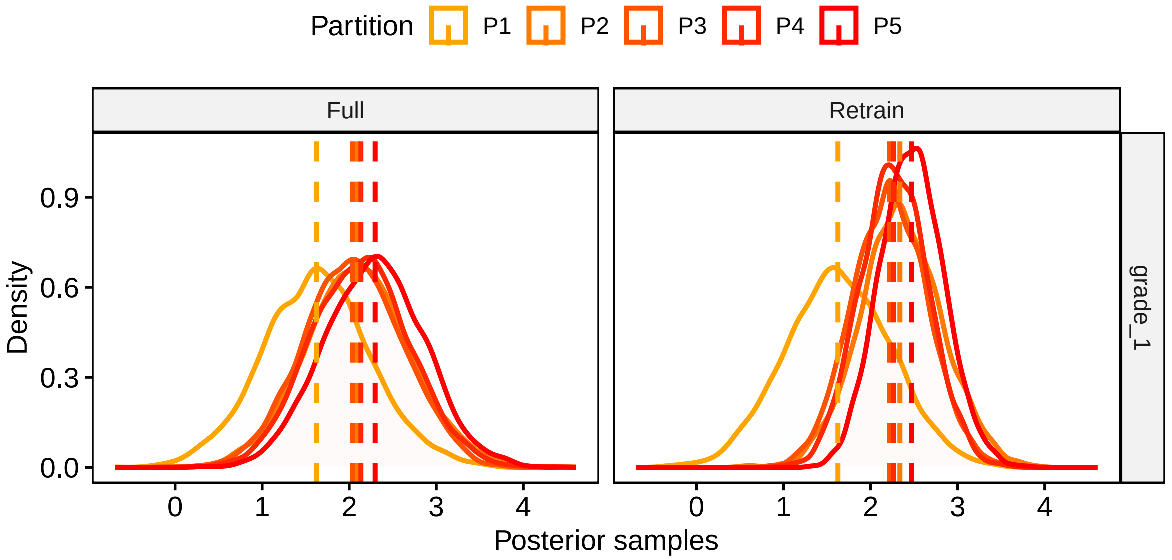
Partition
P1
P2
P3
P4
P5
Full
Retrain
grade_1
Density
Posterior samples

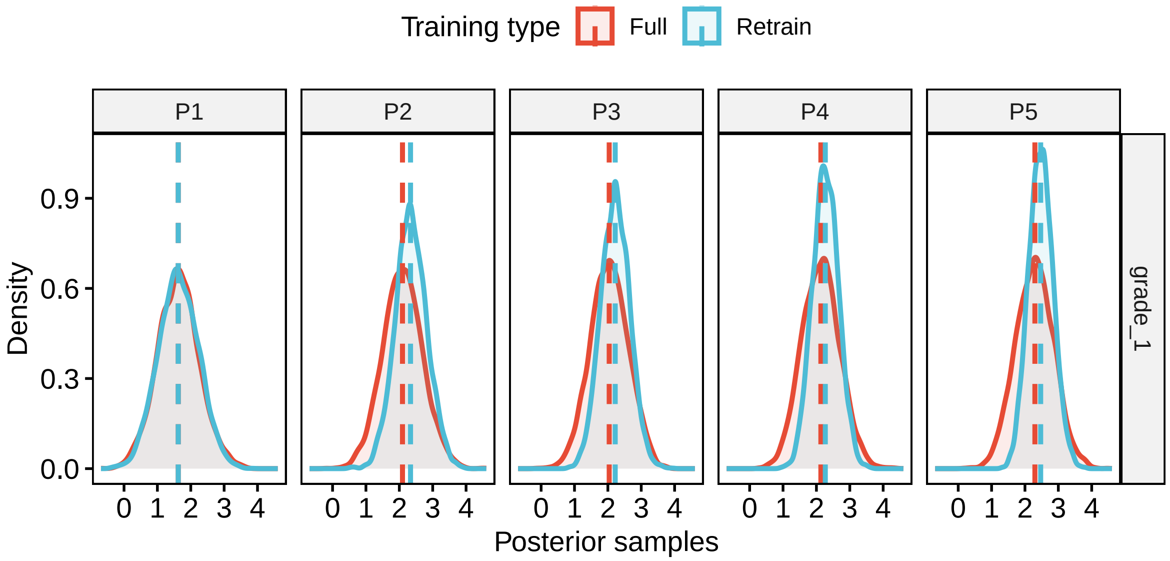
Training type
Full
Retrain
P1
P2
P3
P4
P5
grade_1
Density
0.0
0.3
0.6
0.9
0 1 2 3 4
Posterior samples

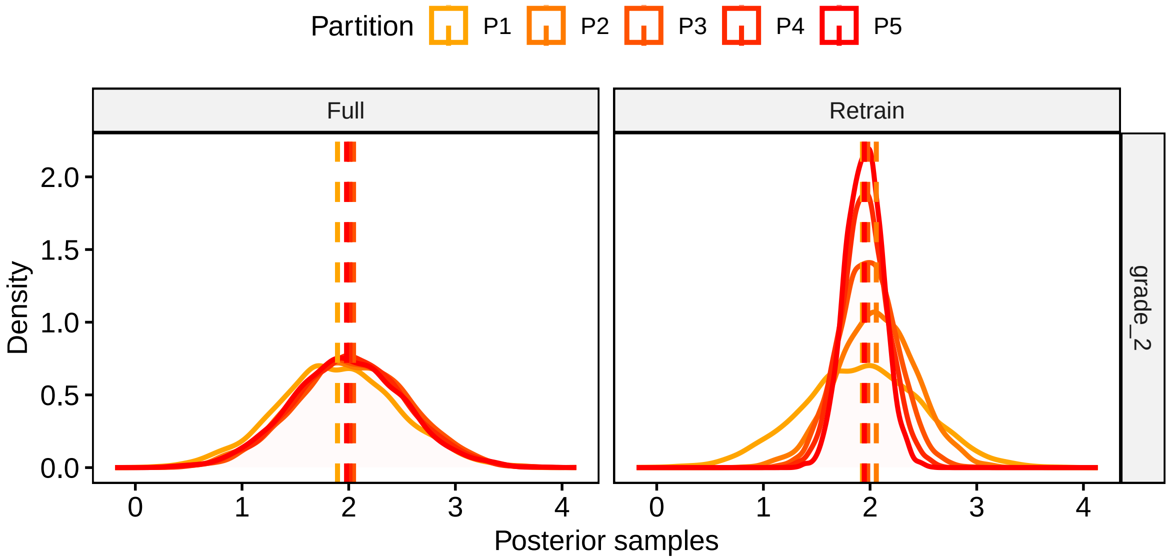
Partition
P1
P2
P3
P4
P5
Full
Retrain
grade_2
Density
0.0
0.5
1.0
1.5
2.0
0
1
2
3
4
Posterior samples

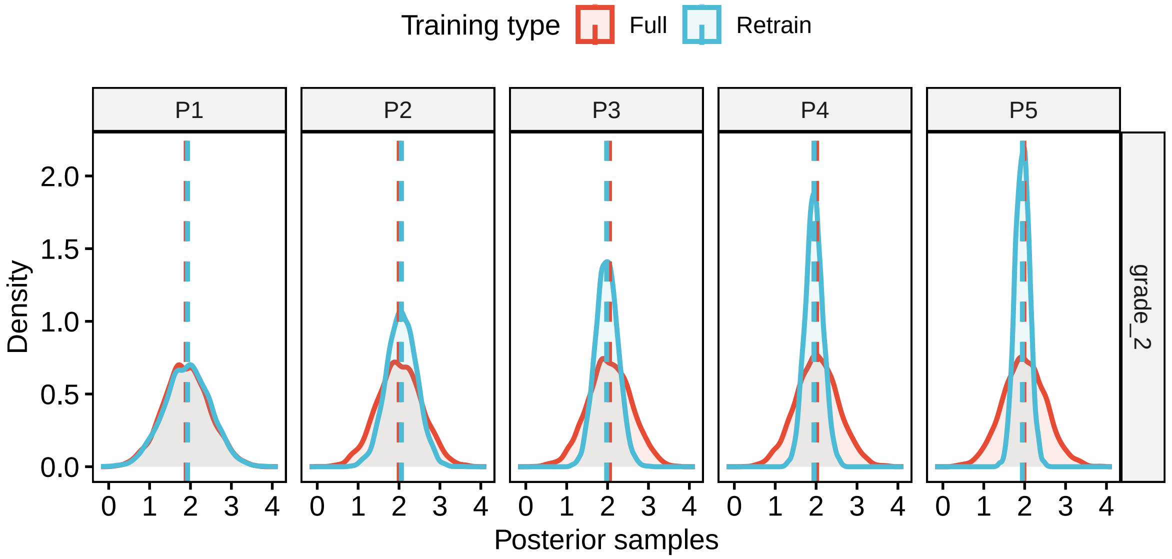
Training type
Full
Retrain
P1
P2
P3
P4
P5
Density
grade_2
Posterior samples

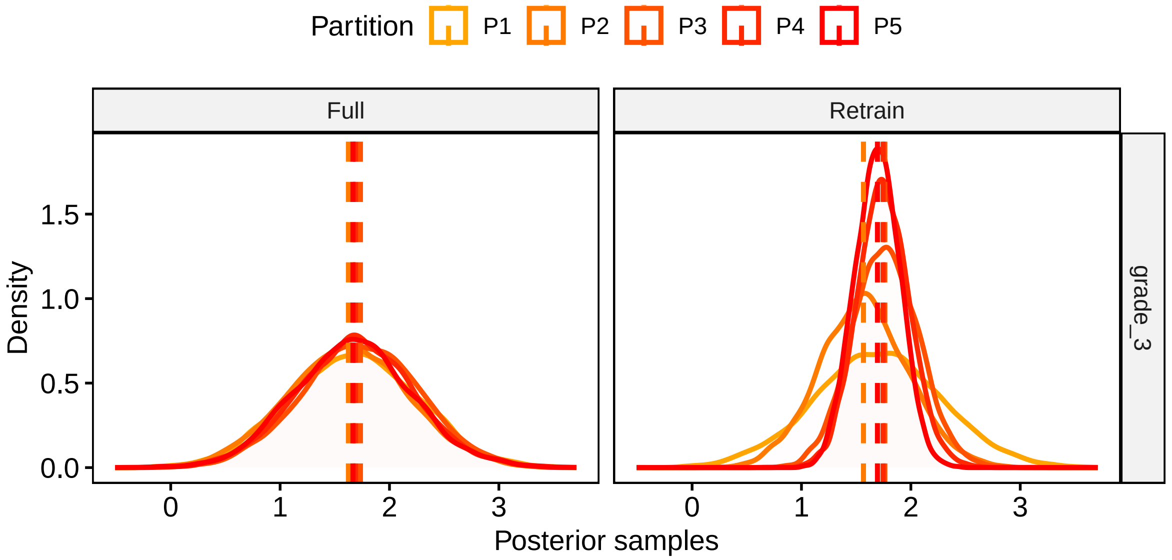
Partition
P1
P2
P3
P4
P5
Full
Retrain
Density
grade_3
Posterior samples

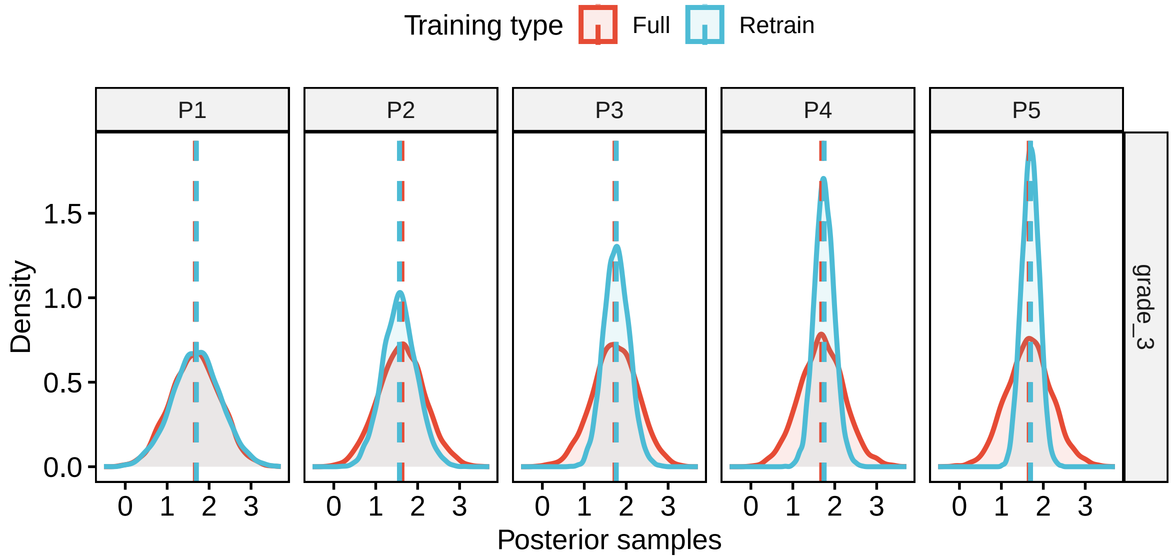

PBC

```
pbc_vars <- data %>% filter(experiment == 'pbc' & model == 'pm_exp') %>% select(var) %>% u


for(var_idx in unique(pbc_vars$var)){
  p1 <- plot_density(data, 'pbc', 'pm_exp', var_idx)
  plot(p1)

  p <- plot_density_2(data, 'pbc', 'pm_exp', var_idx)
  plot(p)
}
```

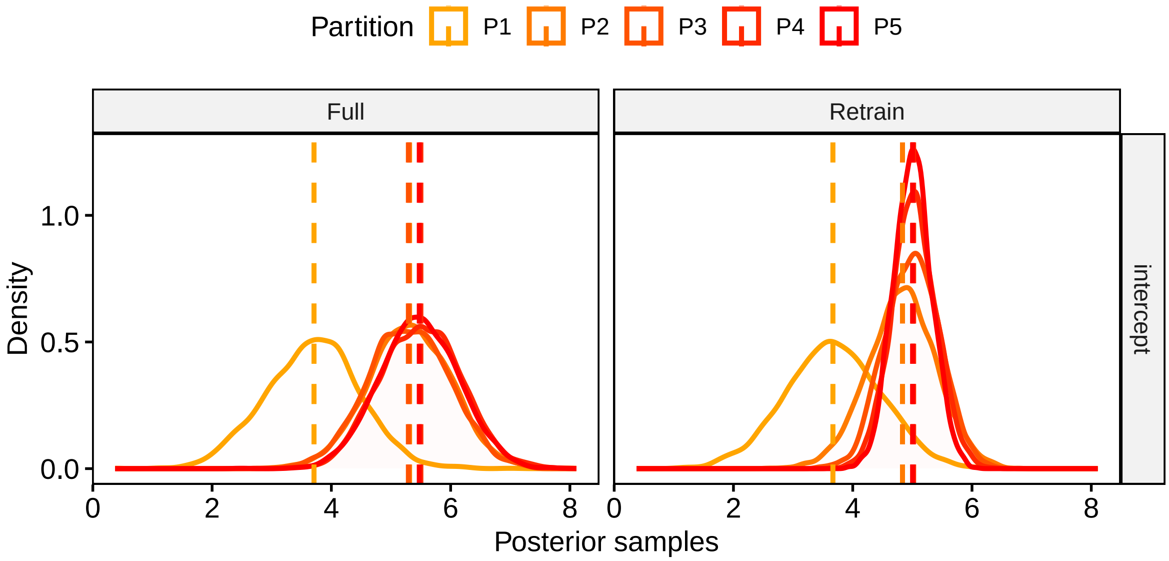

Partition
P1
P2
P3
P4
P5
Full
Retrain
intercept
Density
1.0
0.5
0.0
0
2
4
6
8
Posterior samples

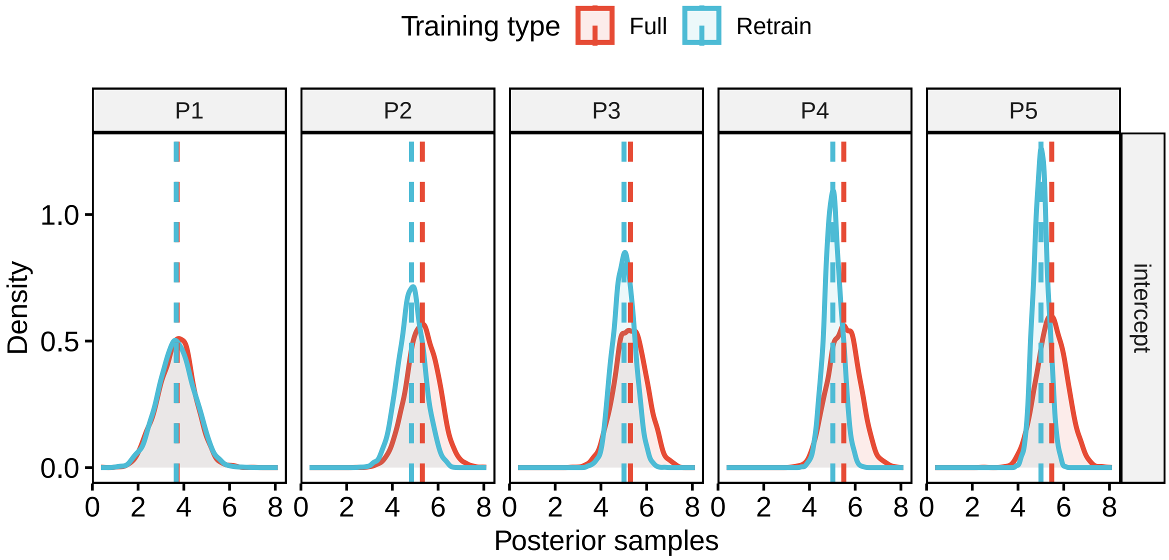

Training type
Full
Retrain
P1
P2
P3
P4
P5
intercept
Density
1.0
0.5
0.0
0
2
4
6
8
Posterior samples

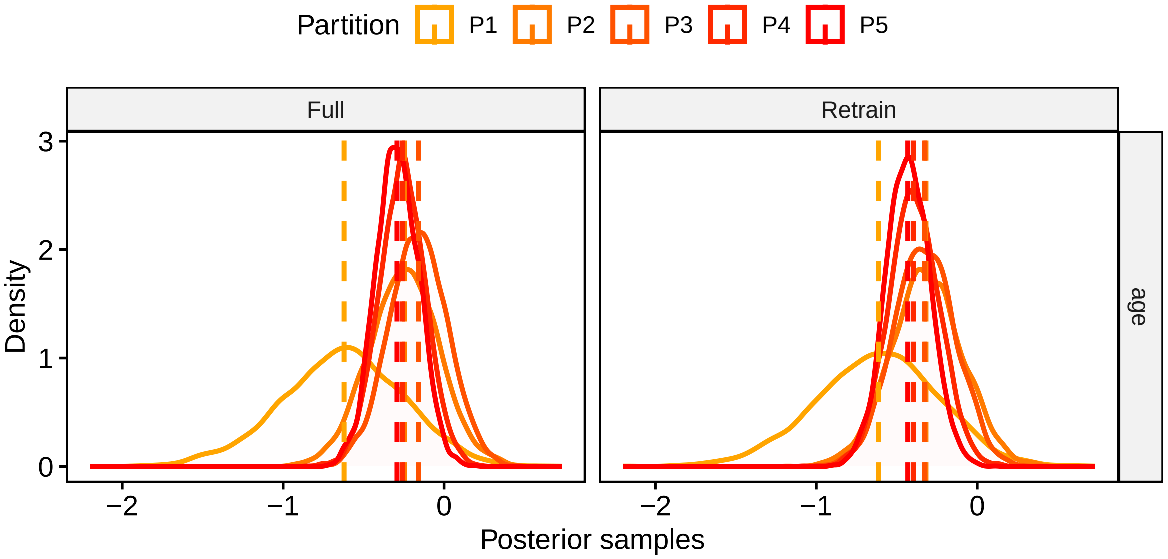
Partition
P1
P2
P3
P4
P5
Full
Retrain
age
Density
Posterior samples
3
2
1
0
−2
−1
0

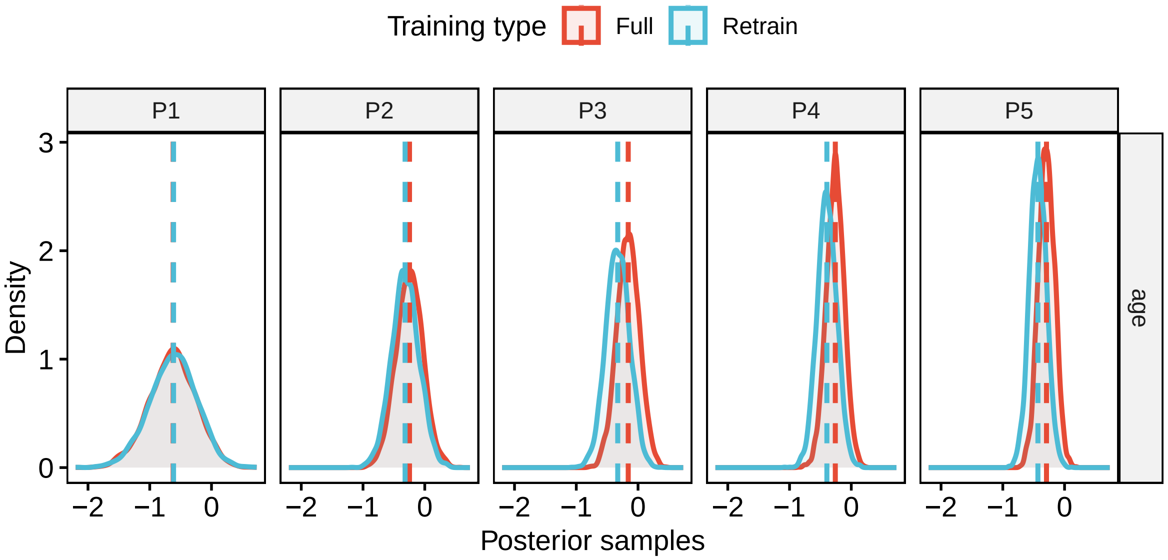
Training type
Full
Retrain
P1
P2
P3
P4
P5
age
Density
Posterior samples
3
2
1
0
−2
−1
0

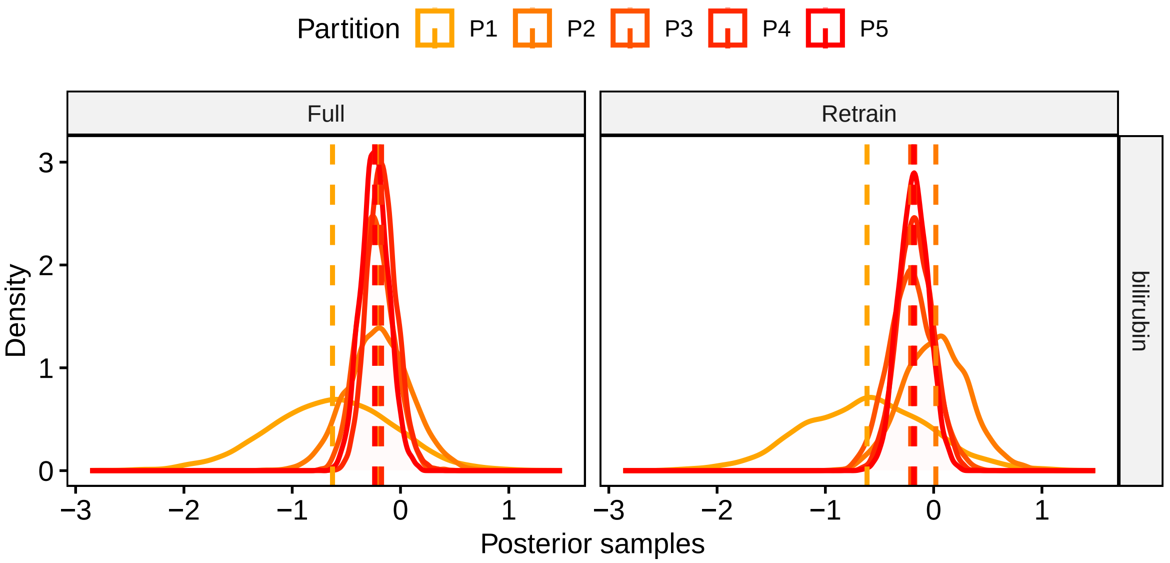
Partition P1 P2 P3 P4 P5
Full
Retrain
bilirubin
Density
Posterior samples
3 2 1 0
−3 −2 −1 0 1

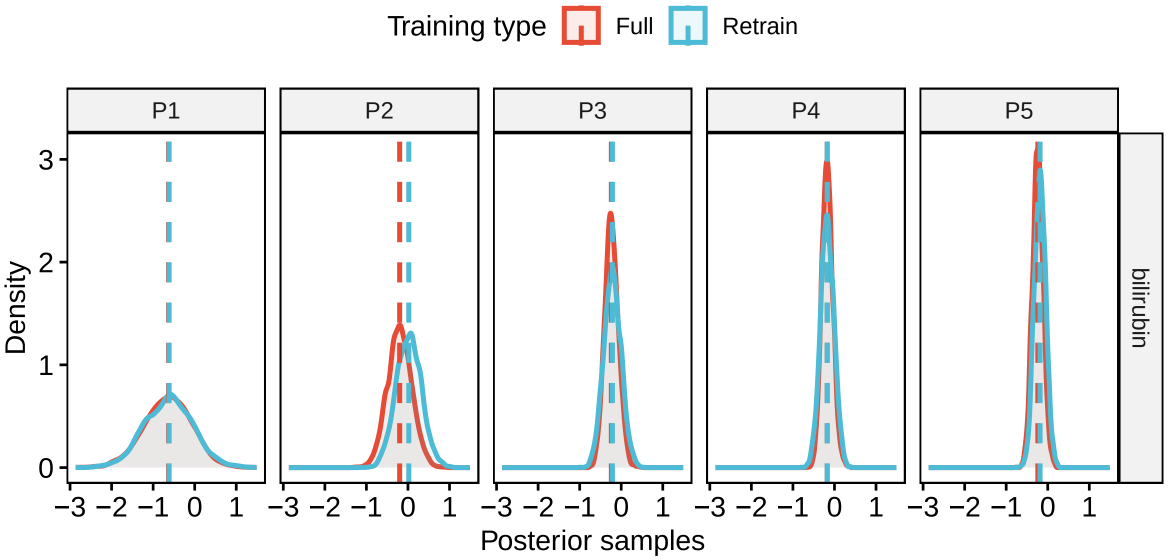
Training type Full Retrain
P1
P2
P3
P4
P5
bilirubin
Density
Posterior samples
3 2 1 0
−3 −2 −1 0 1

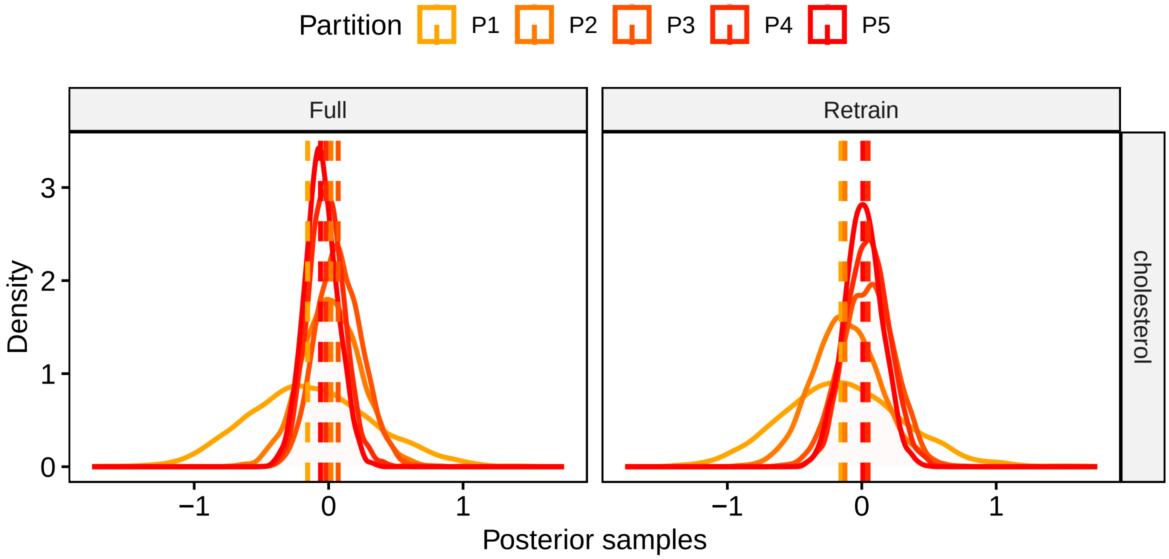

Partition
P1
P2
P3
P4
P5
Full
Retrain
cholesterol
Density
Posterior samples

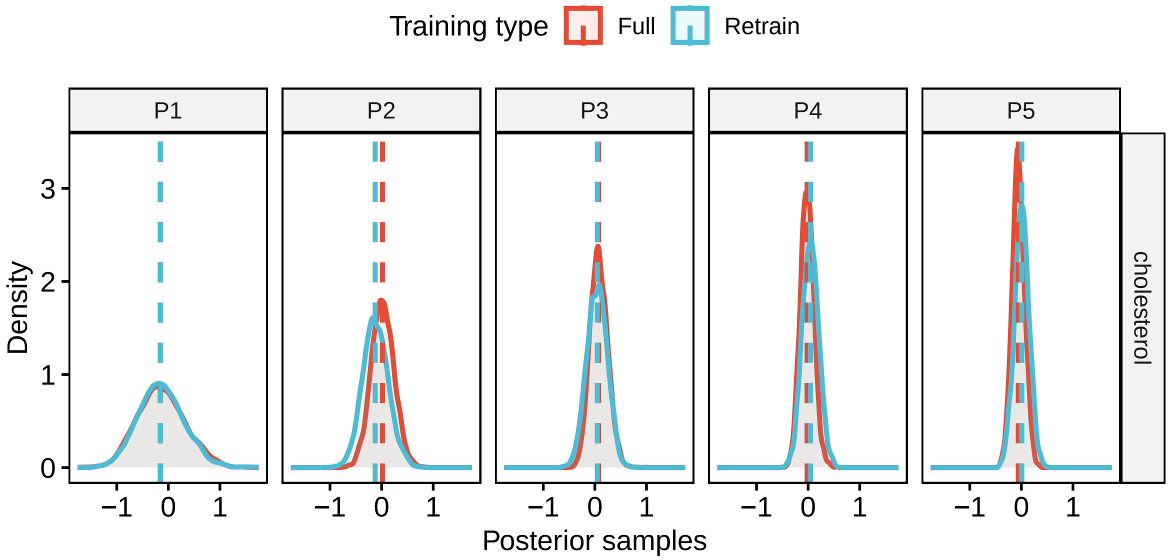

Training type
Full
Retrain
P1
P2
P3
P4
P5
cholesterol
Density
Posterior samples

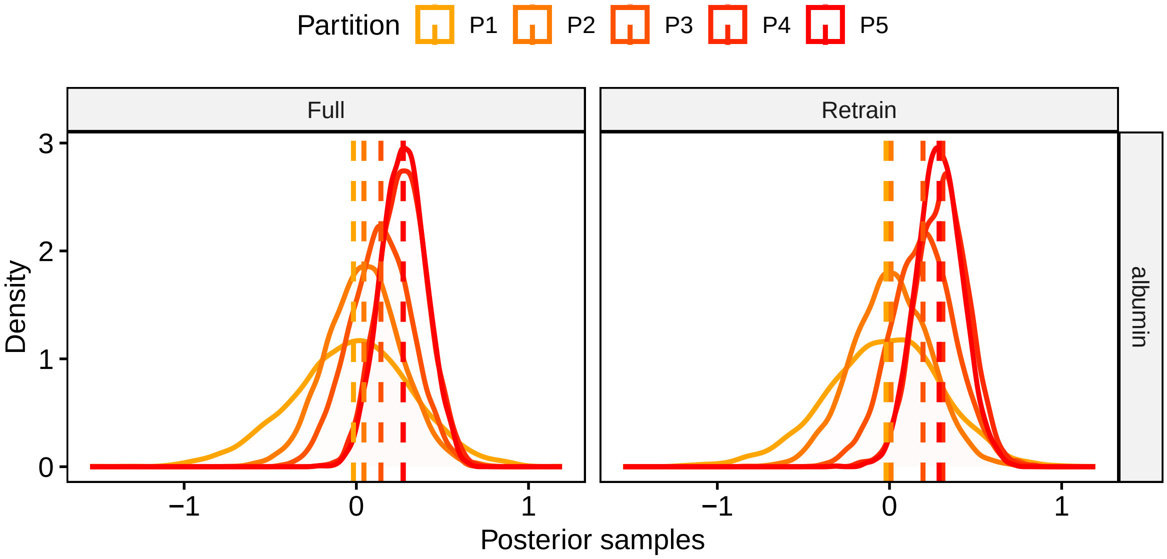
Partition P1 P2 P3 P4 P5
Full
Retrain
albumin
Density
Posterior samples

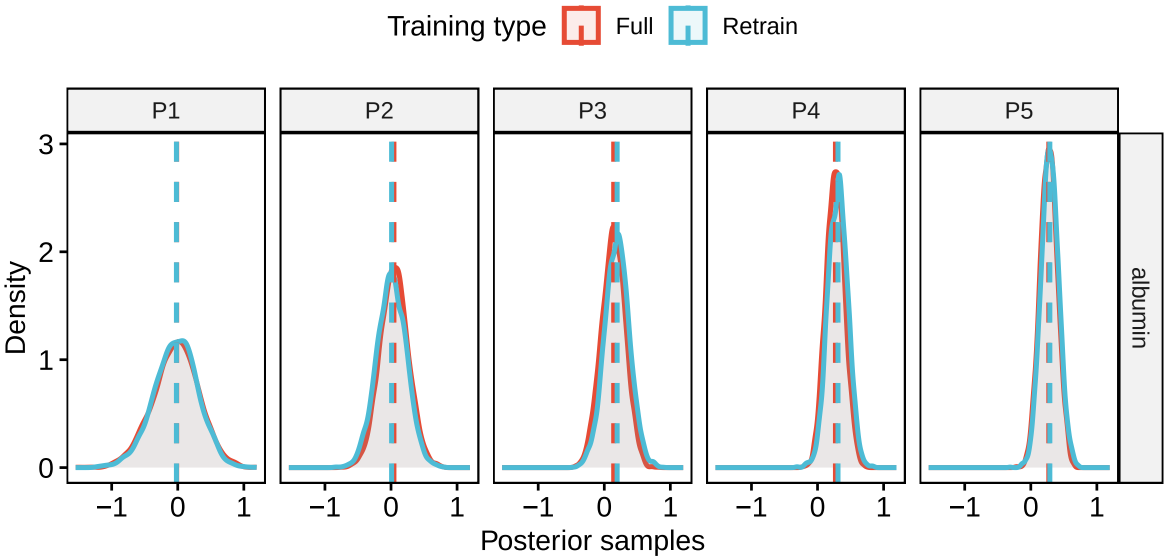
Training type Full Retrain
P1
P2
P3
P4
P5
albumin
Density
Posterior samples

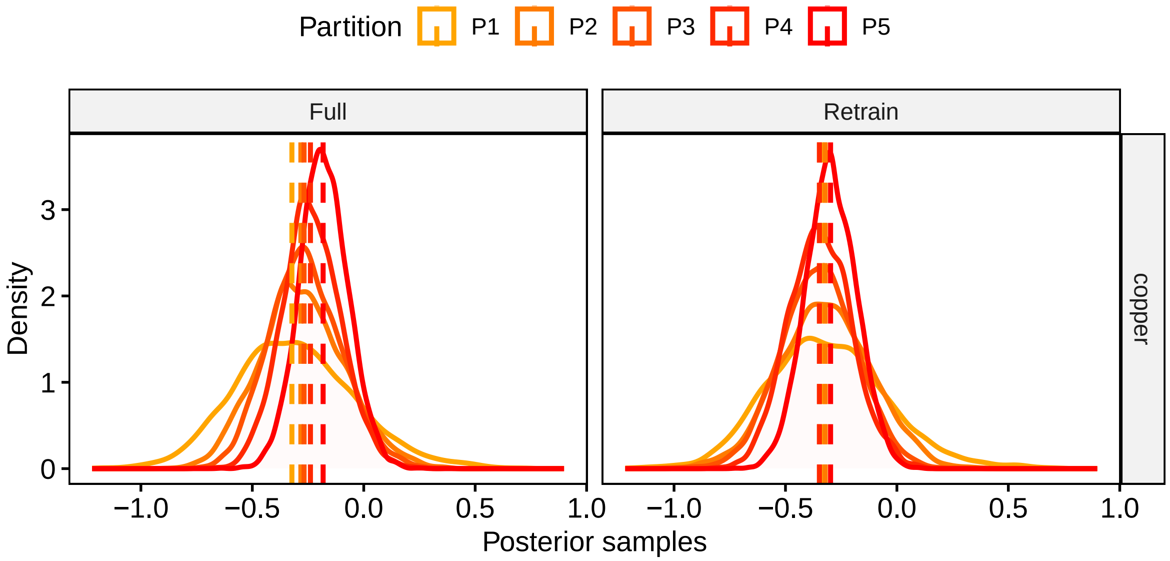
Partition
P1
P2
P3
P4
P5
Full
Retrain
copper
Density
Posterior samples

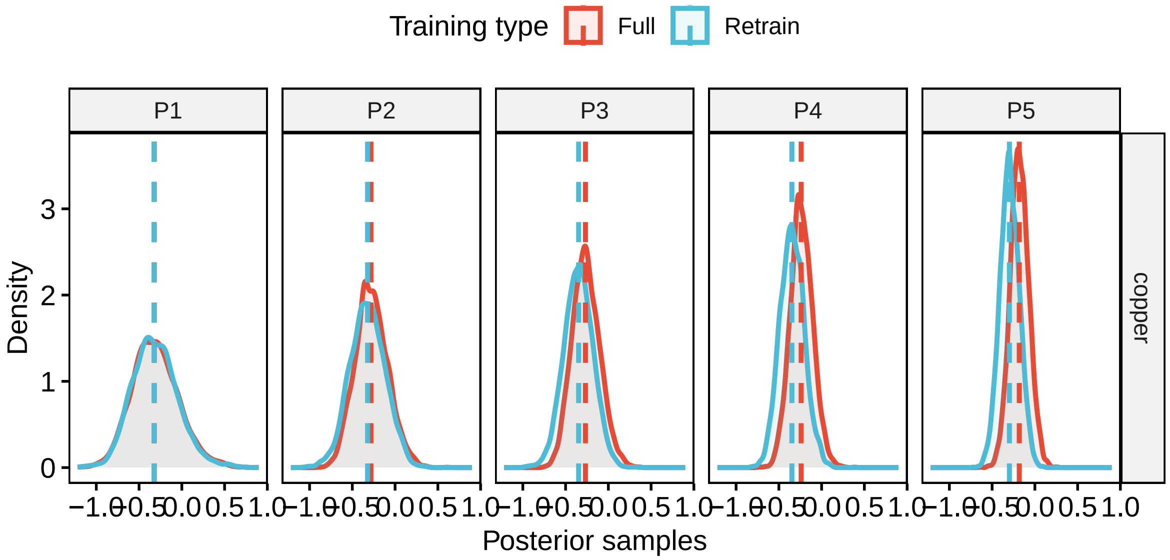
Training type
Full
Retrain
P1
P2
P3
P4
P5
copper
Density
Posterior samples

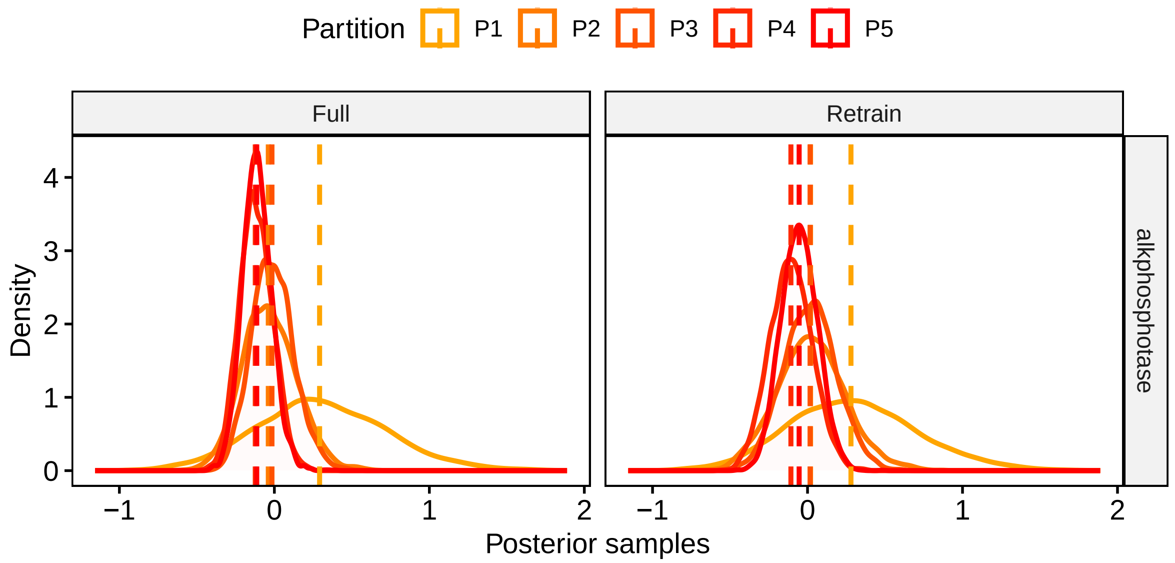
Partition
P1
P2
P3
P4
P5
Full
Retrain
alkphosphotase
Density
Posterior samples

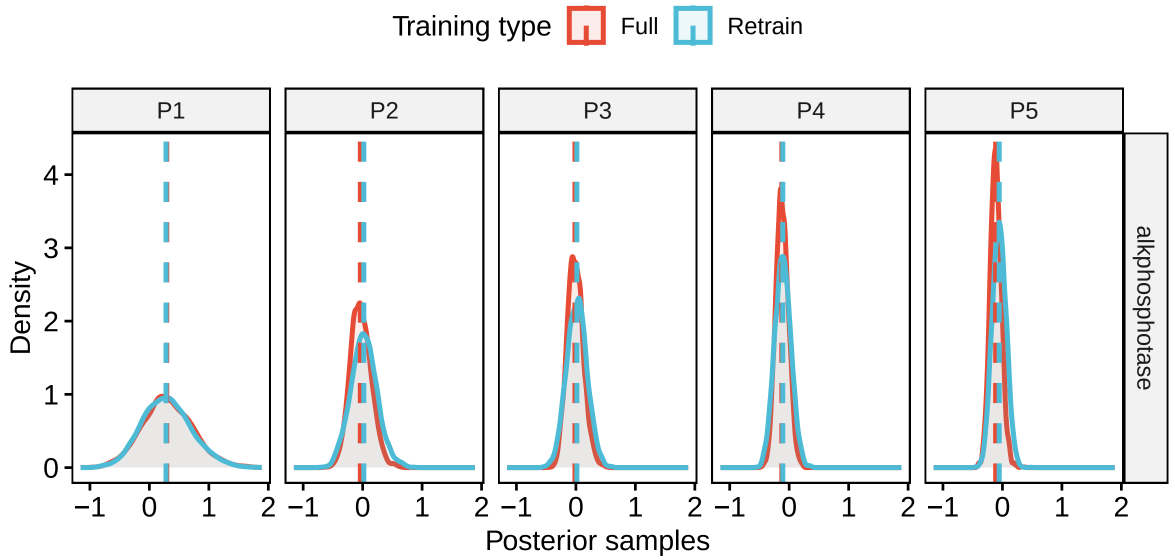
Training type
Full
Retrain
P1
P2
P3
P4
P5
alkphosphotase
Density
Posterior samples

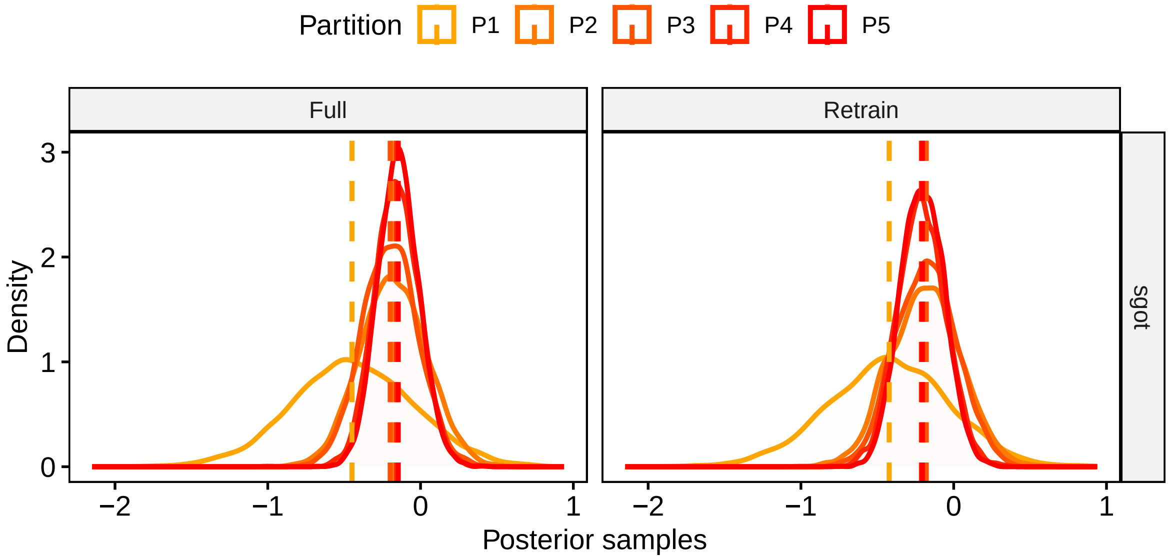

Partition
P1
P2
P3
P4
P5
Full
Retrain
sgot
Density
Posterior samples

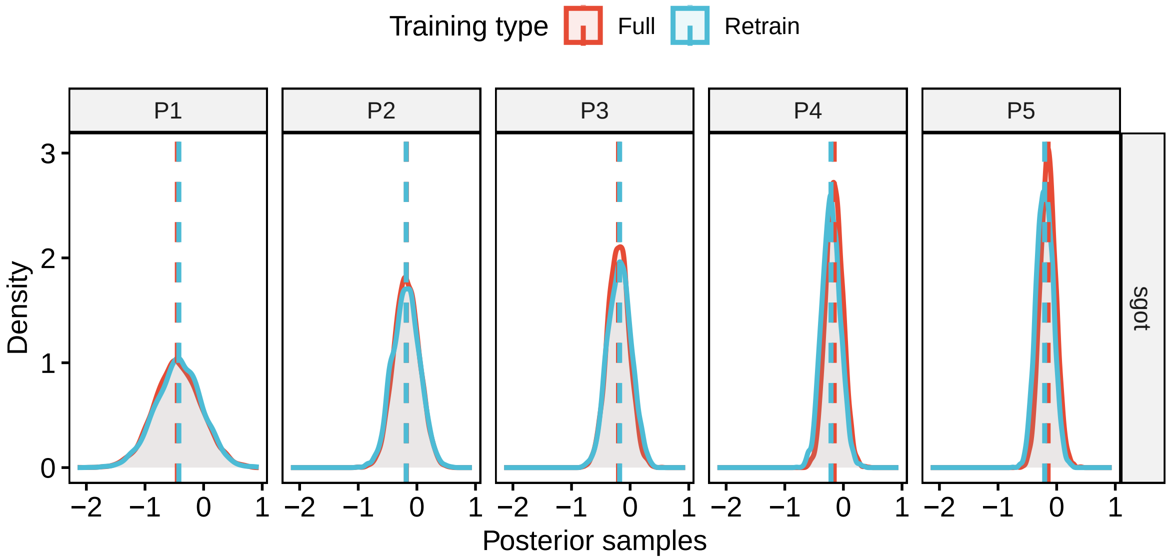

Training type
Full
Retrain
P1
P2
P3
P4
P5
sgot
Density
Posterior samples

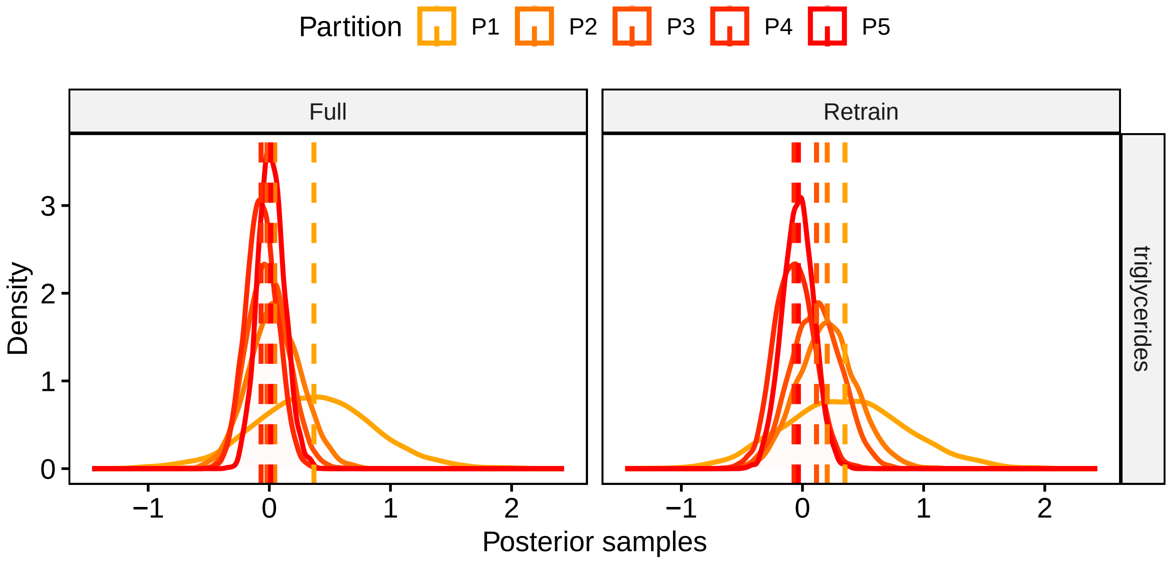

Partition
P1
P2
P3
P4
P5
Full
Retrain
triglycerides
Density
Posterior samples

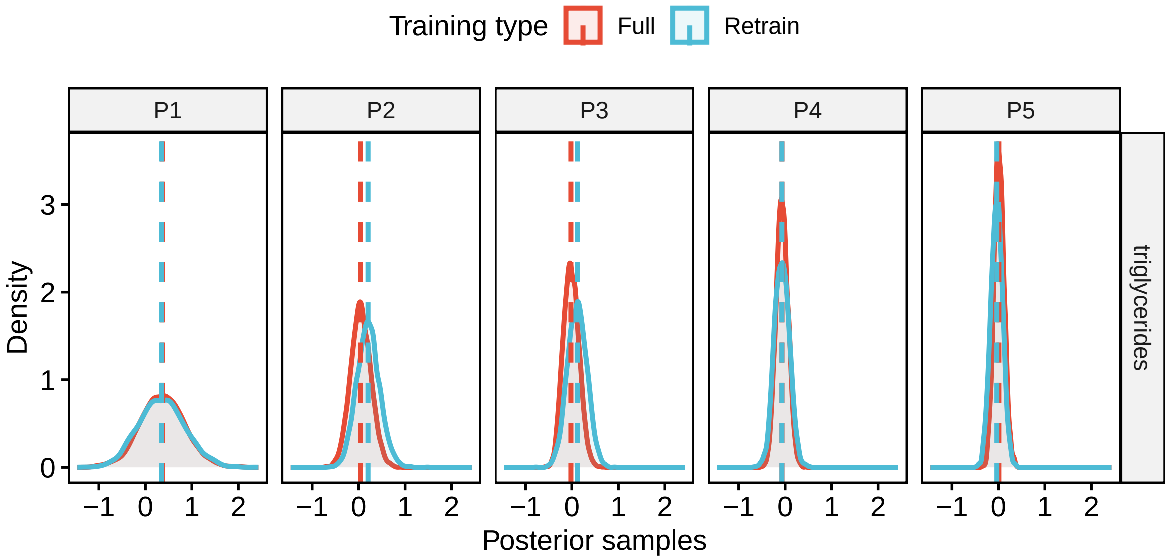

Training type
Full
Retrain
P1
P2
P3
P4
P5
triglycerides
Density
Posterior samples

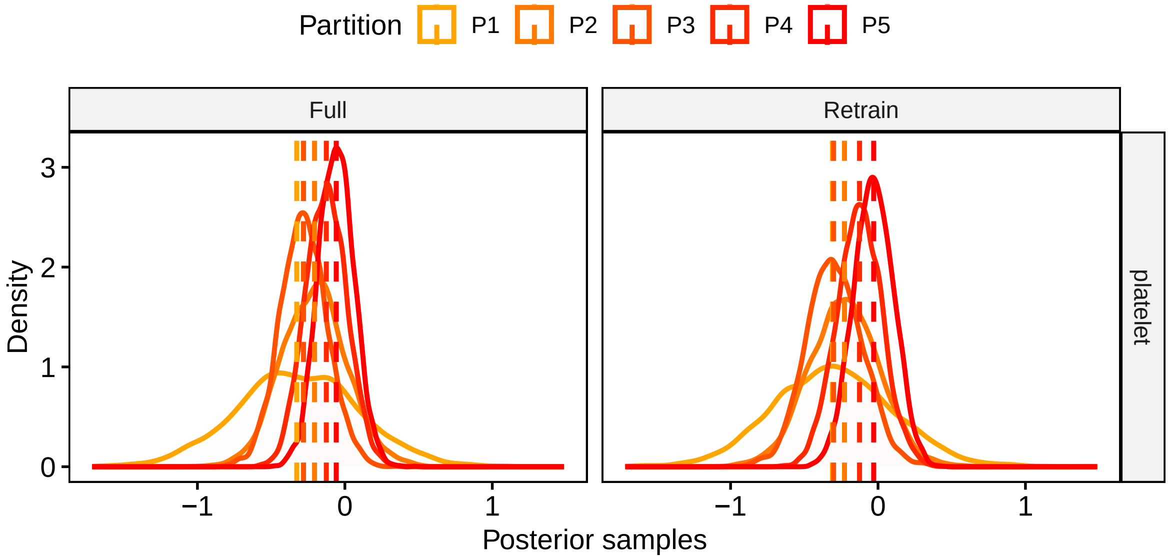
Partition
P1
P2
P3
P4
P5
Full
Retrain
platelet
Density
Posterior samples

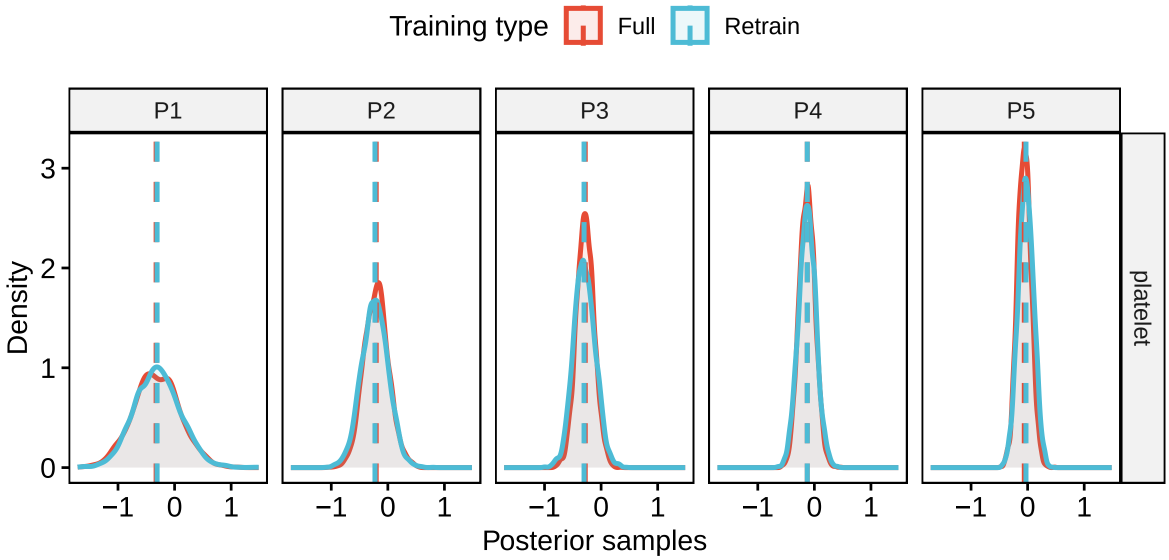
Training type
Full
Retrain
P1
P2
P3
P4
P5
platelet
Density
Posterior samples

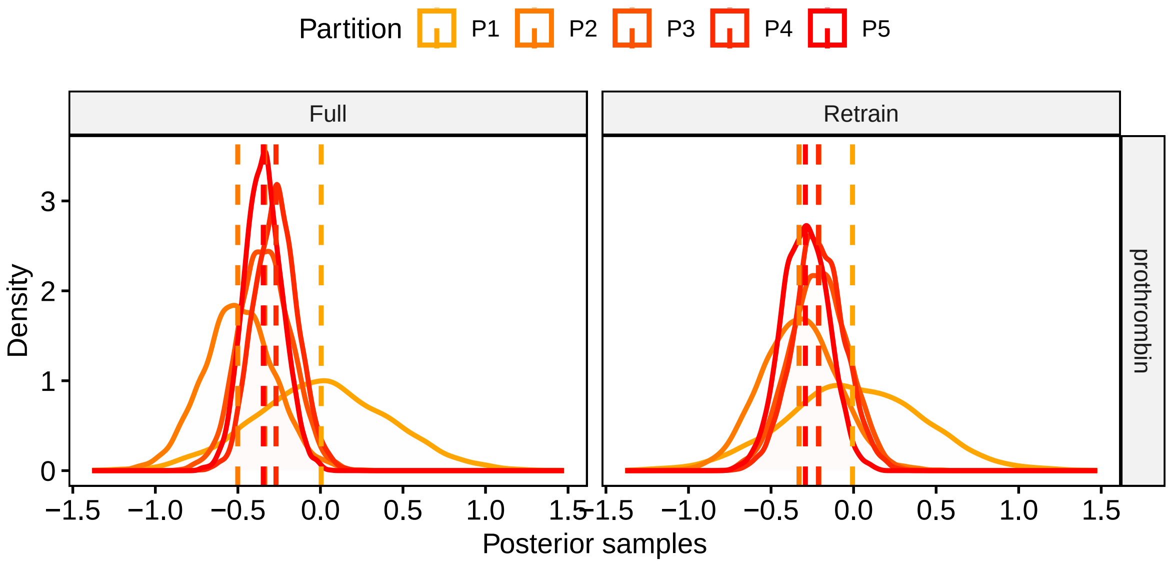

Partition P1 P2 P3 P4 P5
Full
Retrain
prothrombin
Density
Posterior samples

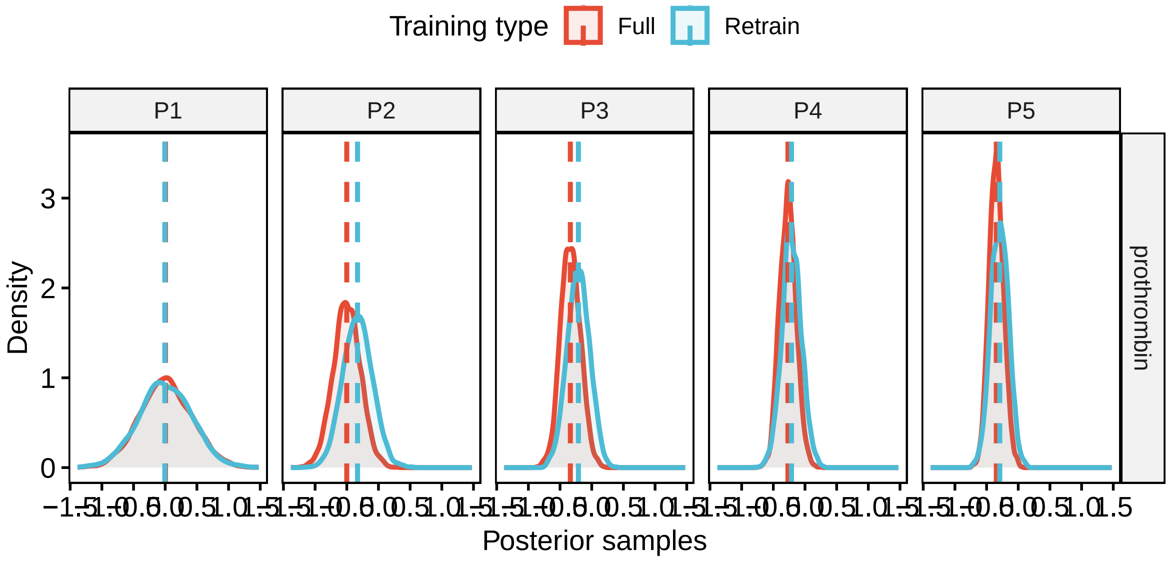

Training type Full Retrain
P1
P2
P3
P4
P5
prothrombin
Density
Posterior samples

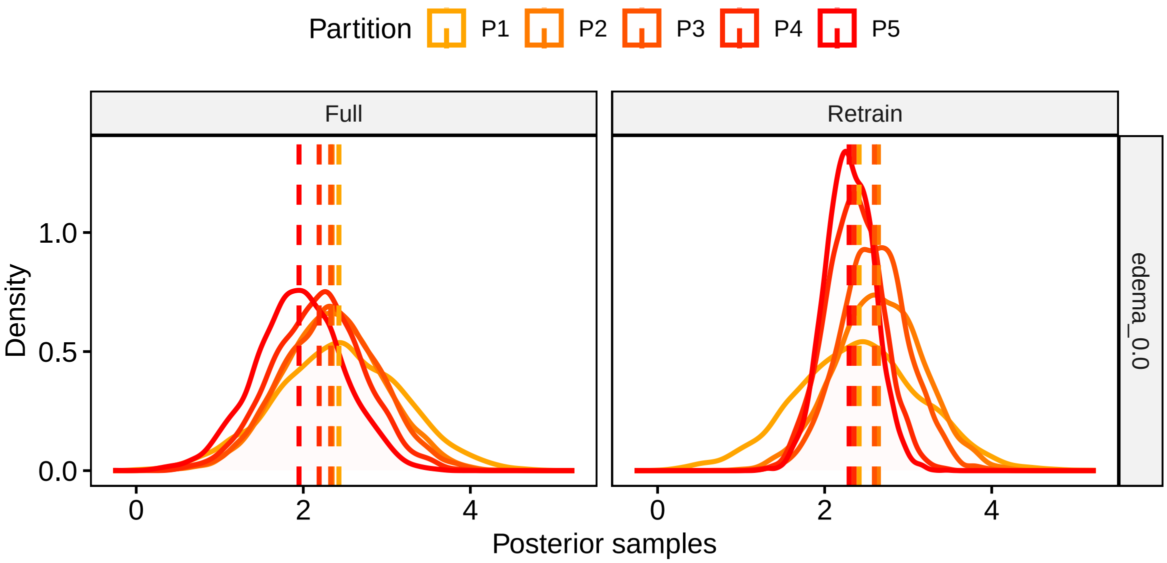

Partition P1 P2 P3 P4 P5
Full
Retrain
edema_0.0
Density
1.0
0.5
0.0
0
2
4
Posterior samples

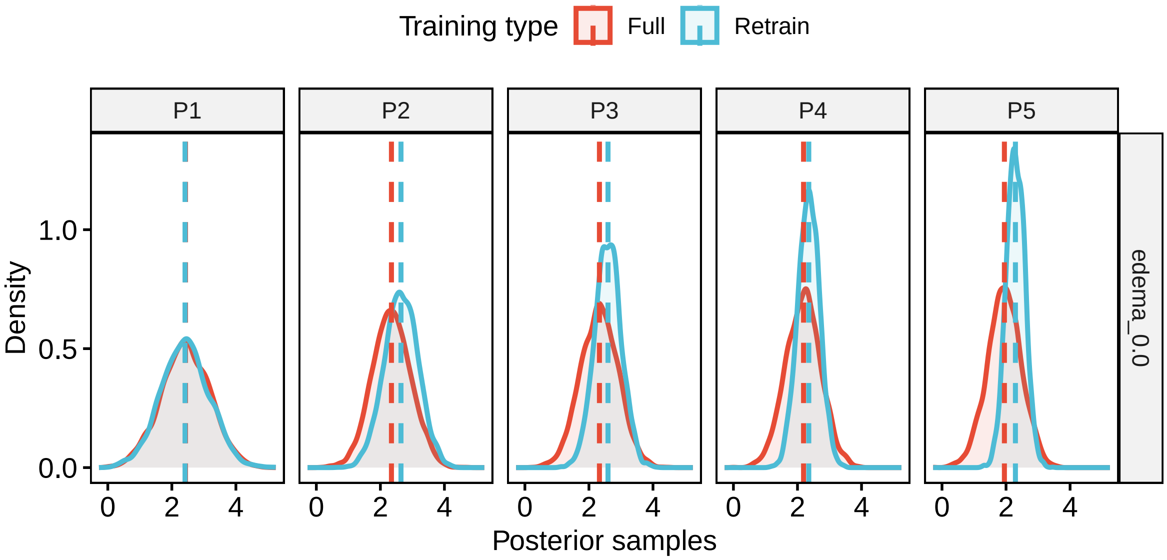

Training type Full Retrain
P1
P2
P3
P4
P5
edema_0.0
Density
1.0
0.5
0.0
0
2
4
Posterior samples

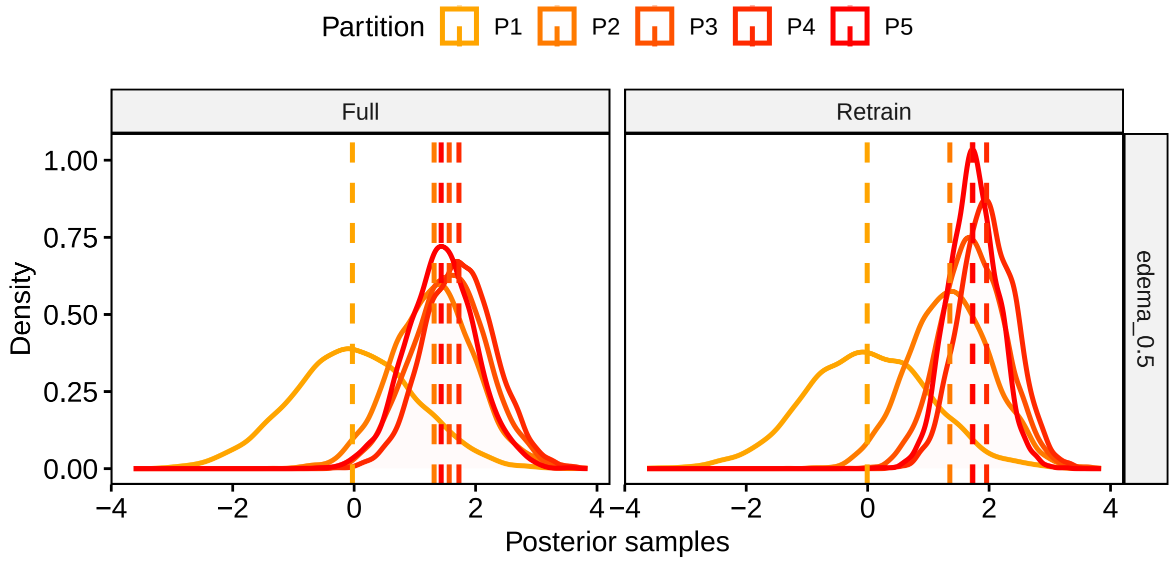
Partition
P1
P2
P3
P4
P5
Full
Retrain
edema_0.5
Density
1.00
0.75
0.50
0.25
0.00
−4
−2
0
2
4
Posterior samples

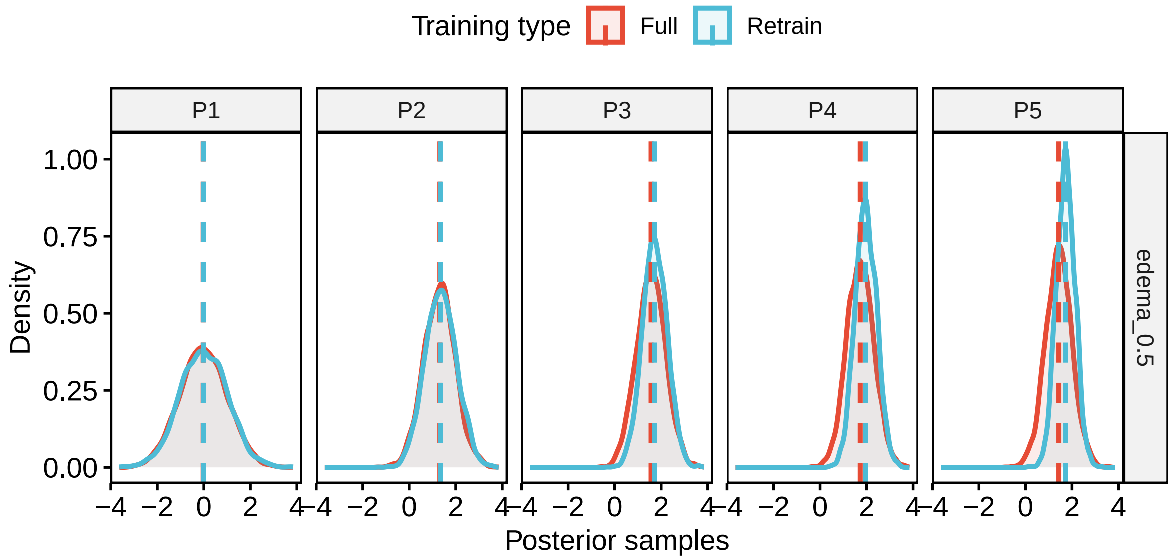
Training type
Full
Retrain
P1
P2
P3
P4
P5
edema_0.5
Density
1.00
0.75
0.50
0.25
0.00
−4
−2
0
2
4
Posterior samples

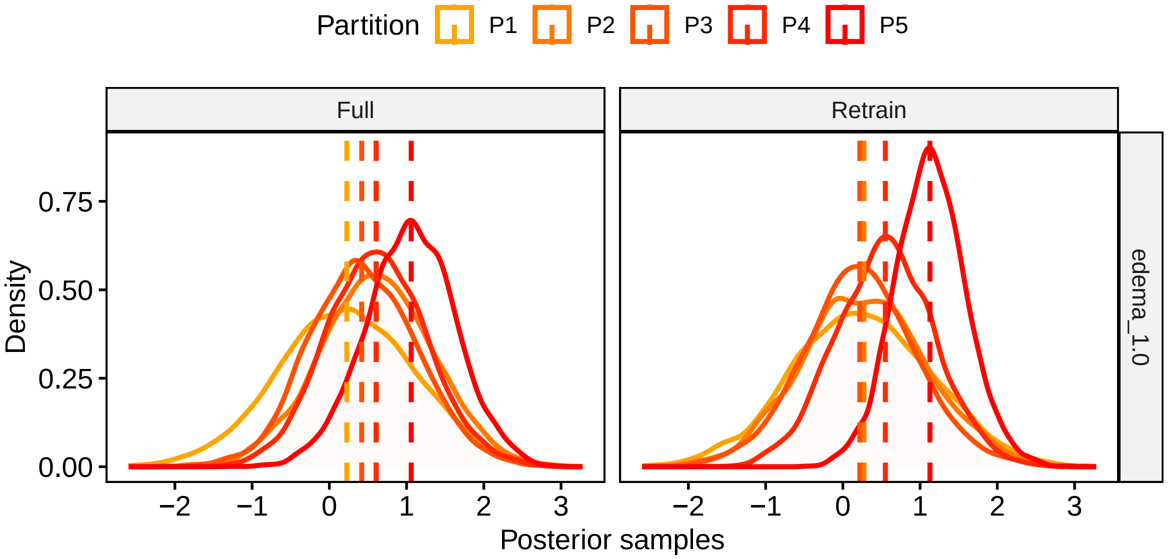
Partition
P1
P2
P3
P4
P5
Full
Retrain
edema_1.0
Density
0.75
0.50
0.25
0.00
−2
−1
0
1
2
3
Posterior samples

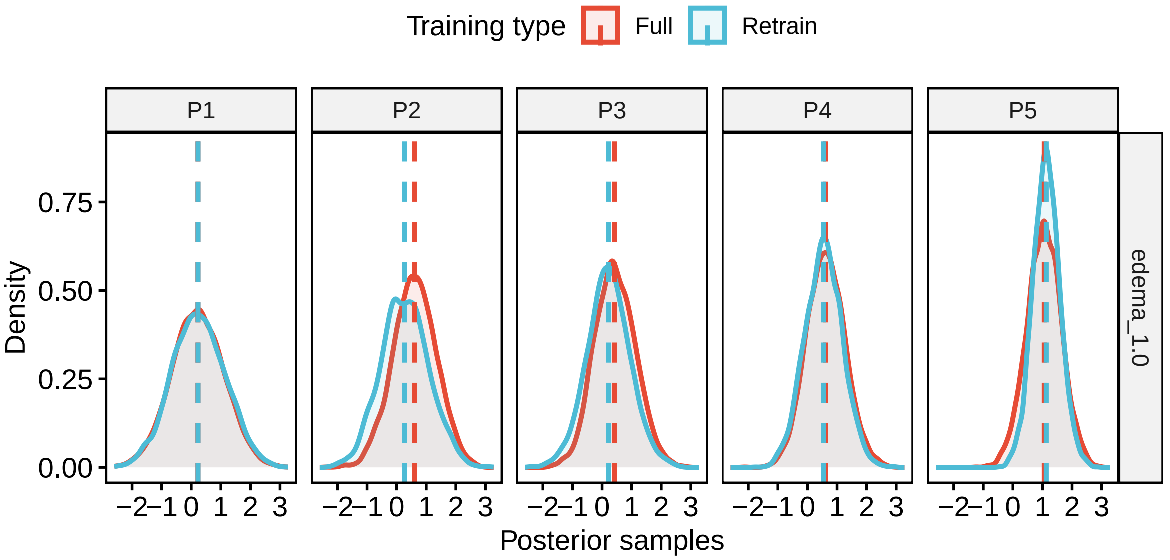
Training type
Full
Retrain
P1
P2
P3
P4
P5
edema_1.0
Density
0.75
0.50
0.25
0.00
−2−1 0 1 2 3
Posterior samples

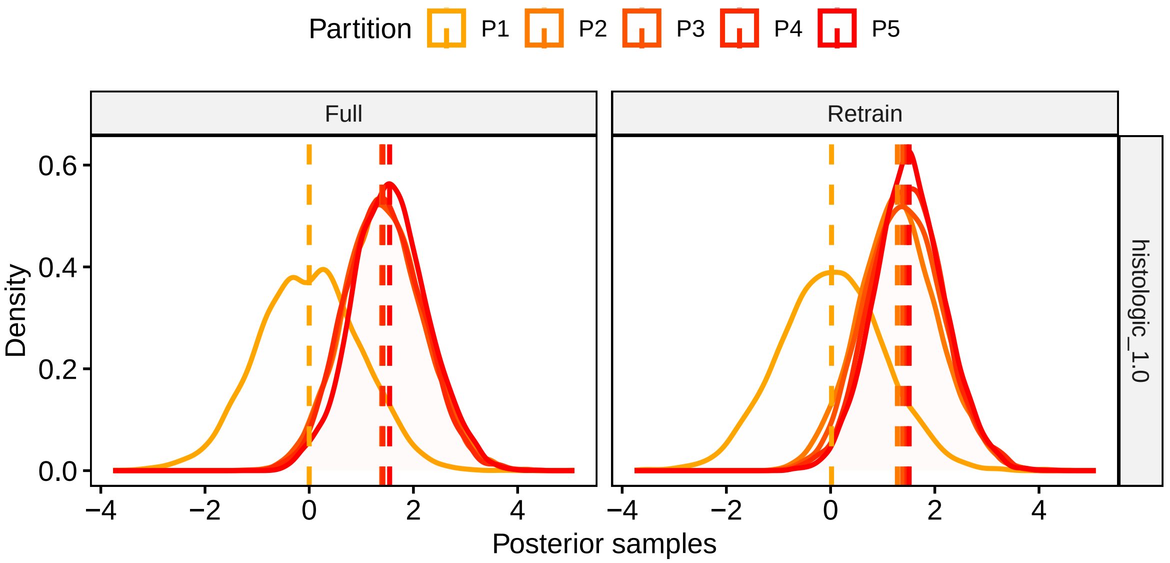
Partition
P1
P2
P3
P4
P5
Full
Retrain
histologic_1.0
Density
0.0
0.2
0.4
0.6
−4
−2
0
2
4
Posterior samples

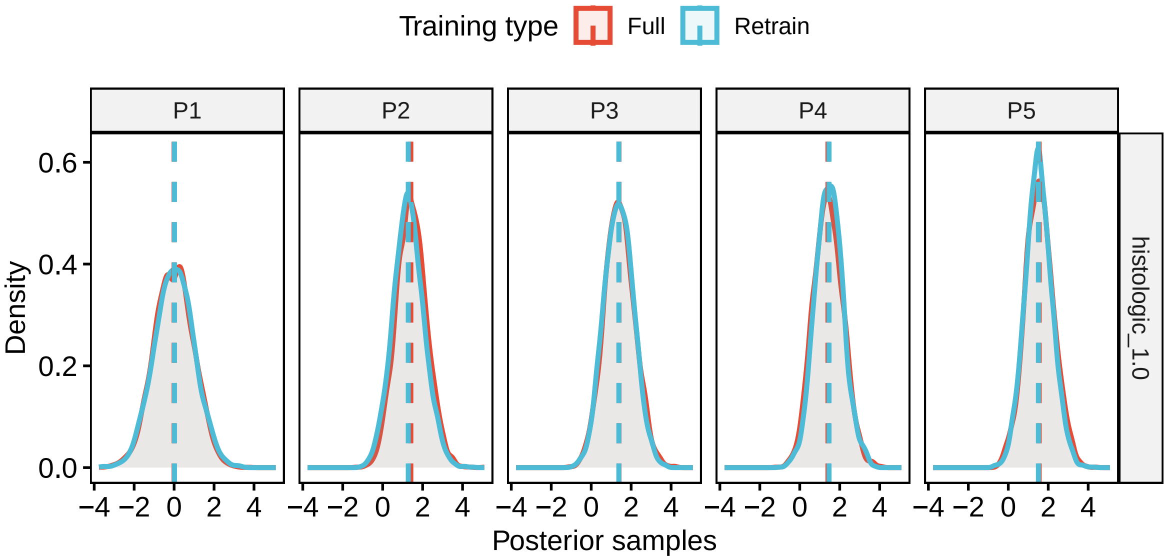
Training type
Full
Retrain
P1
P2
P3
P4
P5
histologic_1.0
Density
0.0
0.2
0.4
0.6
−4
−2
0
2
4
Posterior samples

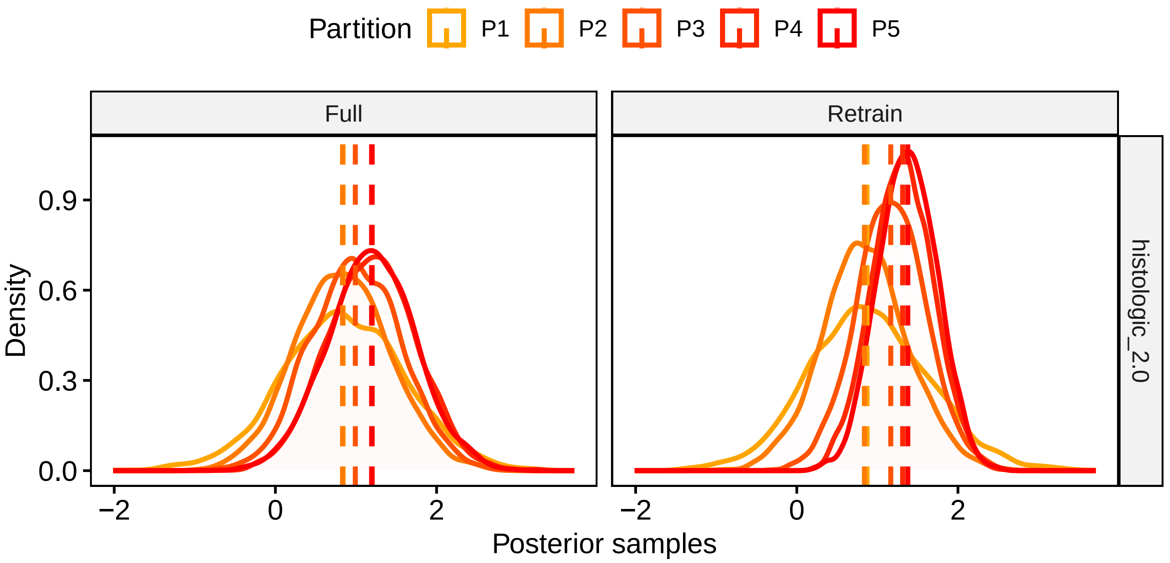
Partition
P1
P2
P3
P4
P5
Full
Retrain
histologic_2.0
Density
0.9
0.6
0.3
0.0
−2
0
2
Posterior samples

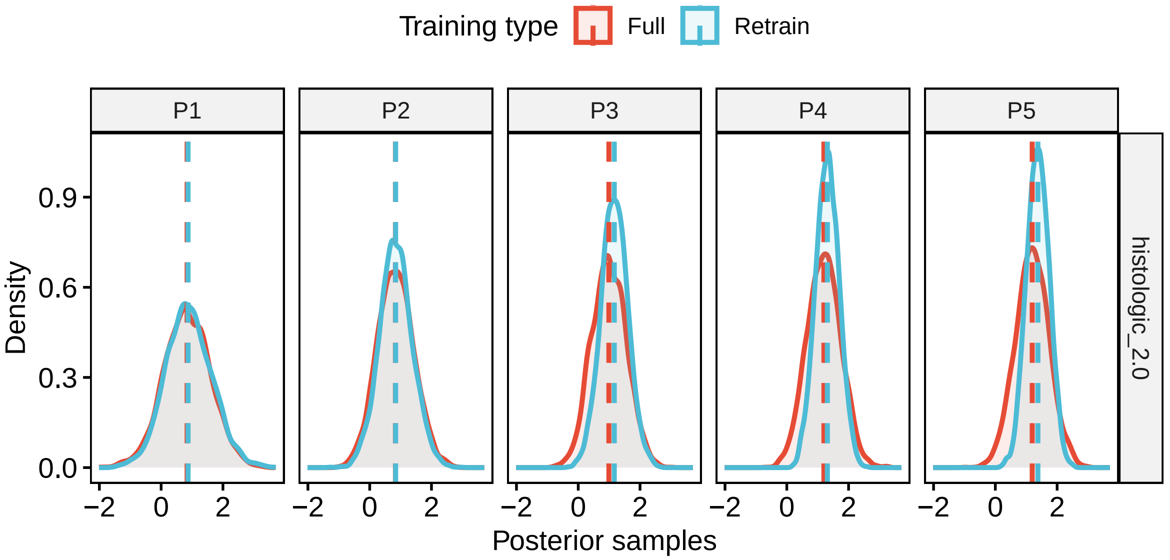
Training type
Full
Retrain
P1
P2
P3
P4
P5
histologic_2.0
Density
0.9
0.6
0.3
0.0
−2
0
2
Posterior samples

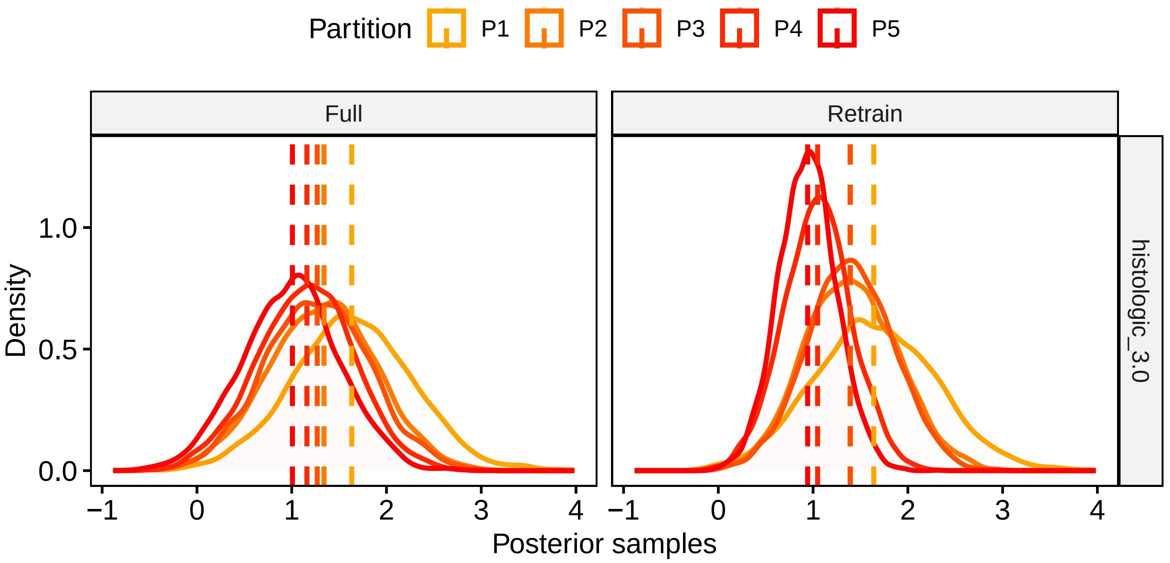
Partition
P1
P2
P3
P4
P5
Full
Retrain
histologic_3.0
Density
Posterior samples

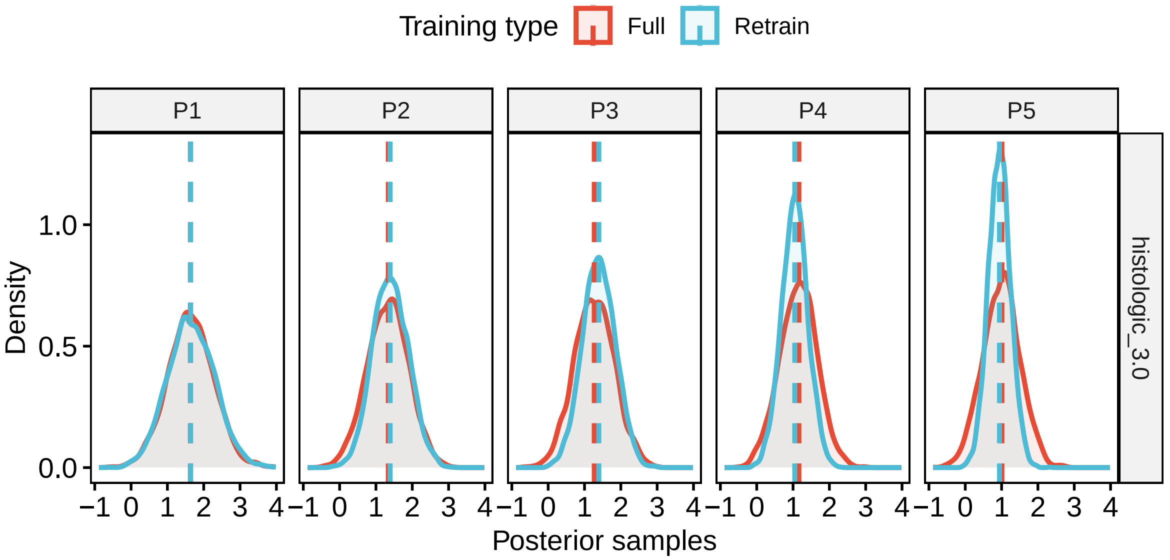
Training type
Full
Retrain
P1
P2
P3
P4
P5
histologic_3.0
Density
Posterior samples

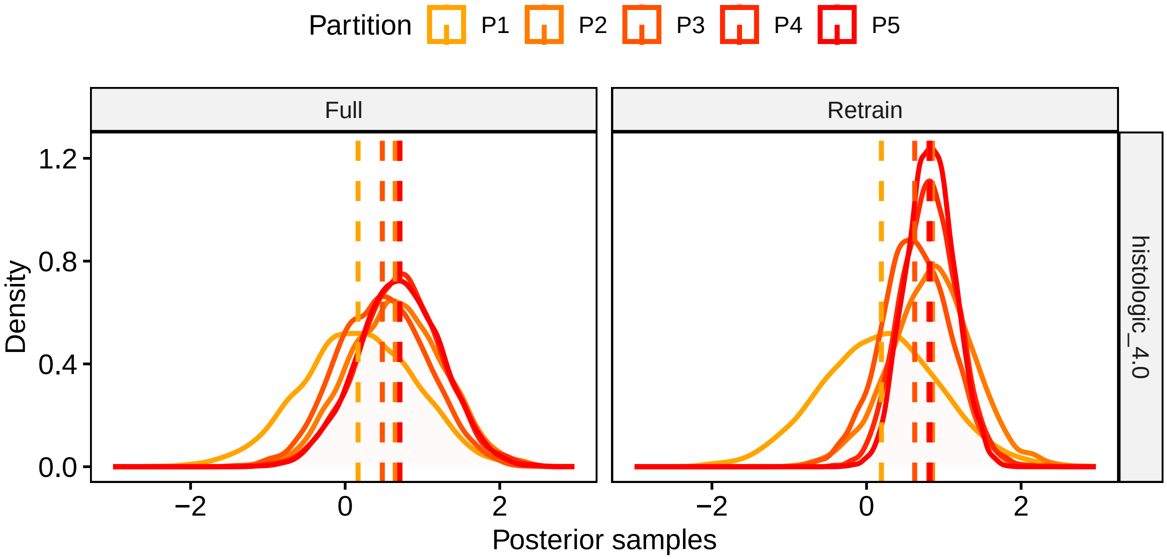
Partition
P1
P2
P3
P4
P5
Full
Retrain
histologic_4.0
Density
1.2
0.8
0.4
0.0
−2
0
2
Posterior samples

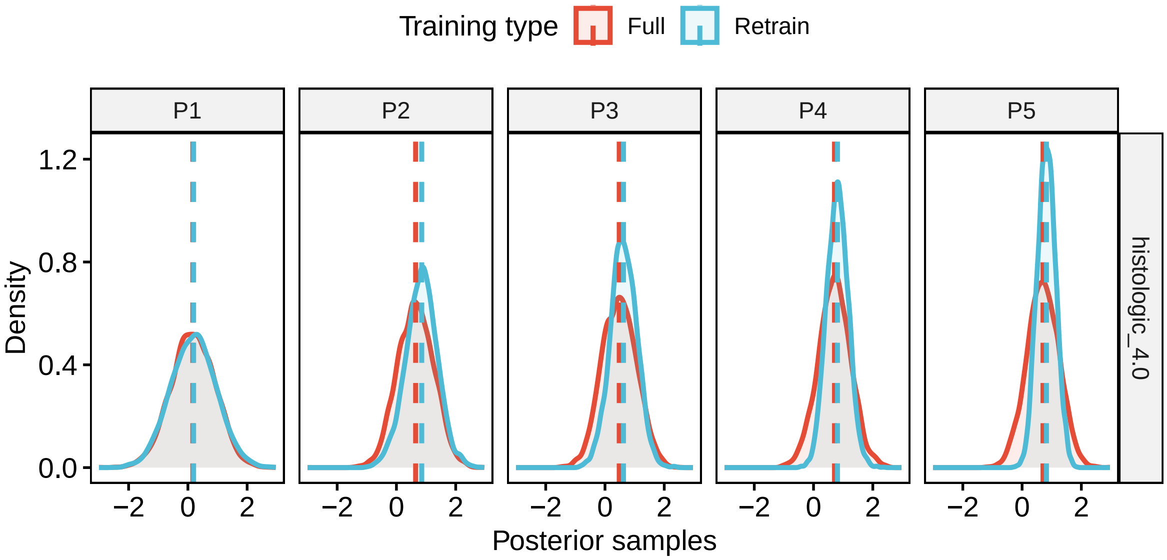
Training type
Full
Retrain
P1
P2
P3
P4
P5
histologic_4.0
Density
1.2
0.8
0.4
0.0
−2
0
2
Posterior samples

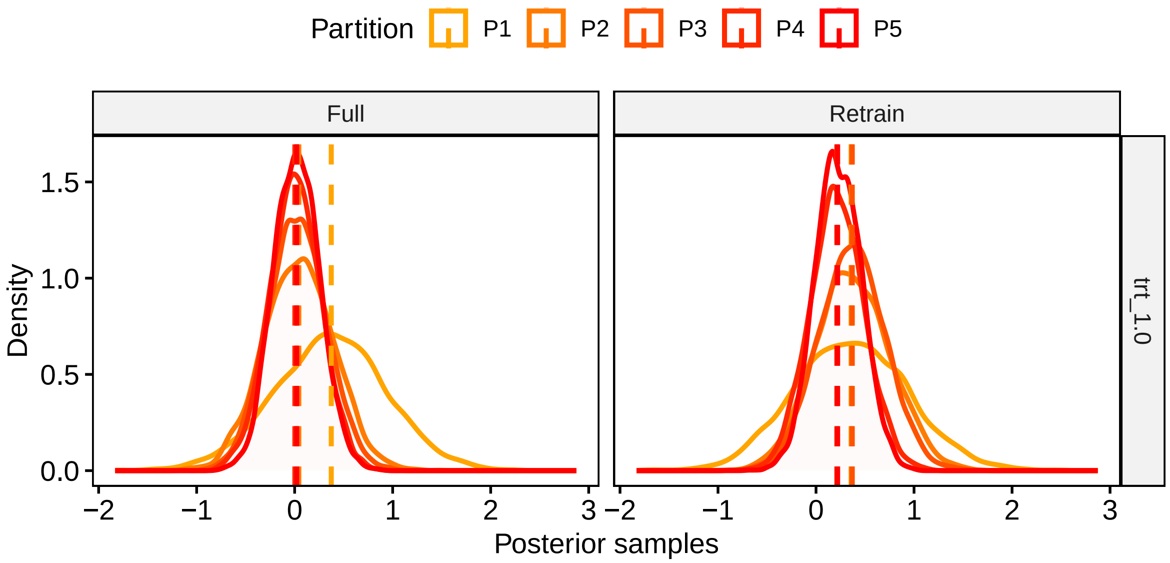
Partition
P1
P2
P3
P4
P5
Full
Retrain
trt_1.0
Density
Posterior samples

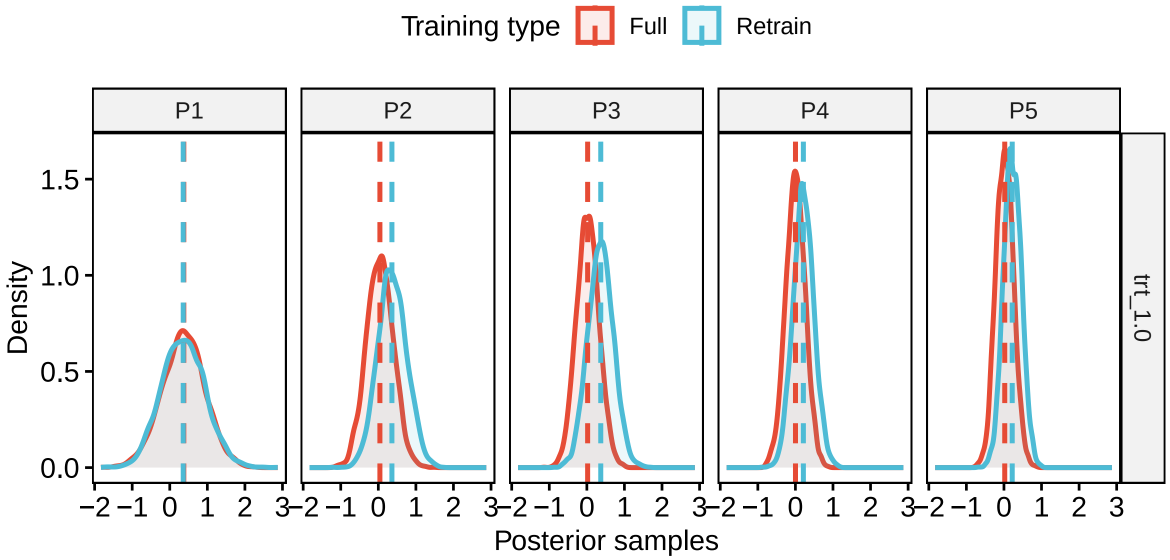
Training type
Full
Retrain
P1
P2
P3
P4
P5
trt_1.0
Density
Posterior samples

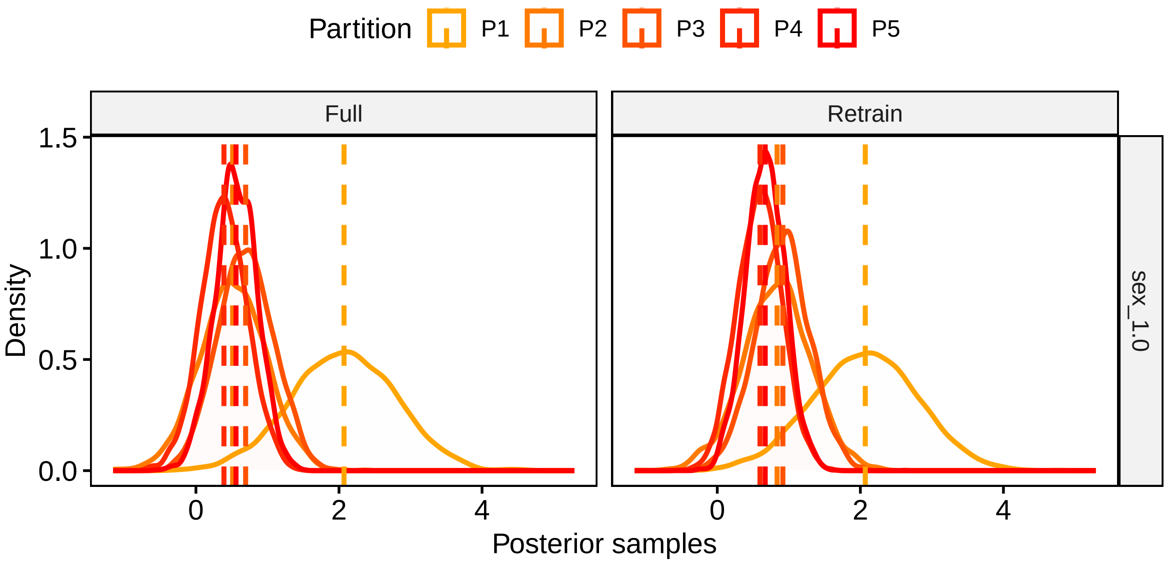

Partition
P1
P2
P3
P4
P5
Full
Retrain
sex_1.0
Density
Posterior samples

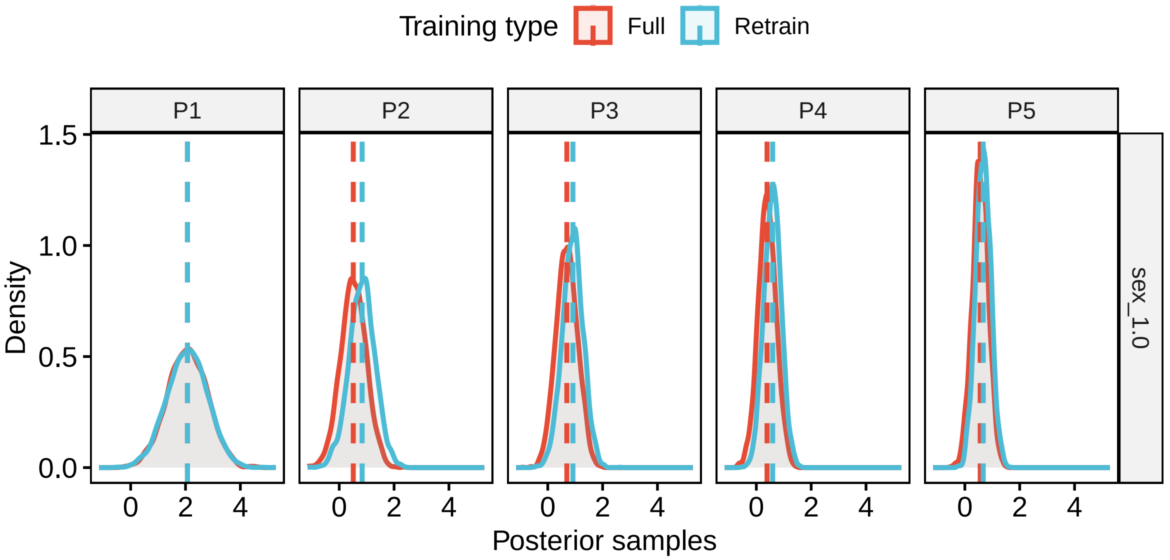

Training type
Full
Retrain
P1
P2
P3
P4
P5
sex_1.0
Density
Posterior samples

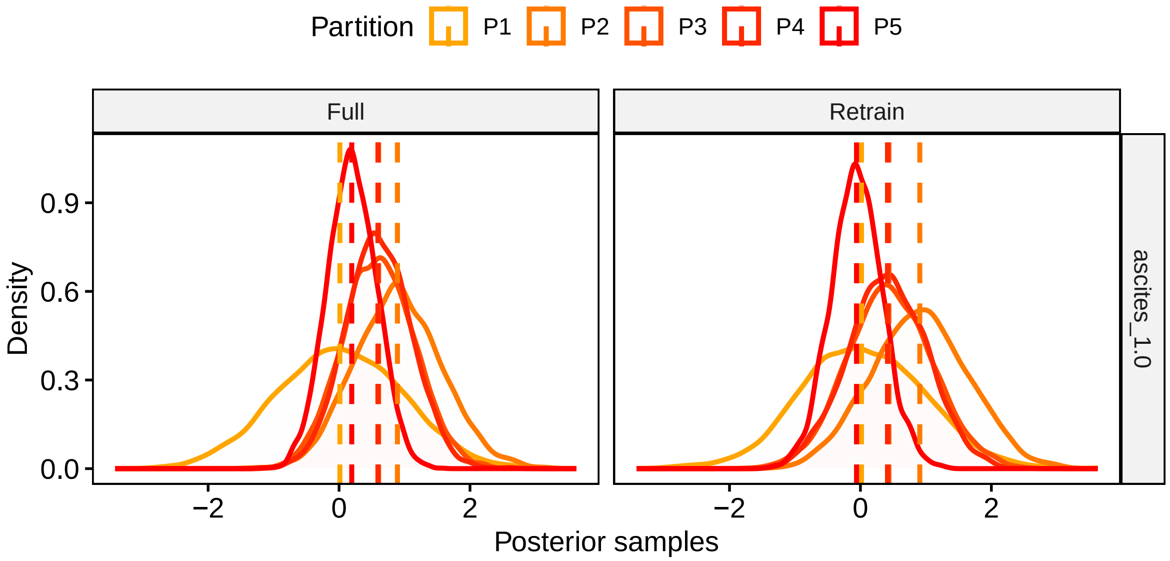

Partition
P1
P2
P3
P4
P5
Full
Retrain
ascites_1.0
Density
0.0
0.3
0.6
0.9
−2
0
2
Posterior samples

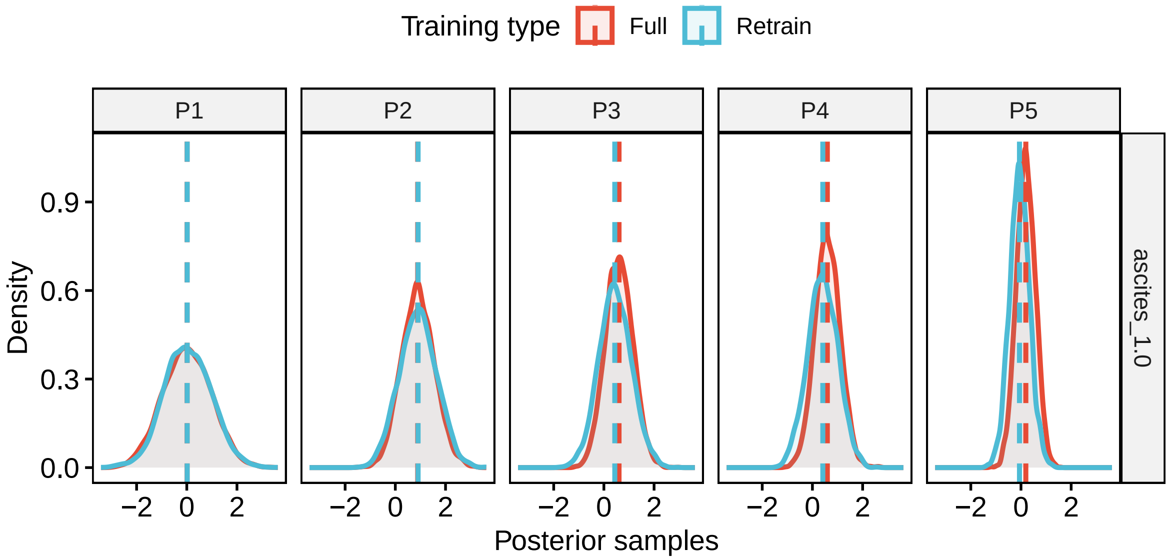

Training type
Full
Retrain
P1
P2
P3
P4
P5
ascites_1.0
Density
0.0
0.3
0.6
0.9
−2
0
2
Posterior samples

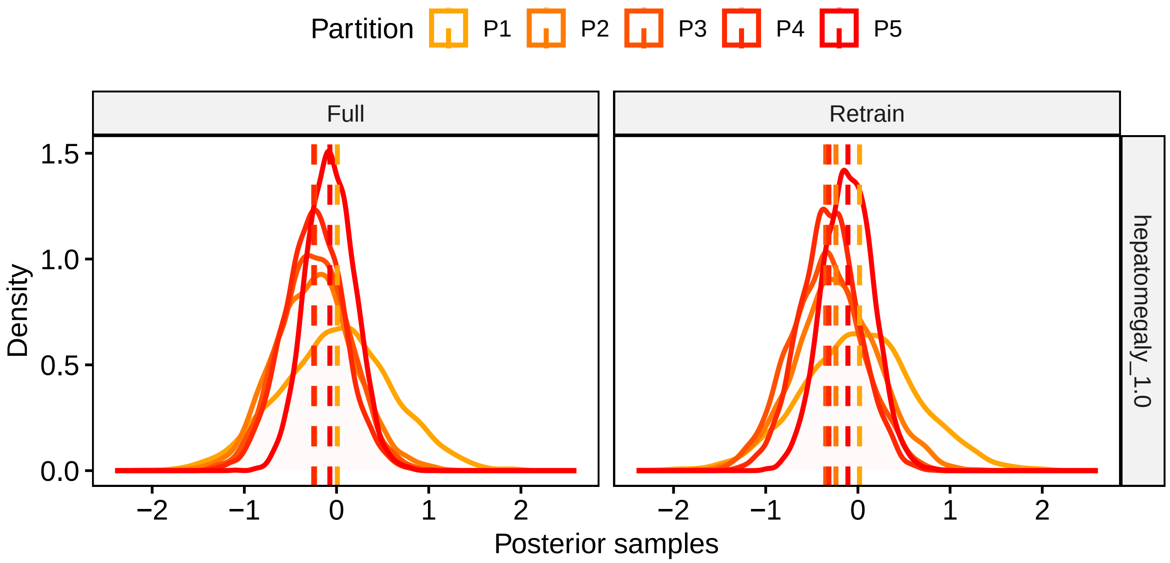
Partition
P1
P2
P3
P4
P5
Full
Retrain
hepatomegaly_1.0
Density
Posterior samples

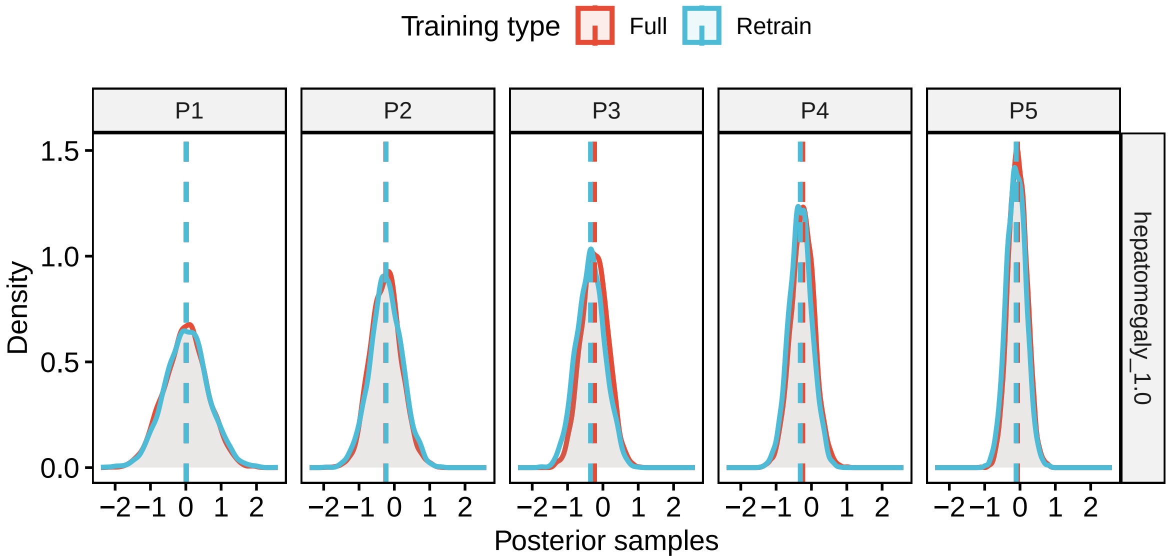
Training type
Full
Retrain
P1
P2
P3
P4
P5
hepatomegaly_1.0
Density
Posterior samples

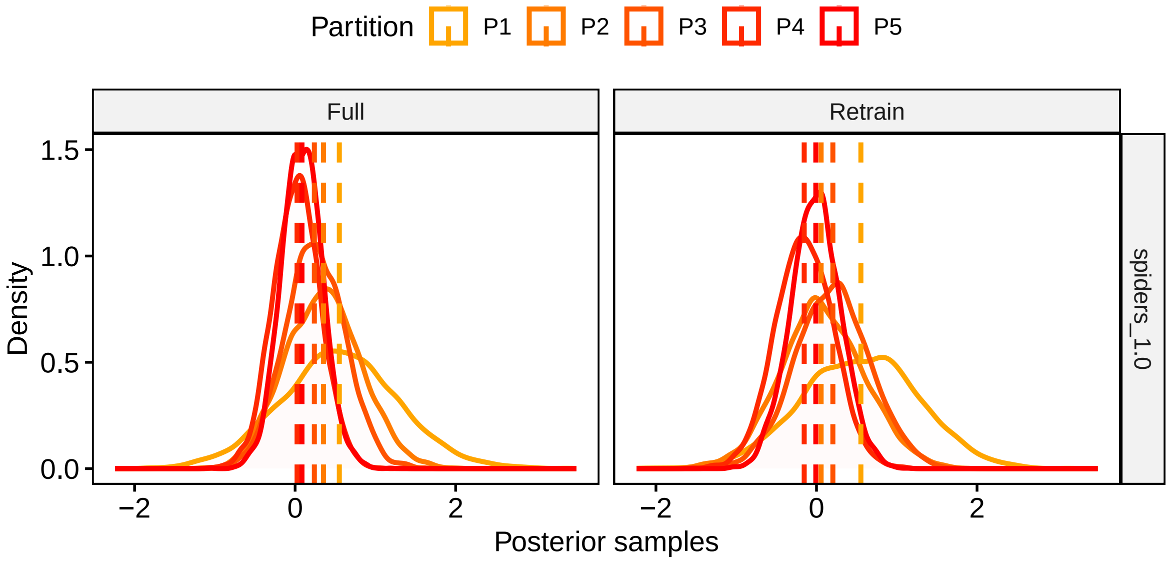
Partition
P1
P2
P3
P4
P5
Full
Retrain
spiders_1.0
Density
1.5
1.0
0.5
0.0
−2
0
2
Posterior samples

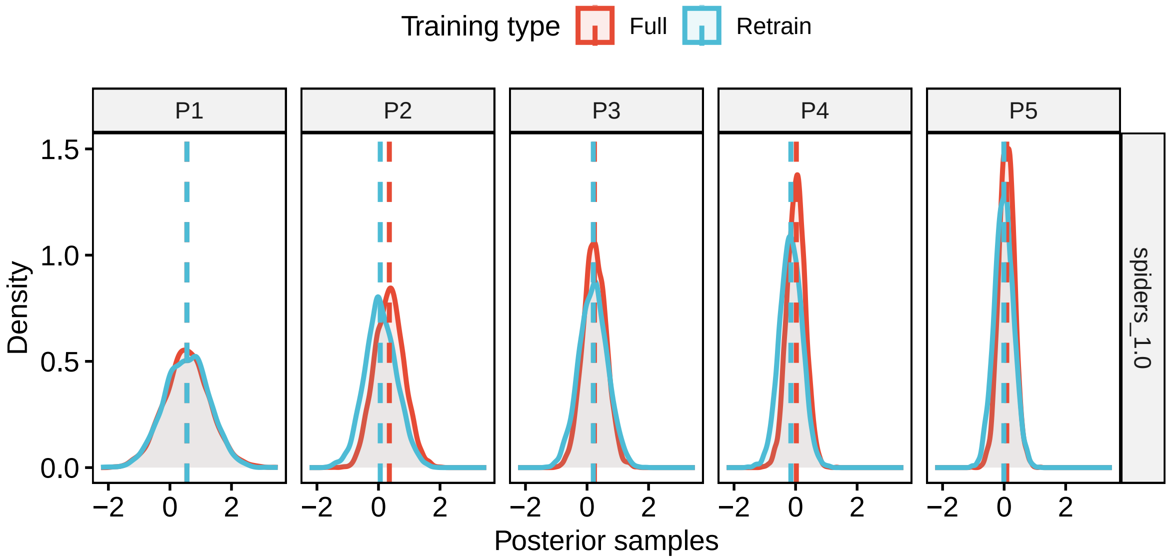
Training type
Full
Retrain
P1
P2
P3
P4
P5
spiders_1.0
Density
1.5
1.0
0.5
0.0
−2
0
2
Posterior samples

ACTG

```
pbc_vars <- data %>% filter(experiment == 'actg' & model == 'pm_exp') %>% select(var) %>%


for(var_idx in unique(pbc_vars$var)){
  p1 <- plot_density(data, 'actg', 'pm_exp', var_idx)
  plot(p1)

  p <- plot_density_2(data, 'actg', 'pm_exp', var_idx)
  plot(p)
}
```

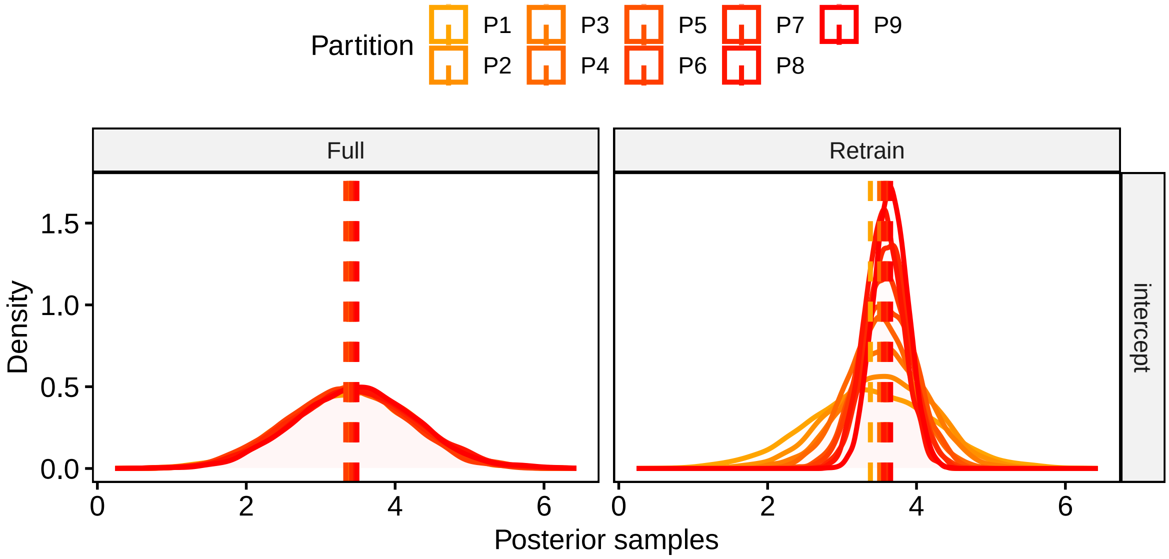

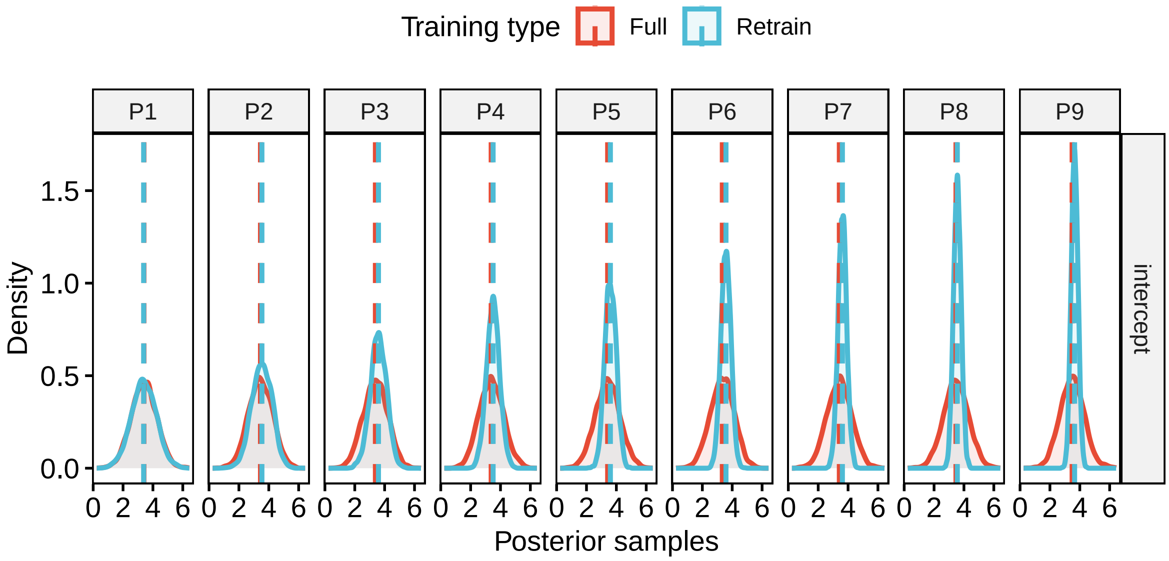
Training type
Full
Retrain
P1
P2
P3
P4
P5
P6
P7
P8
P9
intercept
Density
1.5
1.0
0.5
0.0
0 2 4 6
Posterior samples

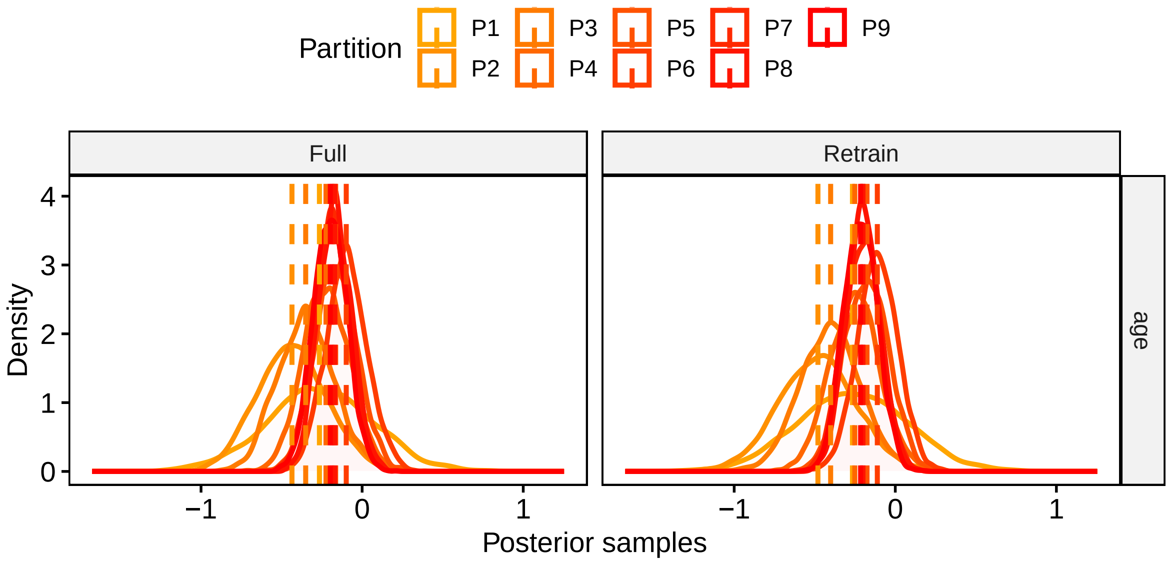
Partition
P1
P2
P3
P4
P5
P6
P7
P8
P9
Full
Retrain
age
Density
4
3
2
1
0
−1
0
1
Posterior samples

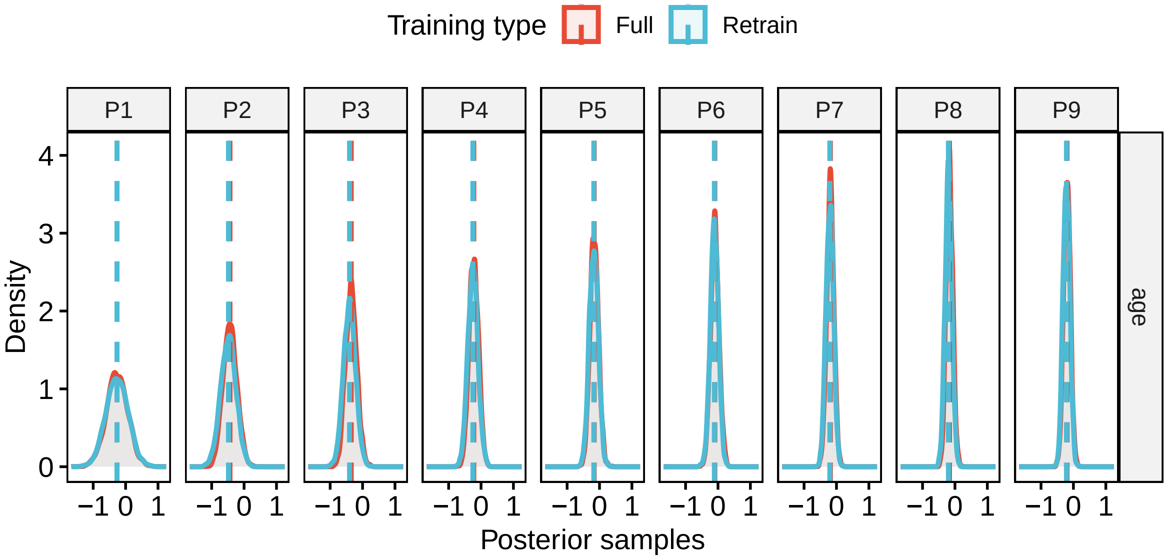
Training type
Full
Retrain
P1
P2
P3
P4
P5
P6
P7
P8
P9
Density
Posterior samples
age

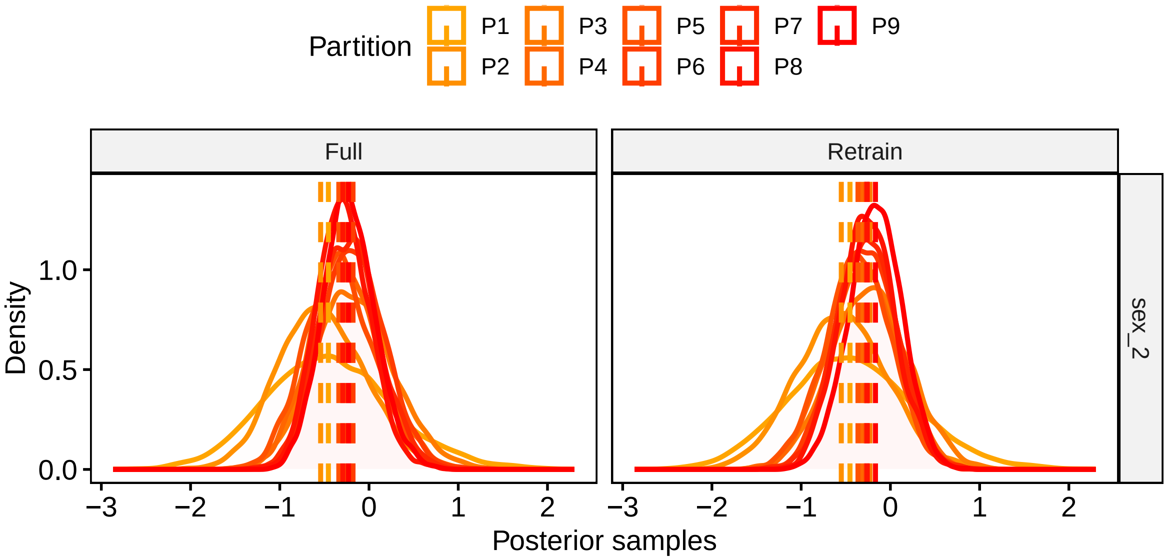
Partition
P1
P2
P3
P4
P5
P6
P7
P8
P9
Full
Retrain
Density
Posterior samples
sex_2

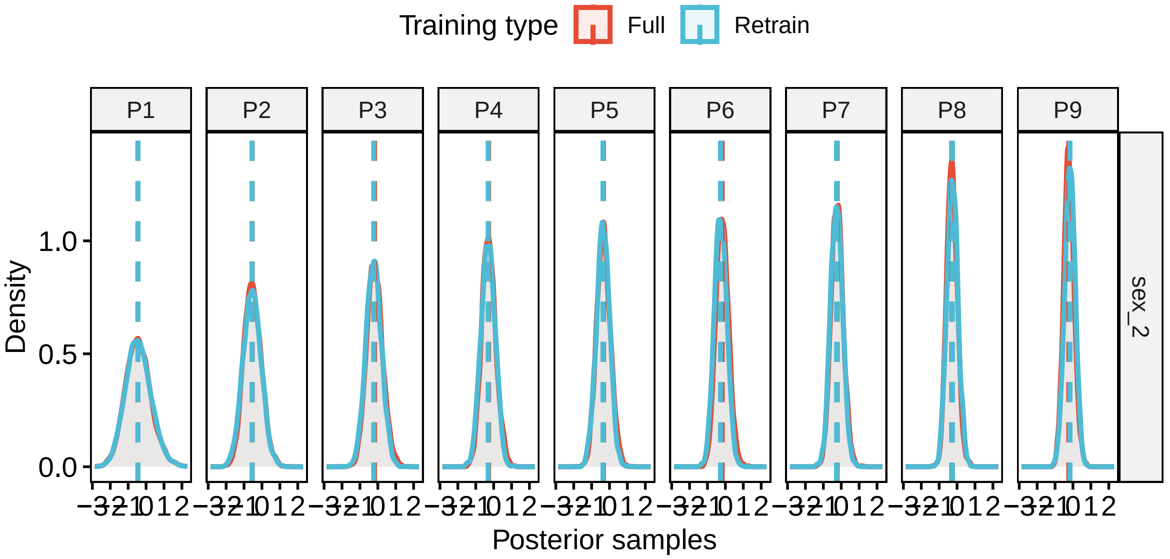
Training type
Full
Retrain
P1
P2
P3
P4
P5
P6
P7
P8
P9
sex_2
Density
1.0
0.5
0.0
Posterior samples

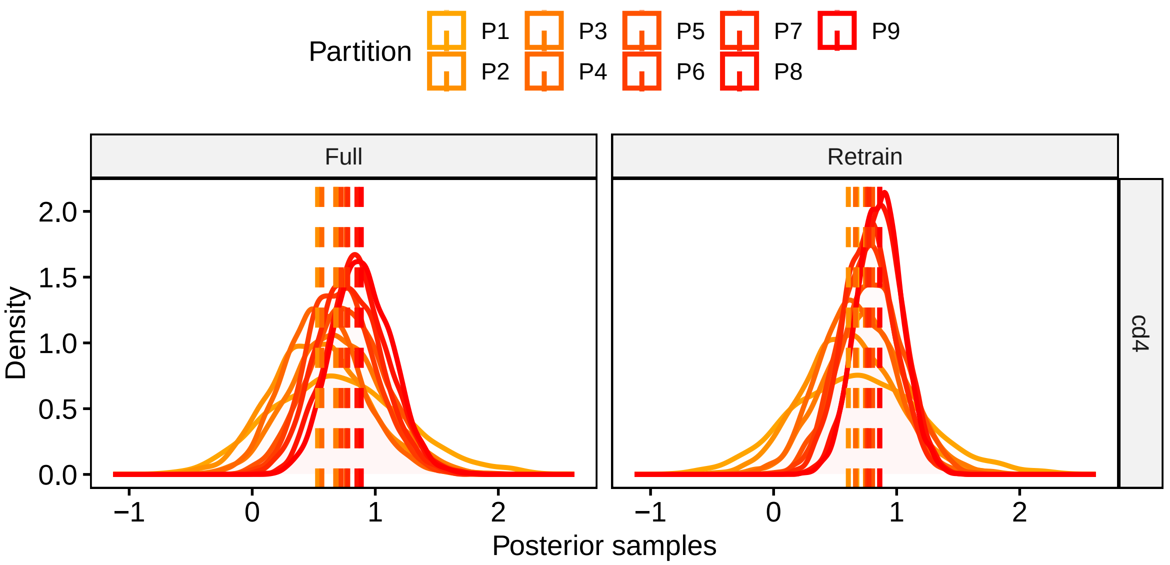
Partition
P1
P2
P3
P4
P5
P6
P7
P8
P9
Full
Retrain
cd4
Density
2.0
1.5
1.0
0.5
0.0
−1
0
1
2
Posterior samples

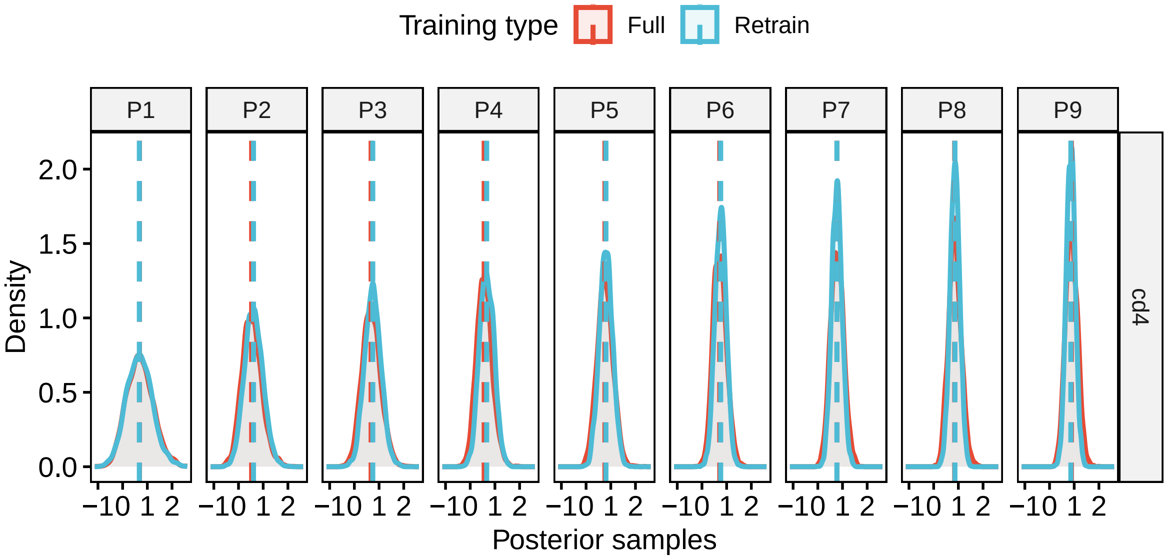
Training type
Full
Retrain
P1
P2
P3
P4
P5
P6
P7
P8
P9
cd4
Density
Posterior samples

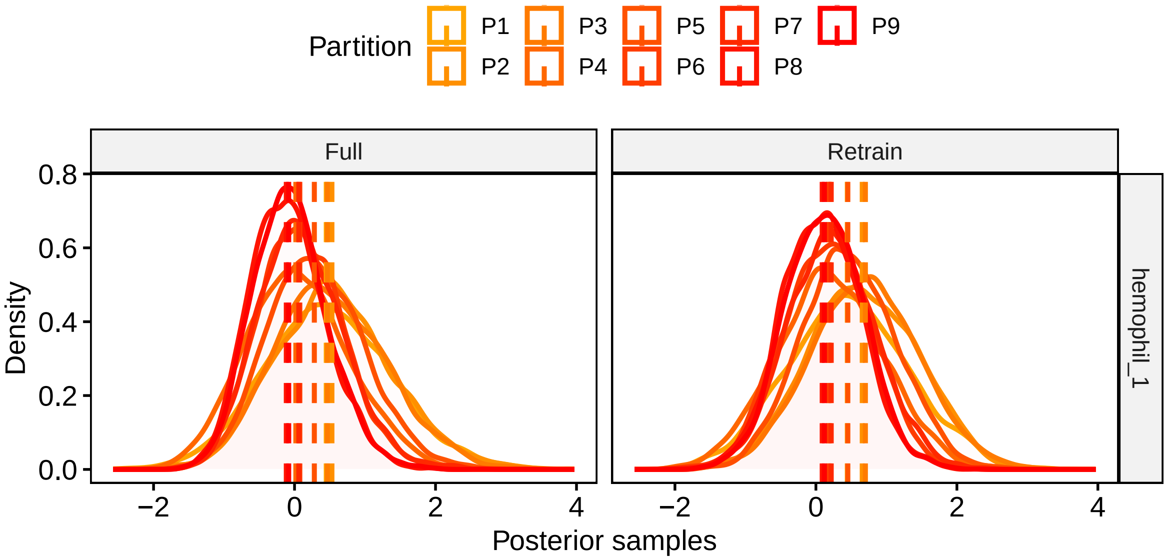
Partition
P1
P2
P3
P4
P5
P6
P7
P8
P9
Full
Retrain
hemophil_1
Density
Posterior samples

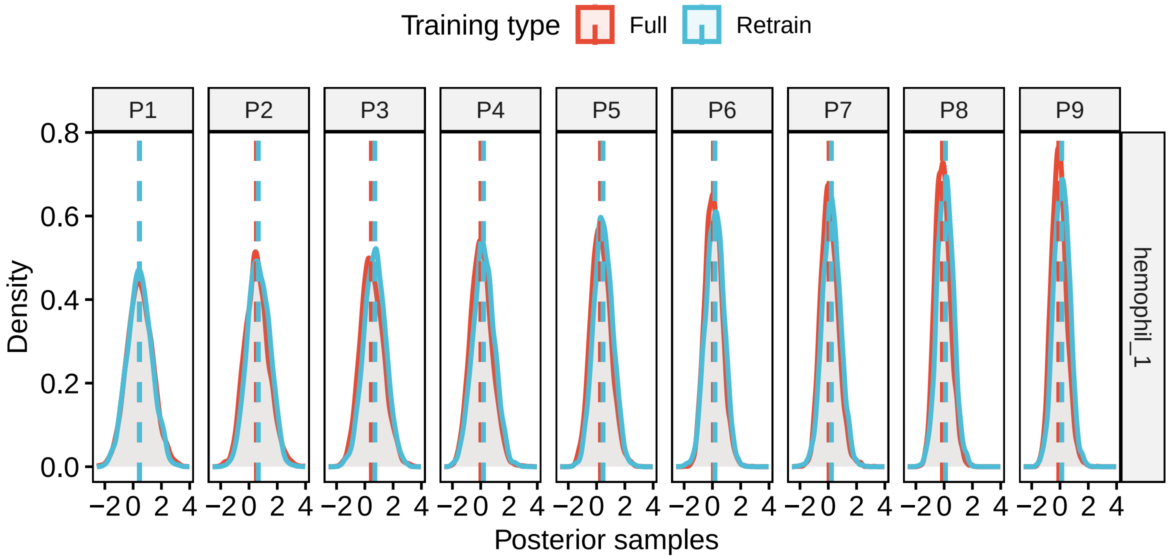
Training type
Full
Retrain
P1
P2
P3
P4
P5
P6
P7
P8
P9
hemophil_1
Density
Posterior samples

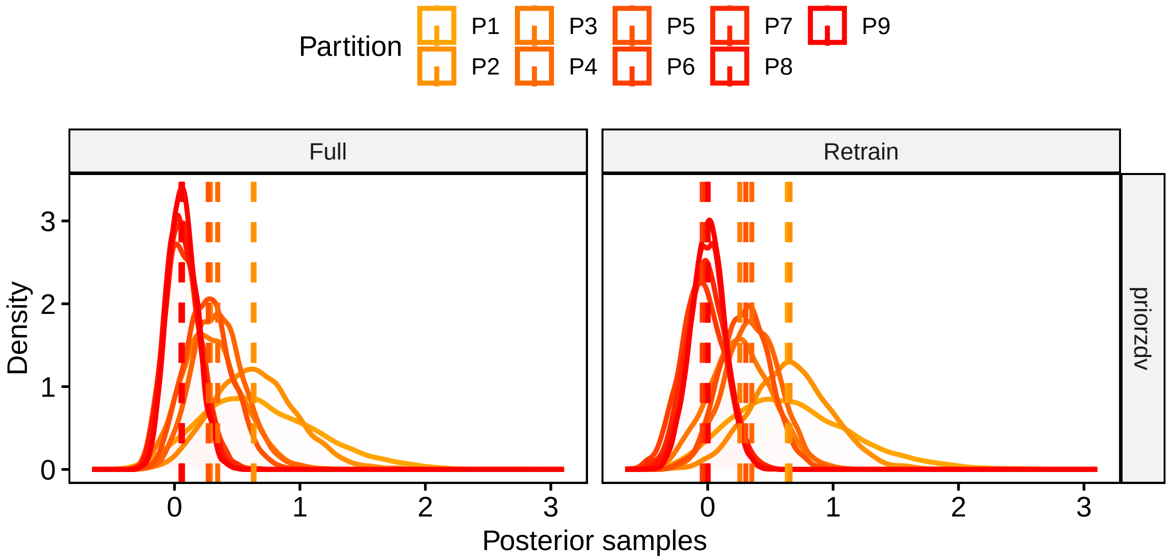
Partition
P1
P2
P3
P4
P5
P6
P7
P8
P9
Full
Retrain
priorzdv
Density
Posterior samples

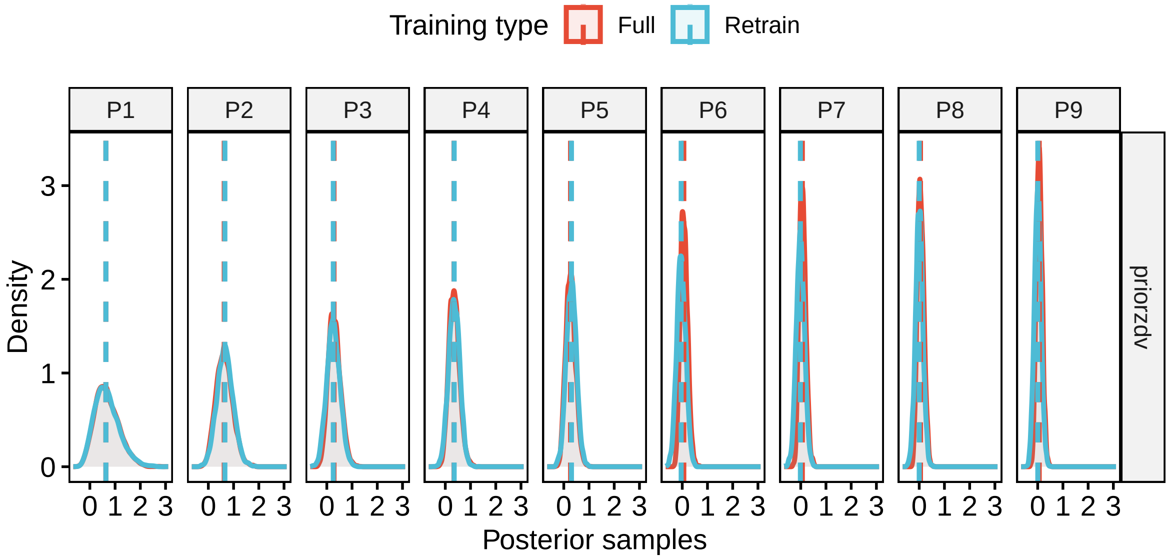
Training type
Full
Retrain
P1
P2
P3
P4
P5
P6
P7
P8
P9
Density
priorzdv
Posterior samples

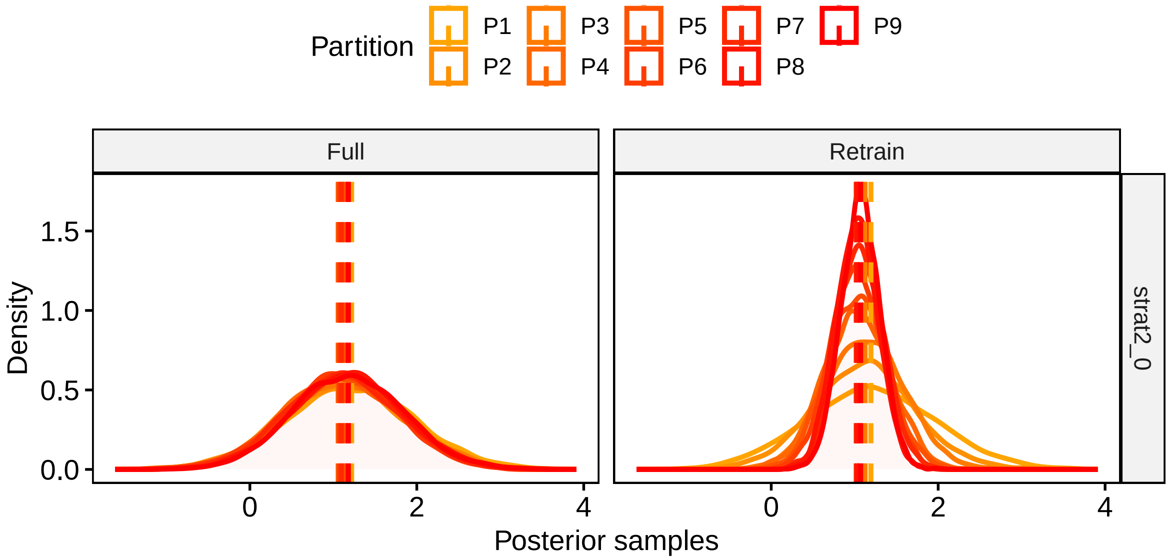
Partition
P1
P2
P3
P4
P5
P6
P7
P8
P9
Full
Retrain
Density
strat2_0
Posterior samples

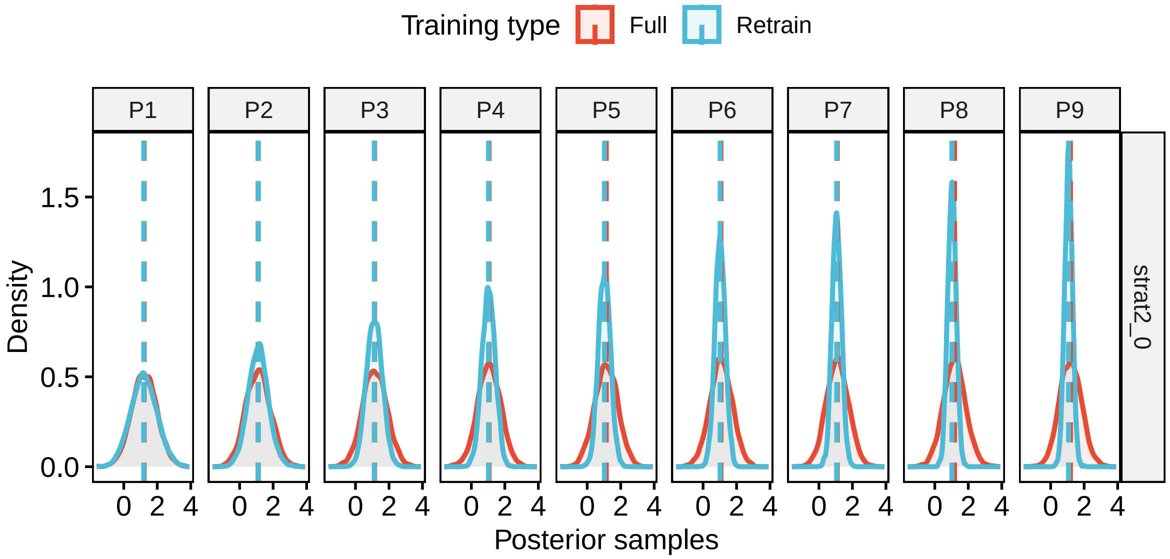
Training type
Full
Retrain
P1
P2
P3
P4
P5
P6
P7
P8
P9
Density
strat2_0
1.5
1.0
0.5
0.0
0 2 4
Posterior samples

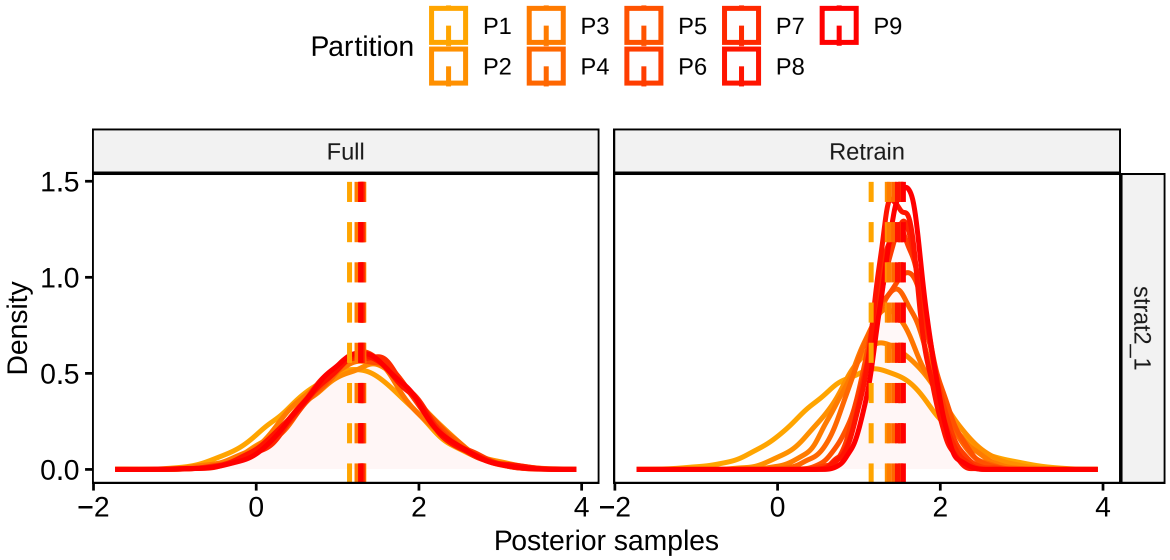
Partition
P1
P2
P3
P4
P5
P6
P7
P8
P9
Full
Retrain
strat2_1
Density
1.5
1.0
0.5
0.0
−2
0
2
4
Posterior samples

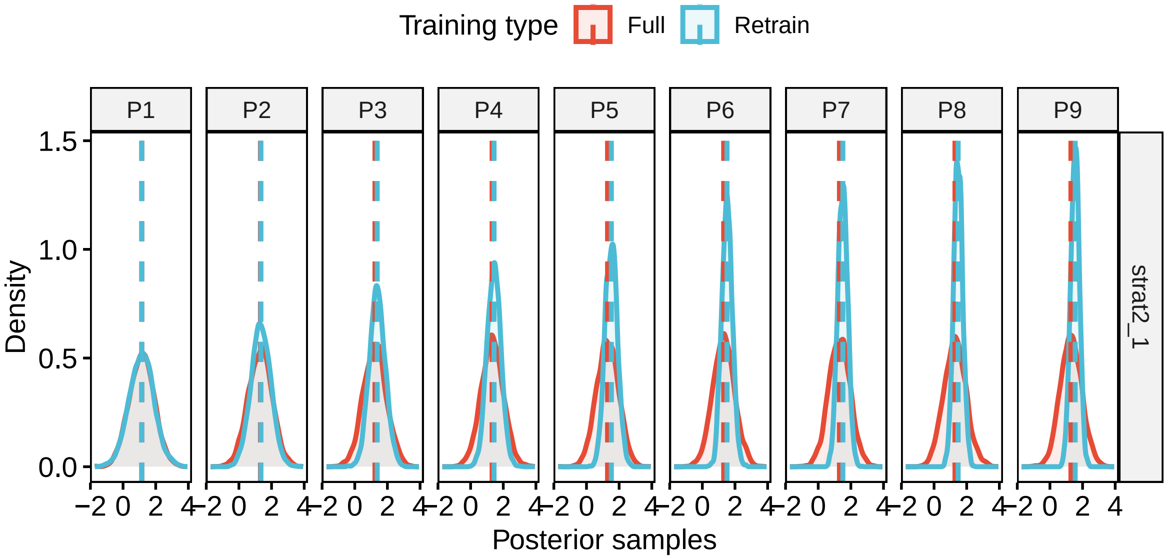

Training type
Full
Retrain
P1
P2
P3
P4
P5
P6
P7
P8
P9
strat2_1
Density
Posterior samples

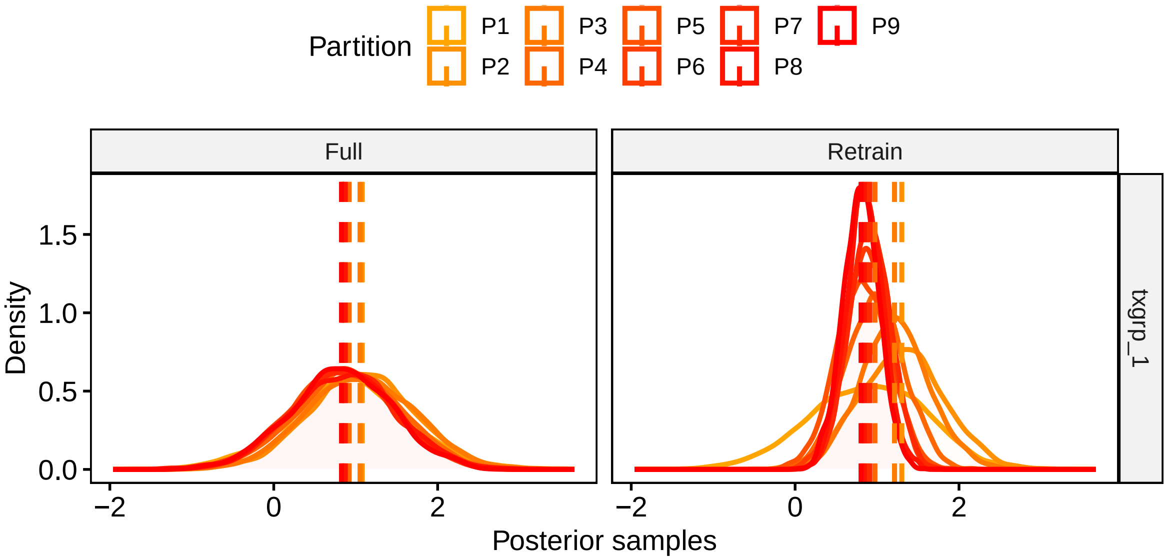

Partition
P1
P2
P3
P4
P5
P6
P7
P8
P9
Full
Retrain
txgrp_1
Density
Posterior samples

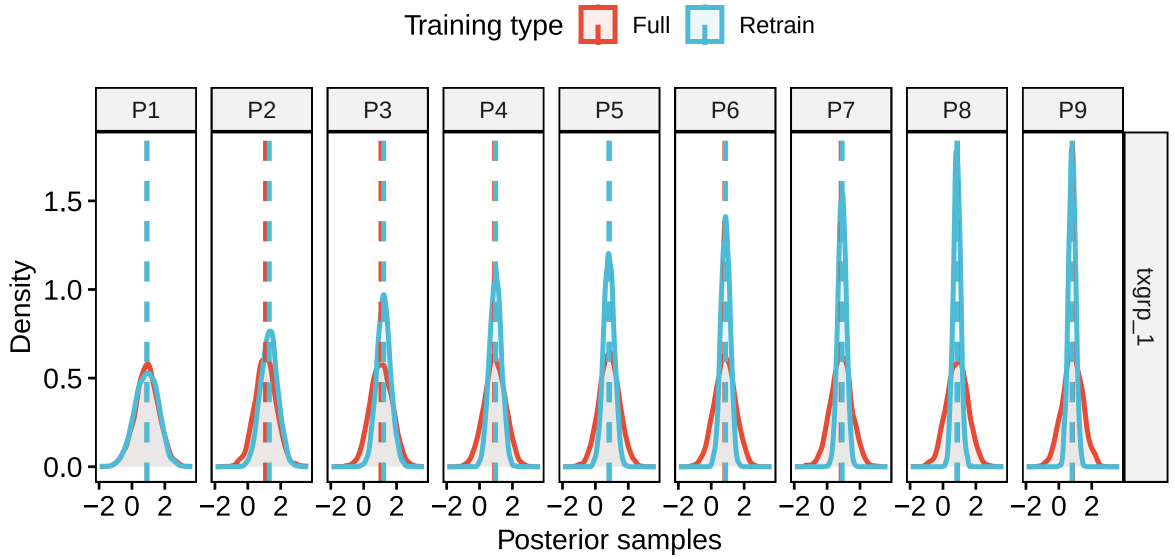

Training type
Full
Retrain
P1
P2
P3
P4
P5
P6
P7
P8
P9
txgrp_1
Density
Posterior samples

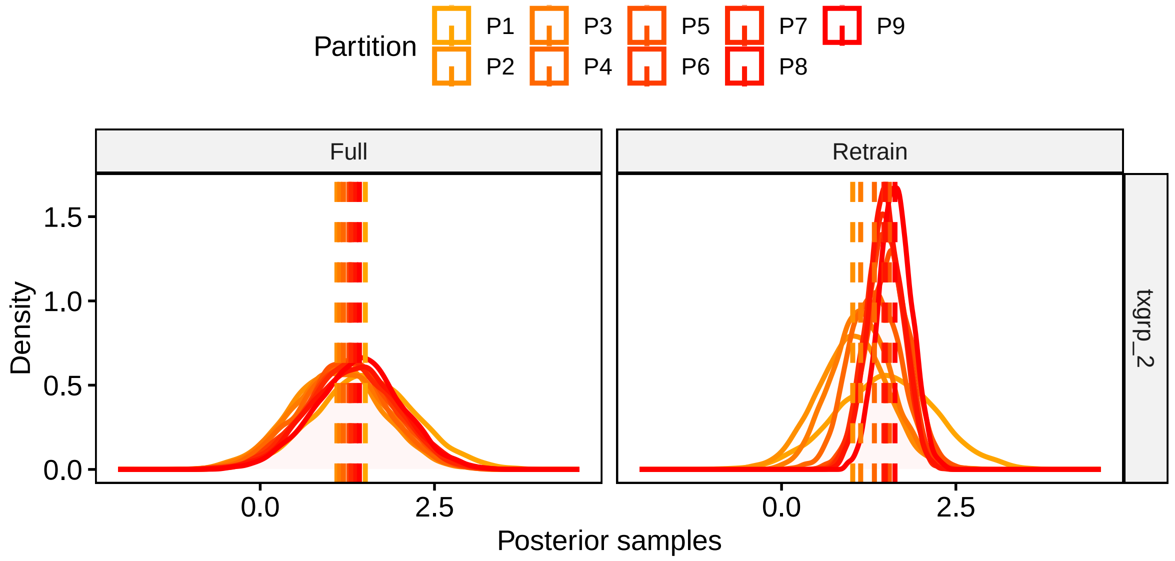

Partition
P1
P2
P3
P4
P5
P6
P7
P8
P9
Full
Retrain
txgrp_2
Density
Posterior samples

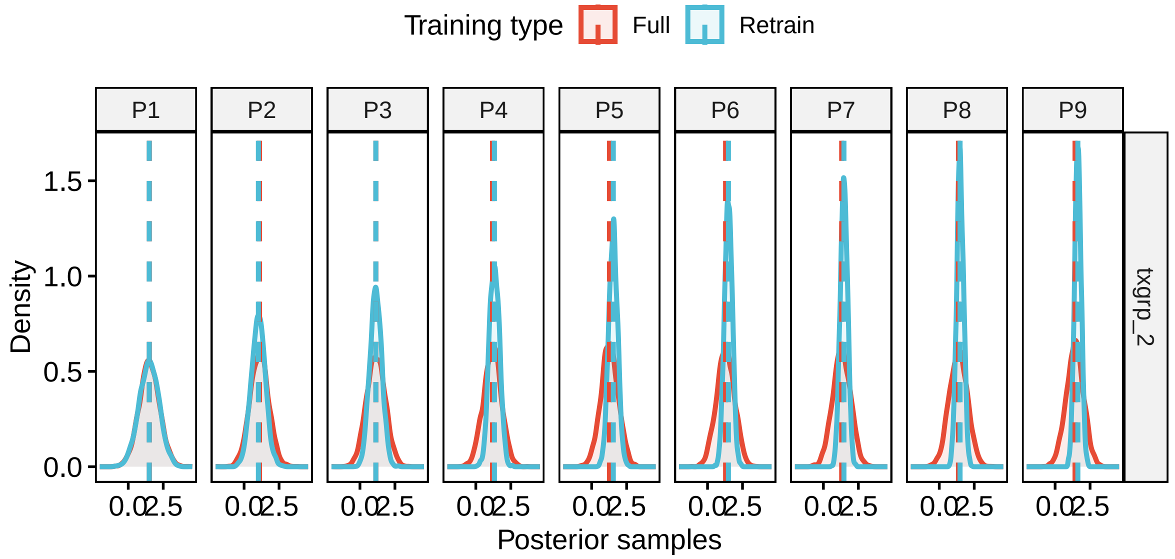
Training type
Full
Retrain
P1
P2
P3
P4
P5
P6
P7
P8
P9
Density
1.5
1.0
0.5
0.0
0.0 2.5
Posterior samples
txgrp_2

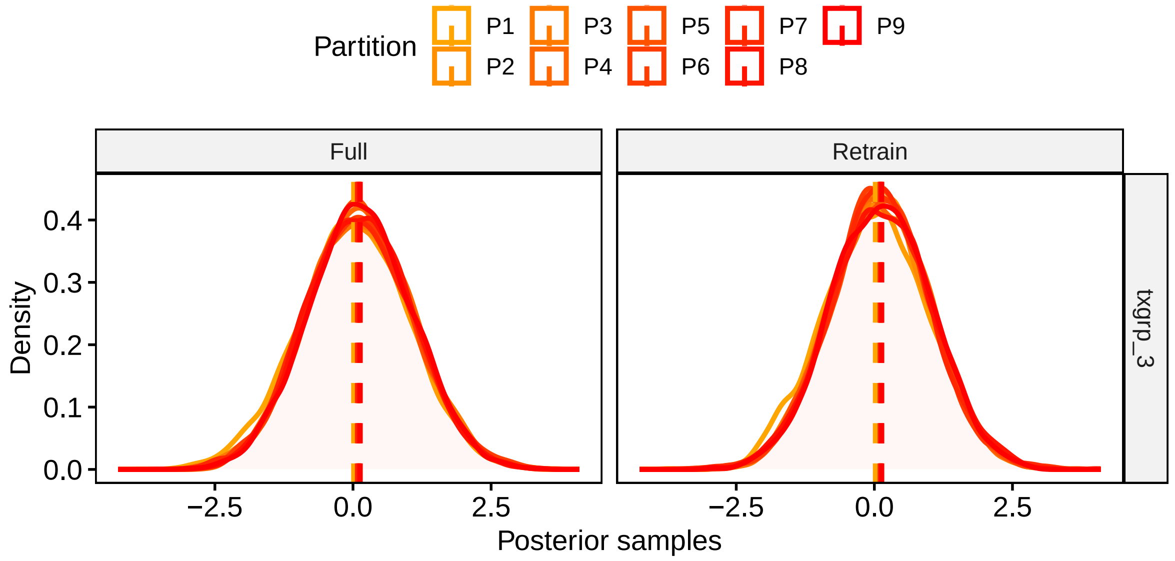
Partition
P1
P2
P3
P4
P5
P6
P7
P8
P9
Full
Retrain
Density
0.4
0.3
0.2
0.1
0.0
−2.5
0.0
2.5
Posterior samples
txgrp_3

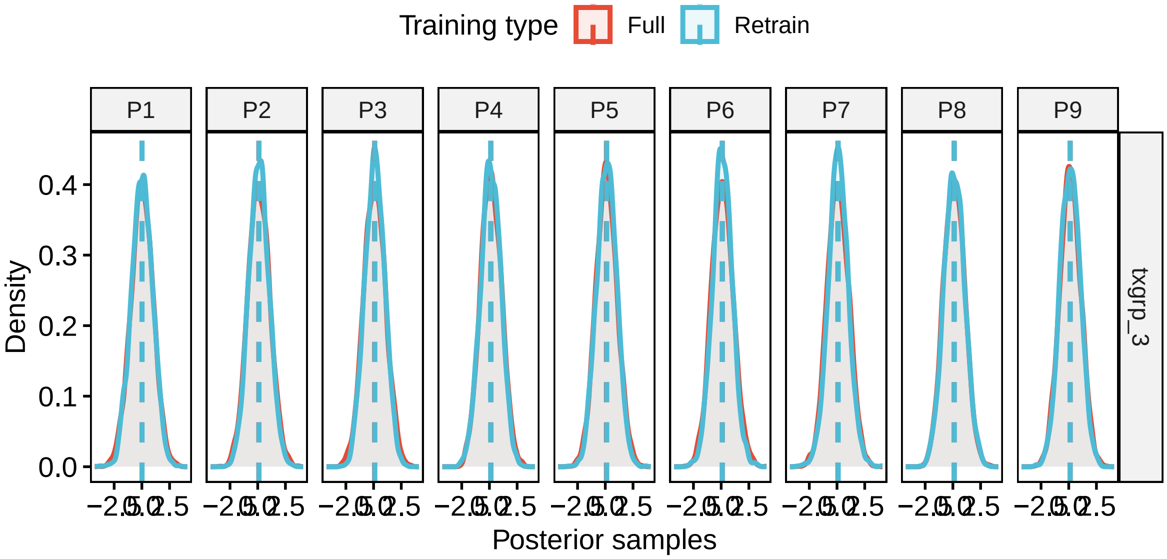
Training type
Full
Retrain
P1
P2
P3
P4
P5
P6
P7
P8
P9
txgrp_3
Density
Posterior samples

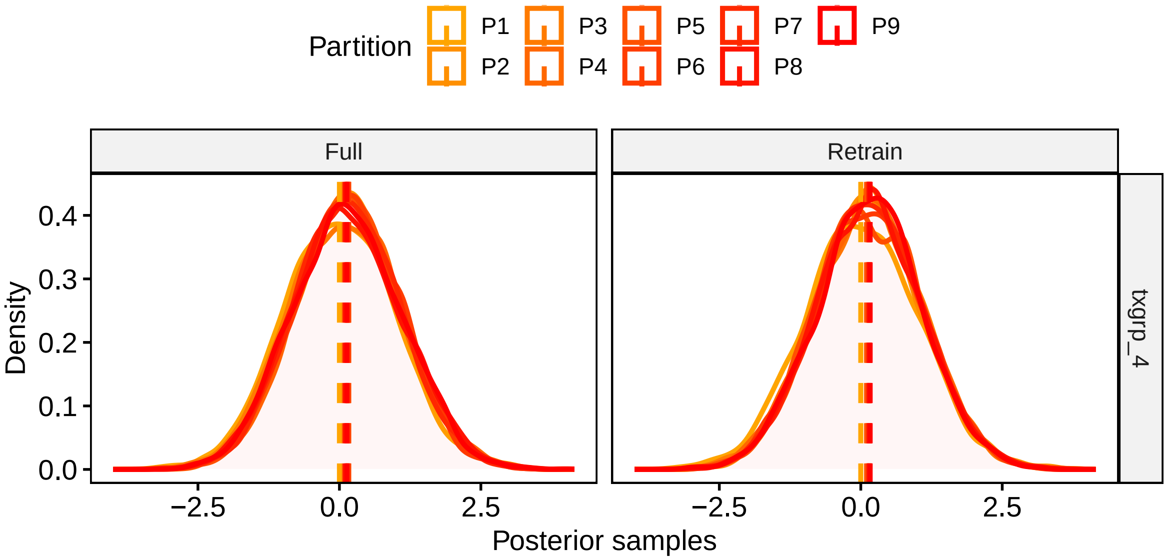
Partition
P1
P2
P3
P4
P5
P6
P7
P8
P9
Full
Retrain
txgrp_4
Density
Posterior samples

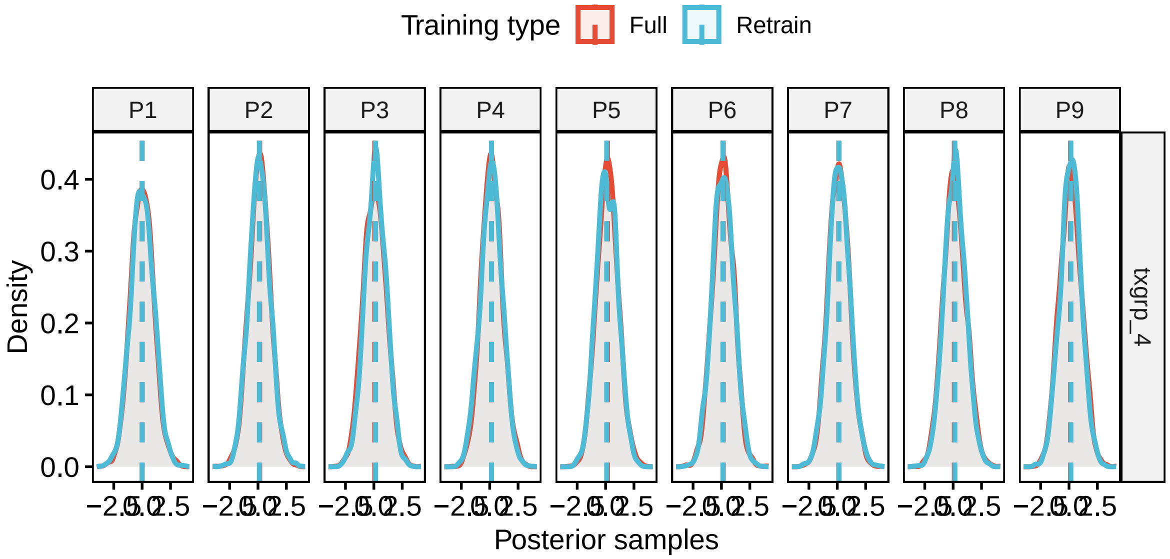
Training type
Full
Retrain
P1
P2
P3
P4
P5
P6
P7
P8
P9
Density
0.4
0.3
0.2
0.1
0.0
txgrp_4
Posterior samples

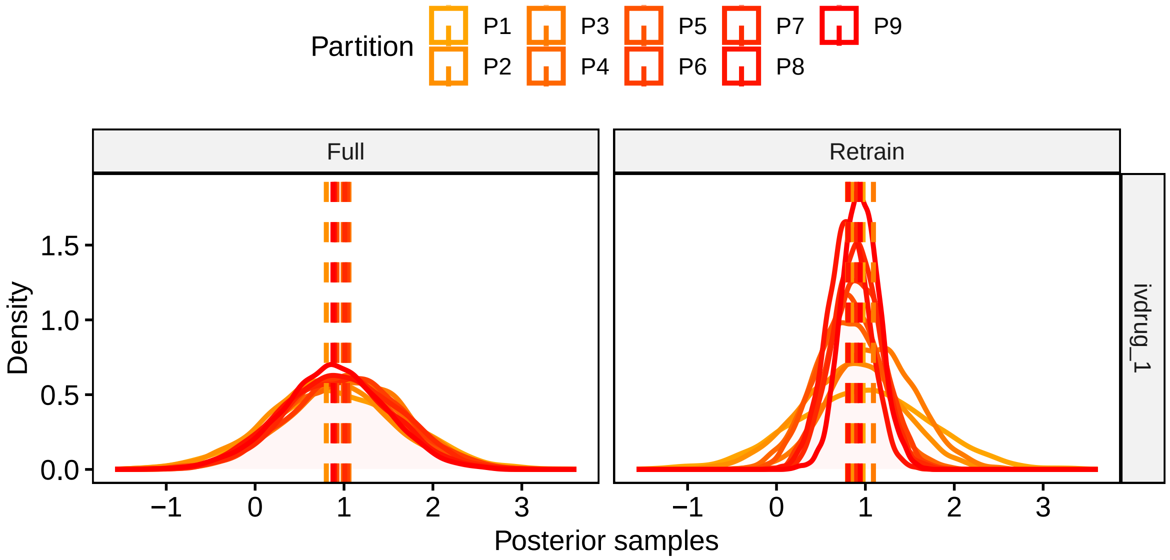
Partition
P1
P2
P3
P4
P5
P6
P7
P8
P9
Full
Retrain
Density
1.5
1.0
0.5
0.0
−1
0
1
2
3
ivdrug_1
Posterior samples

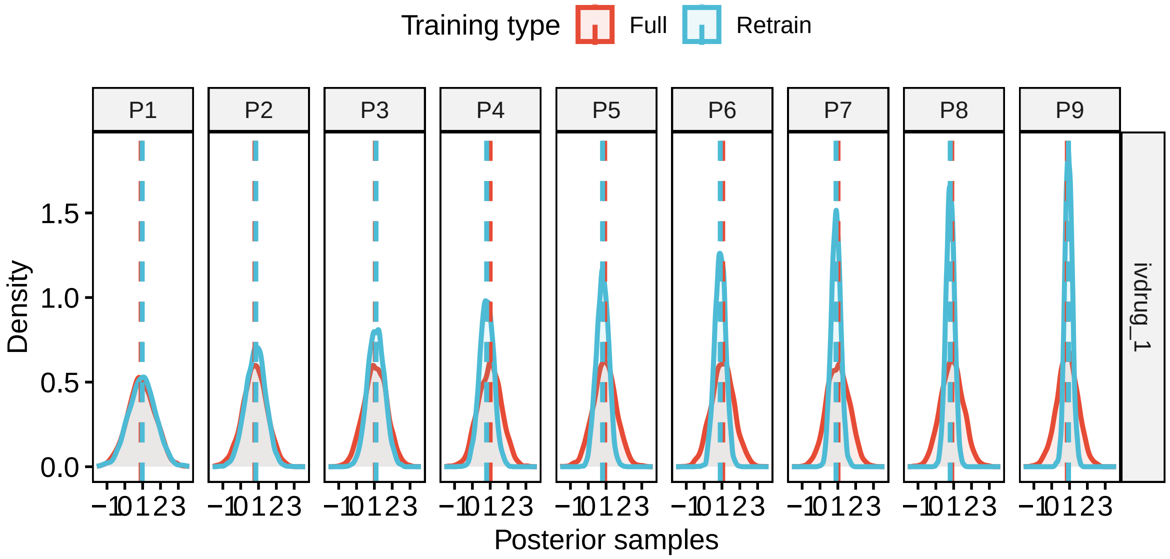
Training type
Full
Retrain
P1
P2
P3
P4
P5
P6
P7
P8
P9
ivdrug_1
Density
Posterior samples

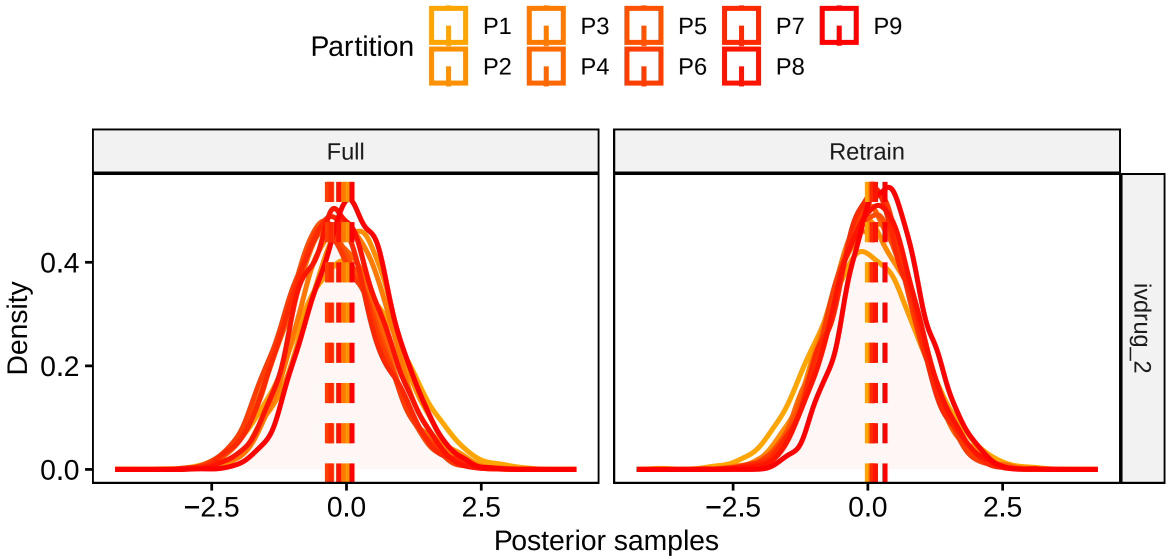
Partition
P1
P2
P3
P4
P5
P6
P7
P8
P9
Full
Retrain
ivdrug_2
Density
Posterior samples

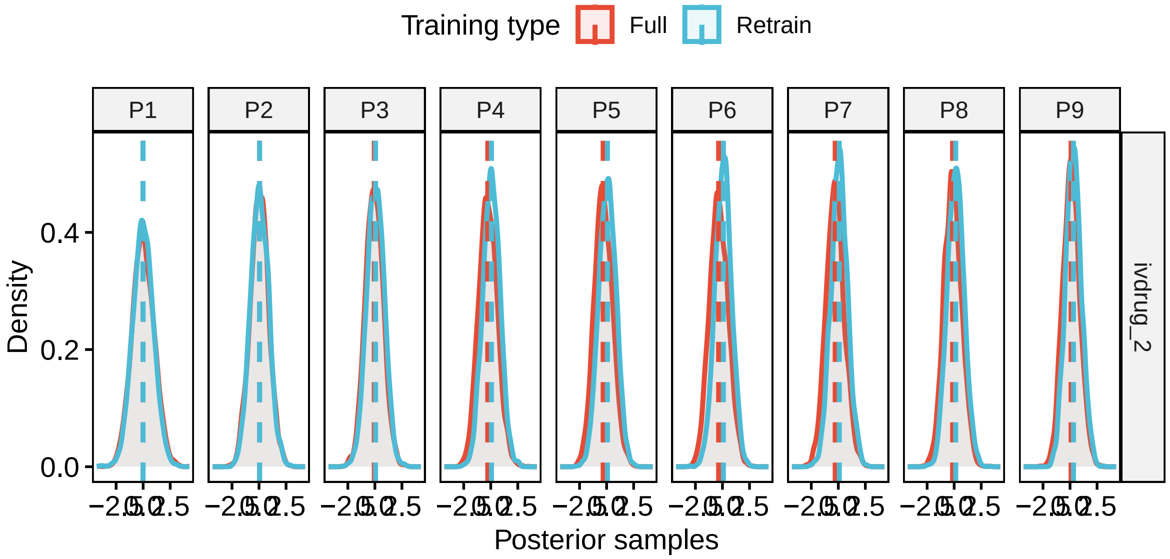
Training type
Full
Retrain
P1
P2
P3
P4
P5
P6
P7
P8
P9
ivdrug_2
Density
0.4
0.2
0.0
Posterior samples

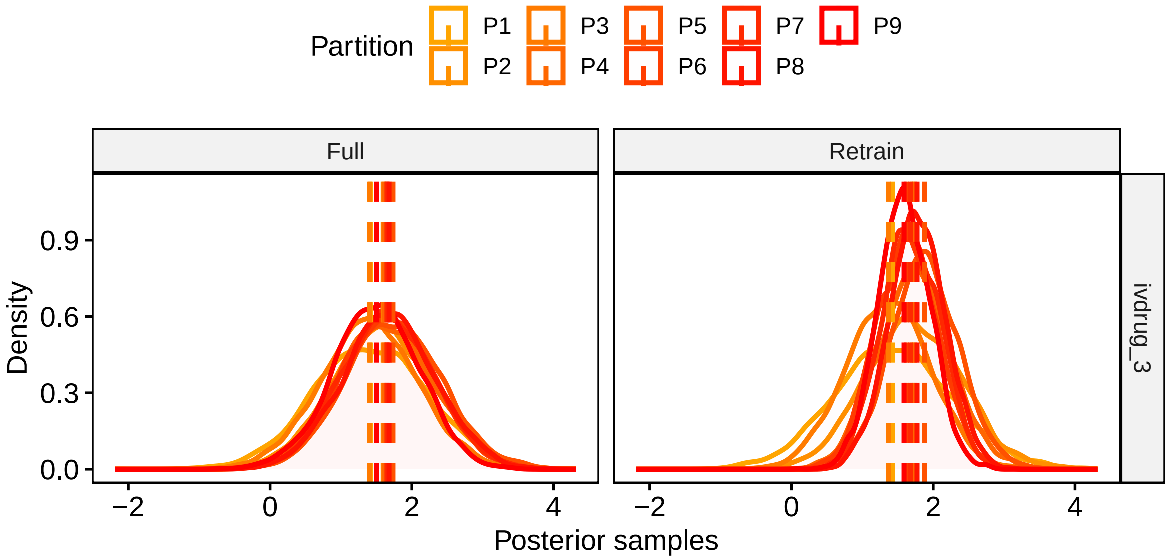
Partition
P1
P2
P3
P4
P5
P6
P7
P8
P9
Full
Retrain
ivdrug_3
Density
0.9
0.6
0.3
0.0
−2
0
2
4
Posterior samples

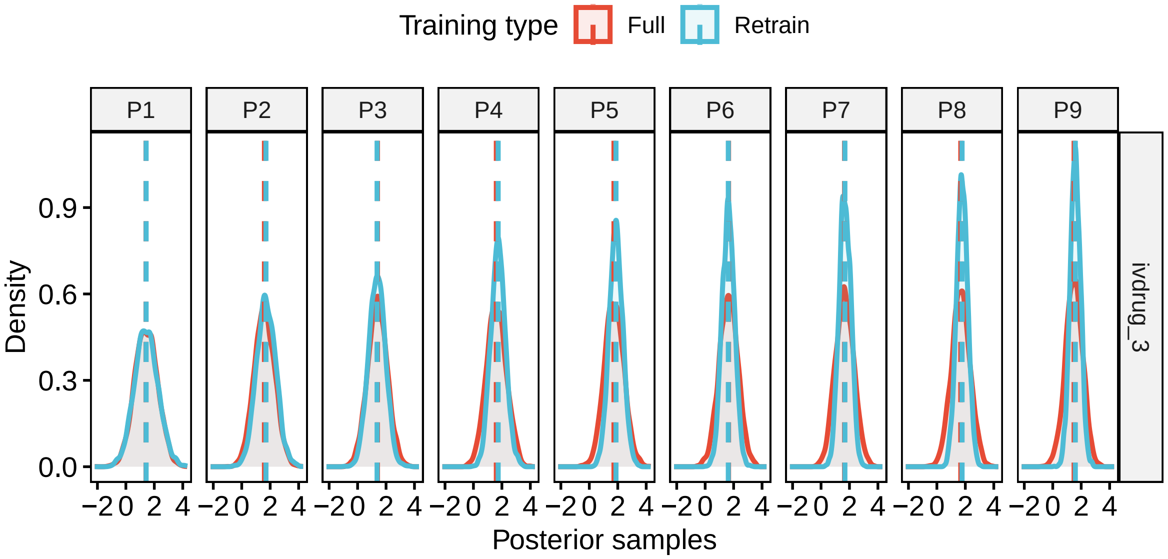
Training type
Full
Retrain
P1
P2
P3
P4
P5
P6
P7
P8
P9
ivdrug_3
Density
0.0
0.3
0.6
0.9
−2 0 2 4
Posterior samples

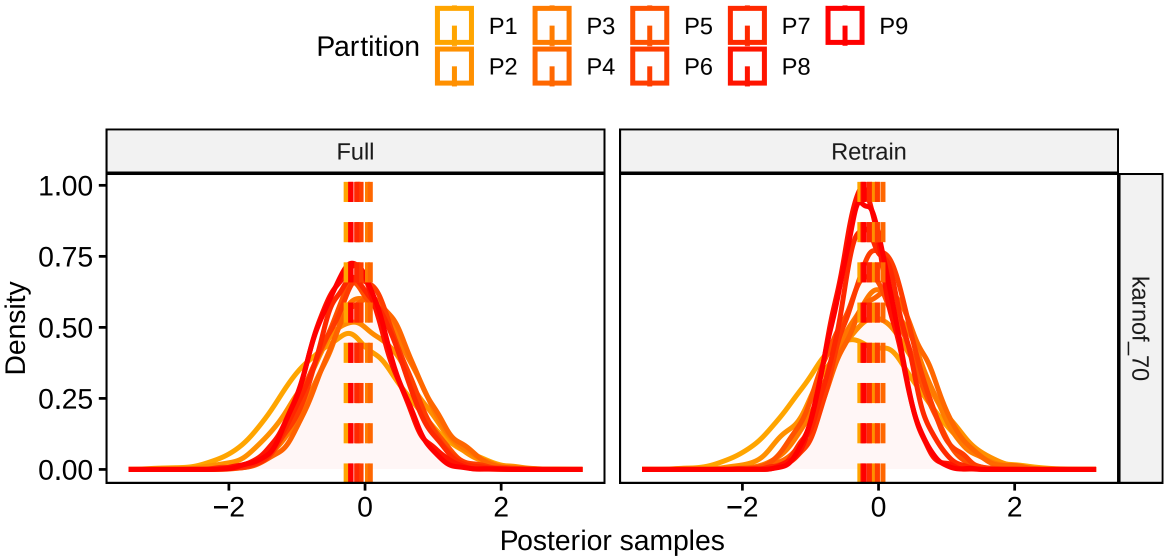
Partition
P1
P2
P3
P4
P5
P6
P7
P8
P9
Full
Retrain
karnof_70
Density
0.00
0.25
0.50
0.75
1.00
−2
0
2
Posterior samples

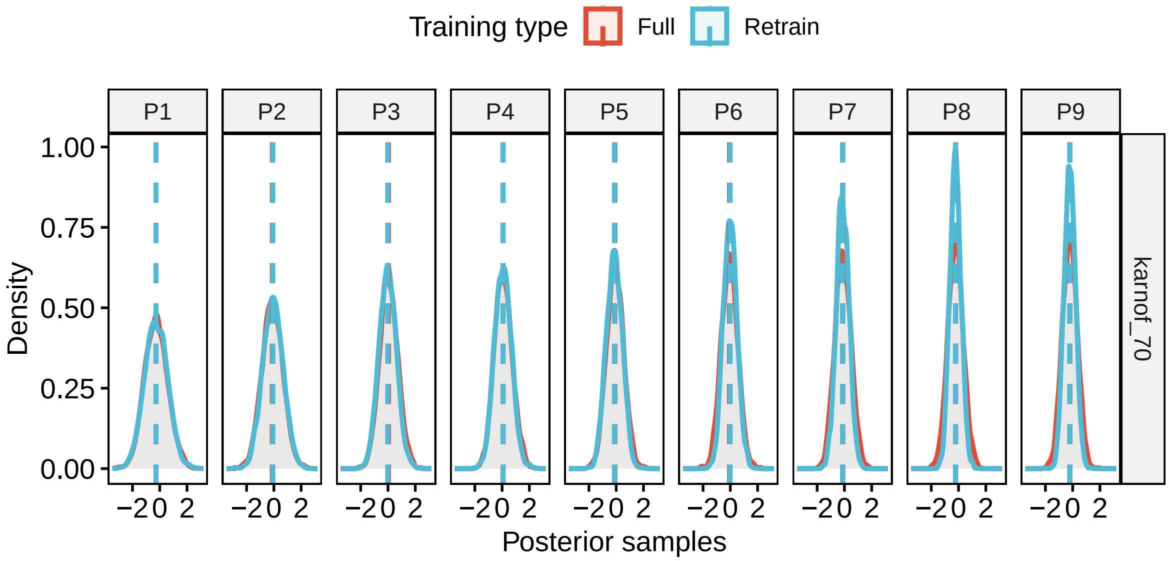
Training type
Full
Retrain
P1
P2
P3
P4
P5
P6
P7
P8
P9
karnof_70
Density
1.00
0.75
0.50
0.25
0.00
−20 2
Posterior samples

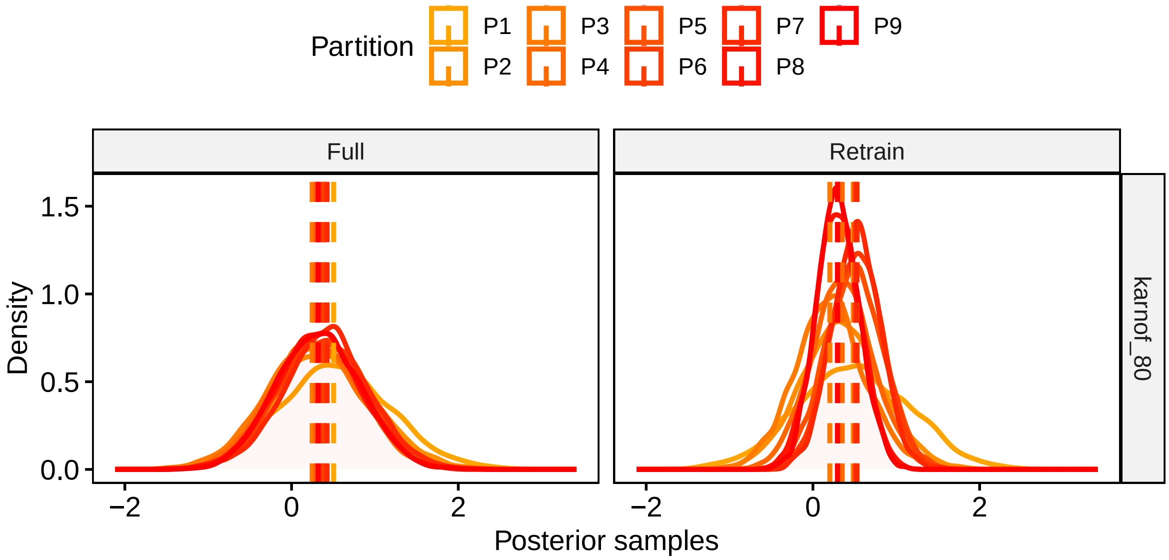
Partition
P1
P2
P3
P4
P5
P6
P7
P8
P9
Full
Retrain
karnof_80
Density
1.5
1.0
0.5
0.0
−2
0
2
Posterior samples

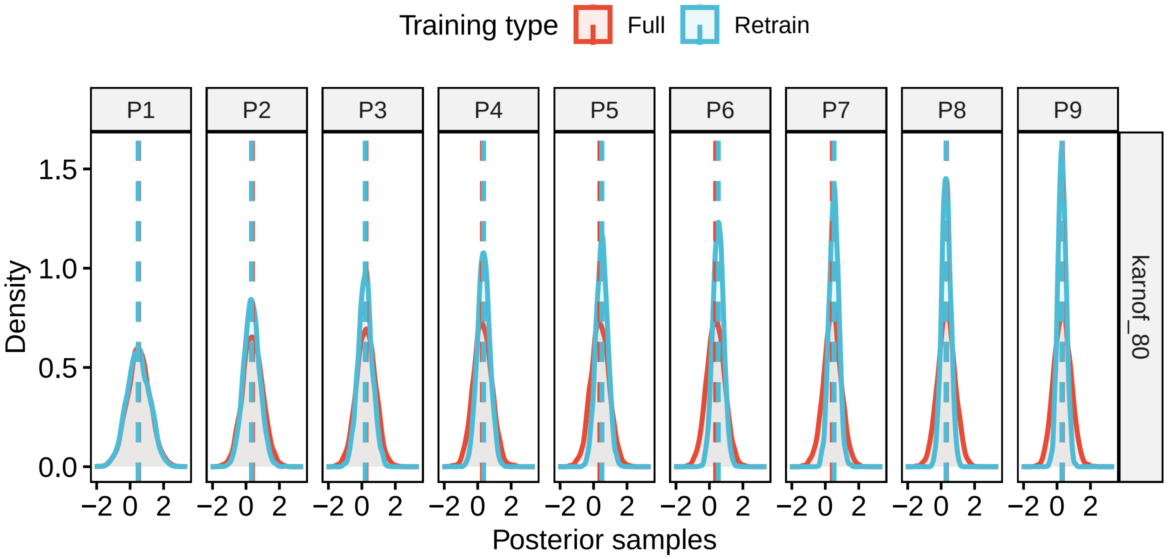
Training type
Full
Retrain
P1
P2
P3
P4
P5
P6
P7
P8
P9
karnof_80
Density
1.5
1.0
0.5
0.0
−2 0 2
Posterior samples

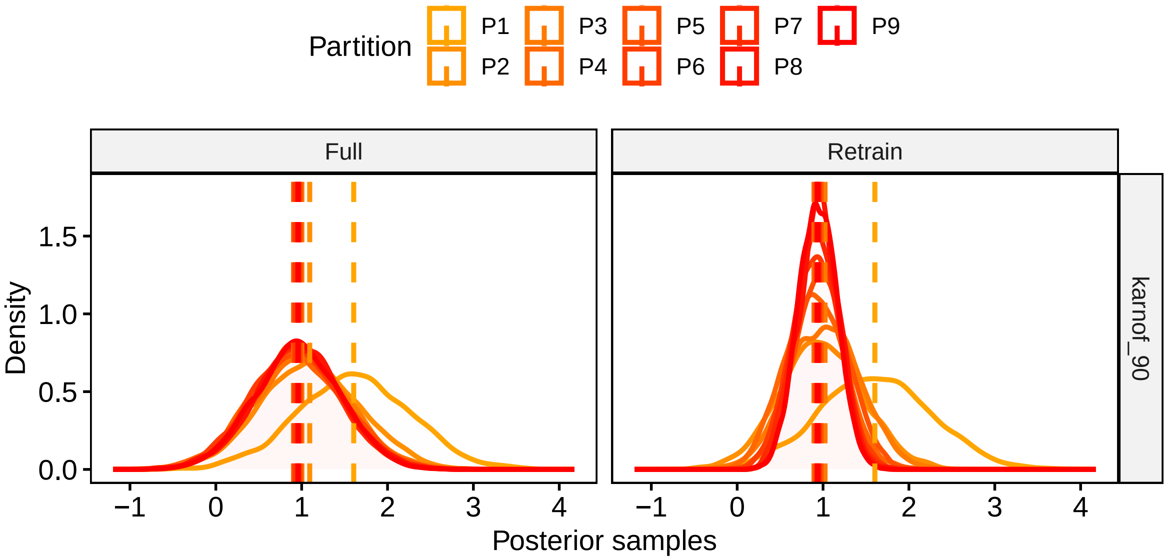
Partition
P1
P2
P3
P4
P5
P6
P7
P8
P9
Full
Retrain
karnof_90
Density
1.5
1.0
0.5
0.0
−1
0
1
2
3
4
Posterior samples

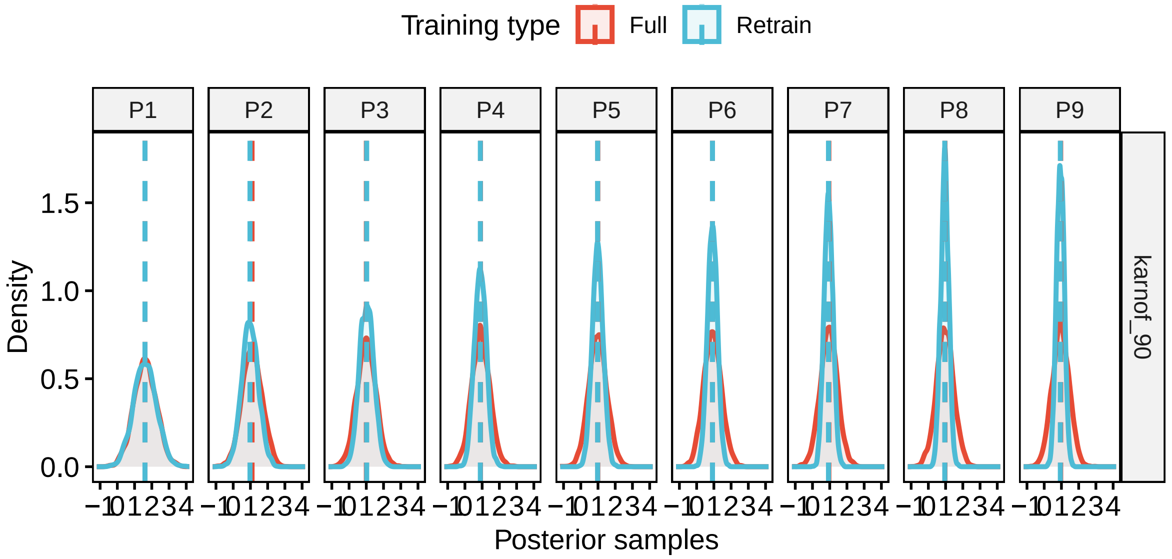
Training type
Full
Retrain
P1
P2
P3
P4
P5
P6
P7
P8
P9
karnof_90
Density
Posterior samples

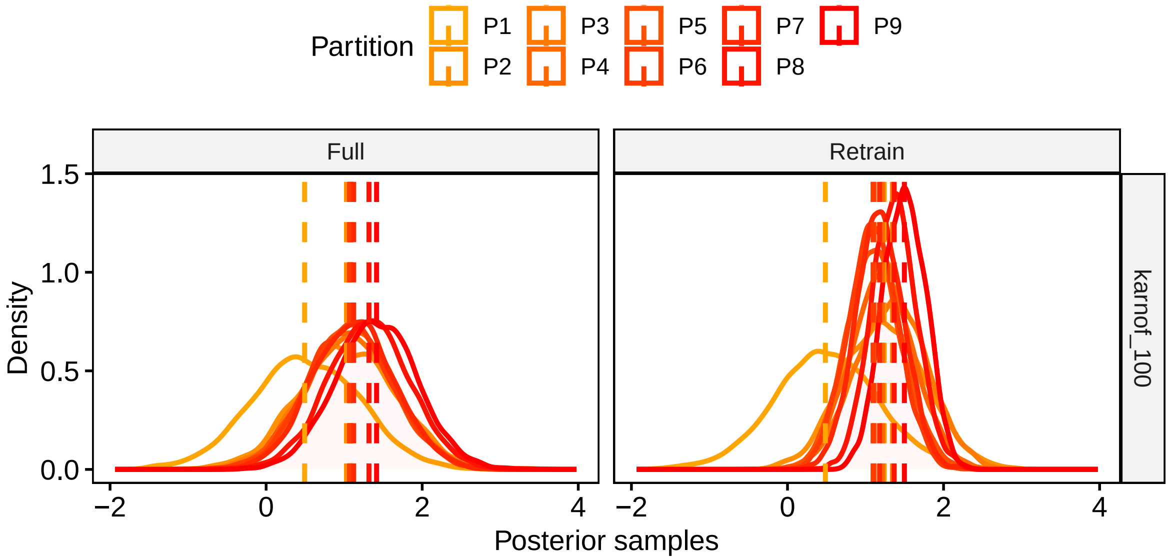
Partition
P1
P2
P3
P4
P5
P6
P7
P8
P9
Full
Retrain
karnof_100
Density
Posterior samples

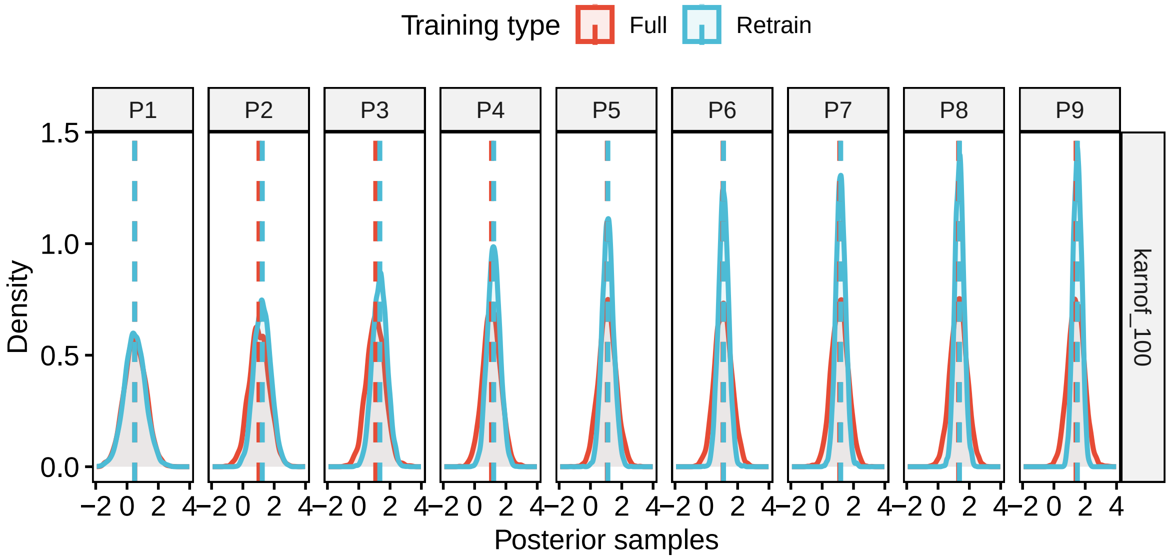

Training type
Full
Retrain
P1
P2
P3
P4
P5
P6
P7
P8
P9
karnof_100
Density
Posterior samples

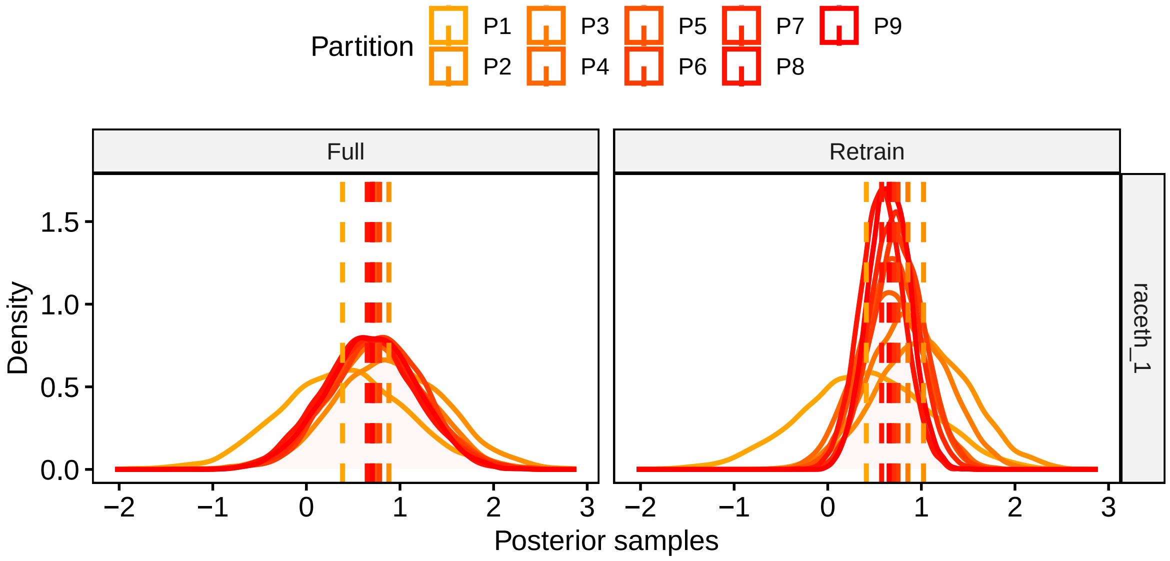

Partition
P1
P2
P3
P4
P5
P6
P7
P8
P9
Full
Retrain
raceth_1
Density
Posterior samples

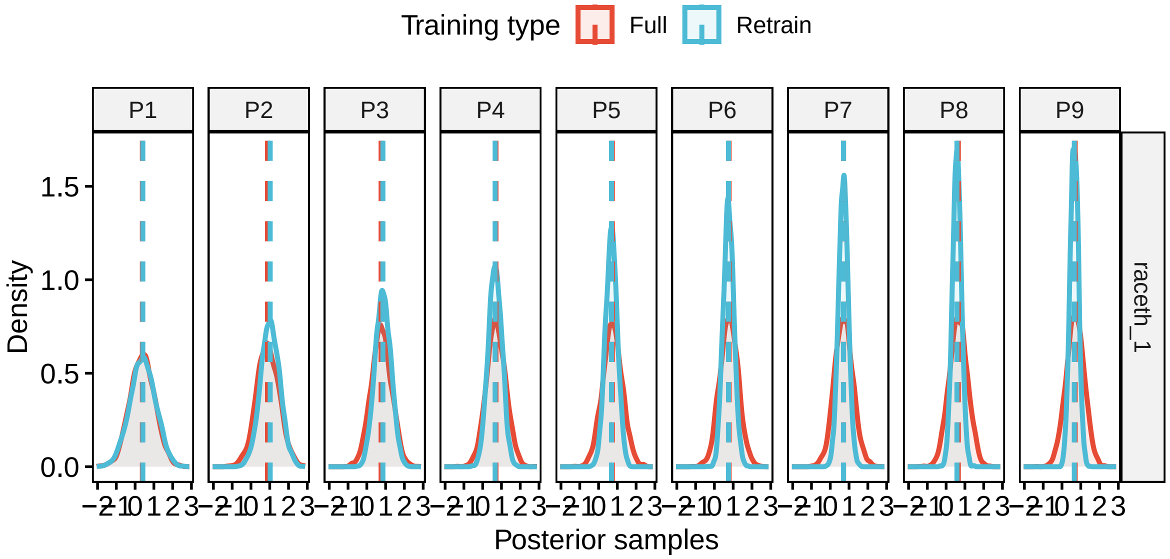
Training type
Full
Retrain
P1
P2
P3
P4
P5
P6
P7
P8
P9
raceth_1
Density
Posterior samples

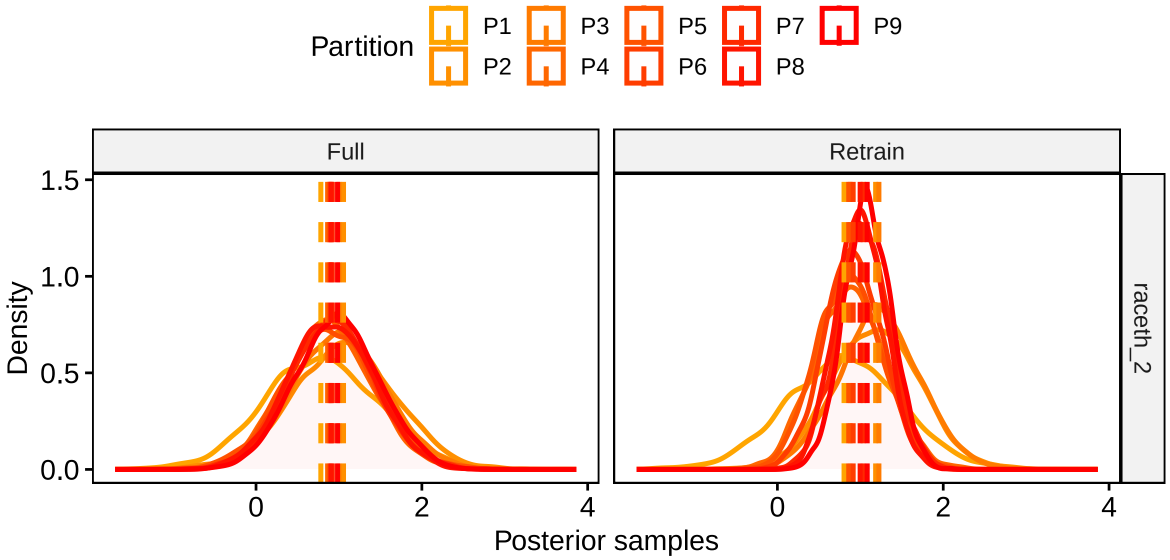
Partition
P1
P2
P3
P4
P5
P6
P7
P8
P9
Full
Retrain
raceth_2
Density
Posterior samples

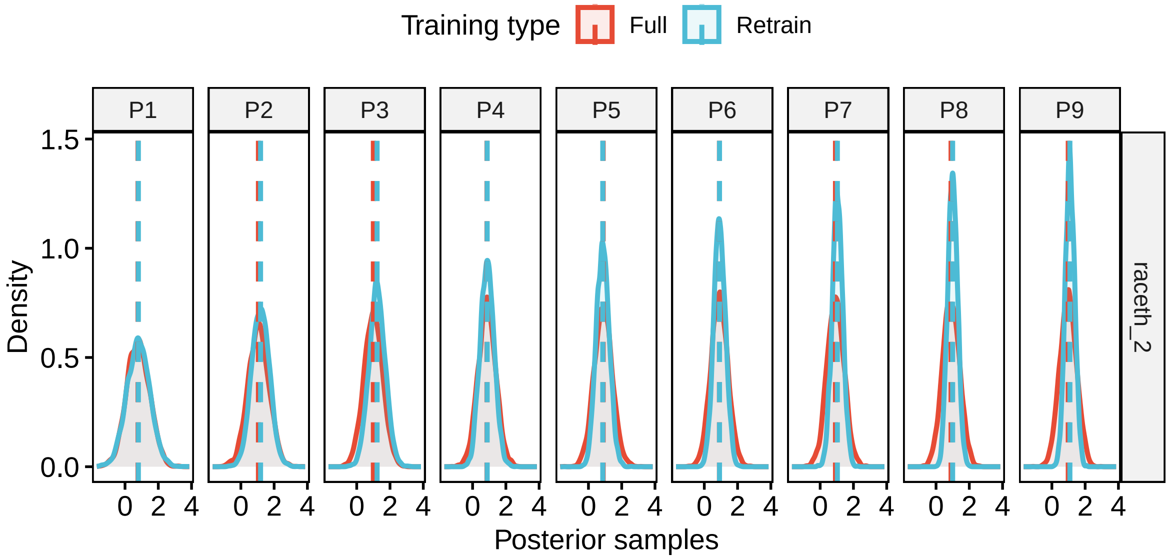
Training type
Full
Retrain
P1
P2
P3
P4
P5
P6
P7
P8
P9
raceth_2
Density
1.5
1.0
0.5
0.0
0 2 4
Posterior samples

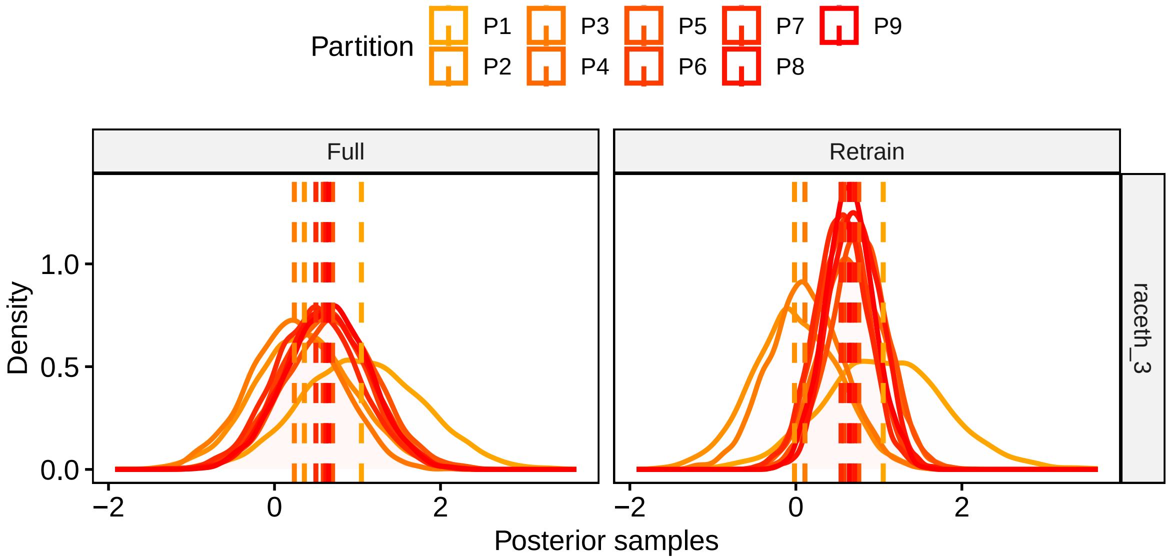
Partition
P1
P2
P3
P4
P5
P6
P7
P8
P9
Full
Retrain
raceth_3
Density
1.0
0.5
0.0
−2
0
2
Posterior samples

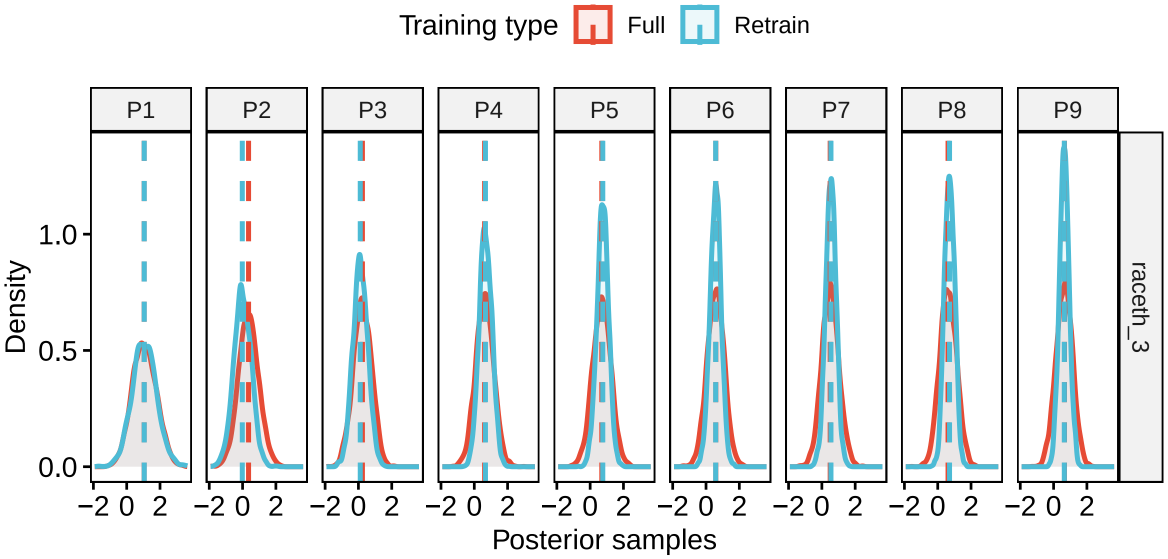
Training type
Full
Retrain
P1
P2
P3
P4
P5
P6
P7
P8
P9
raceth_3
Density
1.0
0.5
0.0
−2 0 2
Posterior samples

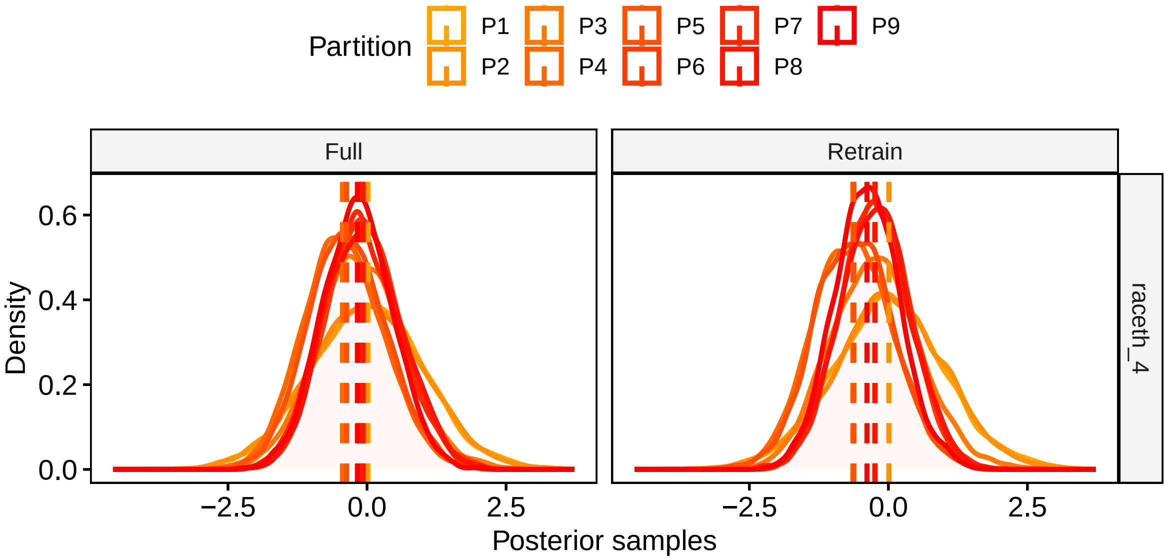
Partition
P1
P2
P3
P4
P5
P6
P7
P8
P9
Full
Retrain
raceth_4
Density
0.6
0.4
0.2
0.0
−2.5
0.0
2.5
Posterior samples

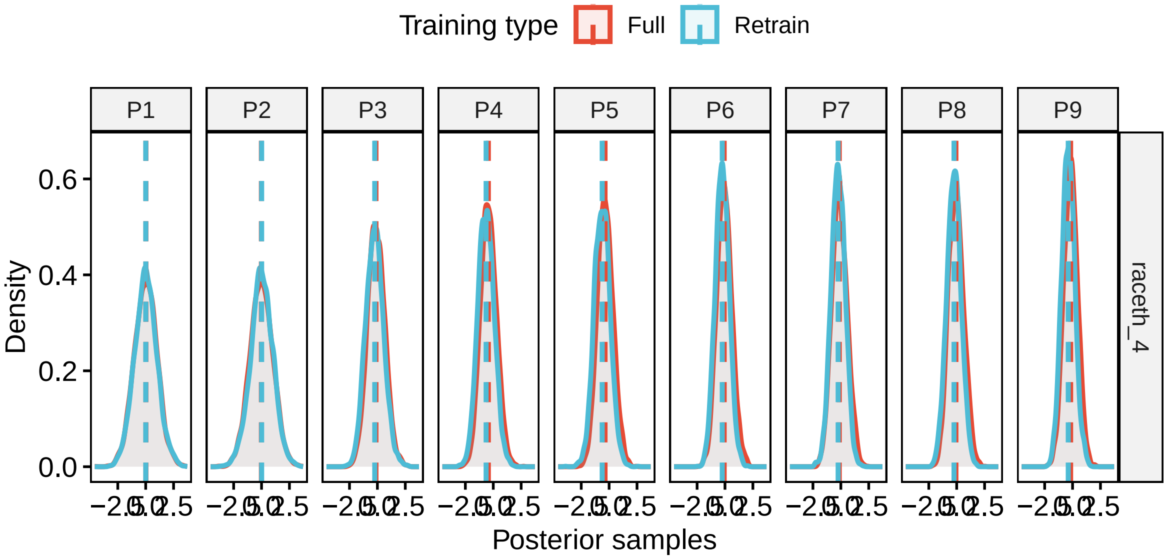
Training type
Full
Retrain
P1
P2
P3
P4
P5
P6
P7
P8
P9
raceth_4
Density
0.6
0.4
0.2
0.0
Posterior samples

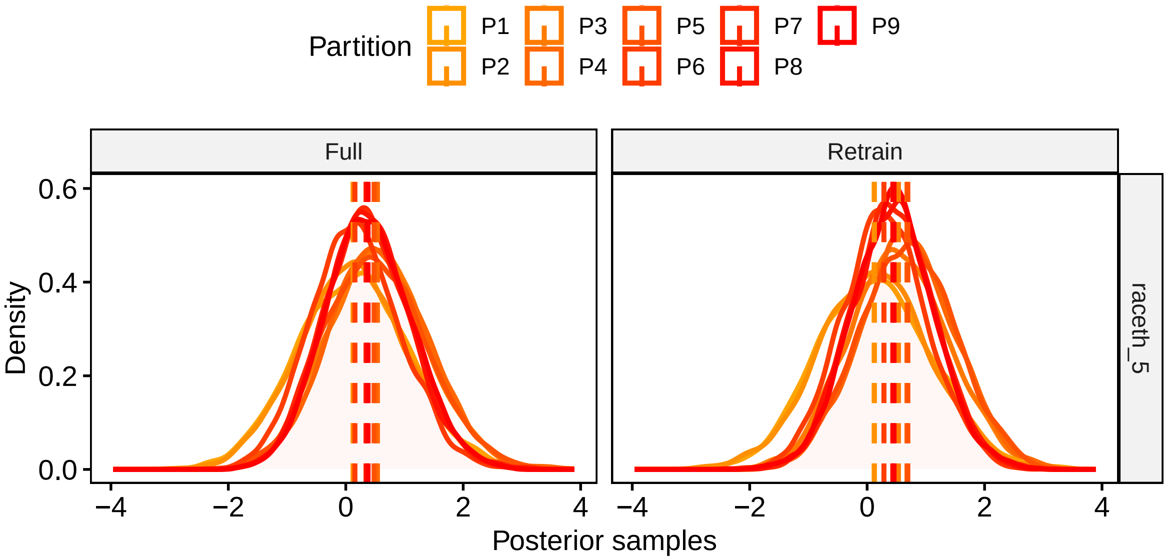
Partition
P1
P2
P3
P4
P5
P6
P7
P8
P9
Full
Retrain
raceth_5
Density
0.6
0.4
0.2
0.0
−4
−2
0
2
4
Posterior samples

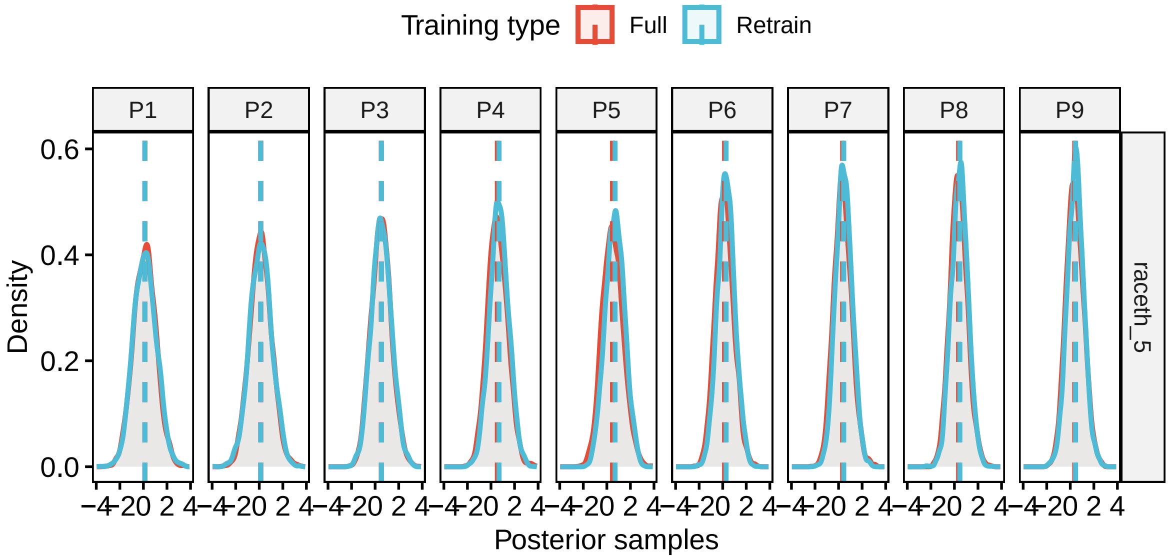

Weibull

WHAS

```
whas_vars <- data %>% filter(experiment == 'whas') %>% select(var) %>% unique(.)

for(var_idx in unique(whas_vars$var)){
  p1 <- plot_density(data, 'whas', 'pm_wb', var_idx)
  plot(p1)

  p <- plot_density_2(data, 'whas', 'pm_wb', var_idx)
  plot(p)
}
```

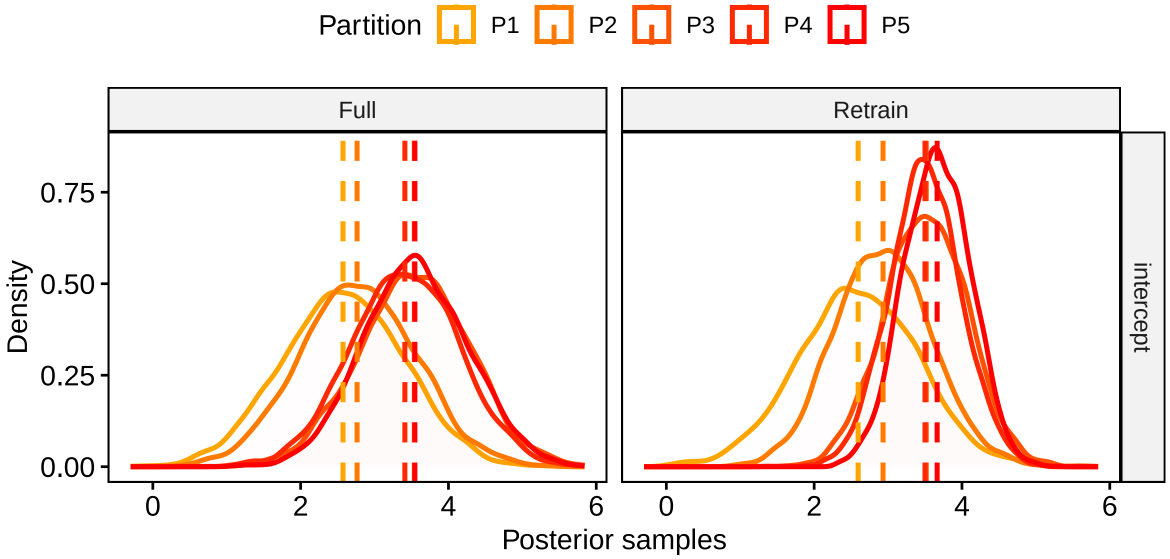
Partition
P1
P2
P3
P4
P5
Full
Retrain
intercept
Density
0.75
0.50
0.25
0.00
0
2
4
6
Posterior samples

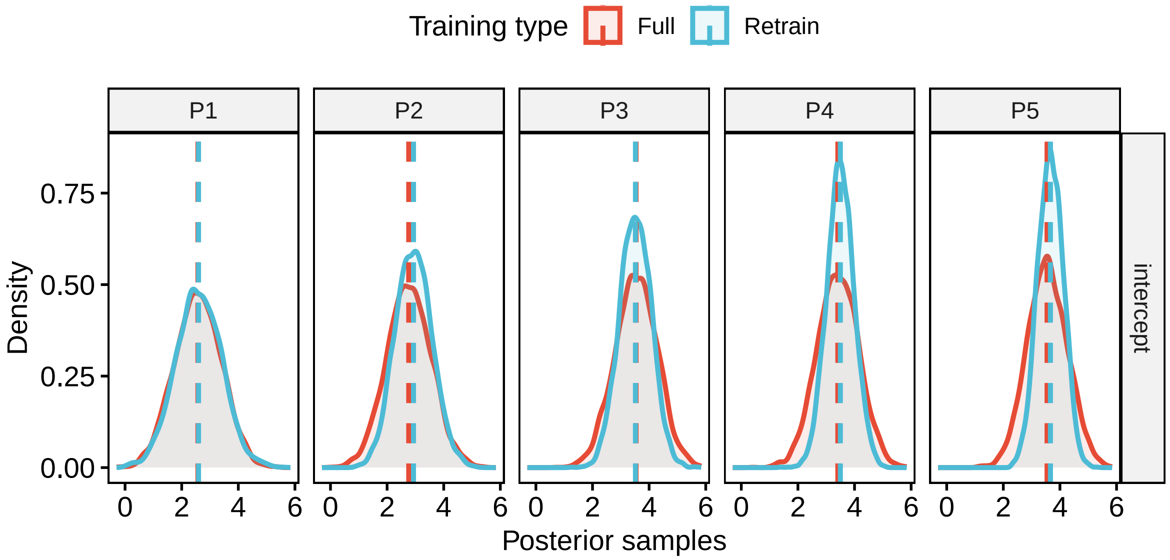
Training type
Full
Retrain
P1
P2
P3
P4
P5
intercept
Density
0.75
0.50
0.25
0.00
0
2
4
6
Posterior samples

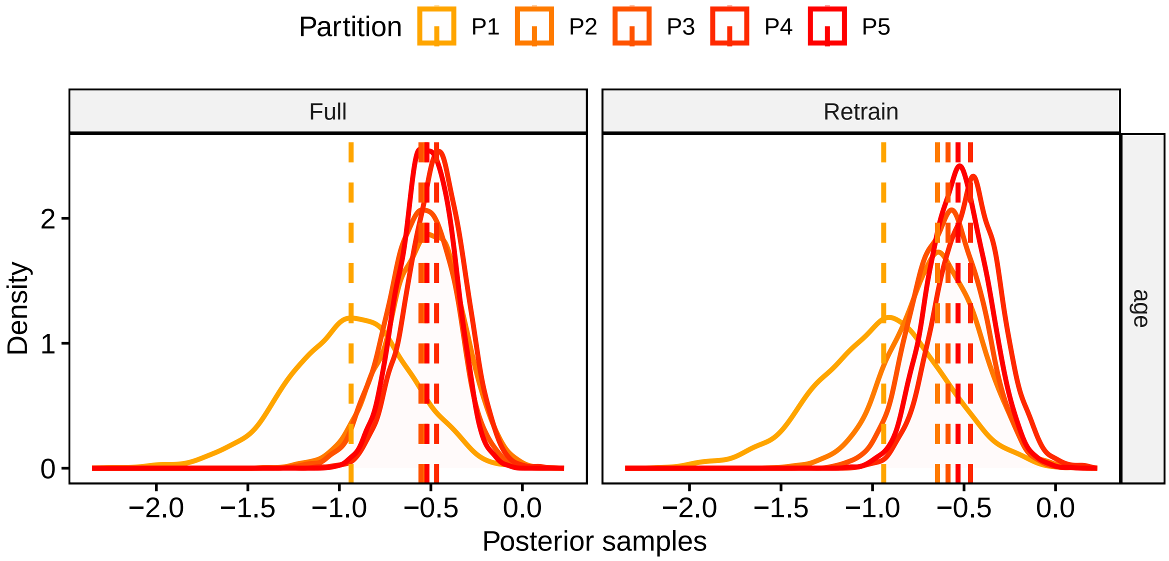
Partition
P1
P2
P3
P4
P5
Full
Retrain
Density
2
1
0
−2.0
−1.5
−1.0
−0.5
0.0
Posterior samples
age

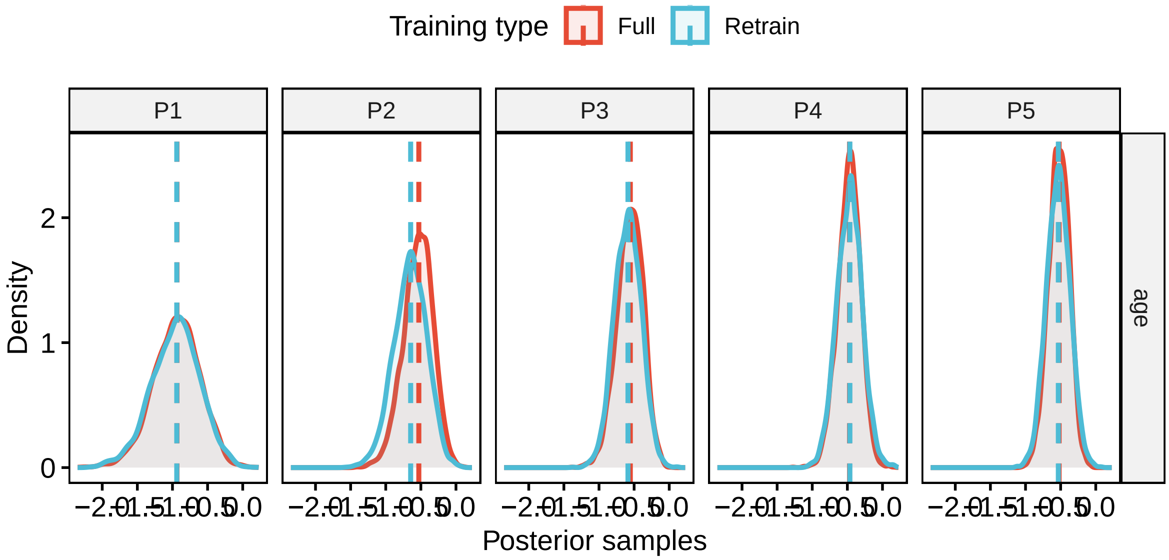
Training type
Full
Retrain
P1
P2
P3
P4
P5
Density
2
1
0
Posterior samples
age

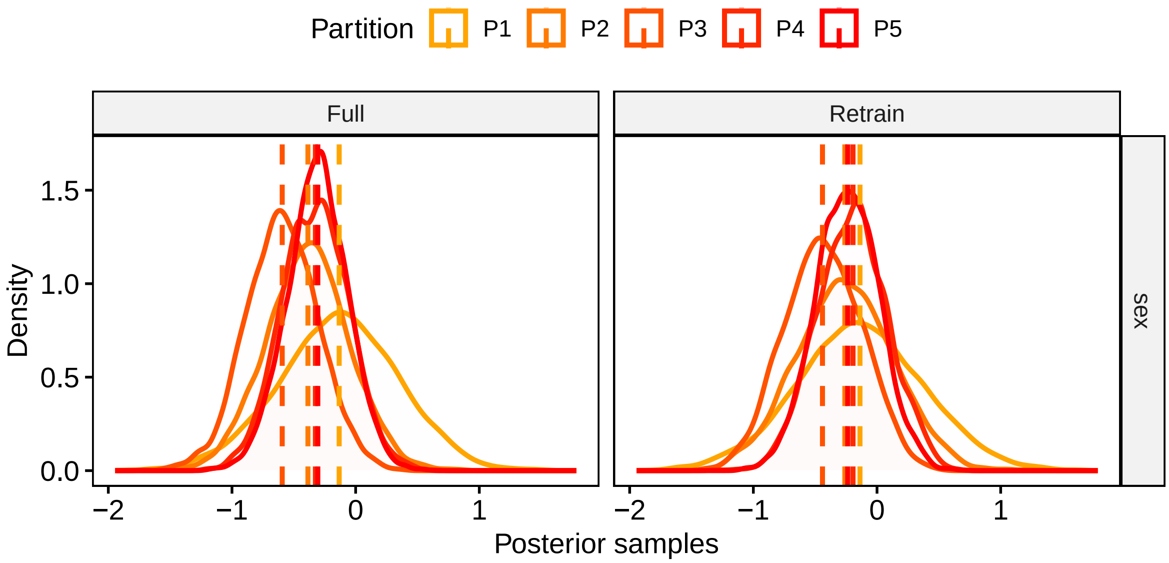
Partition
P1
P2
P3
P4
P5
Full
Retrain
sex
Density
1.5
1.0
0.5
0.0
−2
−1
0
1
Posterior samples

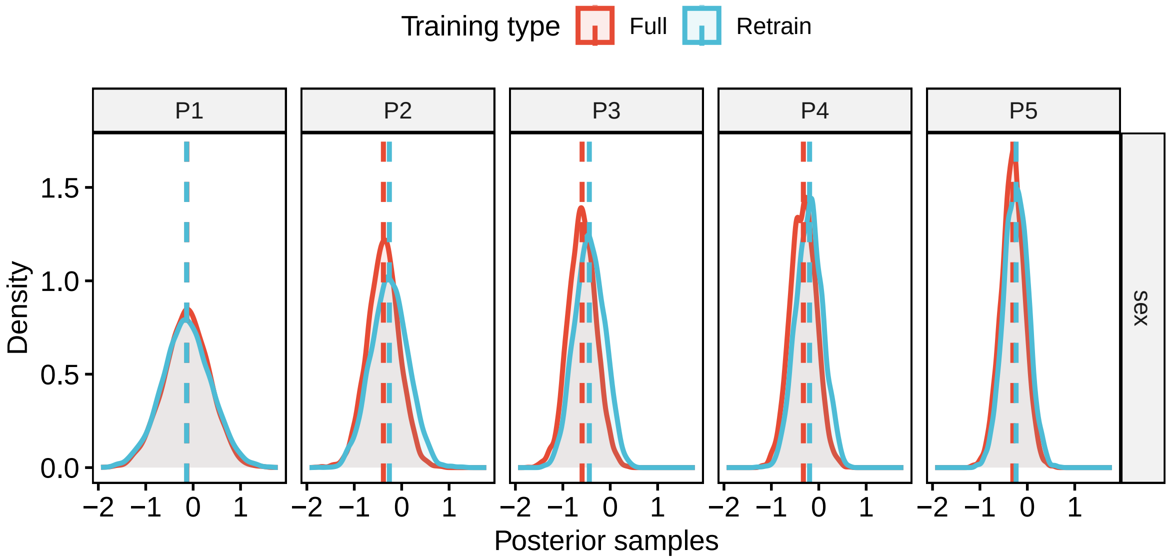
Training type
Full
Retrain
P1
P2
P3
P4
P5
sex
Density
1.5
1.0
0.5
0.0
−2
−1
0
1
Posterior samples

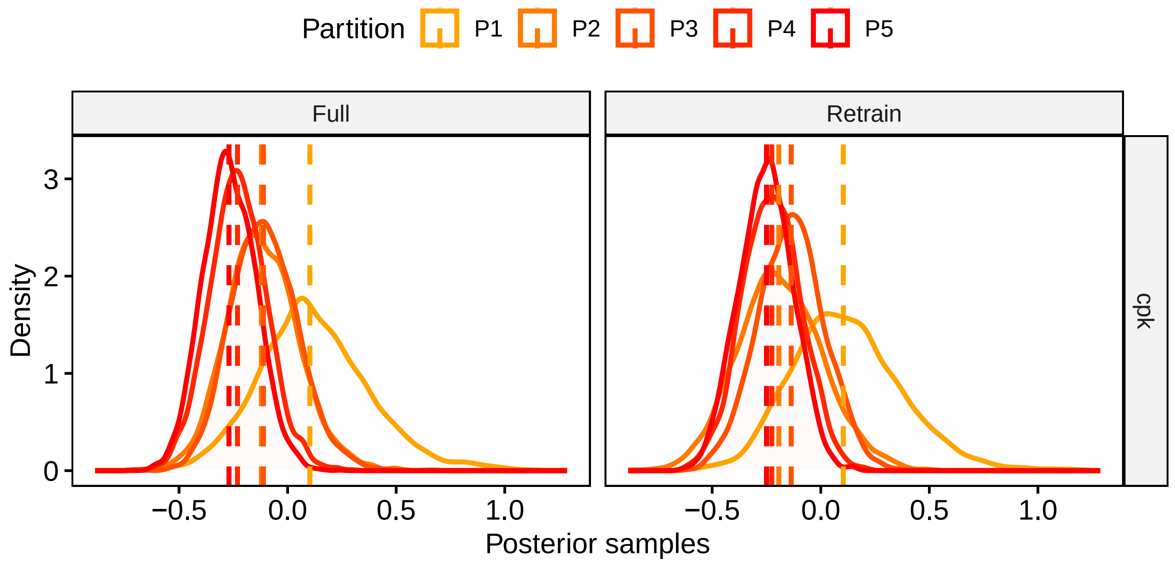
Partition
P1
P2
P3
P4
P5
Full
Retrain
cpk
Density
Posterior samples

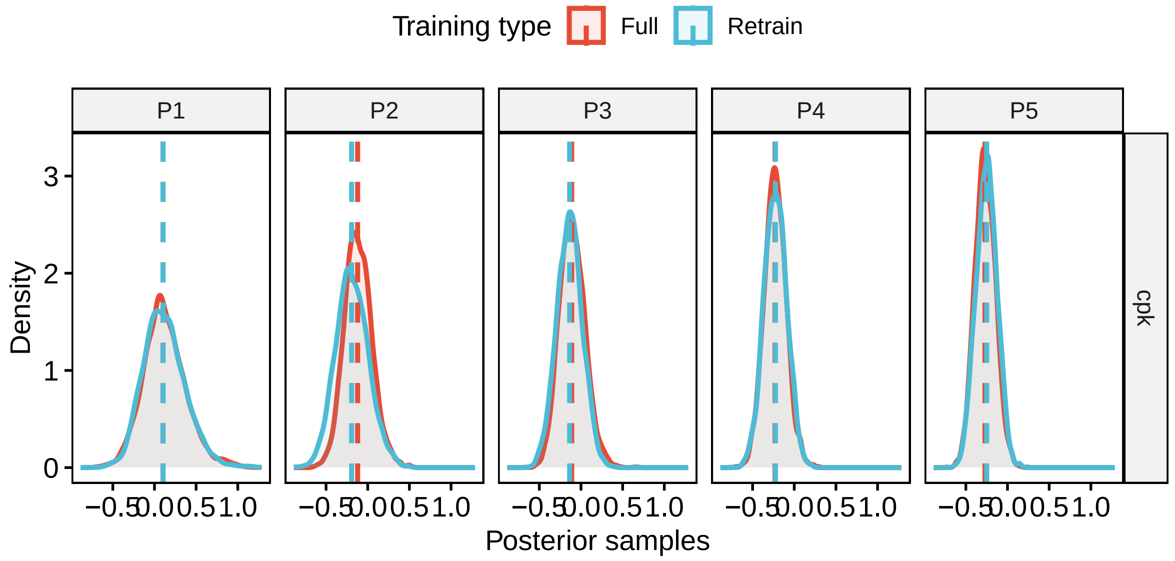
Training type
Full
Retrain
P1
P2
P3
P4
P5
cpk
Density
Posterior samples

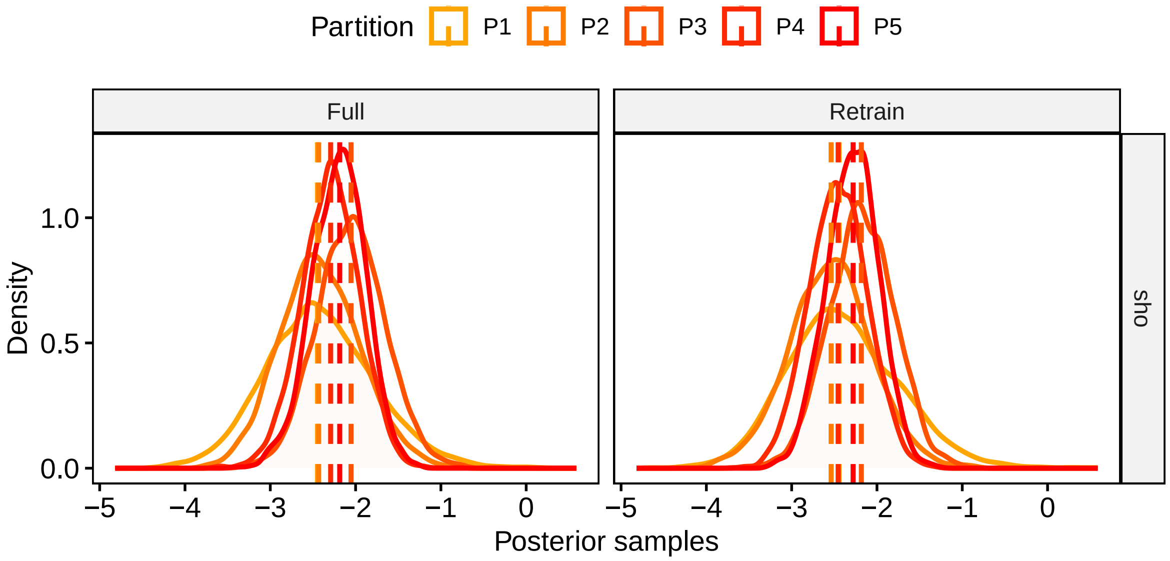

Partition P1 P2 P3 P4 P5
Full
Retrain
sho
Density
1.0
0.5
0.0
−5 −4 −3 −2 −1 0
Posterior samples

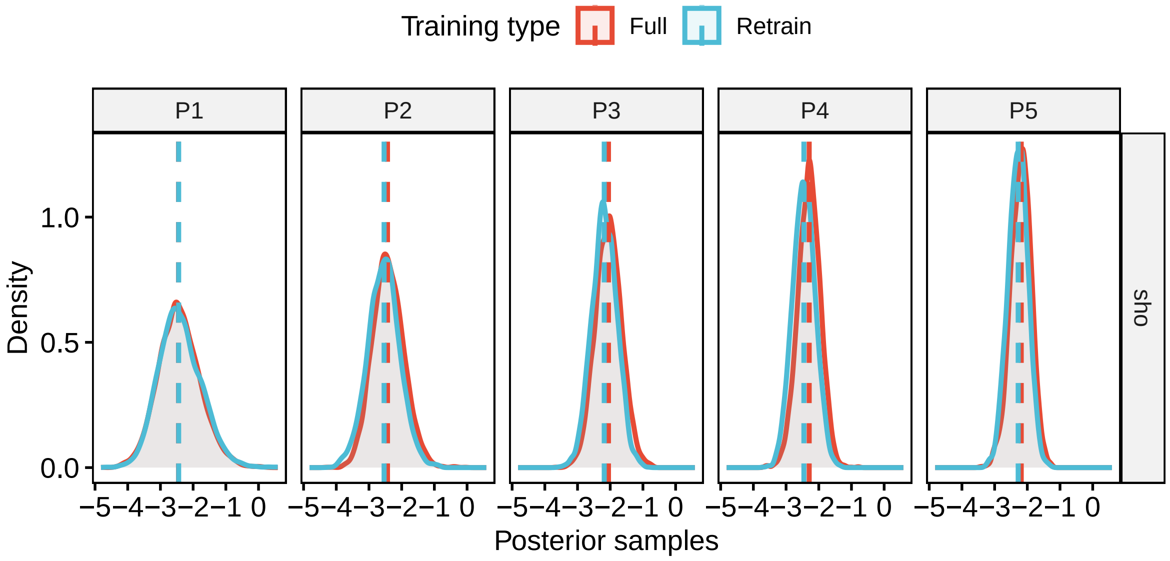

Training type Full Retrain
P1
P2
P3
P4
P5
sho
Density
1.0
0.5
0.0
−5−4−3−2−1 0
Posterior samples

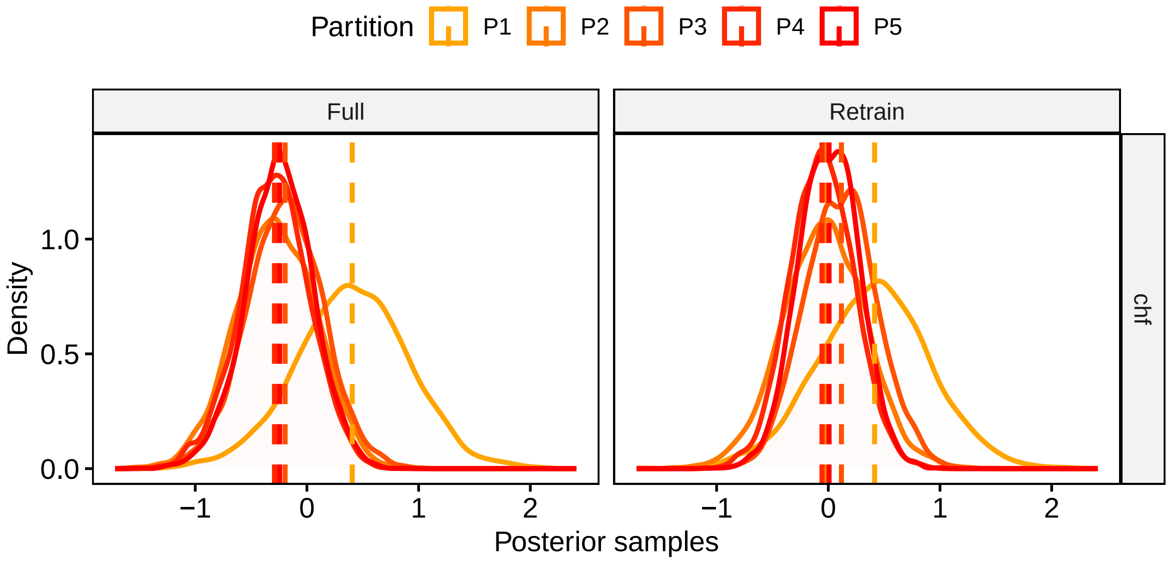
Partition
P1
P2
P3
P4
P5
Full
Retrain
chf
Density
1.0
0.5
0.0
−1
0
1
2
Posterior samples

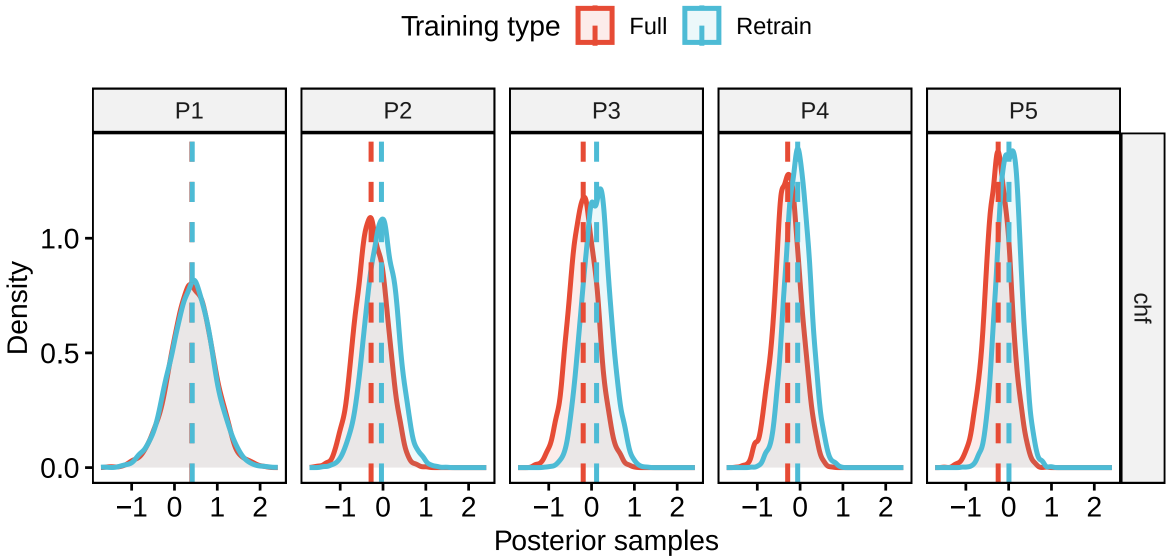
Training type
Full
Retrain
P1
P2
P3
P4
P5
chf
Density
1.0
0.5
0.0
−1
0
1
2
Posterior samples

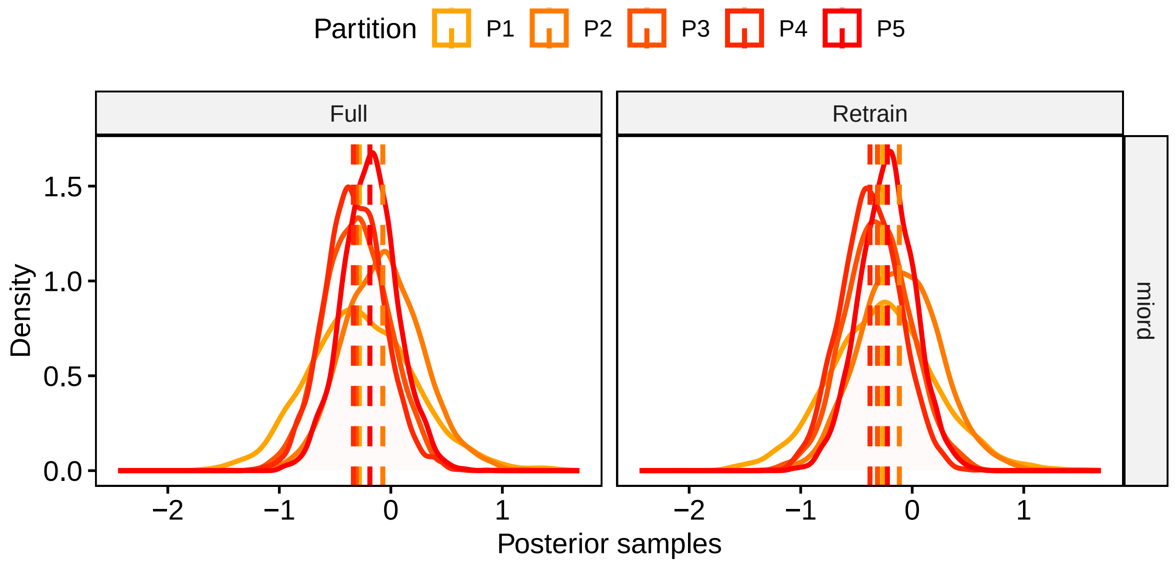
Partition
P1
P2
P3
P4
P5
Full
Retrain
miord
Density
Posterior samples

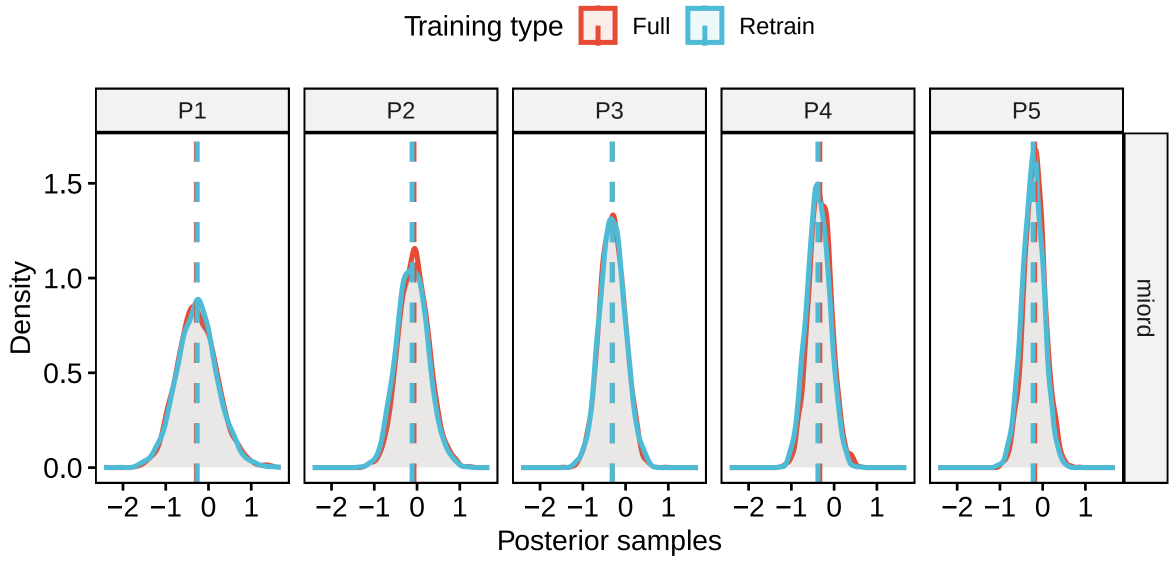
Training type
Full
Retrain
P1
P2
P3
P4
P5
miord
Density
Posterior samples

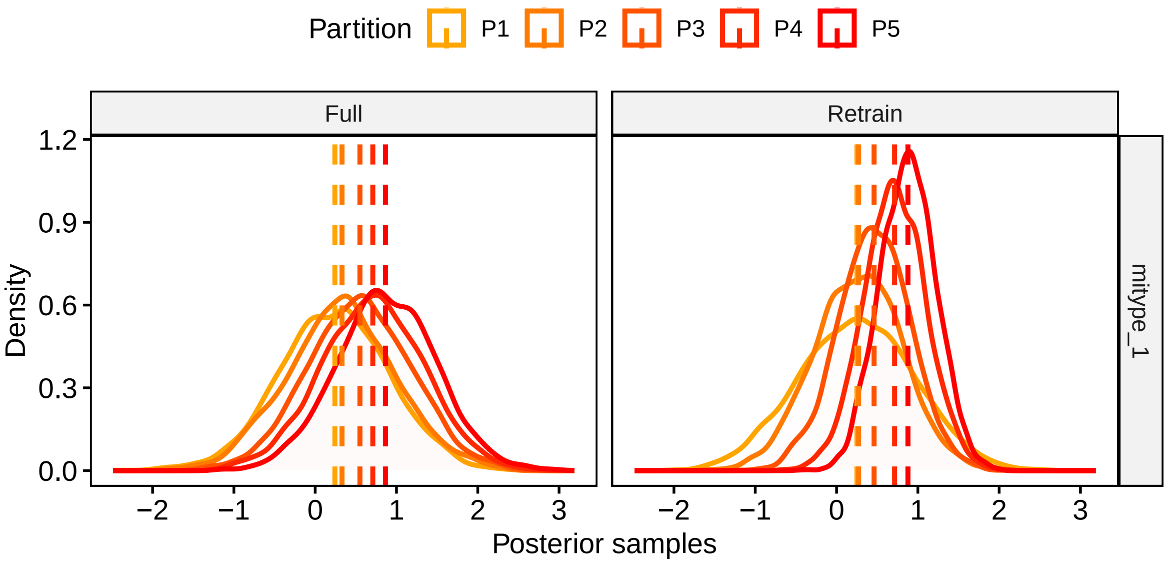
Partition
P1
P2
P3
P4
P5
Full
Retrain
mitype_1
Density
Posterior samples
1.2
0.9
0.6
0.3
0.0
−2
−1
0
1
2
3

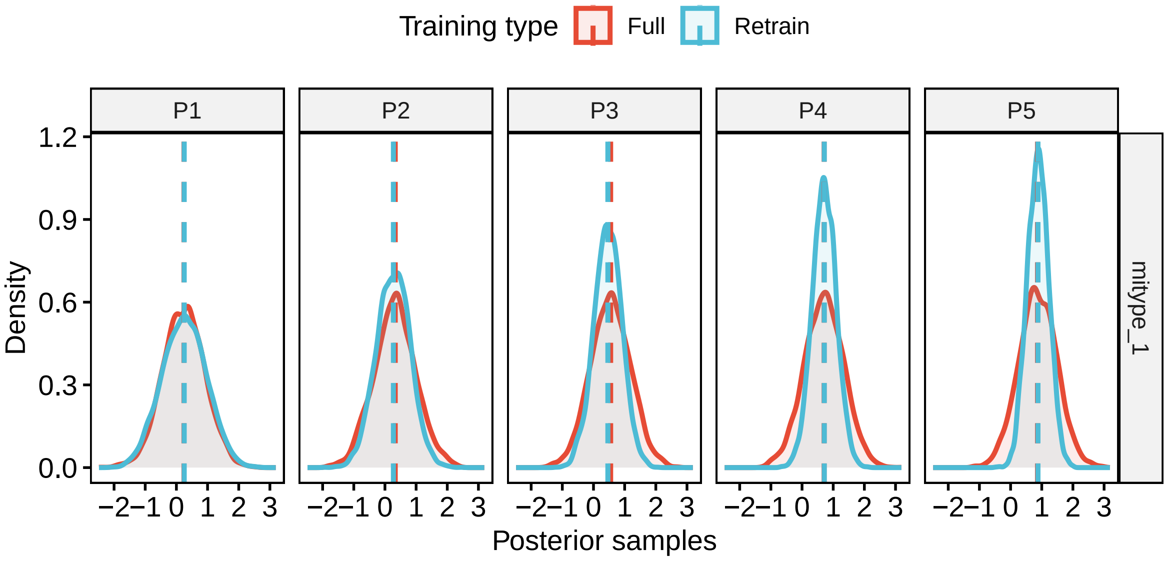
Training type
Full
Retrain
P1
P2
P3
P4
P5
mitype_1
Density
Posterior samples
1.2
0.9
0.6
0.3
0.0
−2−1 0 1 2 3

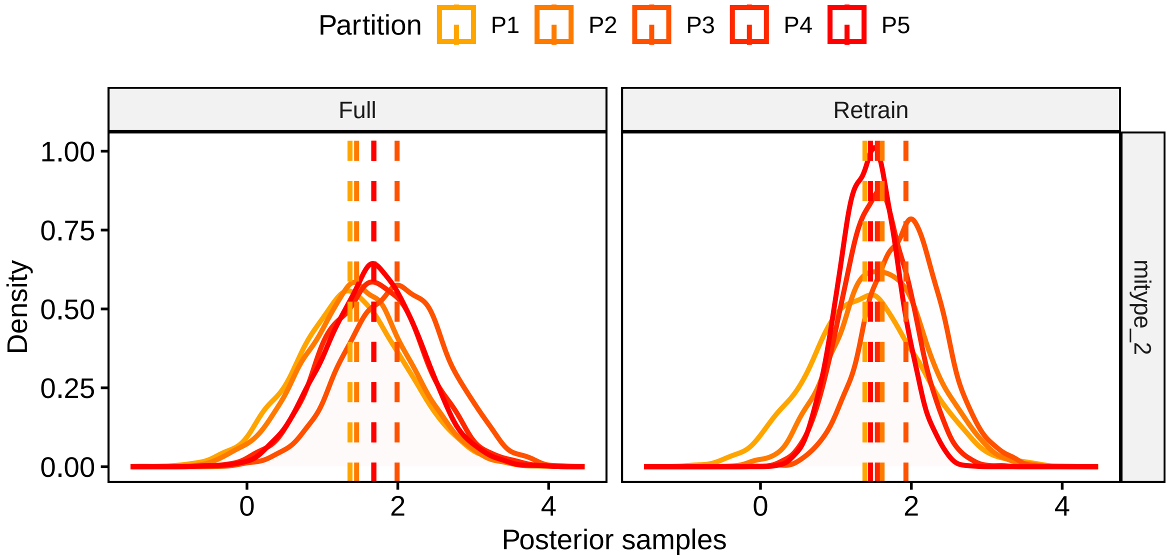
Partition P1 P2 P3 P4 P5
Full
Retrain
mitype_2
Density
Posterior samples
1.00
0.75
0.50
0.25
0.00
0
2
4

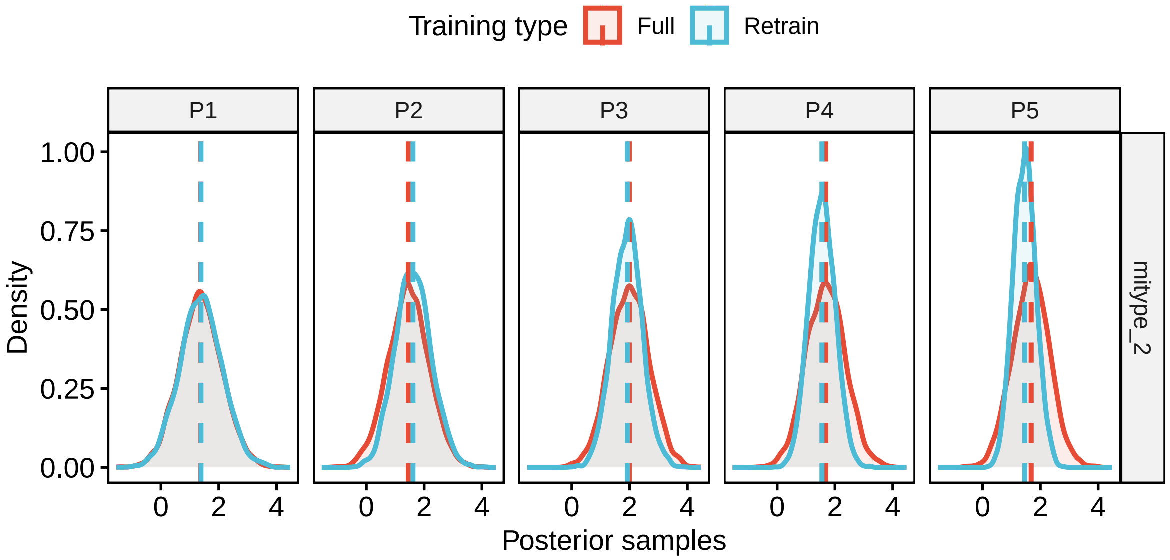
Training type Full Retrain
P1
P2
P3
P4
P5
mitype_2
Density
Posterior samples
1.00
0.75
0.50
0.25
0.00
0
2
4

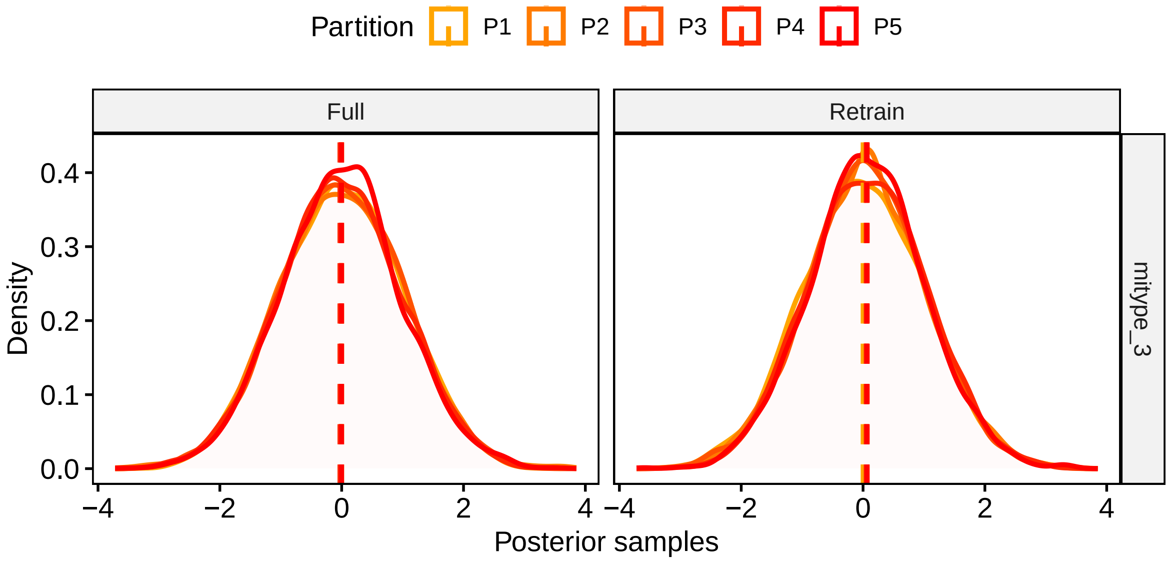
Partition
P1
P2
P3
P4
P5
Full
Retrain
mitype_3
Density
Posterior samples

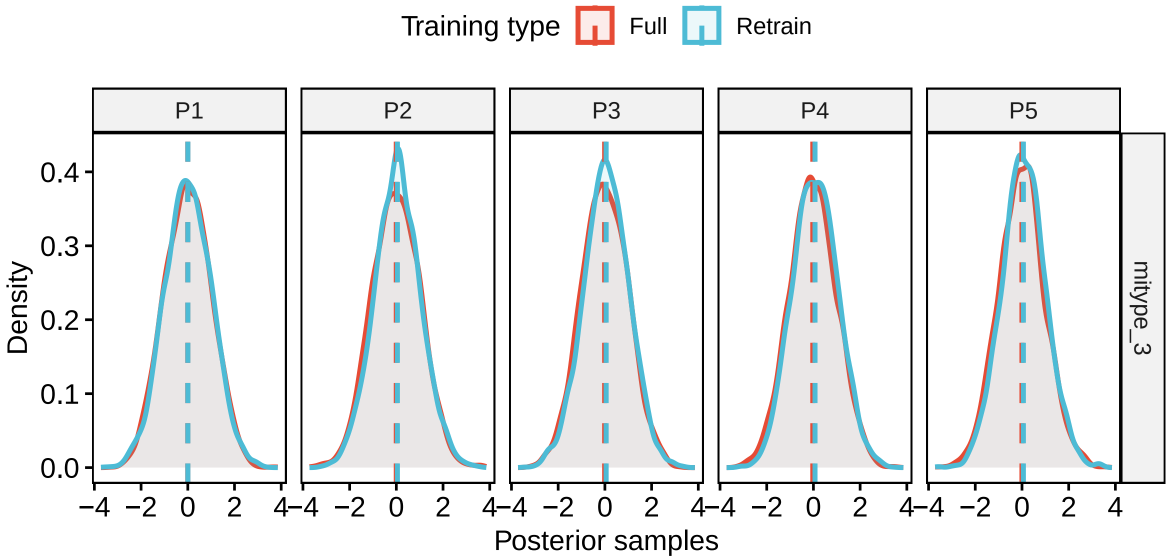
Training type
Full
Retrain
P1
P2
P3
P4
P5
mitype_3
Density
Posterior samples

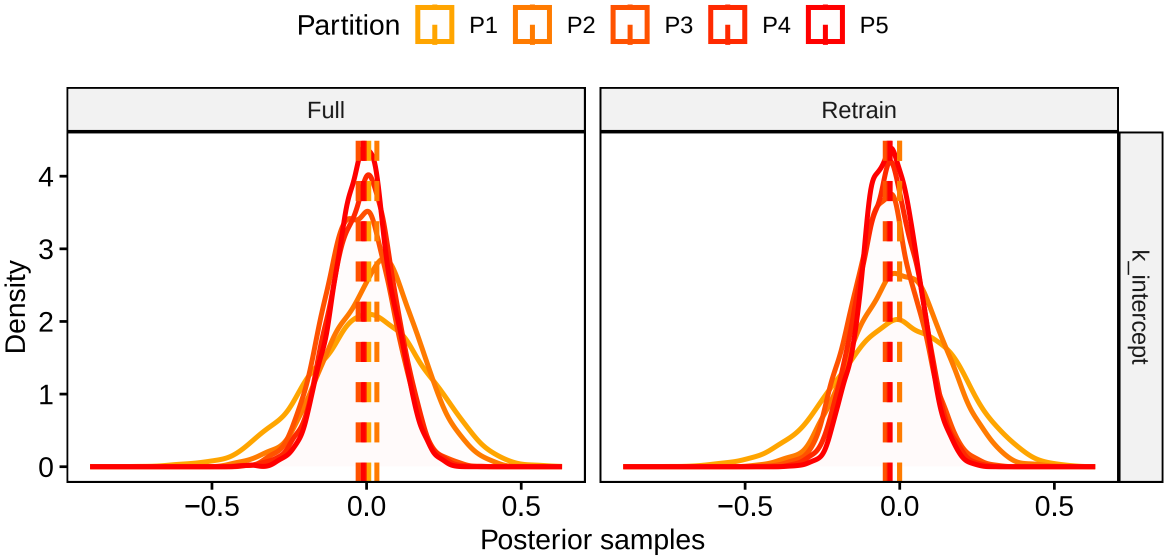

Partition
P1
P2
P3
P4
P5
Full
Retrain
k_intercept
Density
Posterior samples

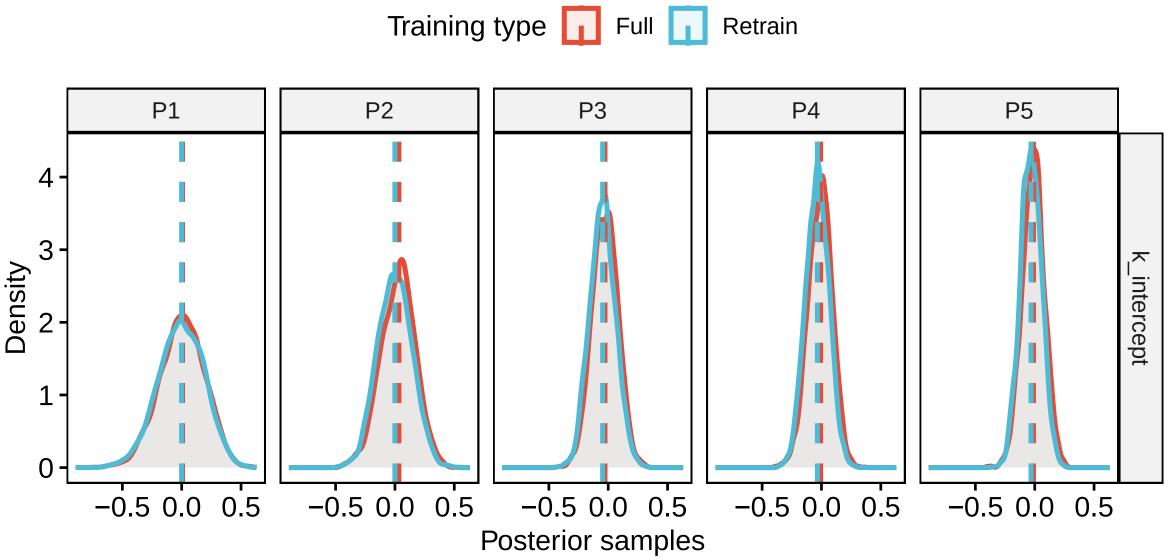

Training type
Full
Retrain
P1
P2
P3
P4
P5
k_intercept
Density
Posterior samples

GBCS

```
gbcs_vars <- data %>% filter(experiment == 'gbcs') %>% select(var) %>% unique(.)


for(var_idx in unique(gbcs_vars$var)){
  p1 <- plot_density(data, 'gbcs', 'pm_wb', var_idx)
  plot(p1)

  p <- plot_density_2(data, 'gbcs', 'pm_wb', var_idx)
  plot(p)
}
```

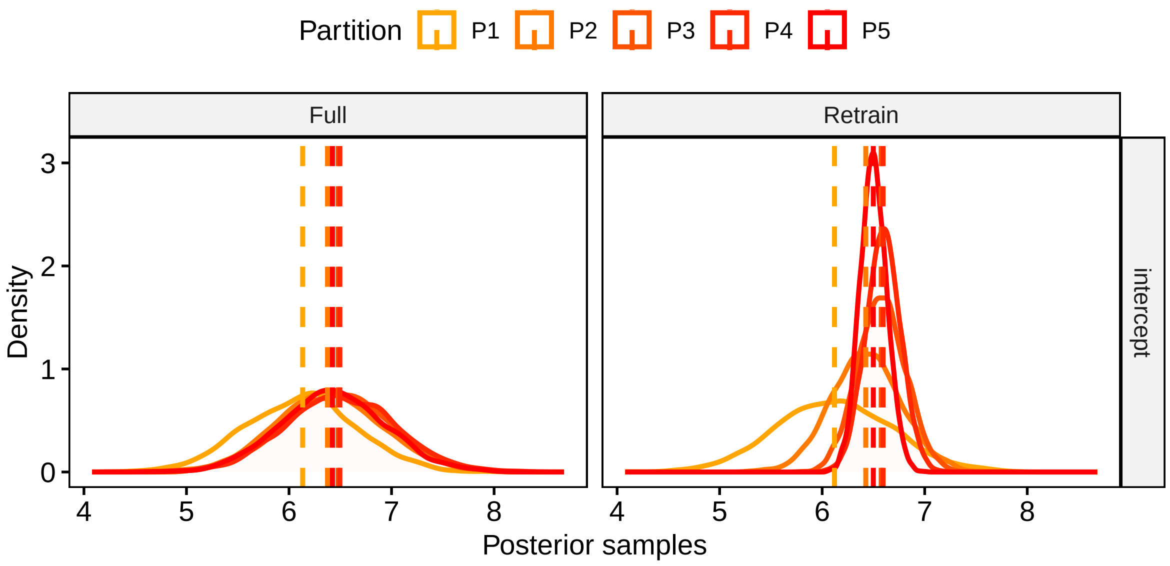

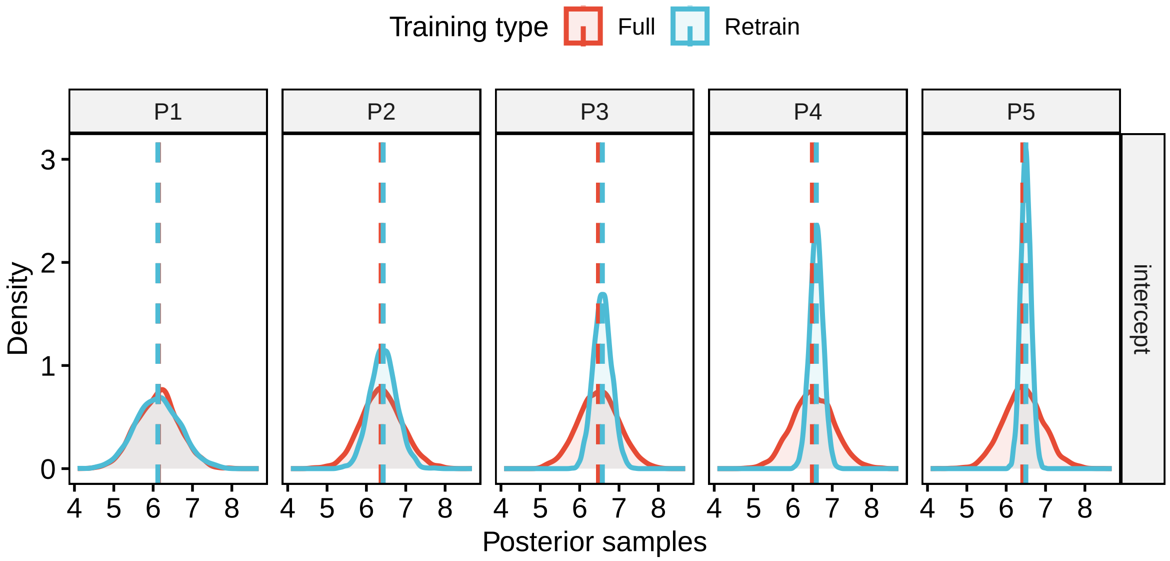
Training type
Full
Retrain
P1
P2
P3
P4
P5
intercept
Density
Posterior samples

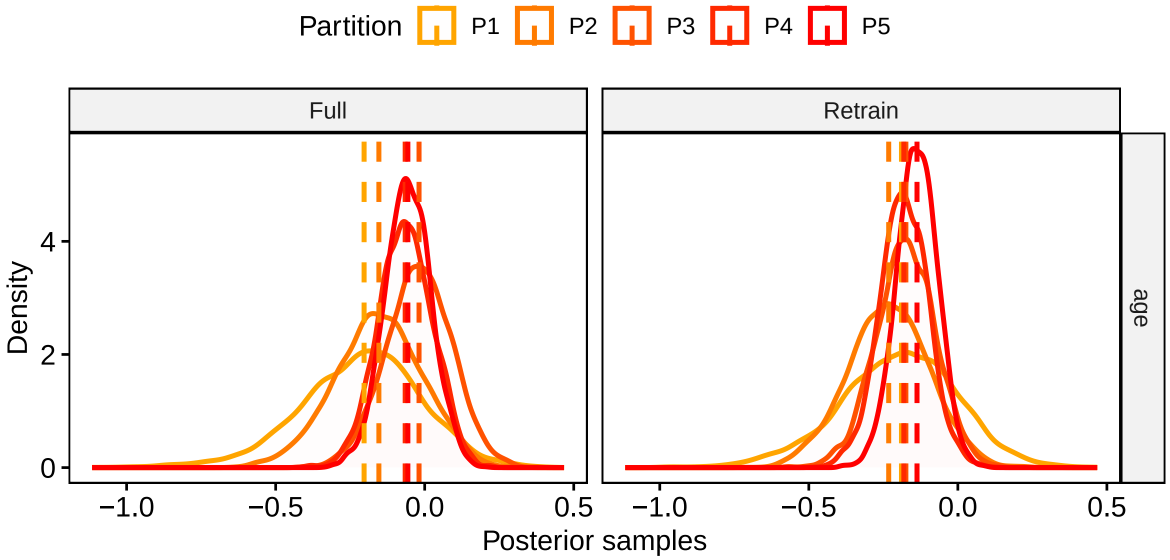
Partition
P1
P2
P3
P4
P5
Full
Retrain
age
Density
Posterior samples

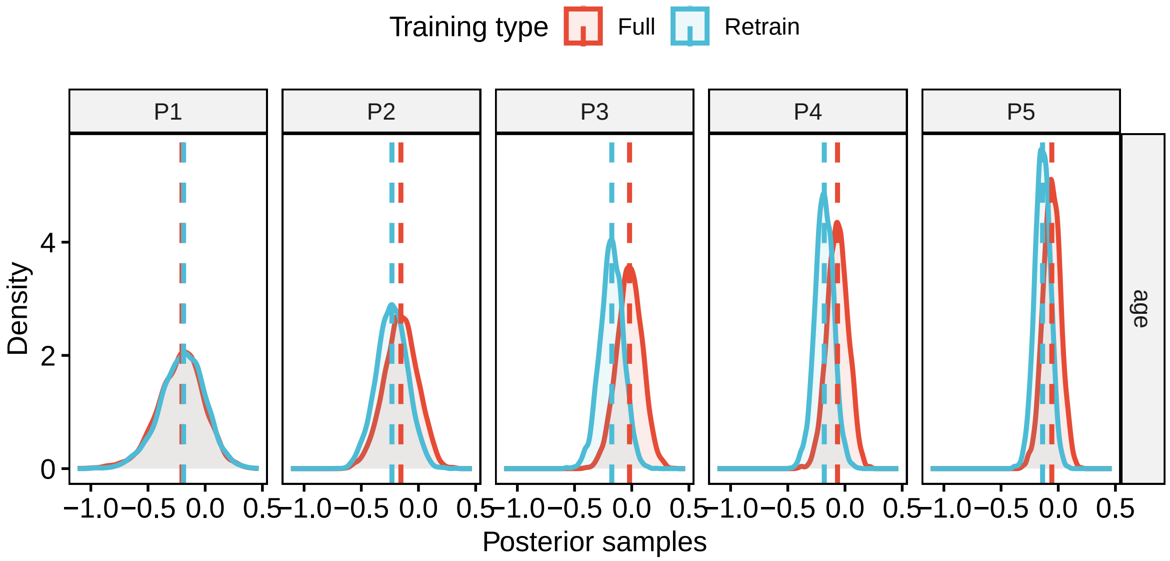
Training type
Full
Retrain
P1
P2
P3
P4
P5
age
Density
Posterior samples

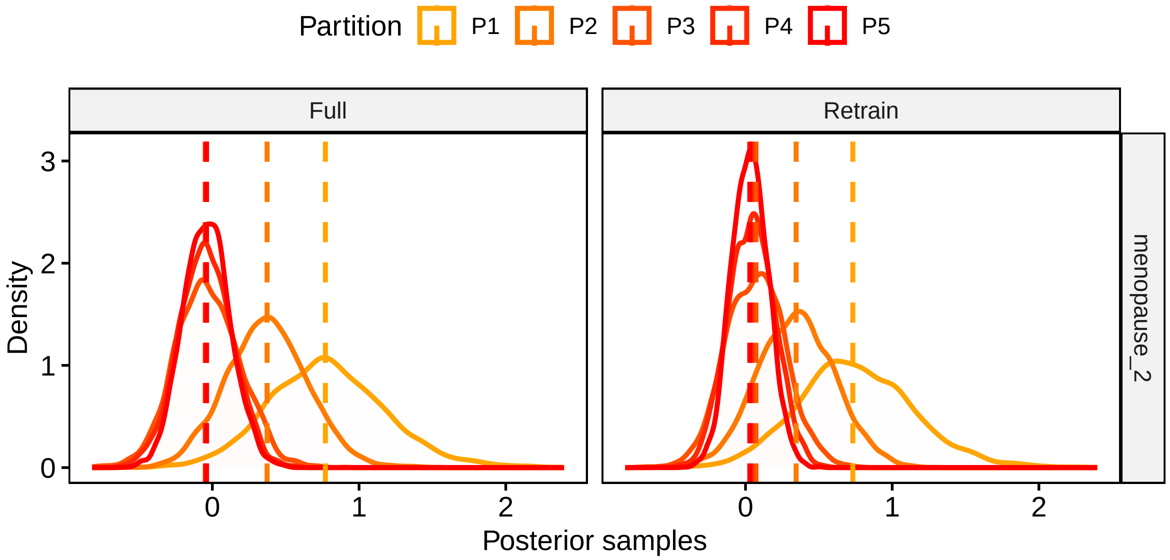
Partition
P1
P2
P3
P4
P5
Full
Retrain
menopause_2
Density
Posterior samples

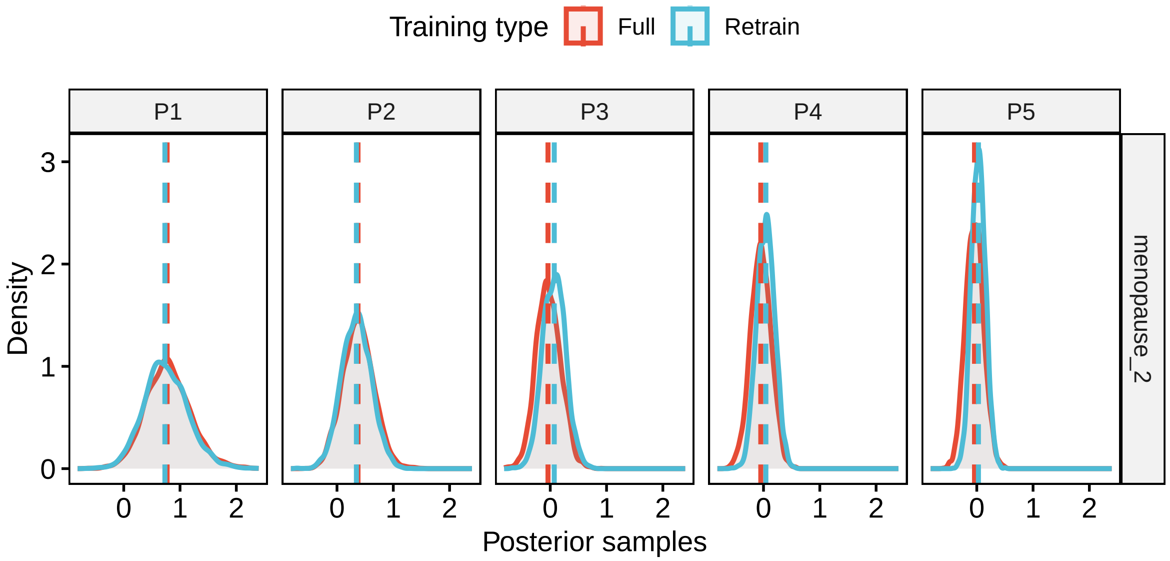
Training type
Full
Retrain
P1
P2
P3
P4
P5
menopause_2
Density
Posterior samples

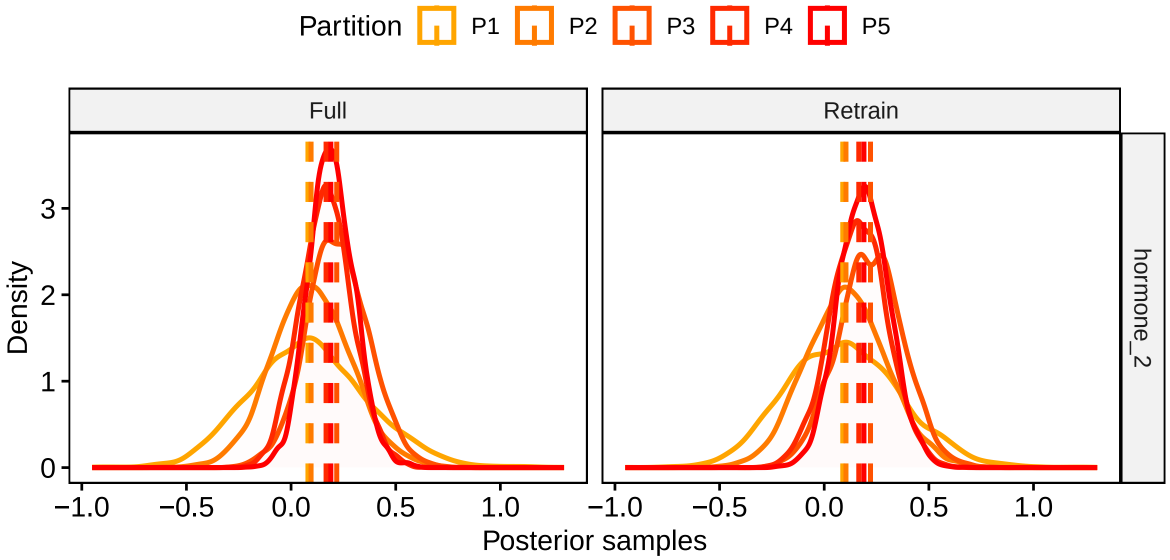
Partition
P1
P2
P3
P4
P5
Full
Retrain
hormone_2
Density
Posterior samples

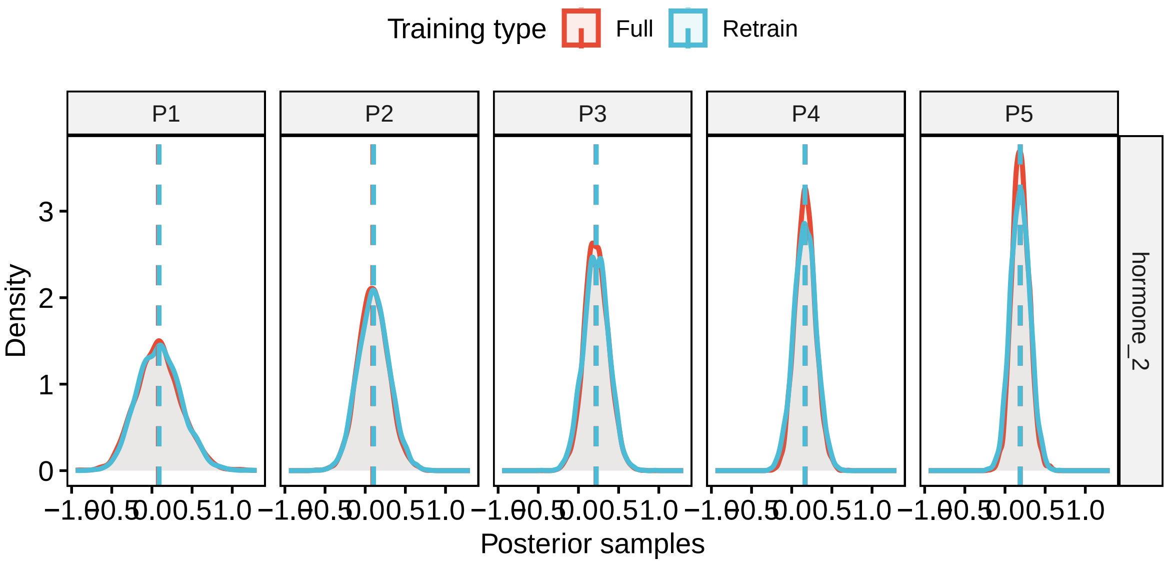
Training type
Full
Retrain
P1
P2
P3
P4
P5
hormone_2
Density
Posterior samples

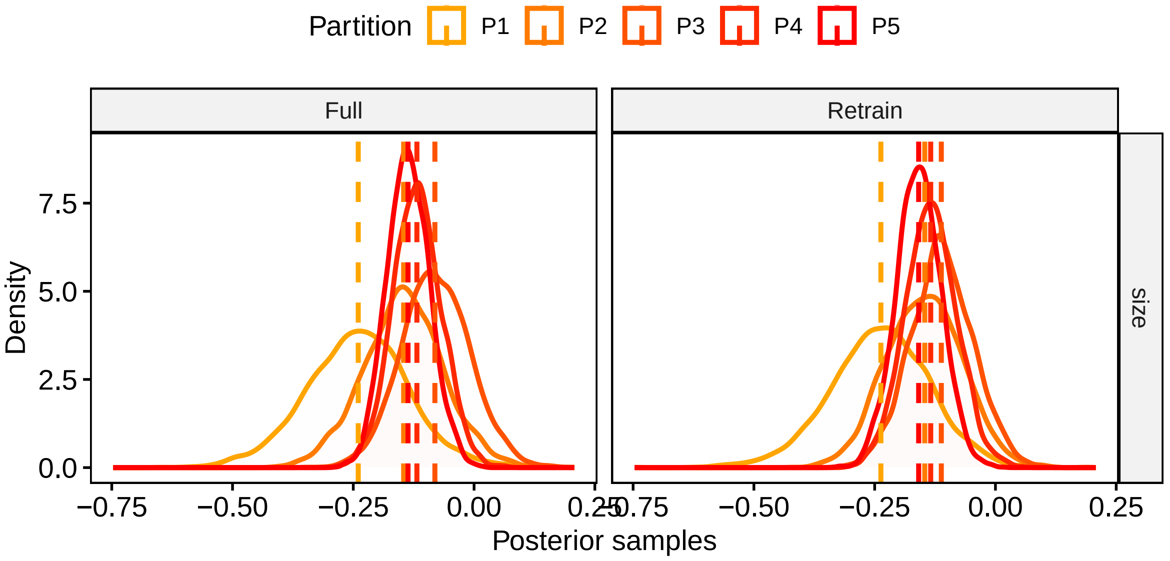
Partition
P1
P2
P3
P4
P5
Full
Retrain
size
Density
Posterior samples

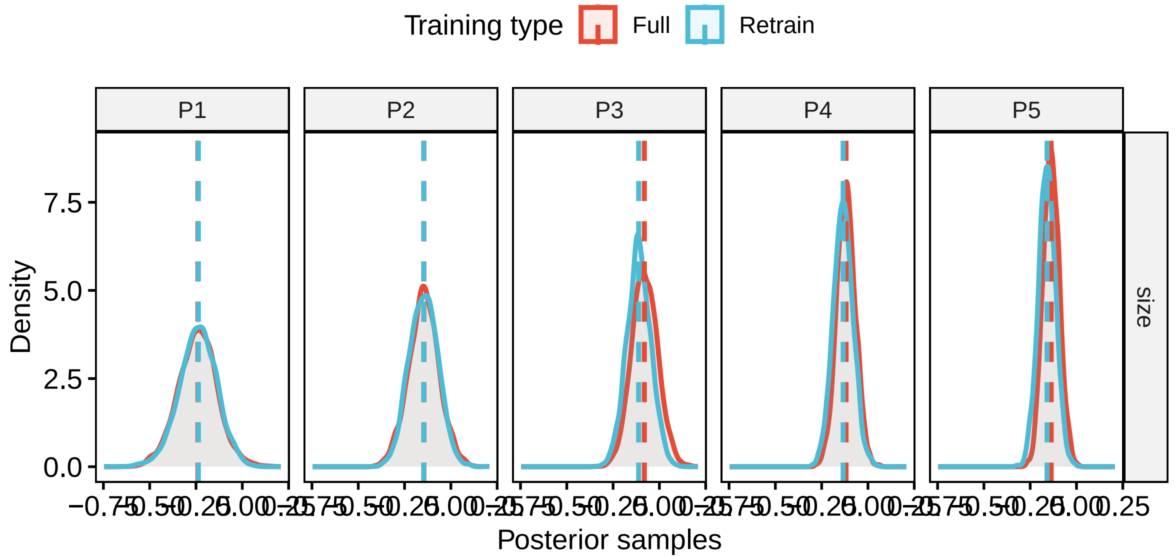

Training type
Full
Retrain
P1
P2
P3
P4
P5
size
Density
Posterior samples

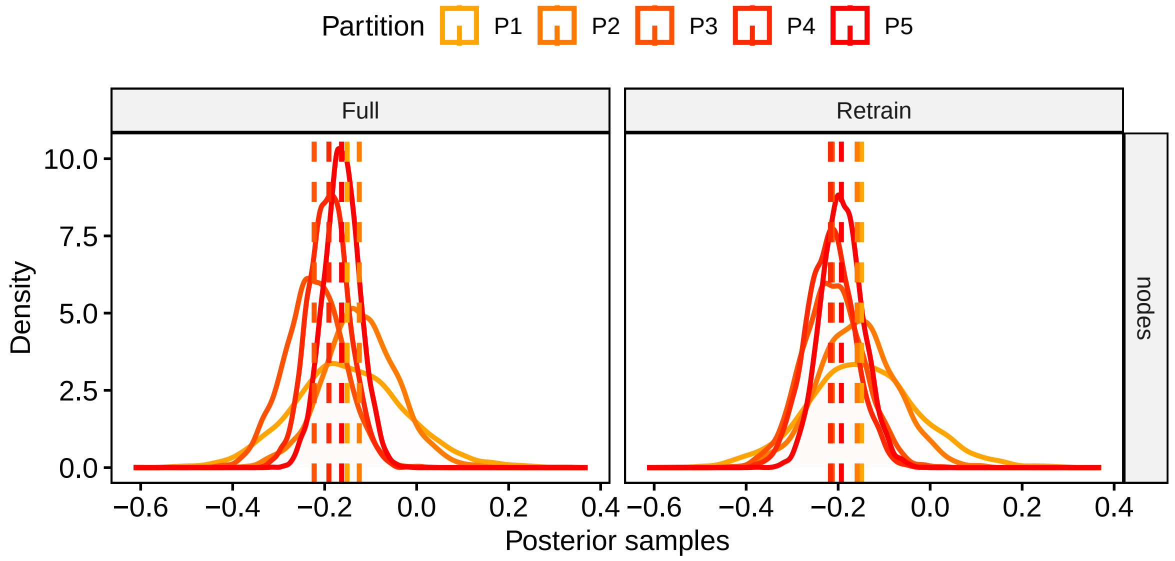

Partition
P1
P2
P3
P4
P5
Full
Retrain
nodes
Density
Posterior samples

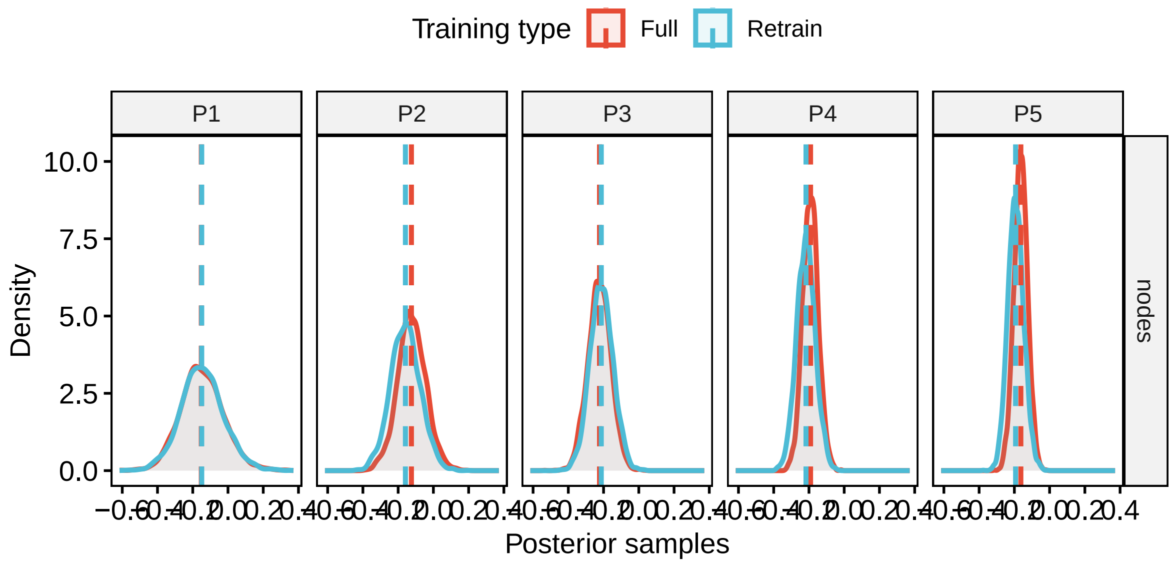
Training type
Full
Retrain
P1
P2
P3
P4
P5
nodes
Density
Posterior samples

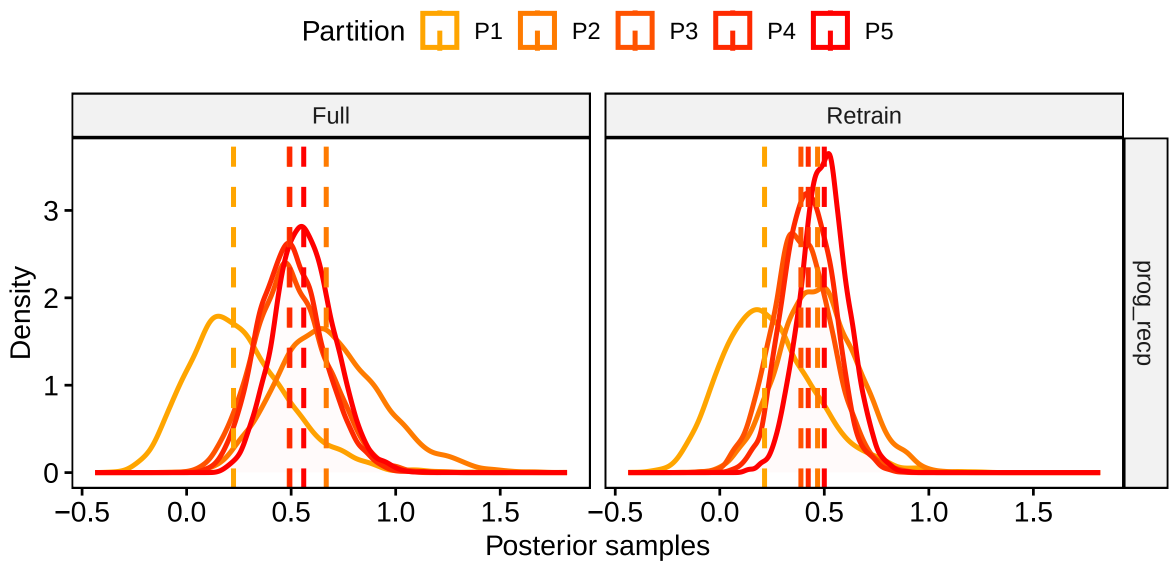
Partition
P1
P2
P3
P4
P5
Full
Retrain
prog_recp
Density
Posterior samples

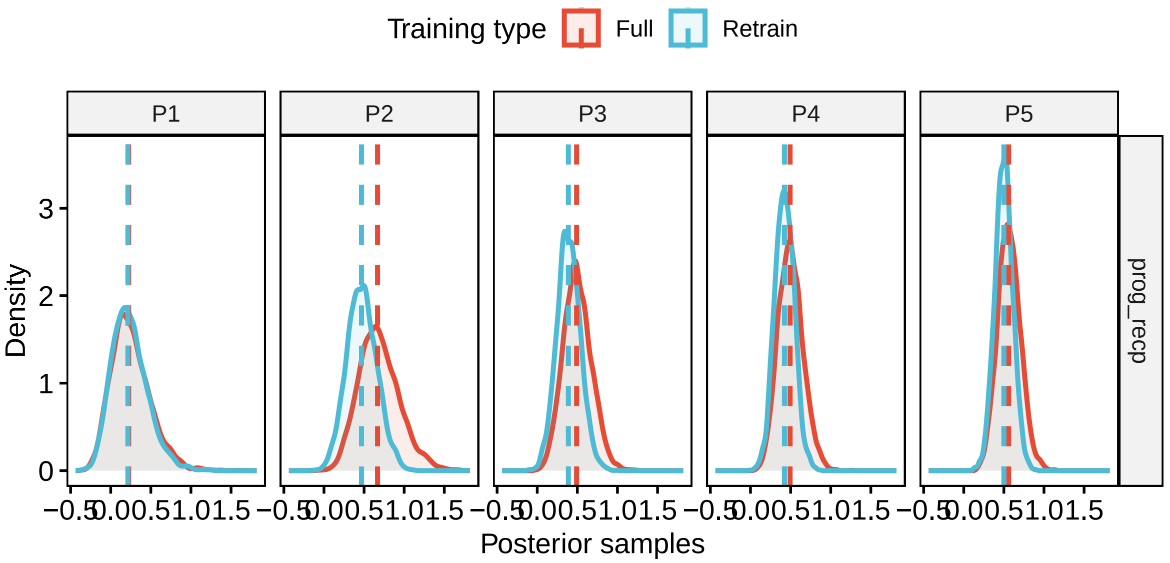
Training type
Full
Retrain
P1
P2
P3
P4
P5
prog_recp
Density
Posterior samples

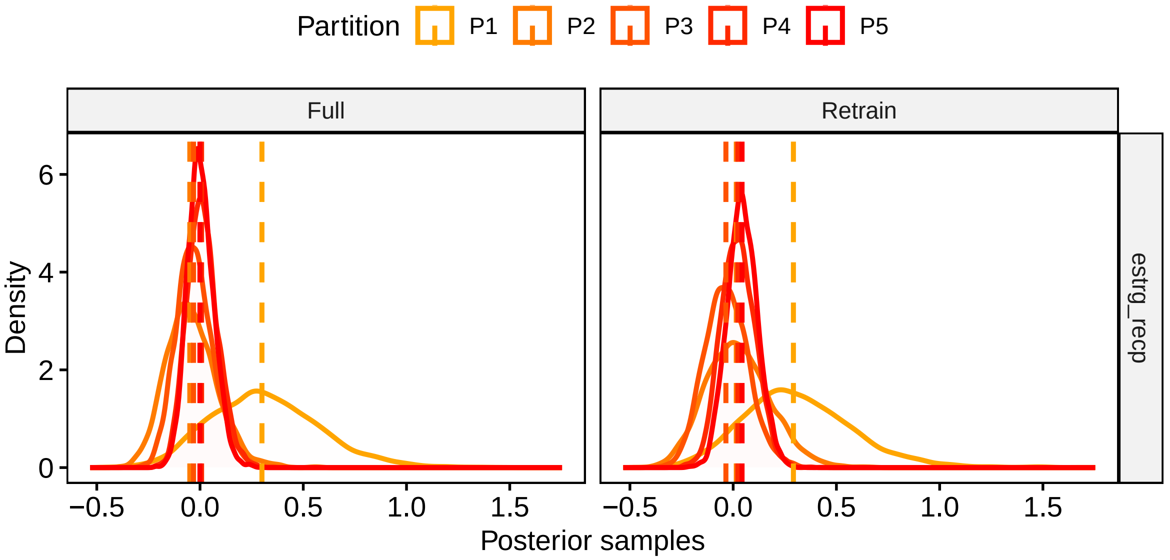
Partition
P1
P2
P3
P4
P5
Full
Retrain
estrg_recp
Density
Posterior samples

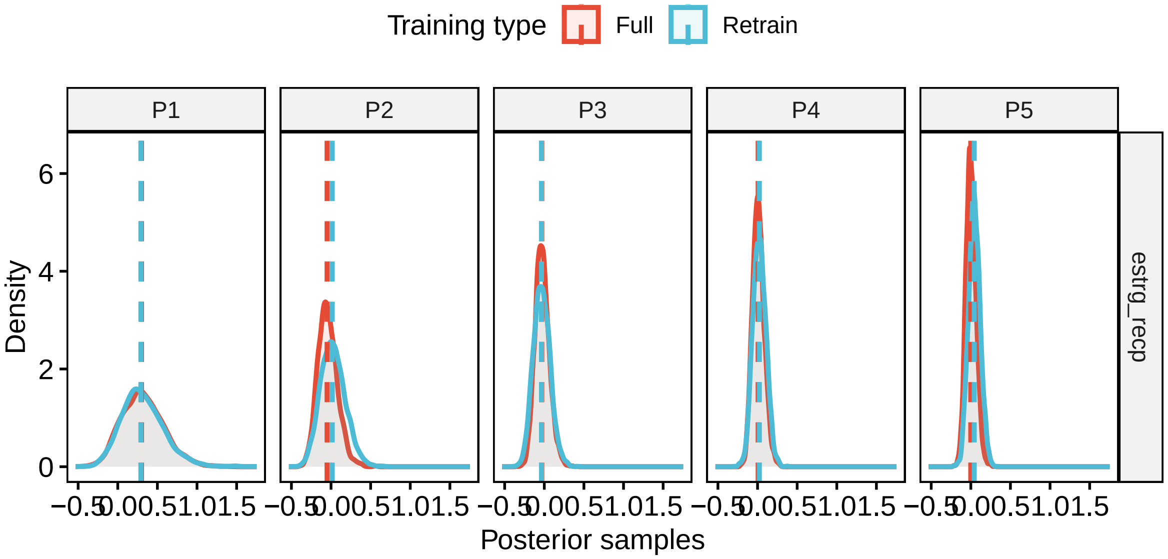
Training type
Full
Retrain
P1
P2
P3
P4
P5
estrg_recp
Density
Posterior samples

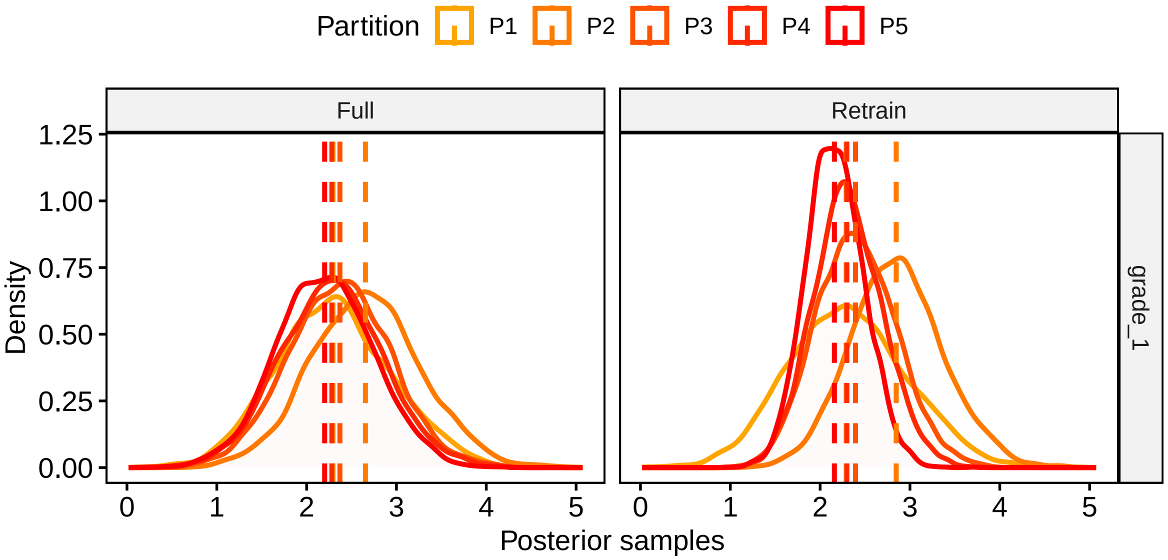
Partition
P1
P2
P3
P4
P5
Full
Retrain
grade_1
Density
Posterior samples

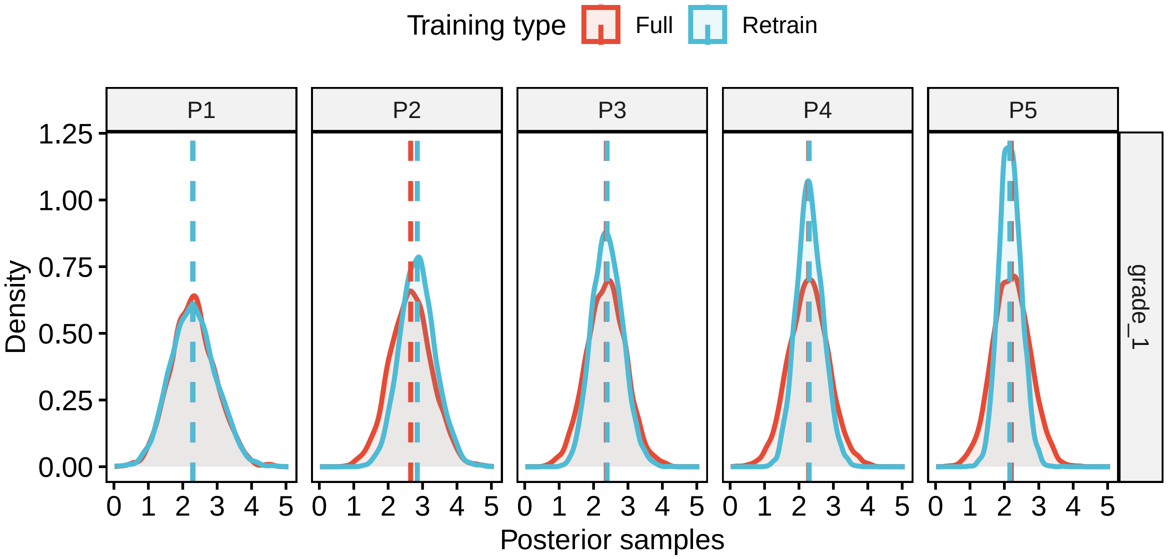

Training type
Full
Retrain
P1
P2
P3
P4
P5
1.25
1.00
0.75
0.50
0.25
0.00
Density
grade_1
0 1 2 3 4 5
Posterior samples

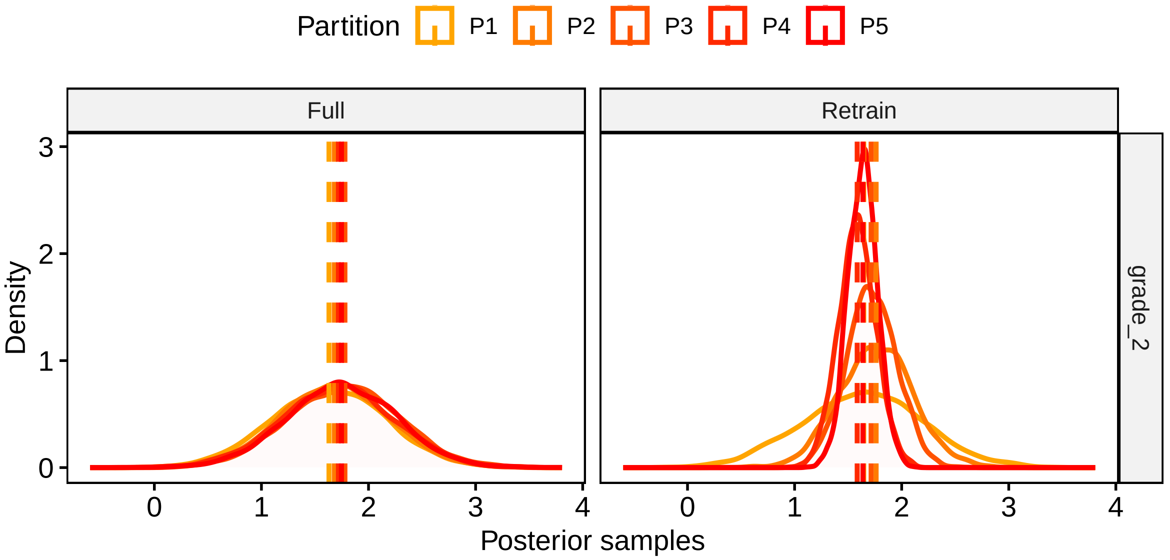

Partition
P1
P2
P3
P4
P5
Full
Retrain
3
2
1
0
Density
grade_2
0 1 2 3 4
Posterior samples

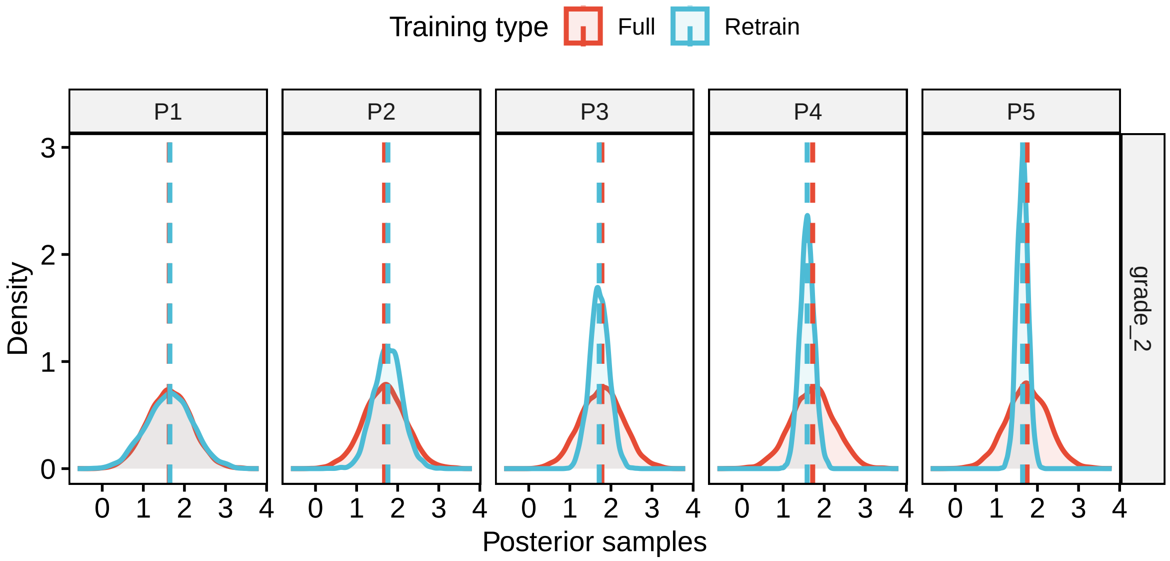

Training type
Full
Retrain
P1
P2
P3
P4
P5
grade_2
Density
Posterior samples

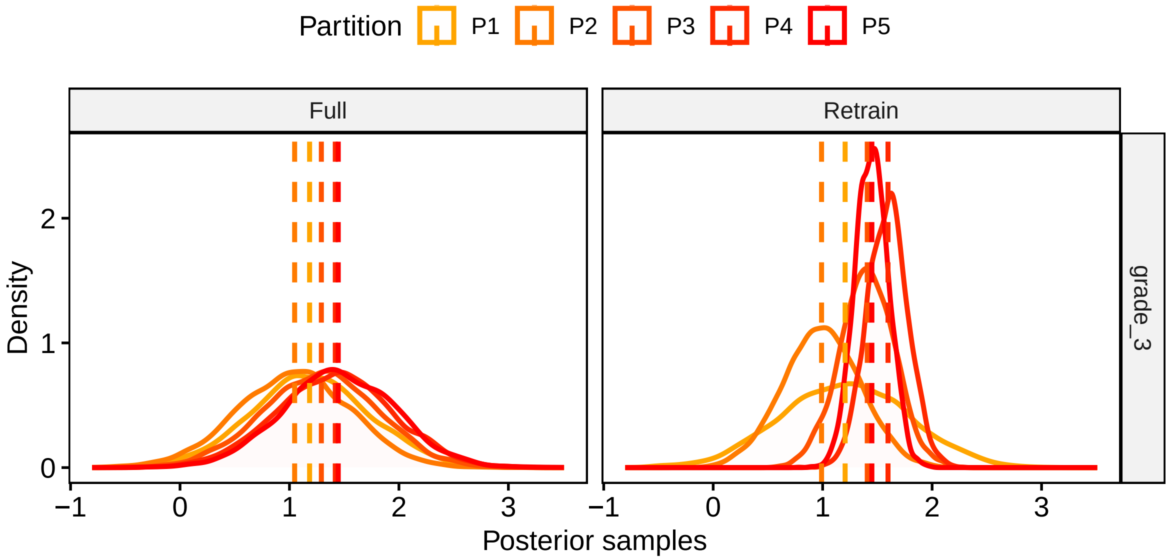

Partition
P1
P2
P3
P4
P5
Full
Retrain
grade_3
Density
Posterior samples

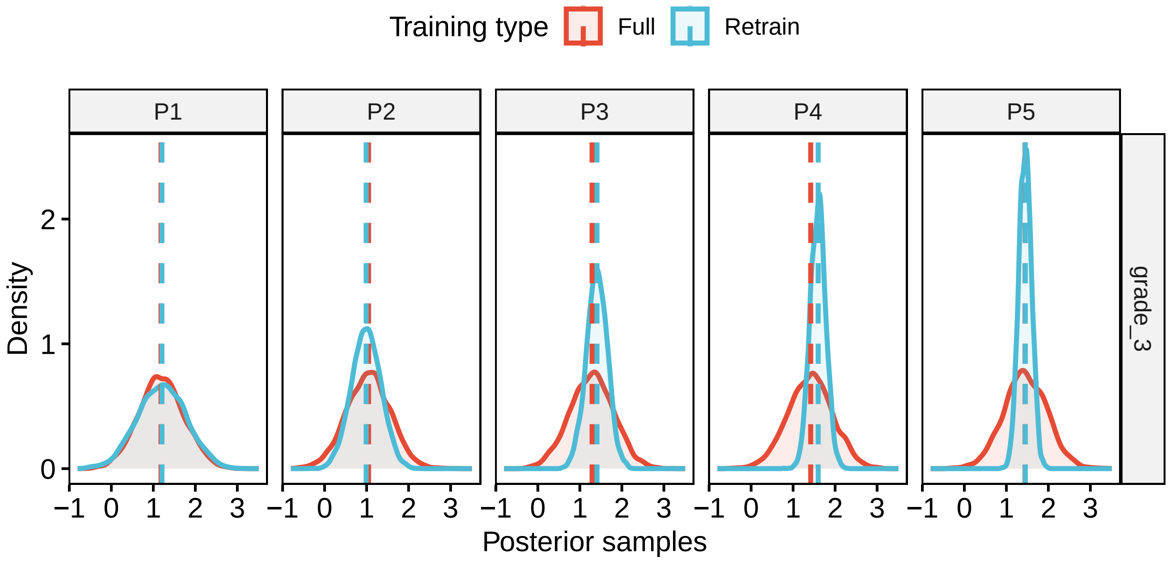

Training type
Full
Retrain
P1
P2
P3
P4
P5
grade_3
Density
Posterior samples

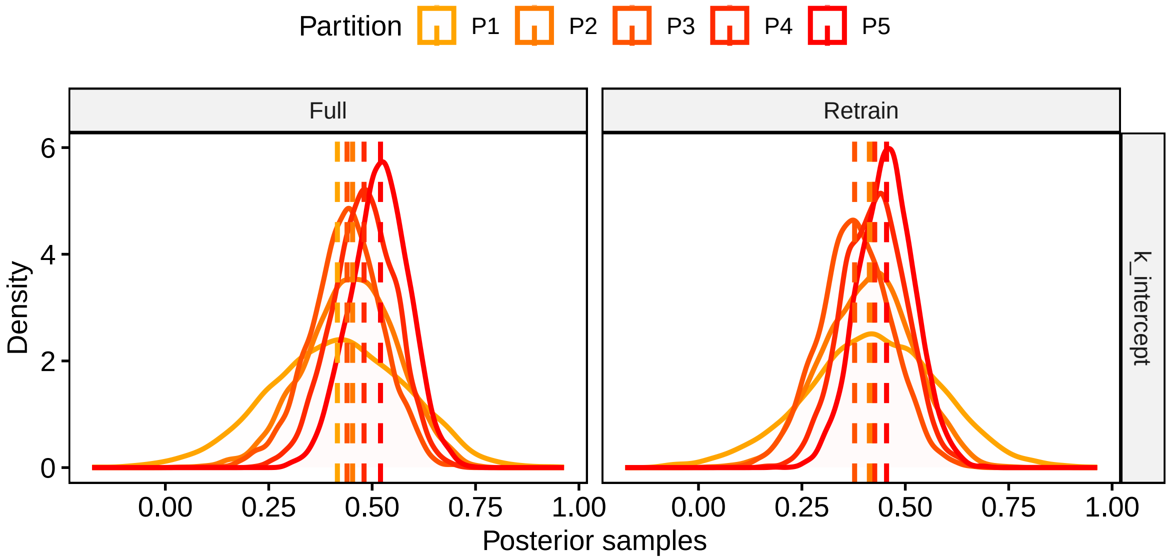

Partition
P1
P2
P3
P4
P5
Full
Retrain
k_intercept
Density
Posterior samples

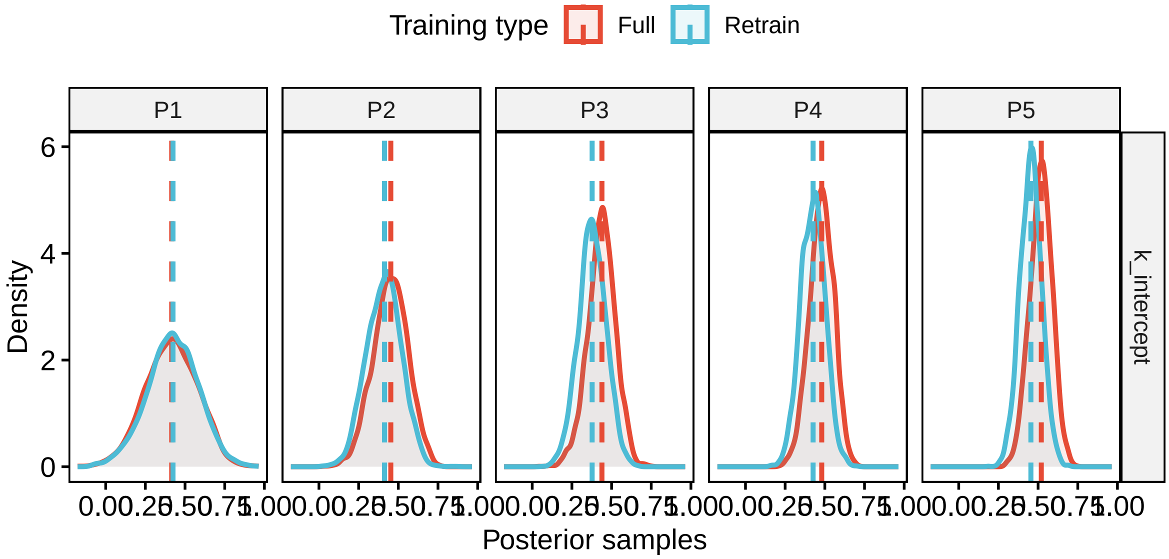

PBC

```
pbc_vars <- data %>% filter(experiment == 'pbc') %>% select(var) %>% unique(.)

for(var_idx in unique(pbc_vars$var)){
  p1 <- plot_density(data, 'pbc', 'pm_wb', var_idx)
  plot(p1)

  p <- plot_density_2(data, 'pbc', 'pm_wb', var_idx)
  plot(p)
}
```

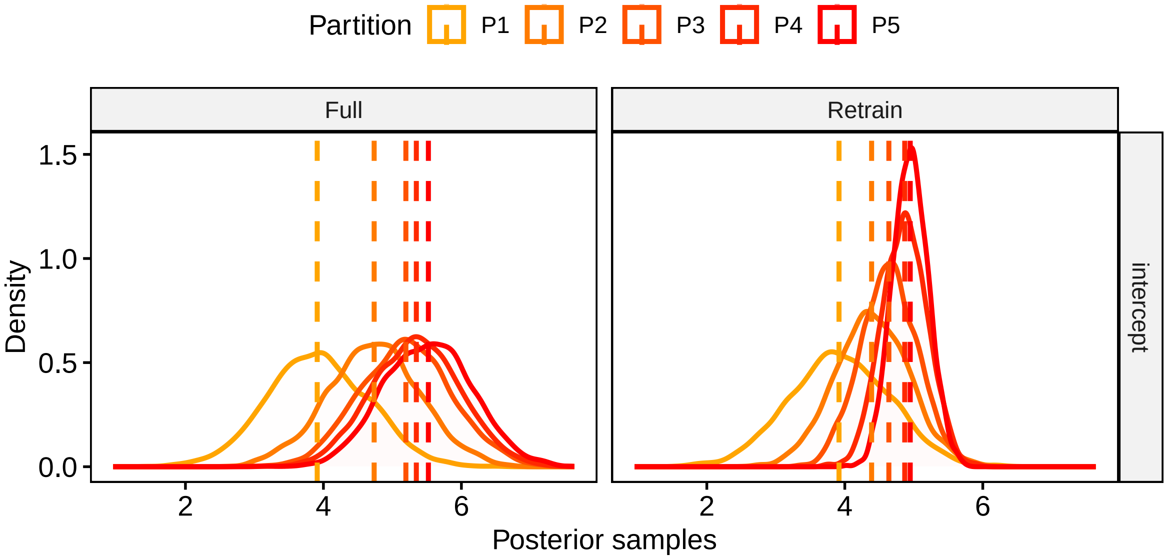

Partition
P1
P2
P3
P4
P5
Full
Retrain
intercept
Density
Posterior samples

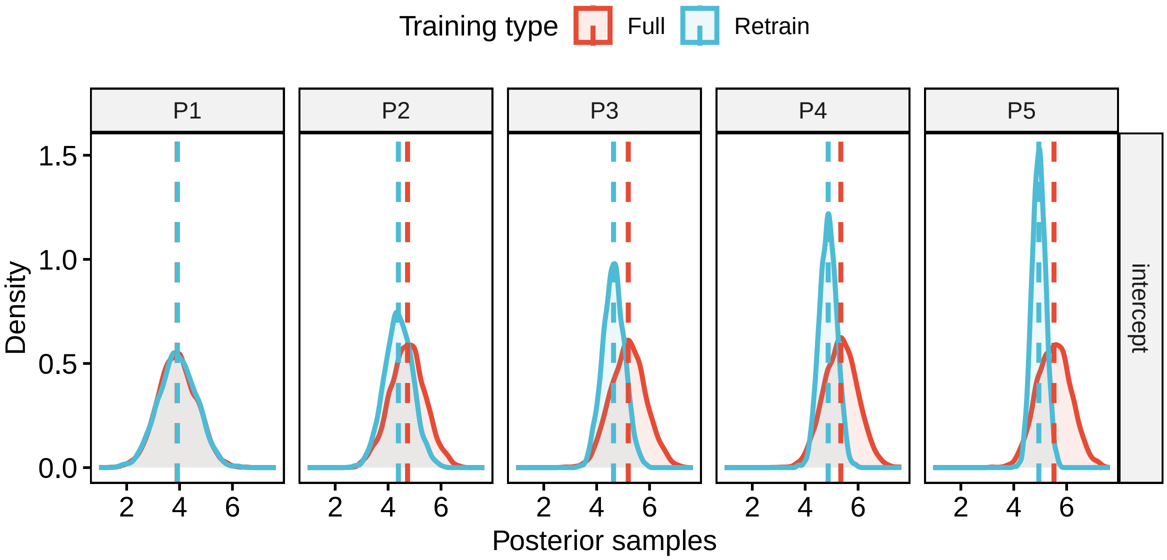

Training type
Full
Retrain
P1
P2
P3
P4
P5
intercept
Density
Posterior samples

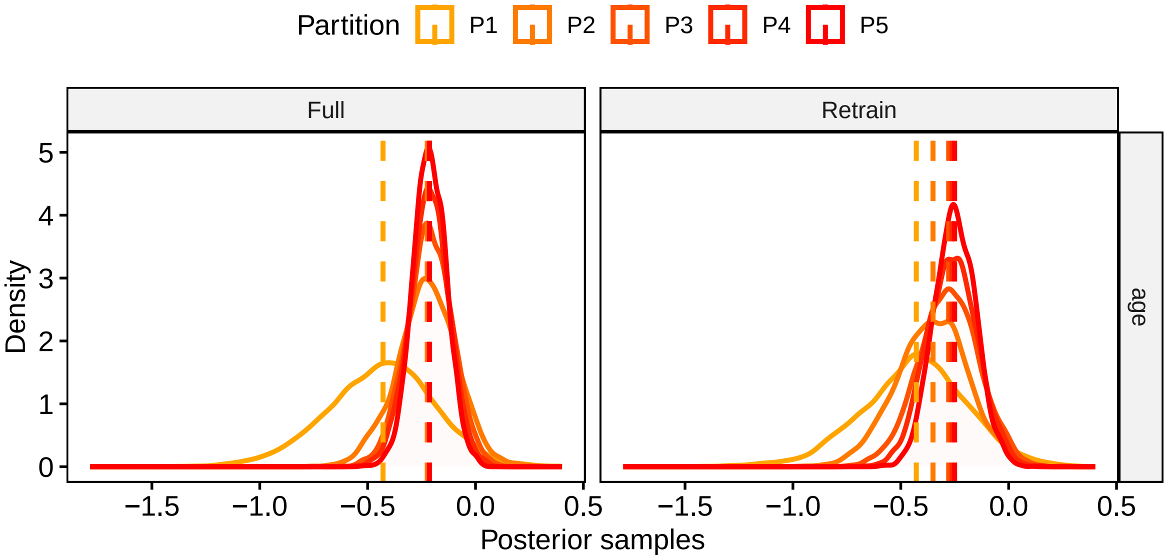
Partition
P1
P2
P3
P4
P5
Full
Retrain
age
Density
Posterior samples

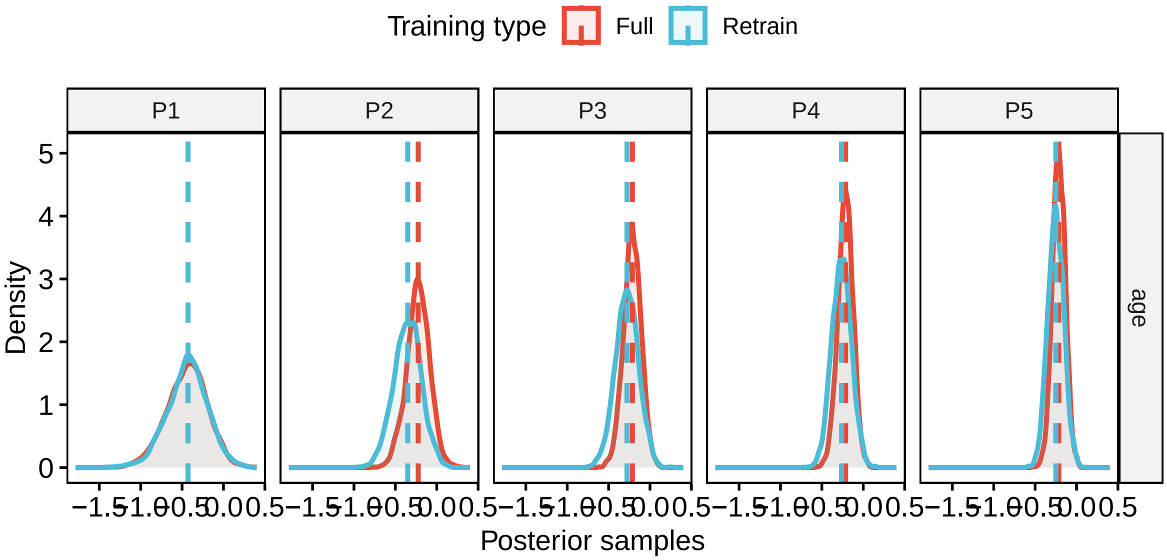
Training type
Full
Retrain
P1
P2
P3
P4
P5
age
Density
Posterior samples

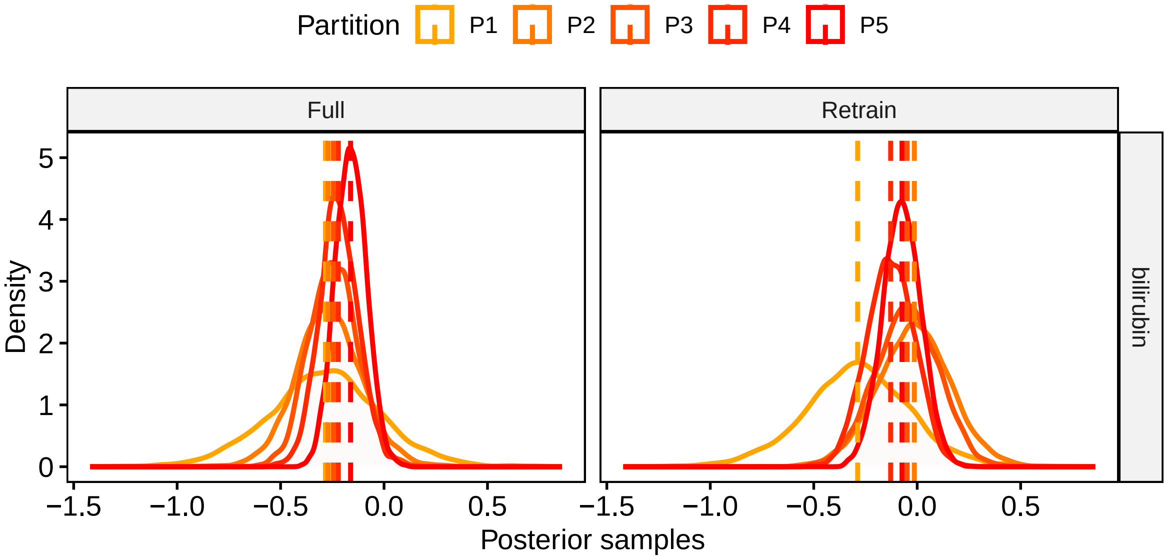

Partition
P1
P2
P3
P4
P5
Full
Retrain
bilirubin
Density
5
4
3
2
1
0
−1.5
−1.0
−0.5
0.0
0.5
Posterior samples

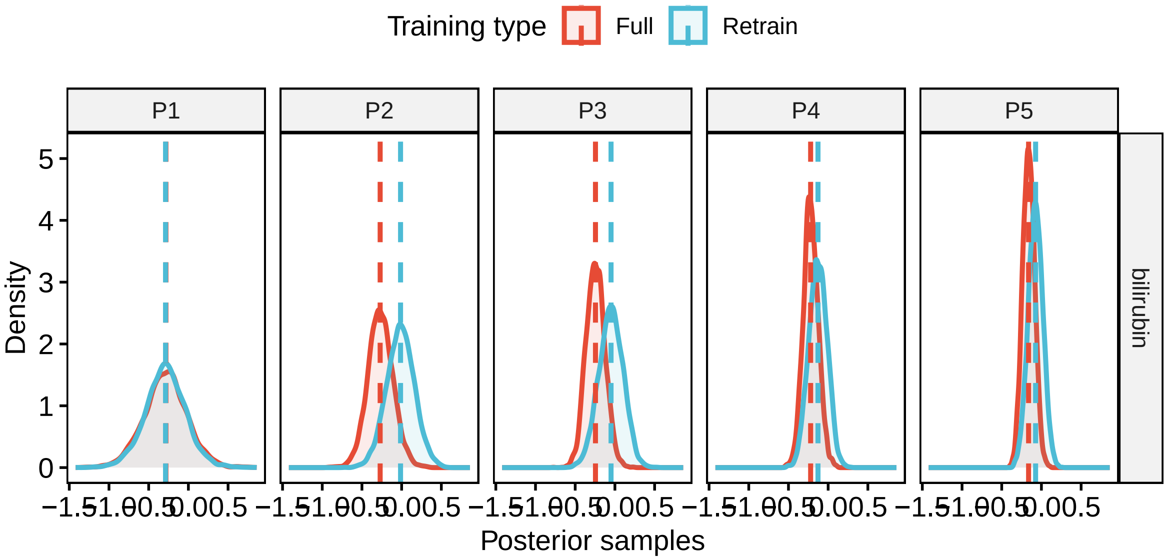

Training type
Full
Retrain
P1
P2
P3
P4
P5
bilirubin
Density
5
4
3
2
1
0
Posterior samples

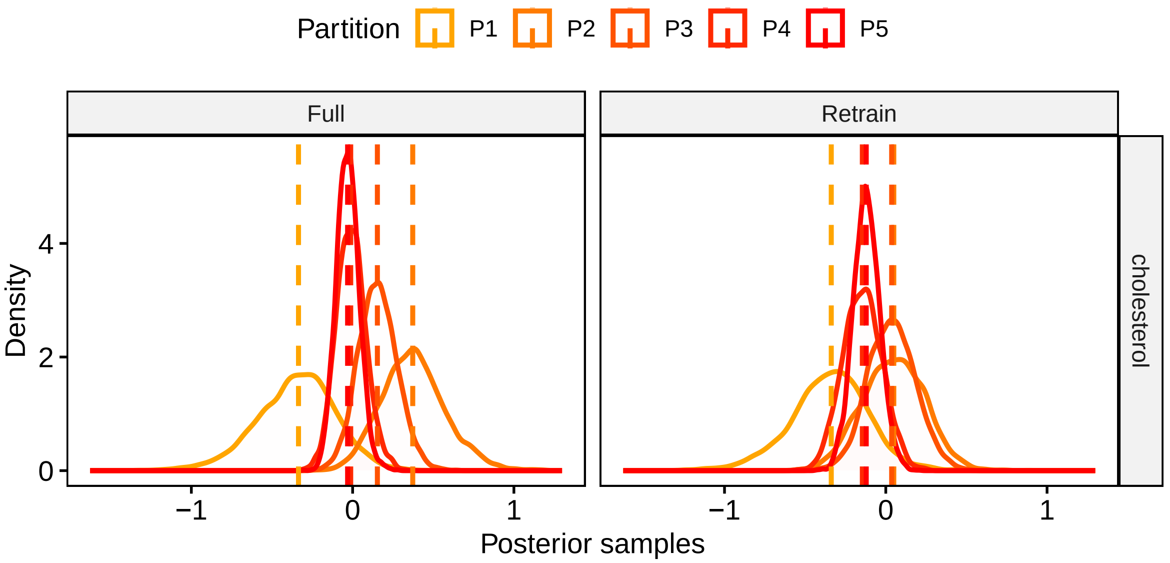
Partition
P1
P2
P3
P4
P5
Full
Retrain
cholesterol
Density
Posterior samples
4
2
0
−1
0
1

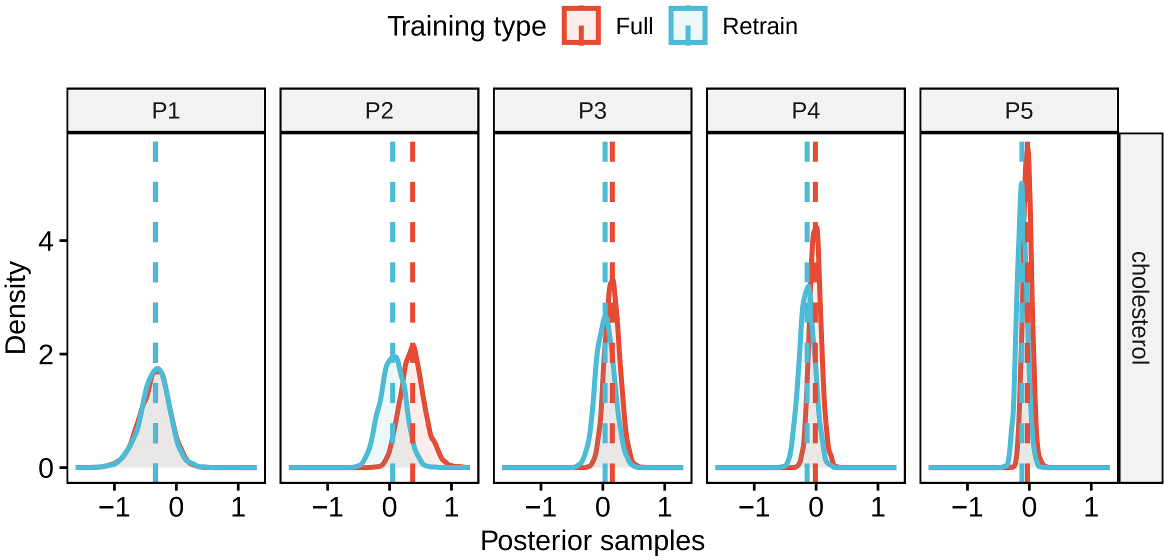
Training type
Full
Retrain
P1
P2
P3
P4
P5
cholesterol
Density
Posterior samples
4
2
0
−1
0
1

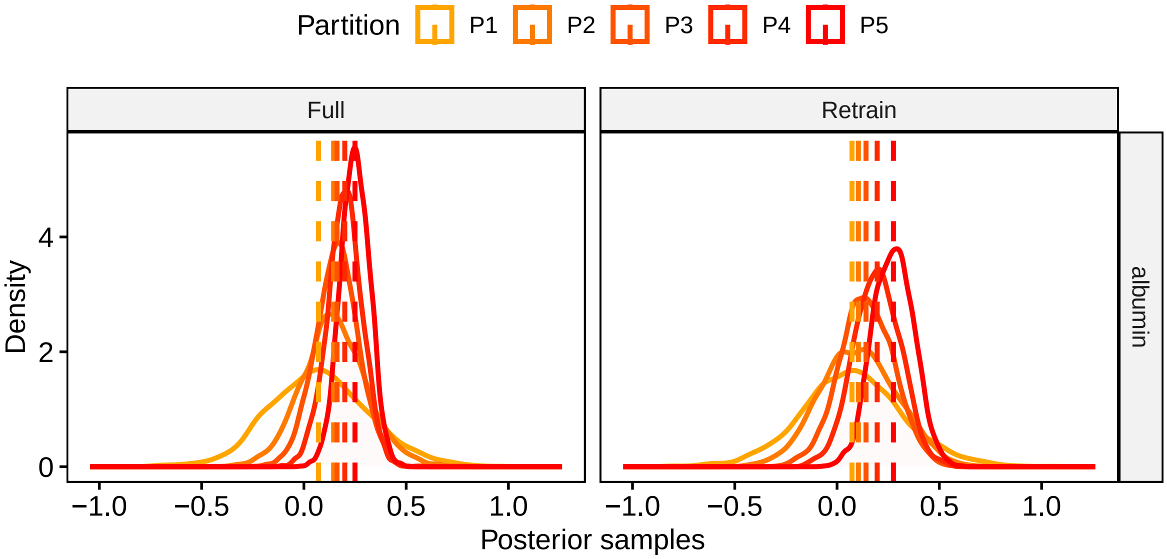
Partition
P1
P2
P3
P4
P5
Full
Retrain
albumin
Density
Posterior samples

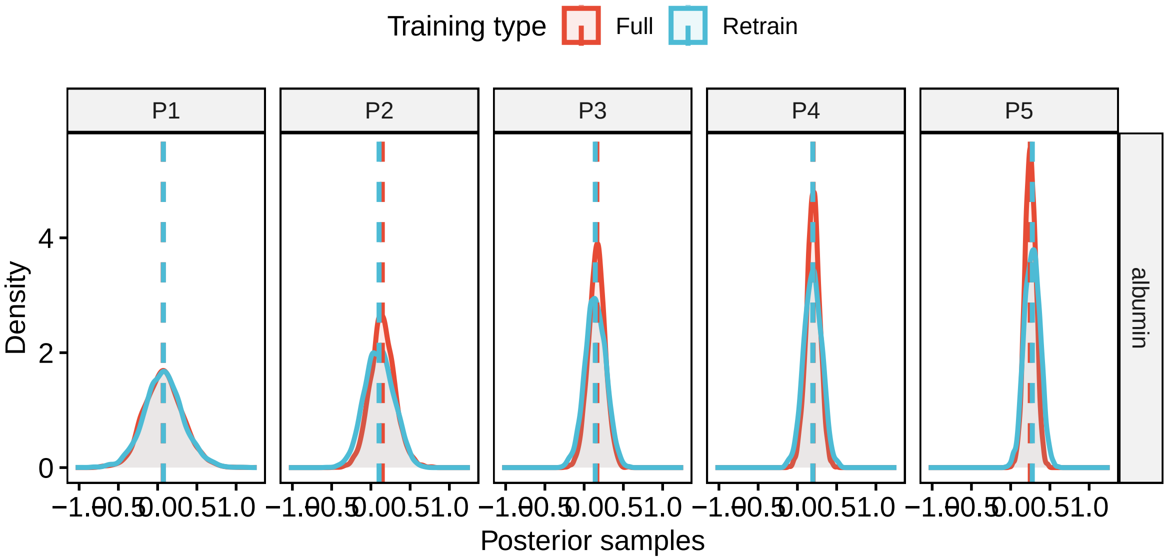
Training type
Full
Retrain
P1
P2
P3
P4
P5
albumin
Density
Posterior samples

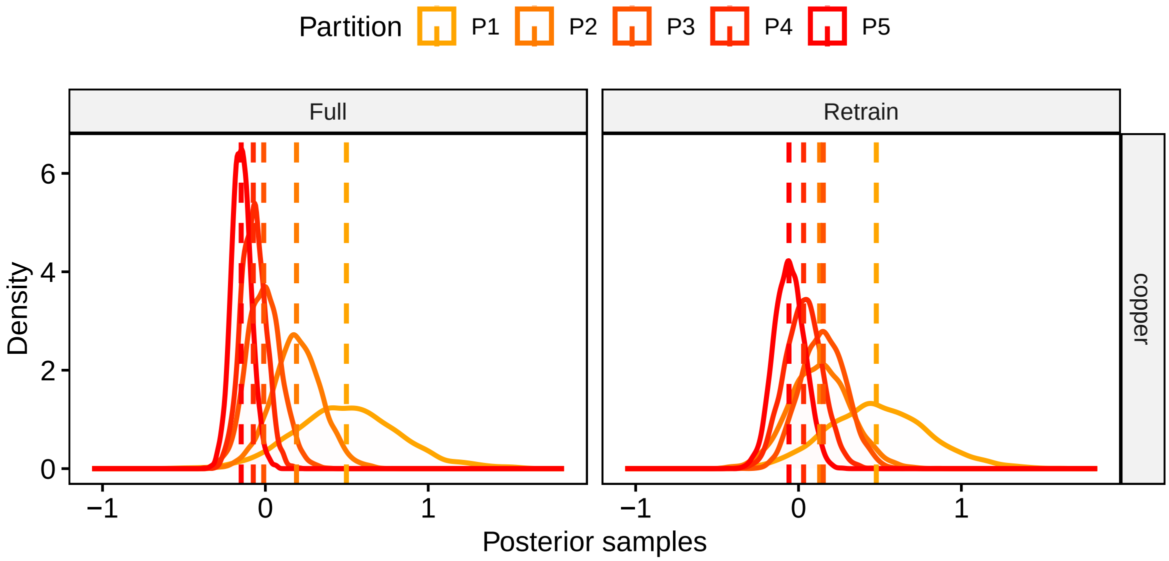
Partition
P1
P2
P3
P4
P5
Full
Retrain
copper
Density
Posterior samples
6
4
2
0
−1
0
1

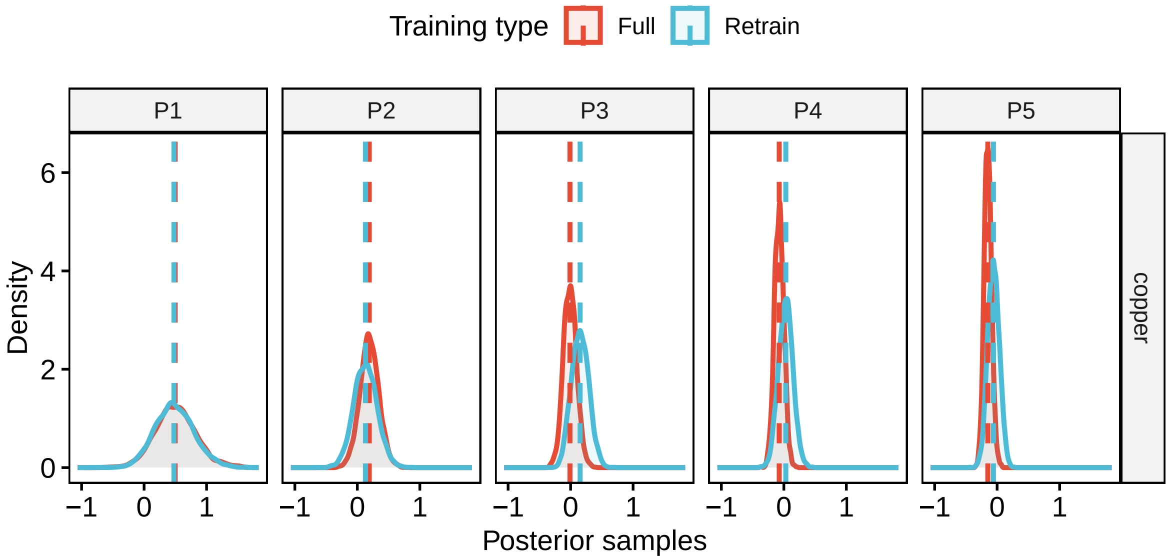
Training type
Full
Retrain
P1
P2
P3
P4
P5
copper
Density
Posterior samples
6
4
2
0
−1
0
1

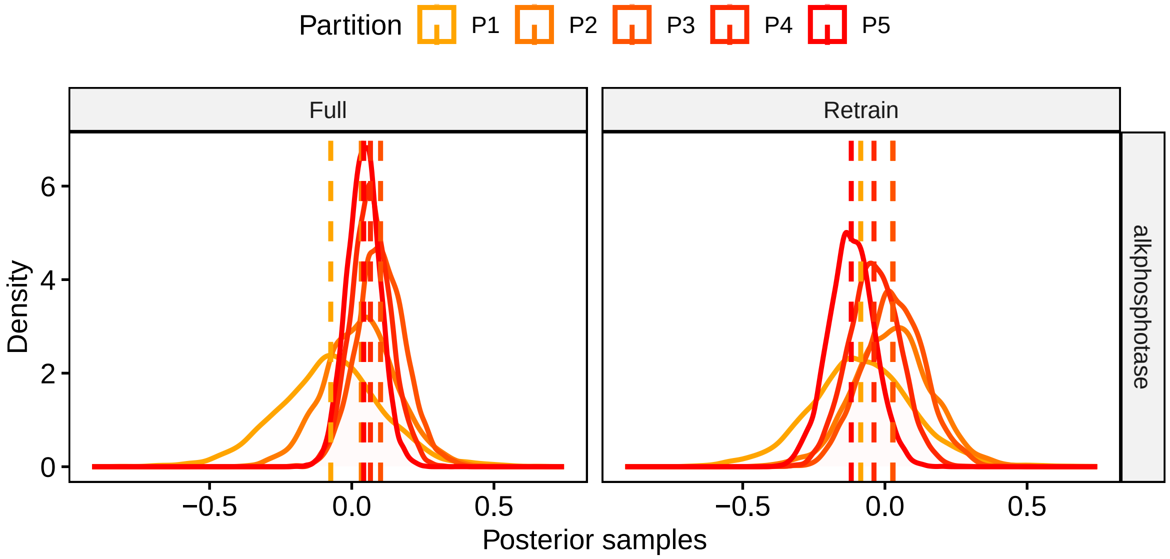
Partition
P1
P2
P3
P4
P5
Full
Retrain
alkphosphotase
Density
Posterior samples

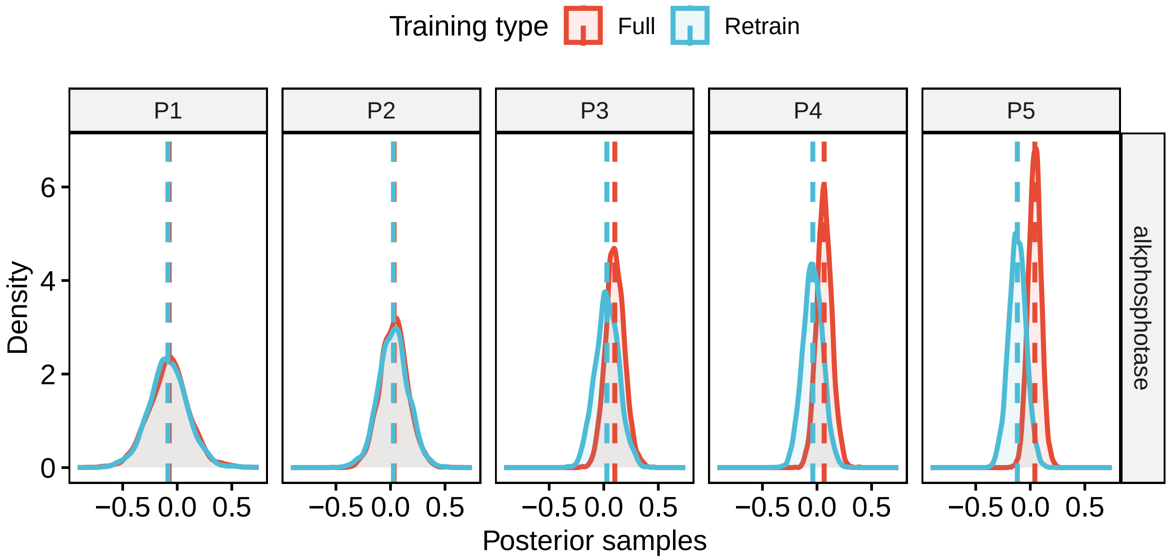
Training type
Full
Retrain
P1
P2
P3
P4
P5
alkphosphotase
Density
Posterior samples

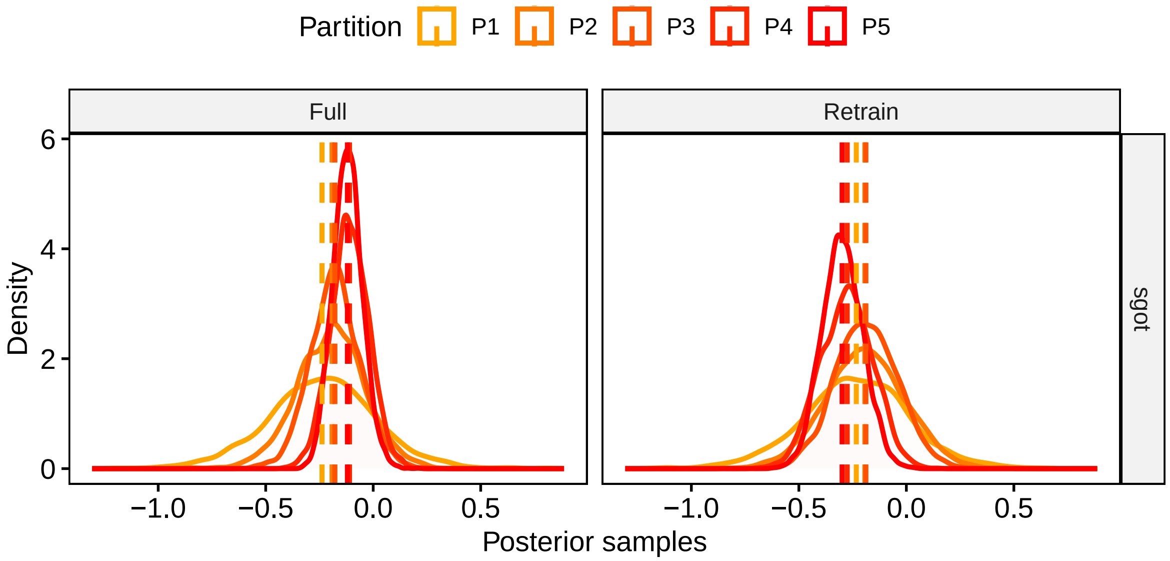
Partition
P1
P2
P3
P4
P5
Full
Retrain
sgot
Density
Posterior samples

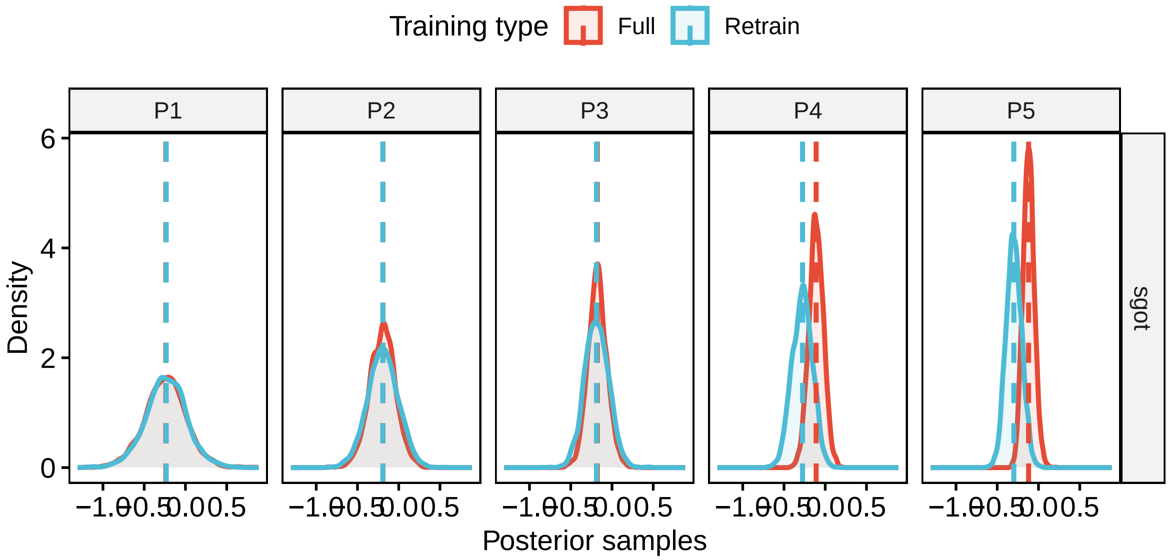
Training type
Full
Retrain
P1
P2
P3
P4
P5
sgot
Density
Posterior samples

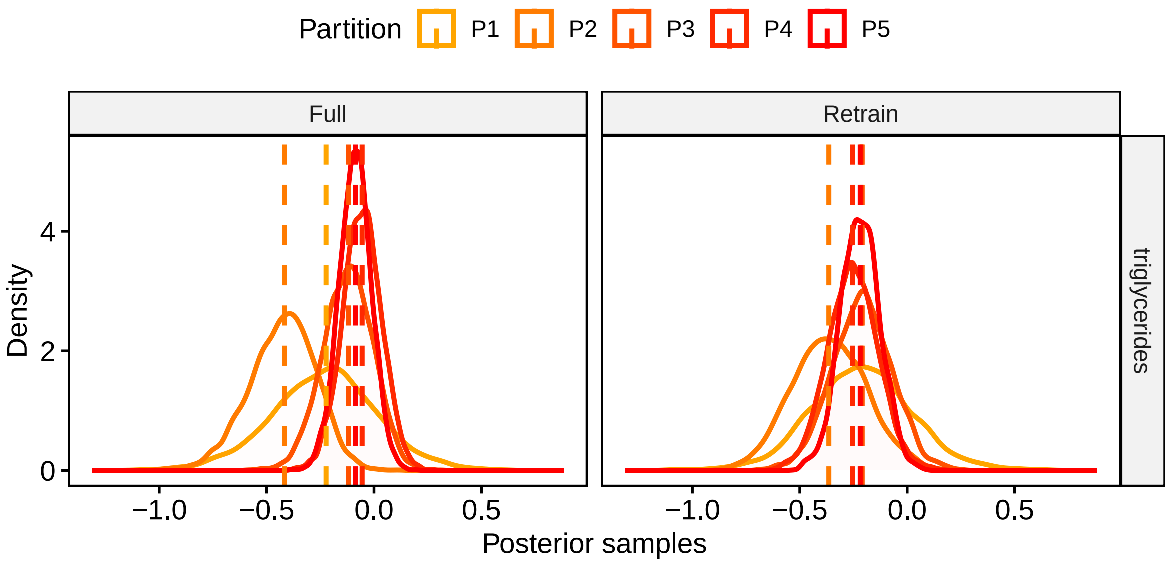
Partition P1 P2 P3 P4 P5
Full
Retrain
triglycerides
Density
4
2
0
−1.0 −0.5 0.0 0.5
Posterior samples

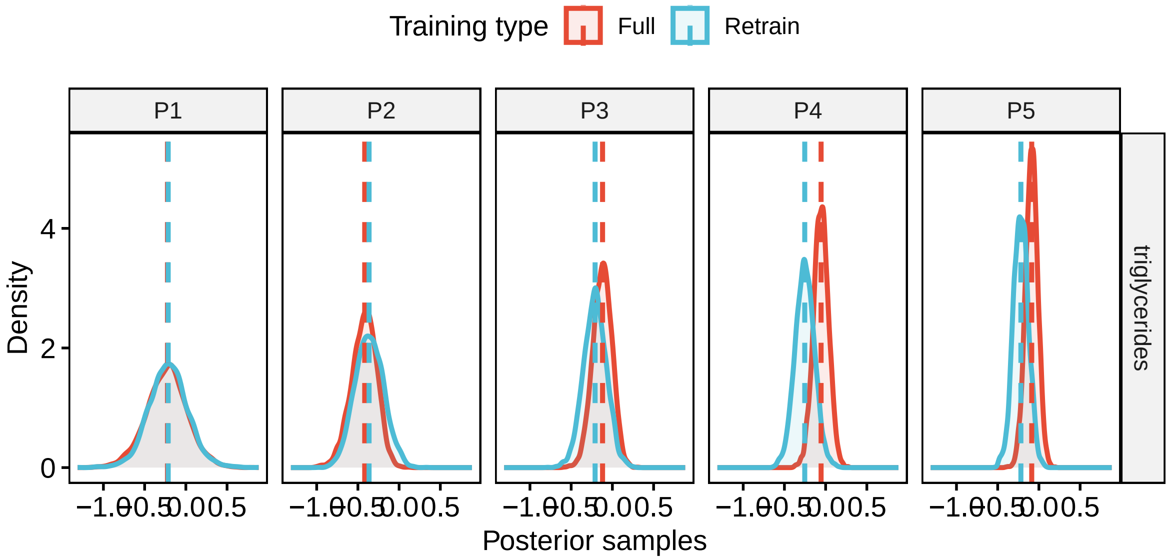
Training type Full Retrain
P1
P2
P3
P4
P5
triglycerides
Density
4
2
0
−1.0−0.5 0.0 0.5
Posterior samples

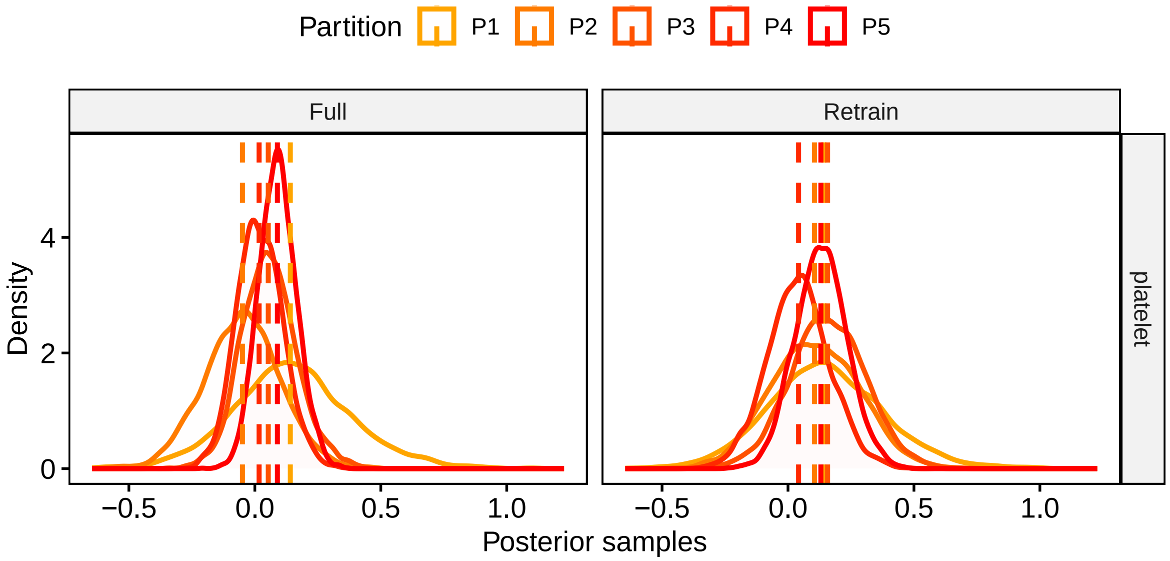
Partition
P1
P2
P3
P4
P5
Full
Retrain
platelet
Density
Posterior samples
4
2
0
−0.5
0.0
0.5
1.0

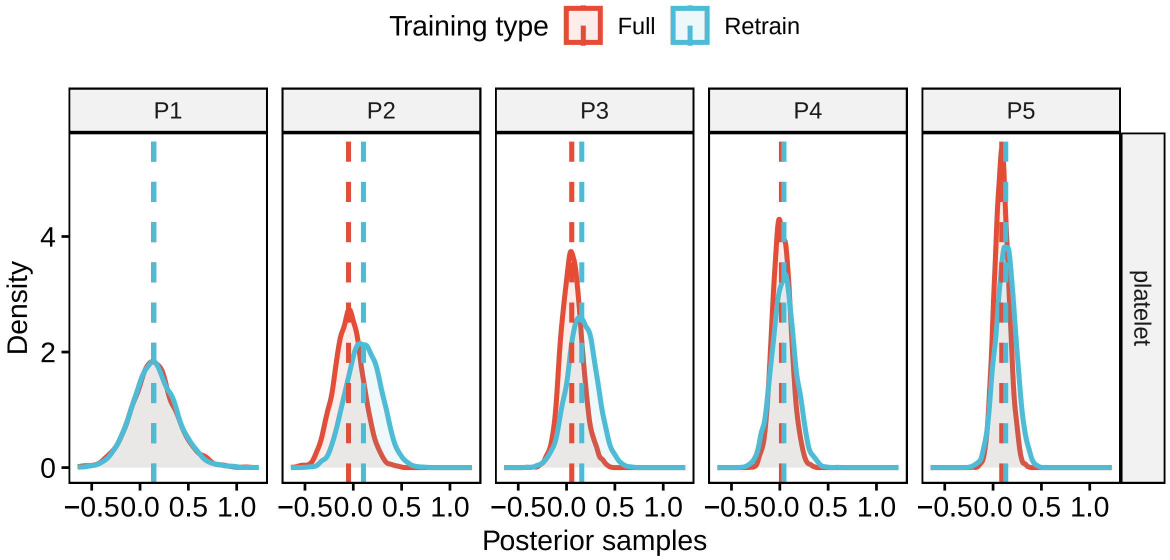
Training type
Full
Retrain
P1
P2
P3
P4
P5
platelet
Density
Posterior samples
4
2
0
−0.50.0 0.5 1.0

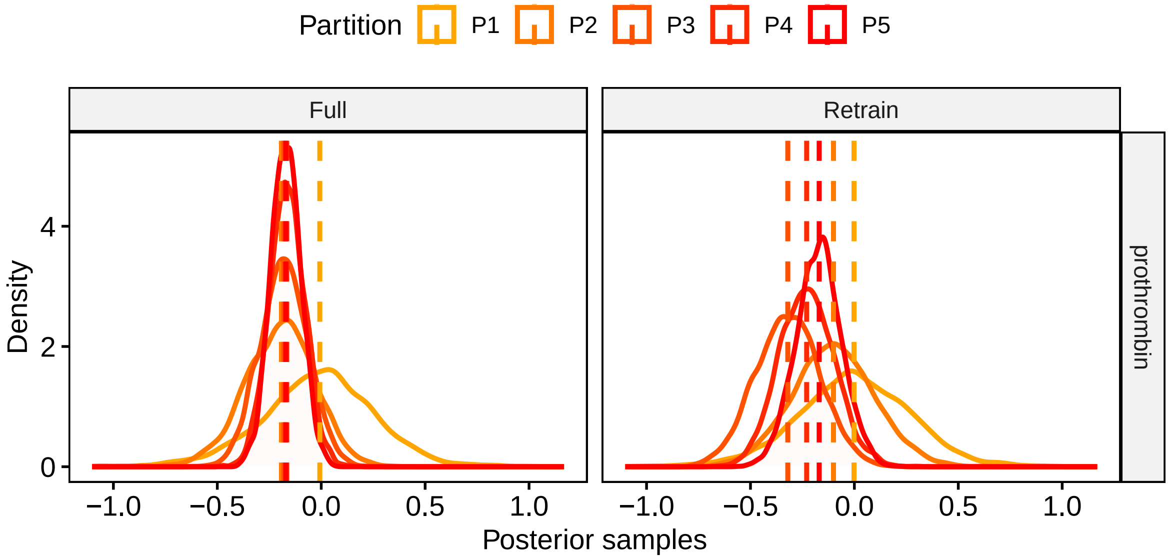
Partition
P1
P2
P3
P4
P5
Full
Retrain
prothrombin
Density
Posterior samples

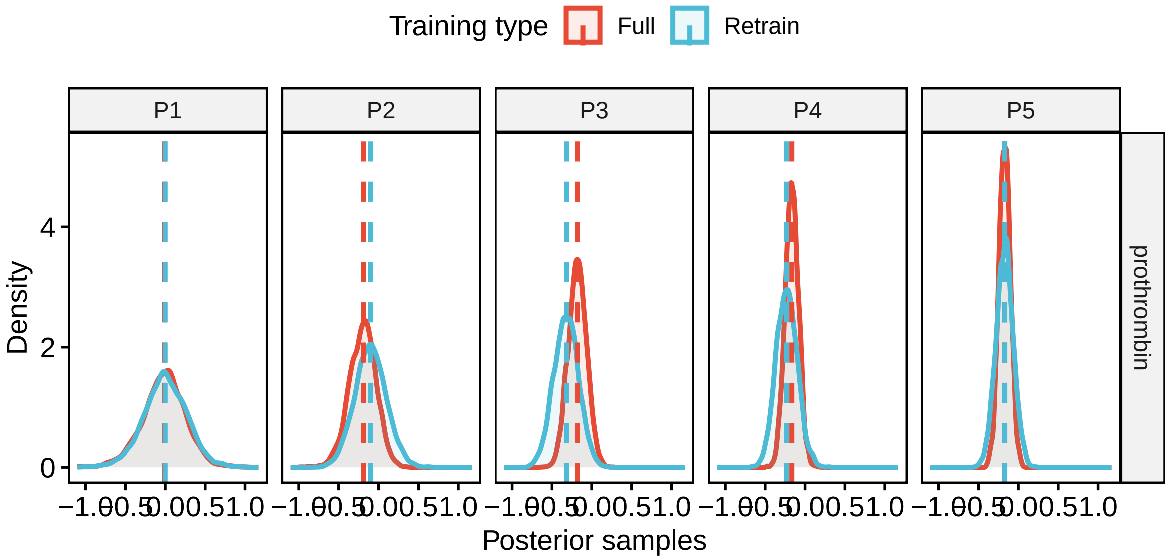
Training type
Full
Retrain
P1
P2
P3
P4
P5
prothrombin
Density
Posterior samples

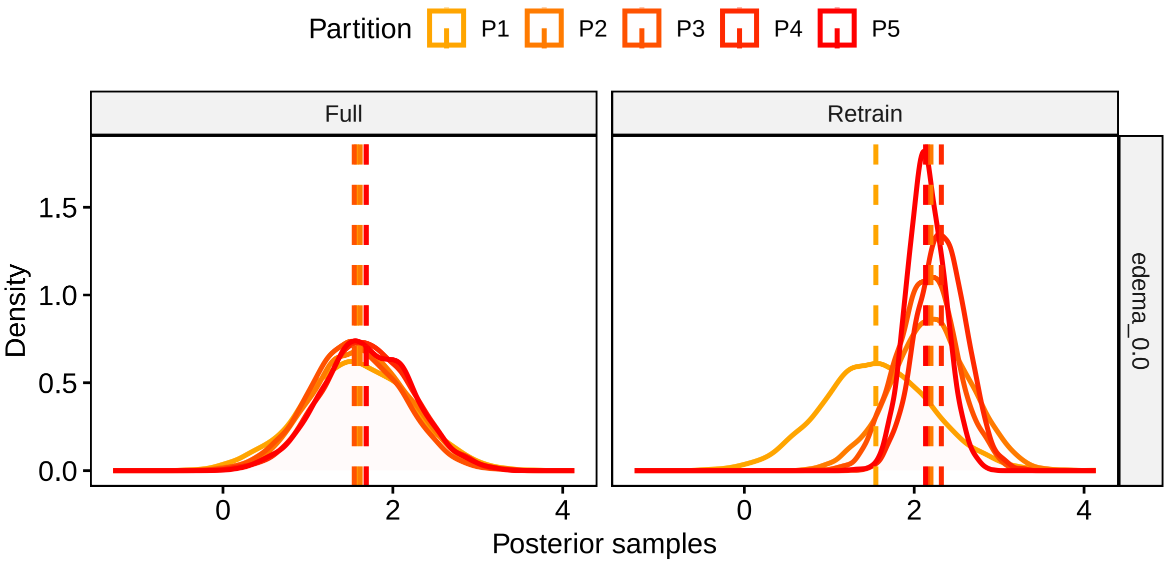
Partition P1 P2 P3 P4 P5
Full
Retrain
edema_0.0
Density
1.5
1.0
0.5
0.0
0
2
4
Posterior samples

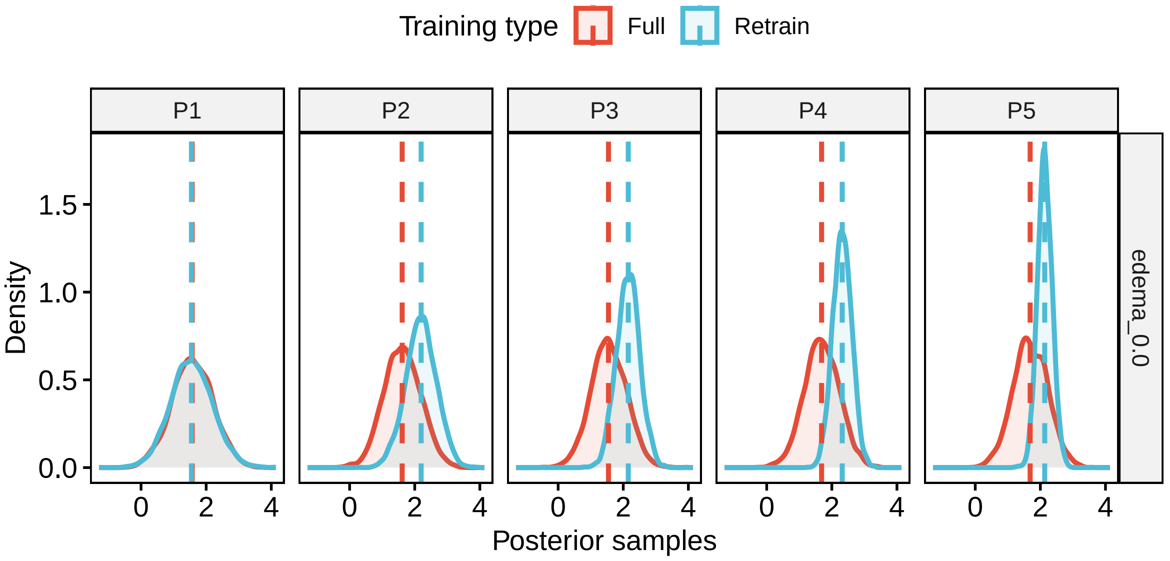
Training type Full Retrain
P1
P2
P3
P4
P5
edema_0.0
Density
1.5
1.0
0.5
0.0
0
2
4
Posterior samples

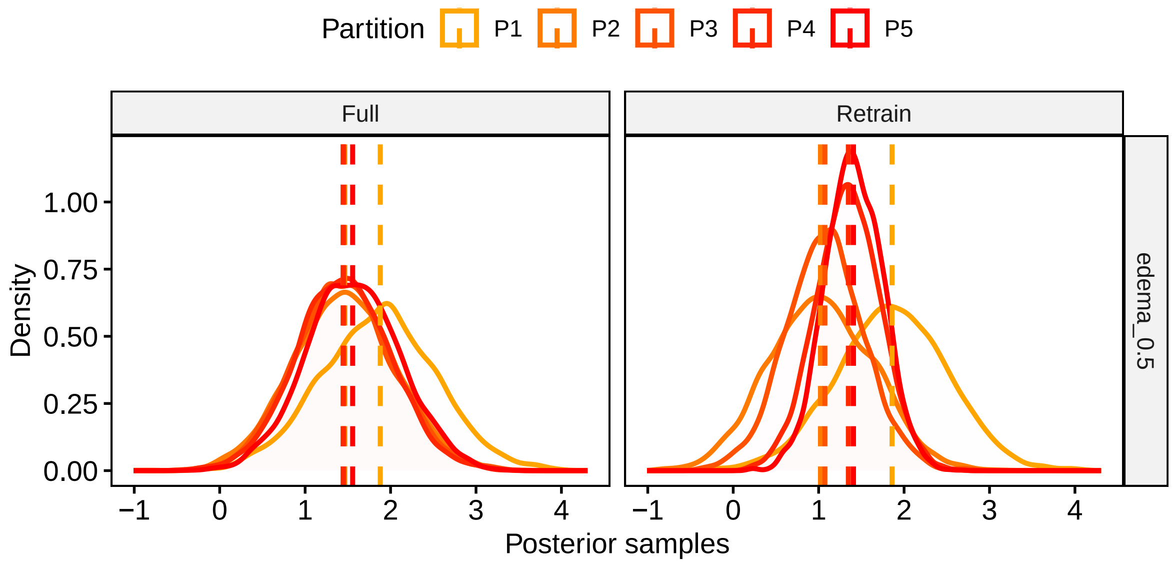

Partition
P1
P2
P3
P4
P5
Full
Retrain
edema_0.5
Density
1.00
0.75
0.50
0.25
0.00
−1
0
1
2
3
4
Posterior samples

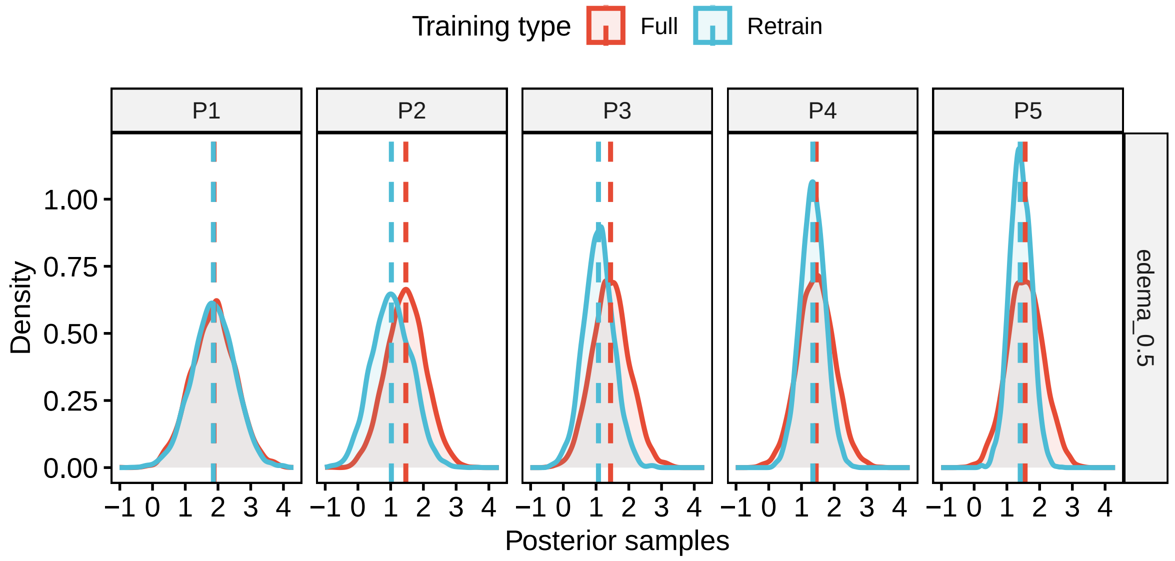

Training type
Full
Retrain
P1
P2
P3
P4
P5
edema_0.5
Density
1.00
0.75
0.50
0.25
0.00
−1 0 1 2 3 4
Posterior samples

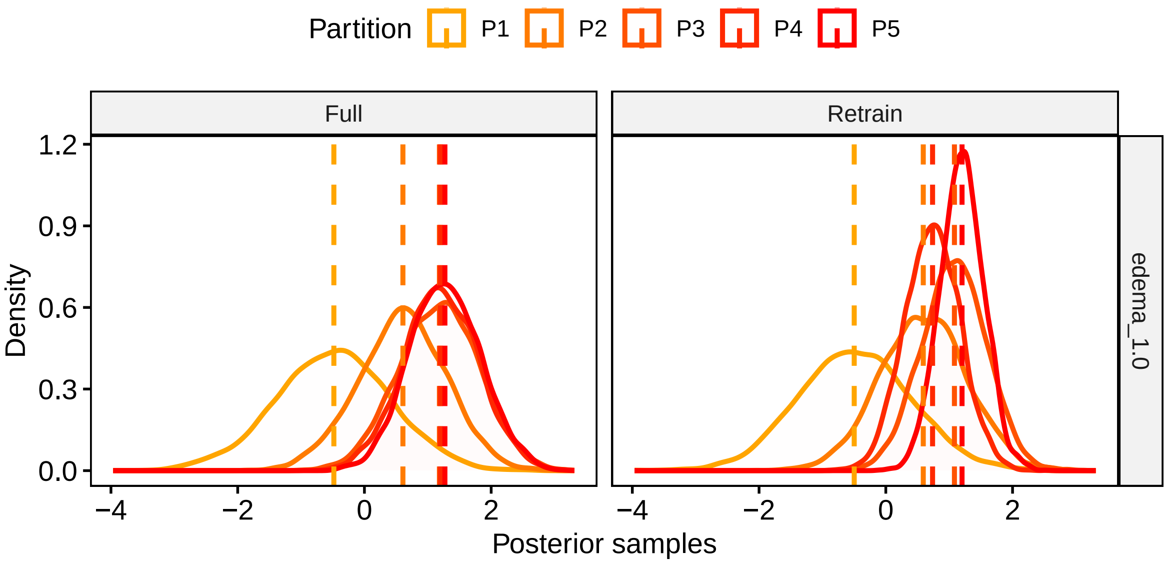
Partition P1 P2 P3 P4 P5
Full
Retrain
edema_1.0
Density
Posterior samples

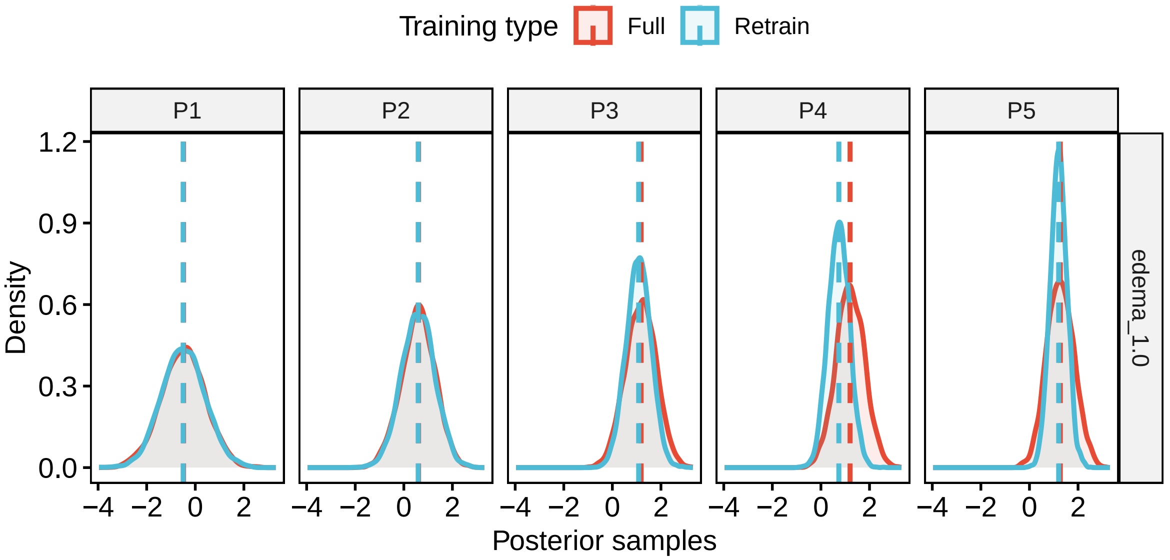
Training type Full Retrain
P1
P2
P3
P4
P5
edema_1.0
Density
Posterior samples

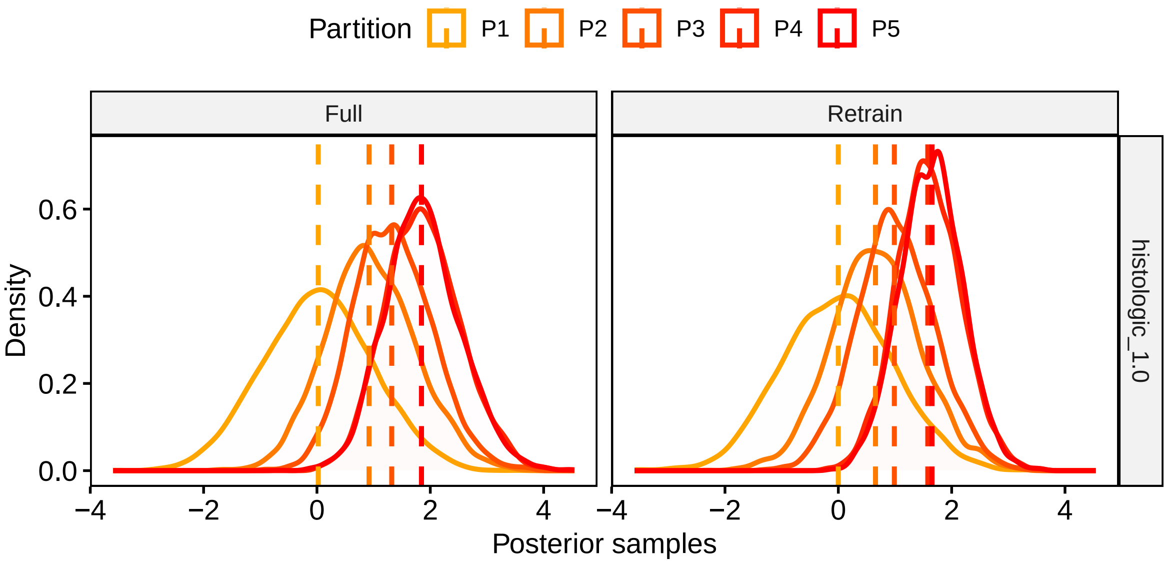

Partition
P1
P2
P3
P4
P5
Full
Retrain
histologic_1.0
Density
Posterior samples

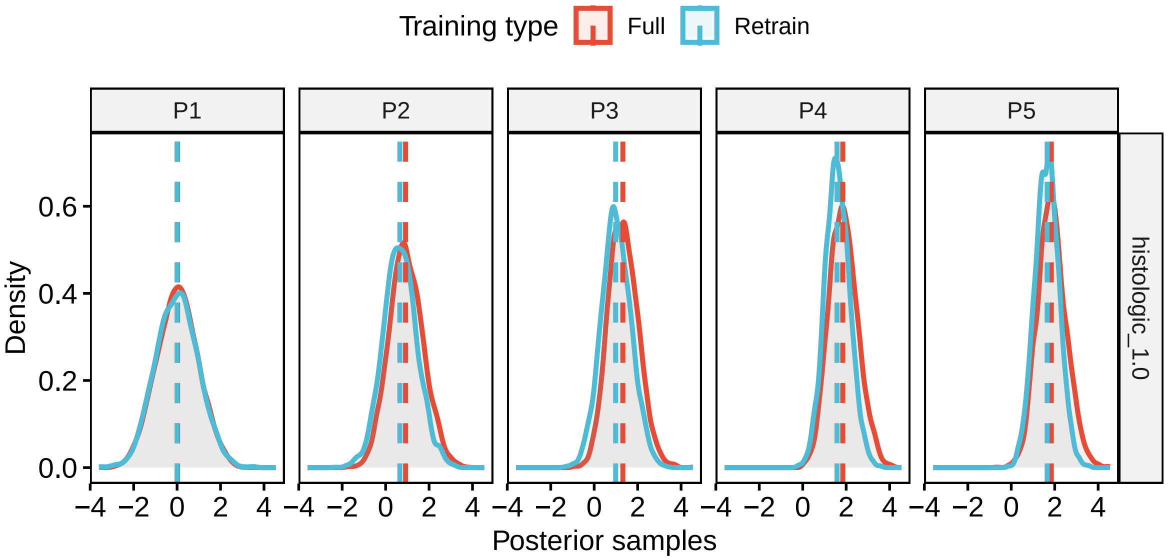

Training type
Full
Retrain
P1
P2
P3
P4
P5
histologic_1.0
Density
Posterior samples

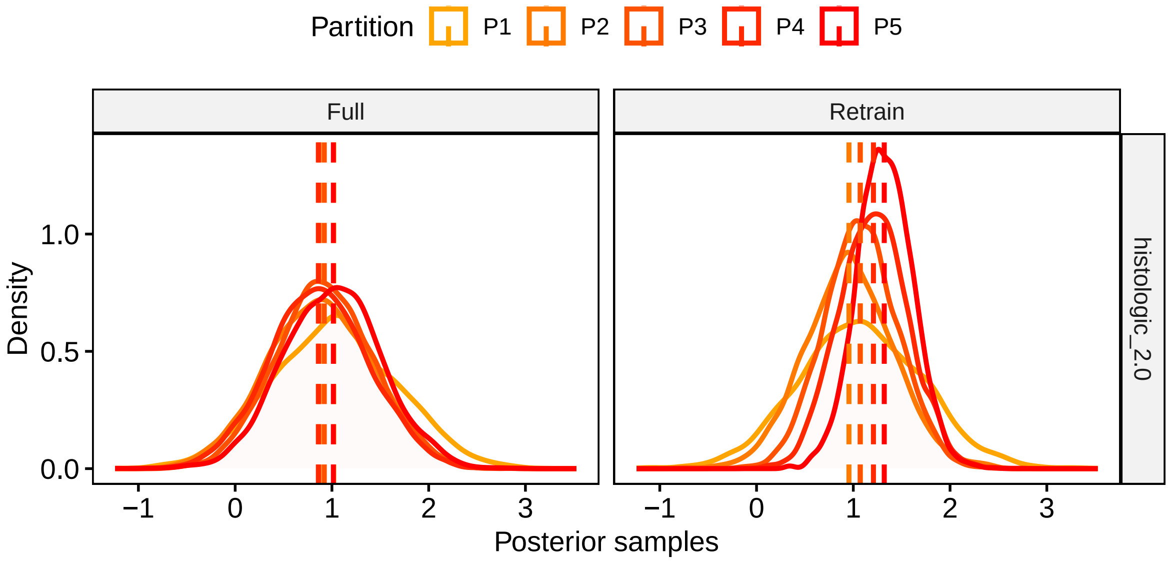

Partition
P1
P2
P3
P4
P5
Full
Retrain
histologic_2.0
Density
1.0
0.5
0.0
−1
0
1
2
3
Posterior samples

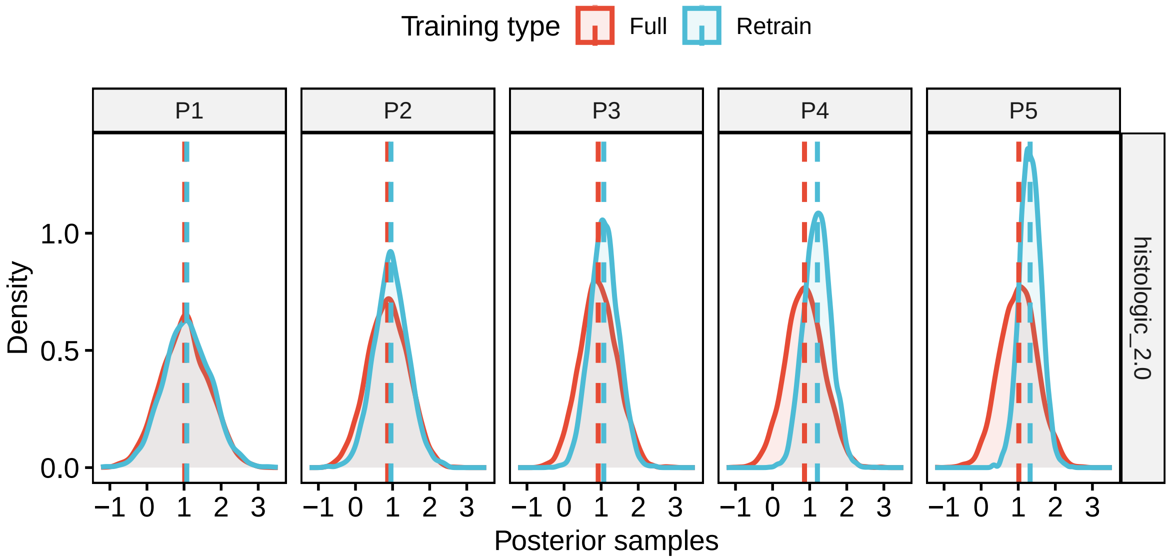

Training type
Full
Retrain
P1
P2
P3
P4
P5
histologic_2.0
Density
1.0
0.5
0.0
−1 0 1 2 3
Posterior samples

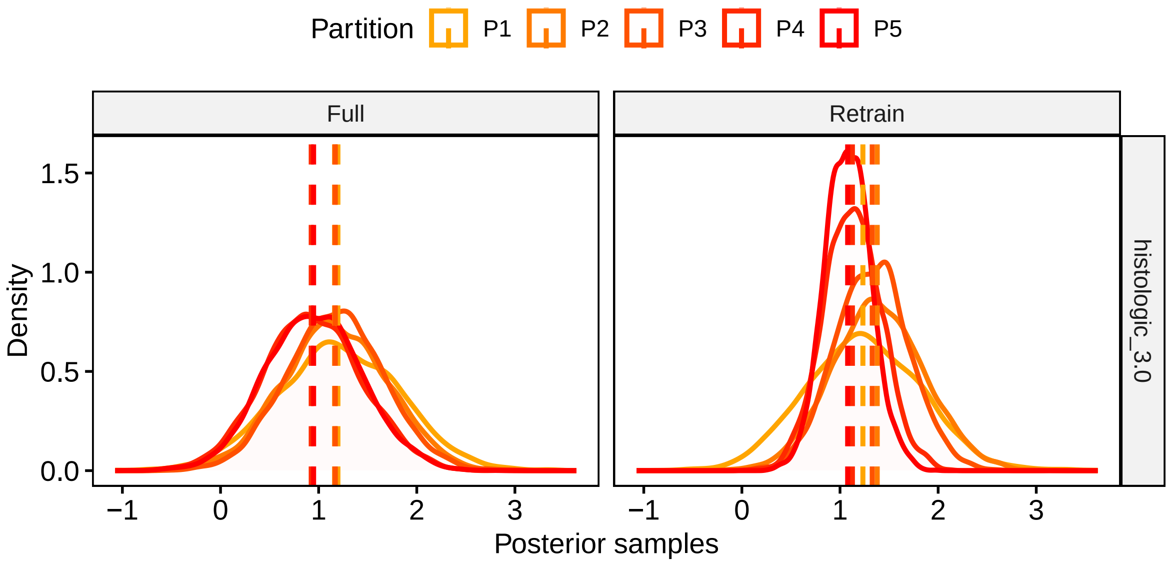
Partition
P1
P2
P3
P4
P5
Full
Retrain
histologic_3.0
Density
Posterior samples

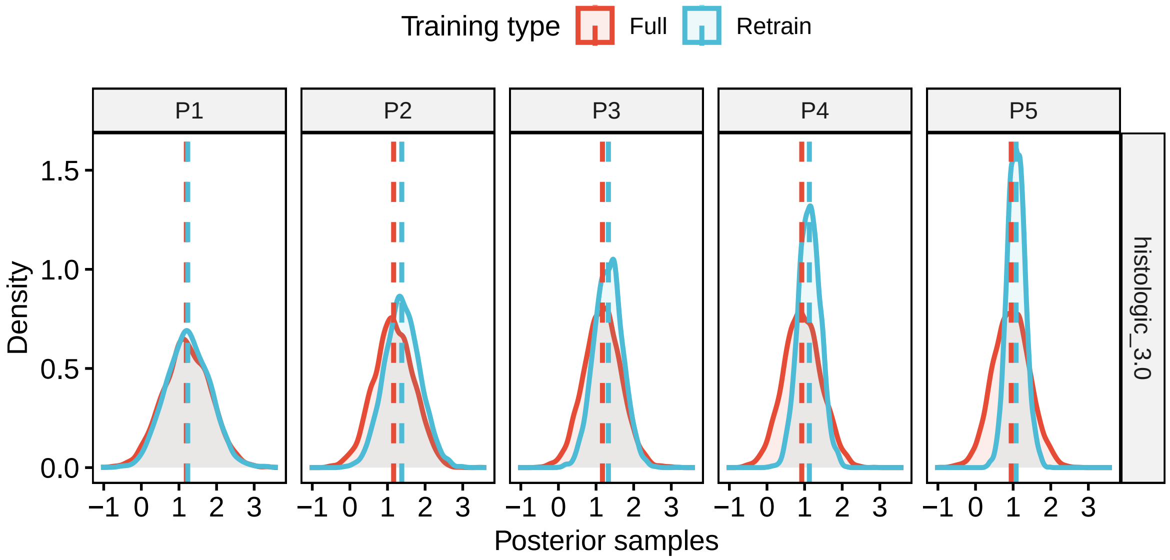
Training type
Full
Retrain
P1
P2
P3
P4
P5
histologic_3.0
Density
Posterior samples

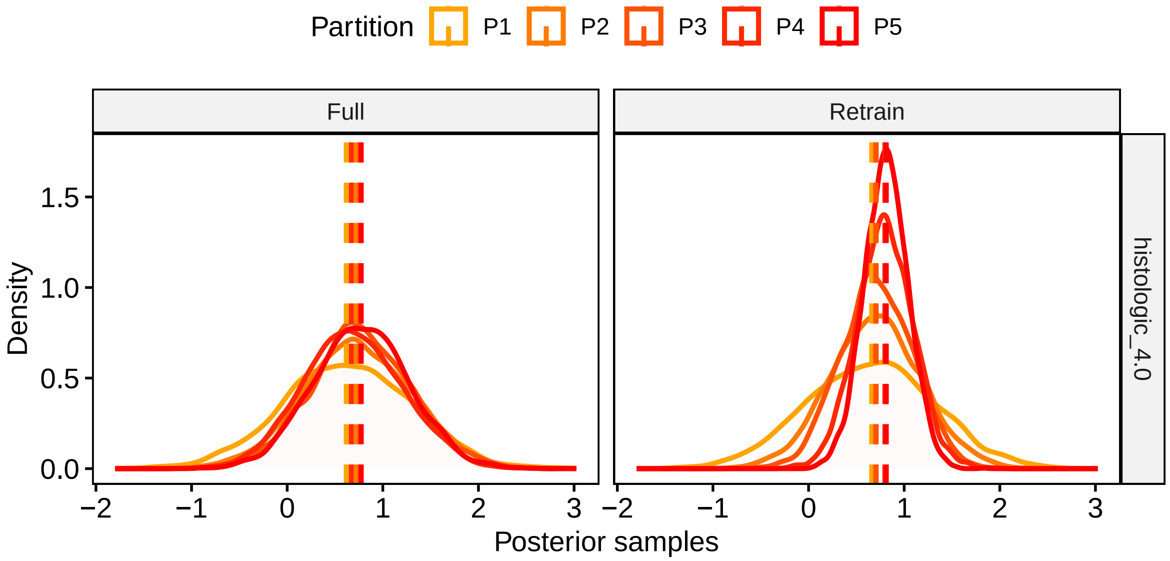

Partition
P1
P2
P3
P4
P5
Full
Retrain
histologic_4.0
Density
1.5
1.0
0.5
0.0
−2
−1
0
1
2
3
Posterior samples

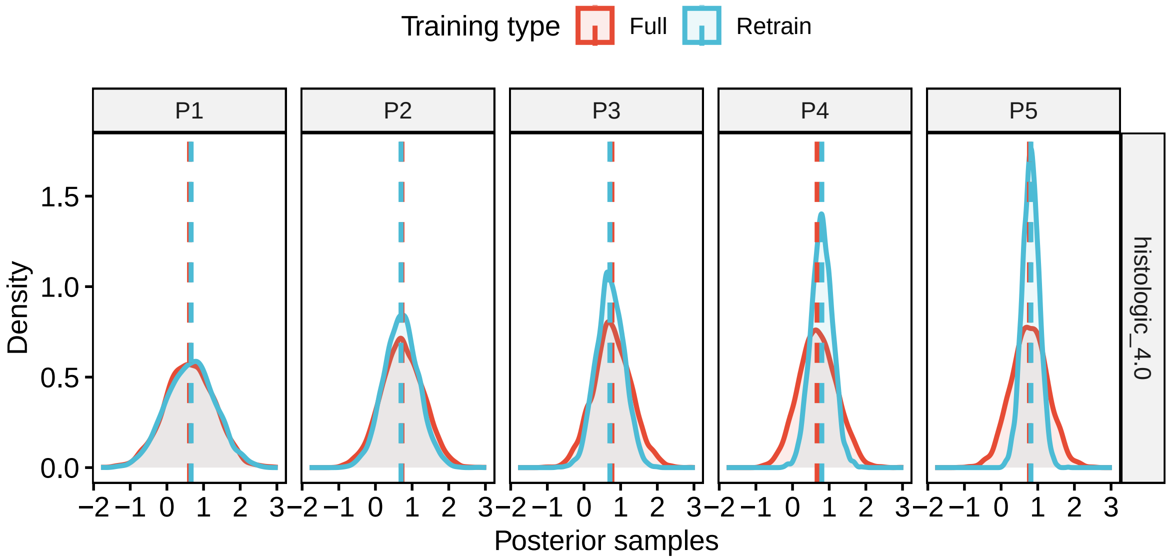

Training type
Full
Retrain
P1
P2
P3
P4
P5
histologic_4.0
Density
1.5
1.0
0.5
0.0
−2 −1 0 1 2 3
Posterior samples

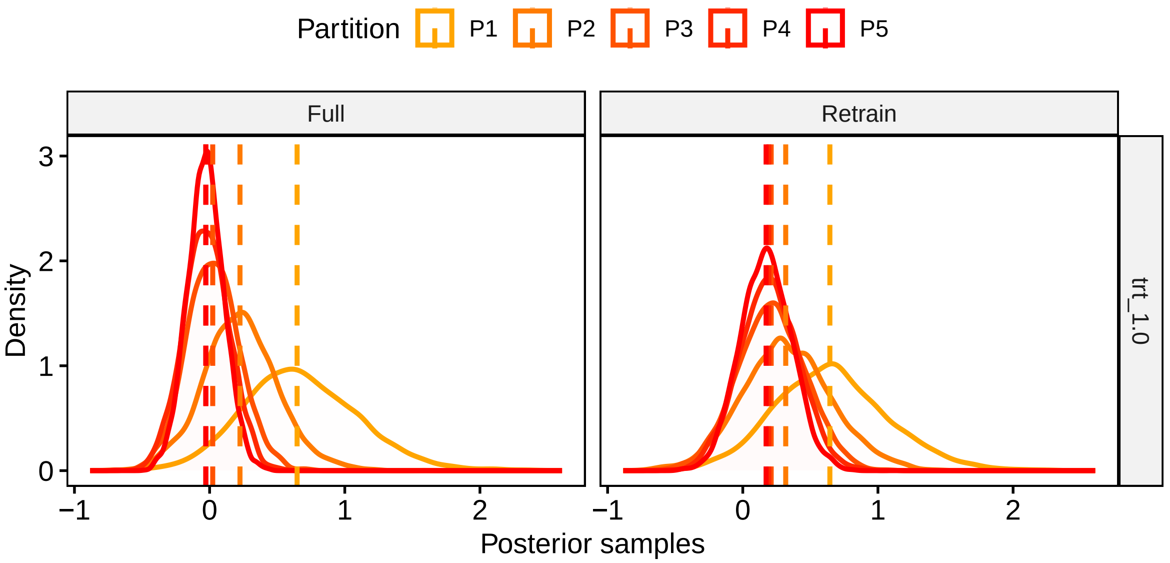
Partition
P1
P2
P3
P4
P5
Full
Retrain
trt_1.0
Density
Posterior samples

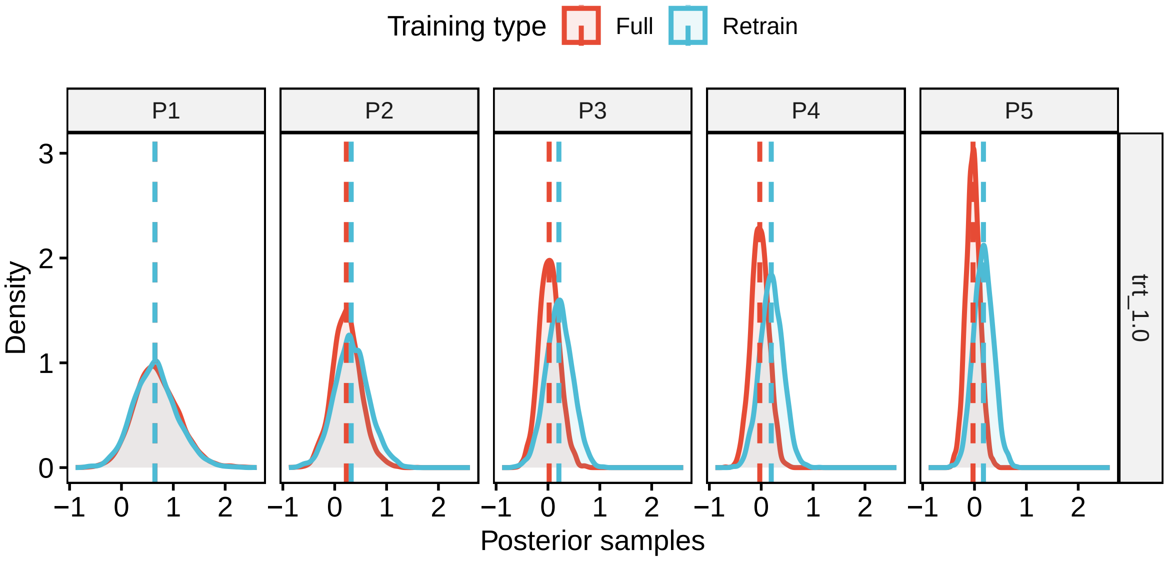
Training type
Full
Retrain
P1
P2
P3
P4
P5
trt_1.0
Density
Posterior samples

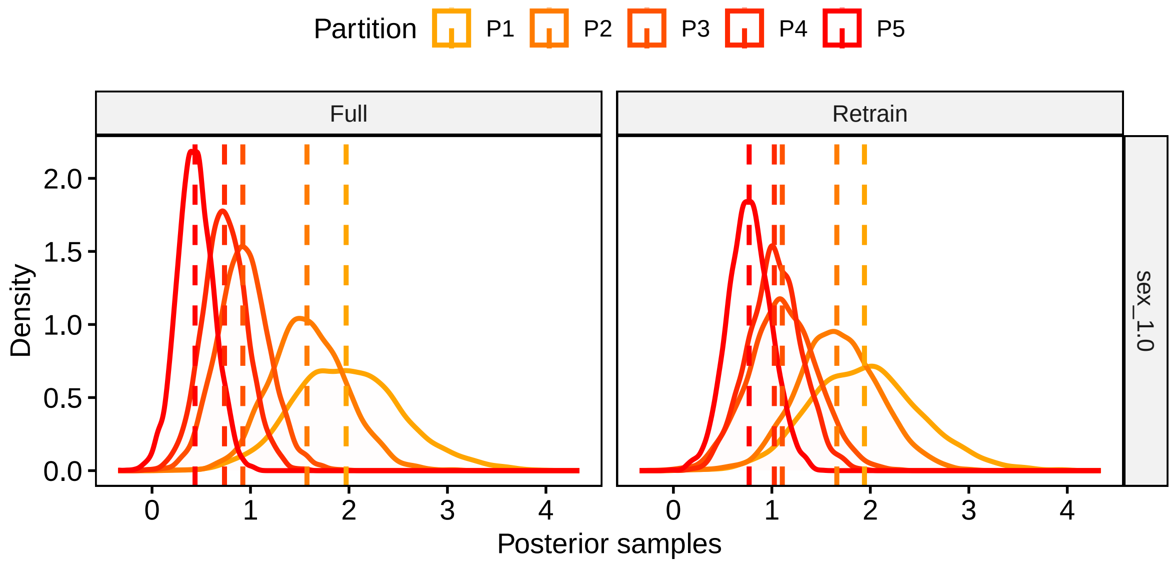
Partition
P1
P2
P3
P4
P5
Full
Retrain
sex_1.0
Density
0.0
0.5
1.0
1.5
2.0
0
1
2
3
4
Posterior samples

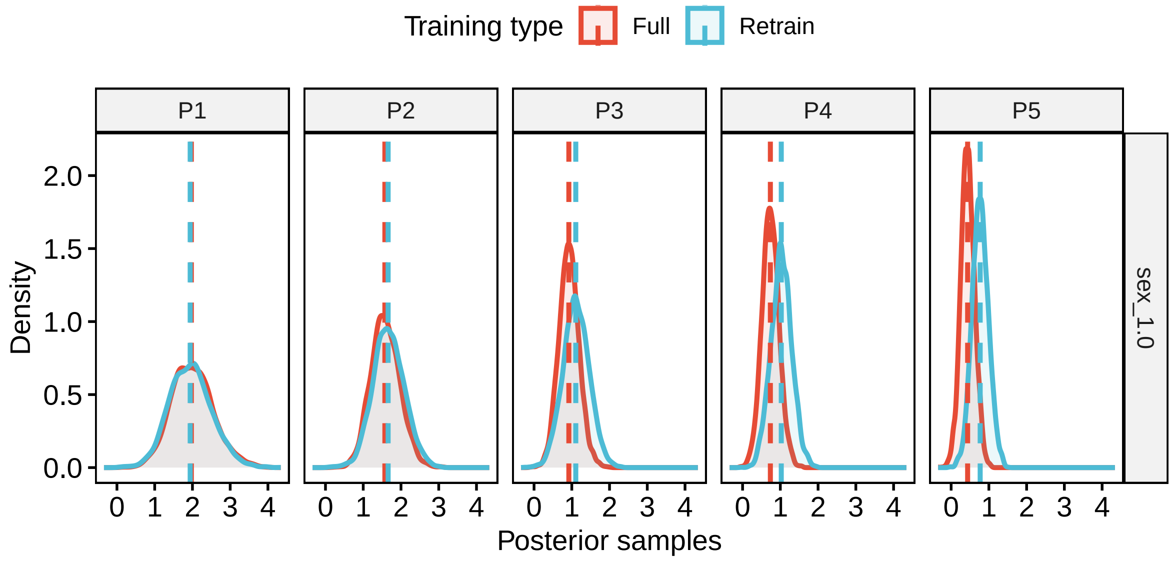
Training type
Full
Retrain
P1
P2
P3
P4
P5
sex_1.0
Density
0.0
0.5
1.0
1.5
2.0
0
1
2
3
4
Posterior samples

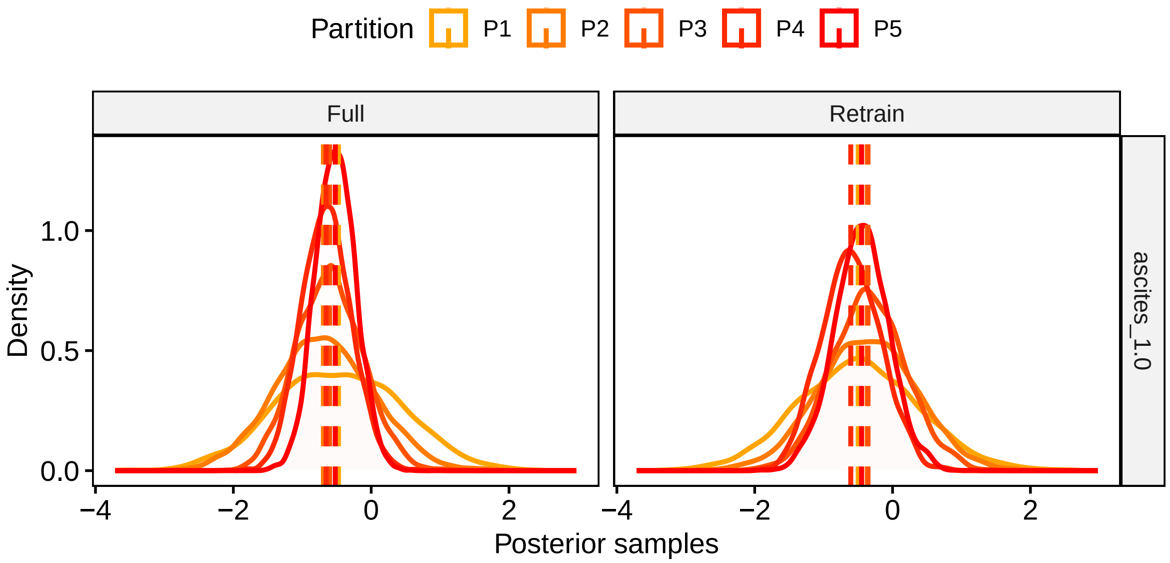
Partition
P1
P2
P3
P4
P5
Full
Retrain
ascites_1.0
Density
1.0
0.5
0.0
−4
−2
0
2
Posterior samples

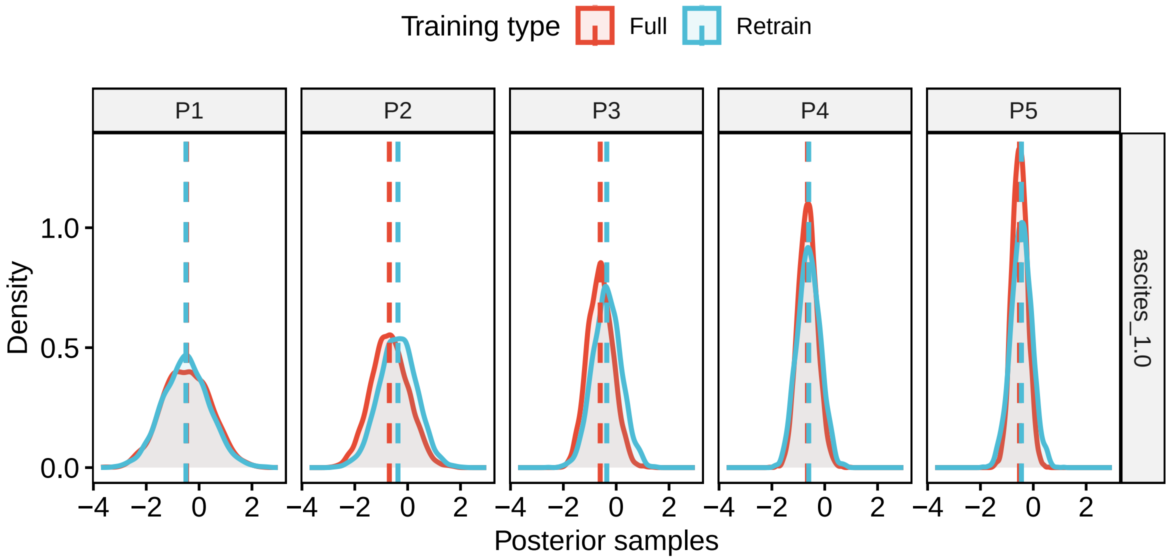
Training type
Full
Retrain
P1
P2
P3
P4
P5
ascites_1.0
Density
1.0
0.5
0.0
−4
−2
0
2
Posterior samples

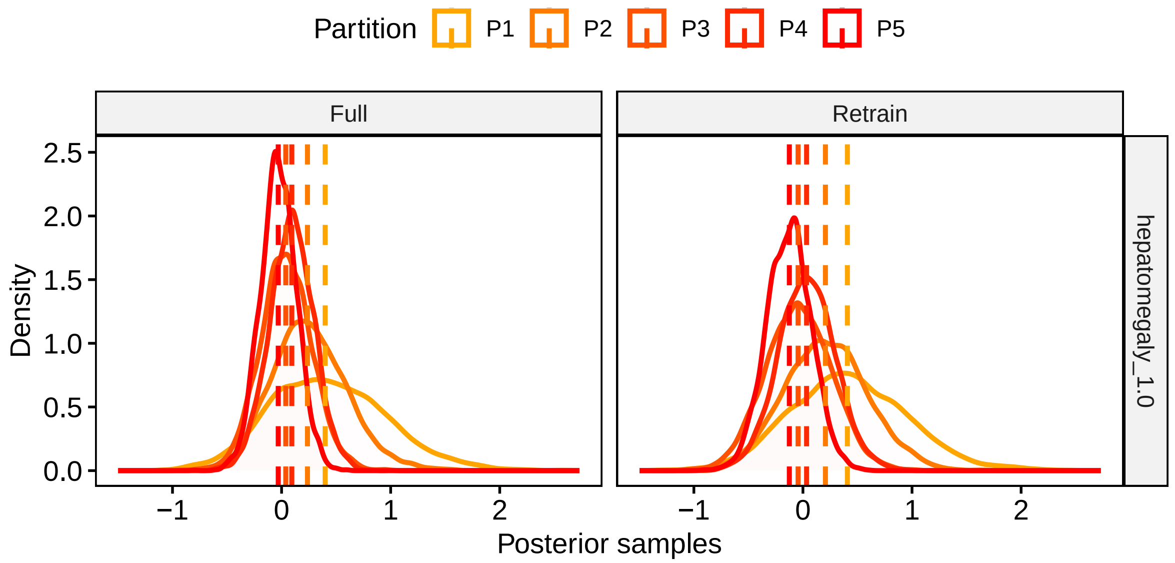
Partition
P1
P2
P3
P4
P5
Full
Retrain
hepatomegaly_1.0
Density
Posterior samples
2.5
2.0
1.5
1.0
0.5
0.0
−1
0
1
2

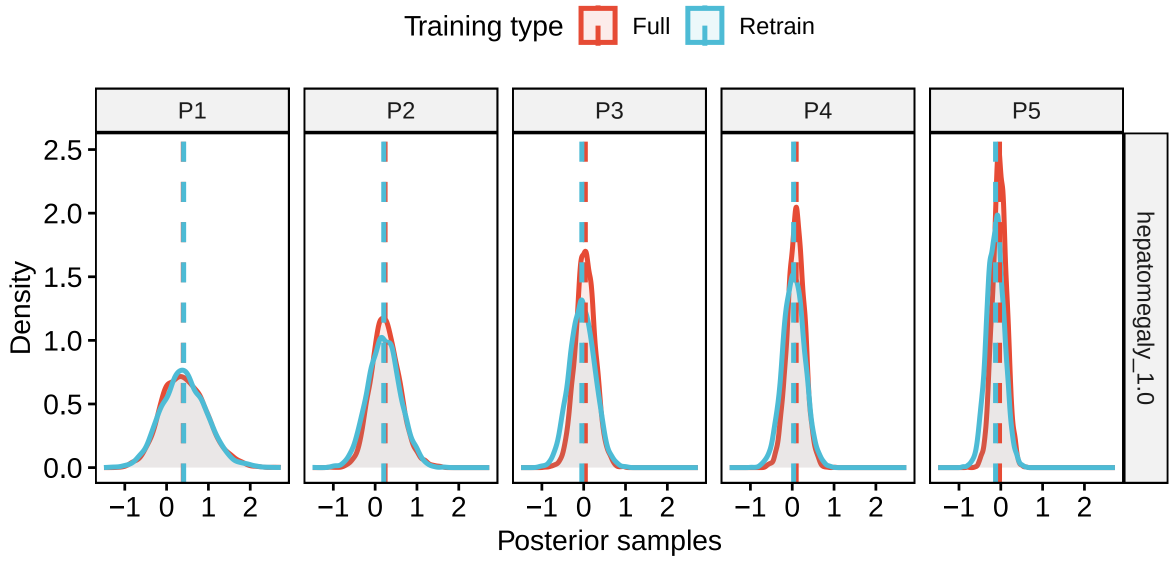
Training type
Full
Retrain
P1
P2
P3
P4
P5
hepatomegaly_1.0
Density
Posterior samples
2.5
2.0
1.5
1.0
0.5
0.0
−1
0
1
2

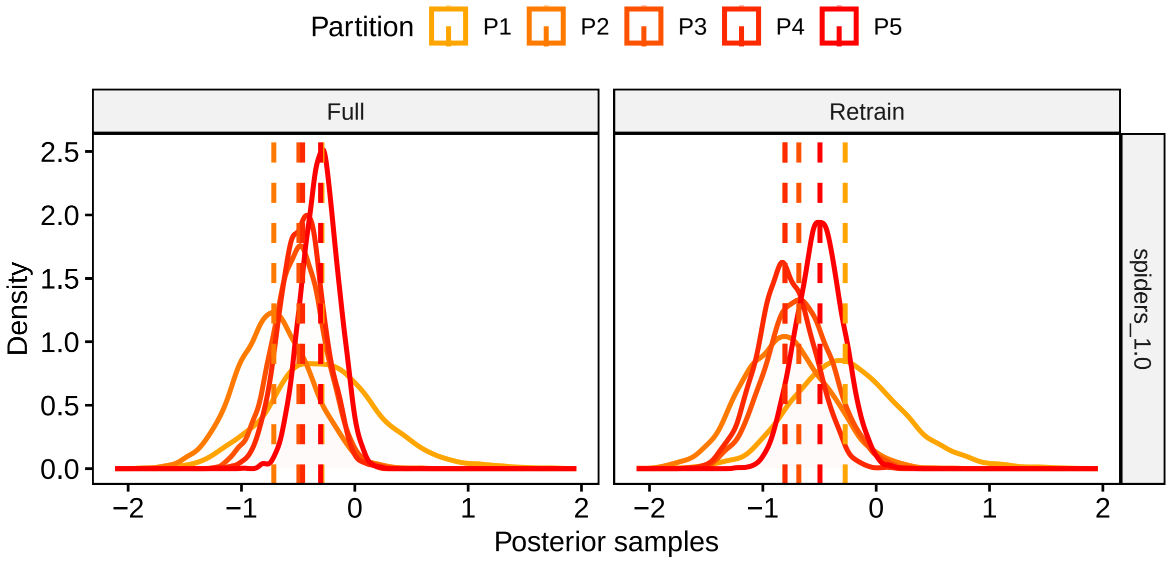
Partition
P1
P2
P3
P4
P5
Full
Retrain
spiders_1.0
Density
Posterior samples
2.5
2.0
1.5
1.0
0.5
0.0
−2
−1
0
1
2

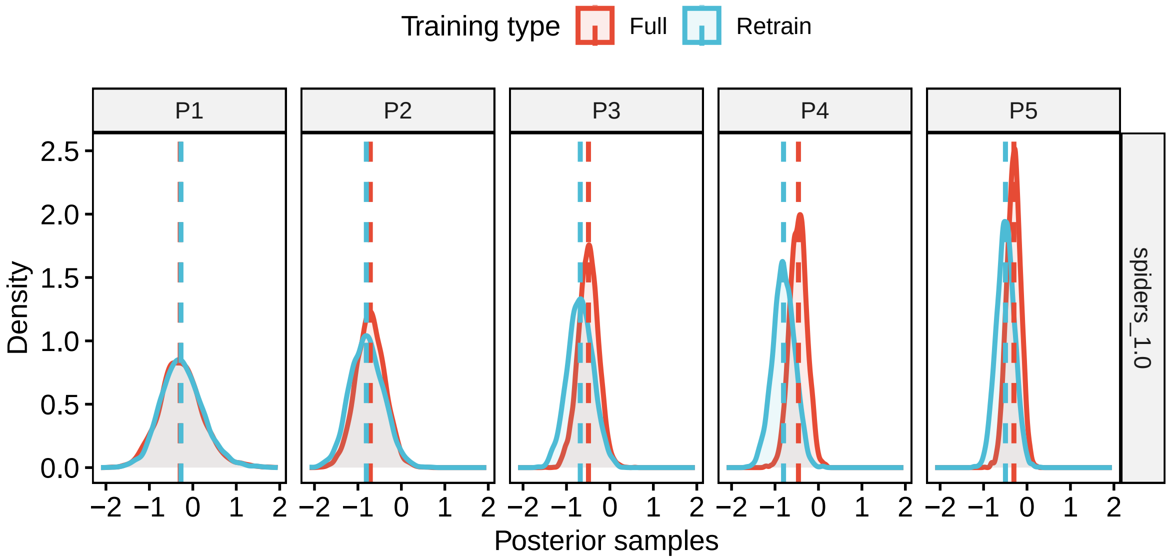
Training type
Full
Retrain
P1
P2
P3
P4
P5
spiders_1.0
Density
Posterior samples
2.5
2.0
1.5
1.0
0.5
0.0
−2
−1
0
1
2

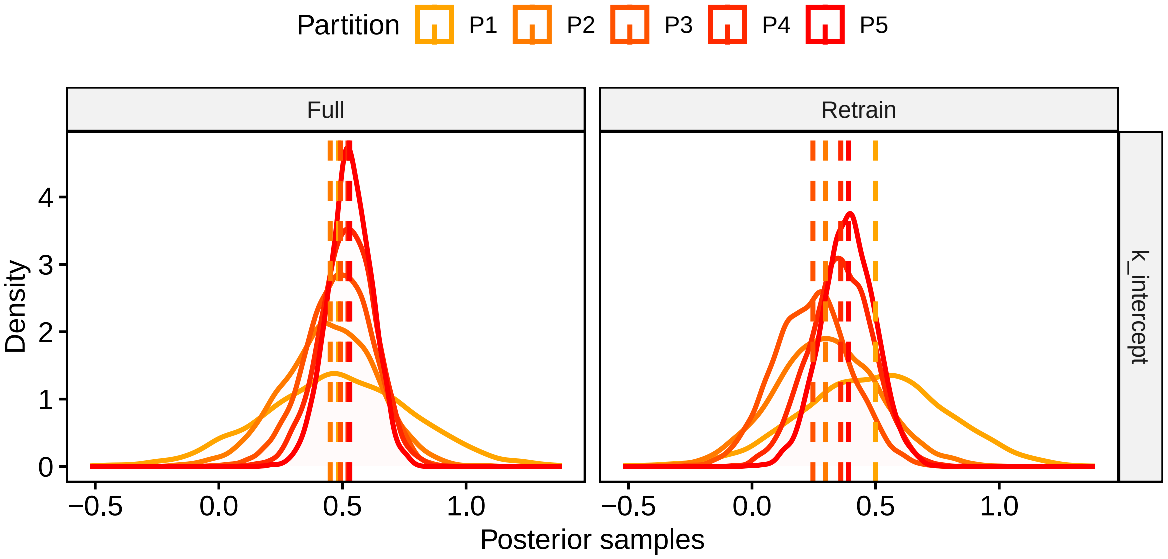
Partition
P1
P2
P3
P4
P5
Full
Retrain
k_intercept
Density
Posterior samples
−0.5
0.0
0.5
1.0
0
1
2
3
4

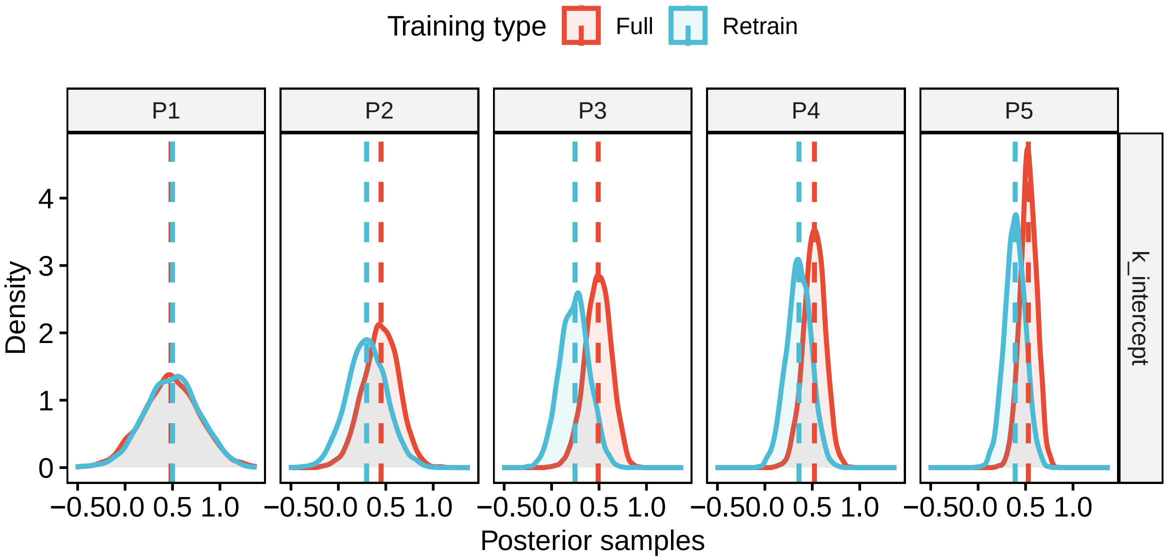
Training type
Full
Retrain
P1
P2
P3
P4
P5
k_intercept
Density
Posterior samples
−0.50.0 0.5 1.0
0
1
2
3
4

ACTG

```
pbc_vars <- data %>% filter(experiment == 'actg') %>% select(var) %>% unique(.)

for(var_idx in unique(pbc_vars$var)){
  p1 <- plot_density(data, 'actg', 'pm_wb', var_idx)
  plot(p1)

  p <- plot_density_2(data, 'actg', 'pm_wb', var_idx)
  plot(p)
}
```

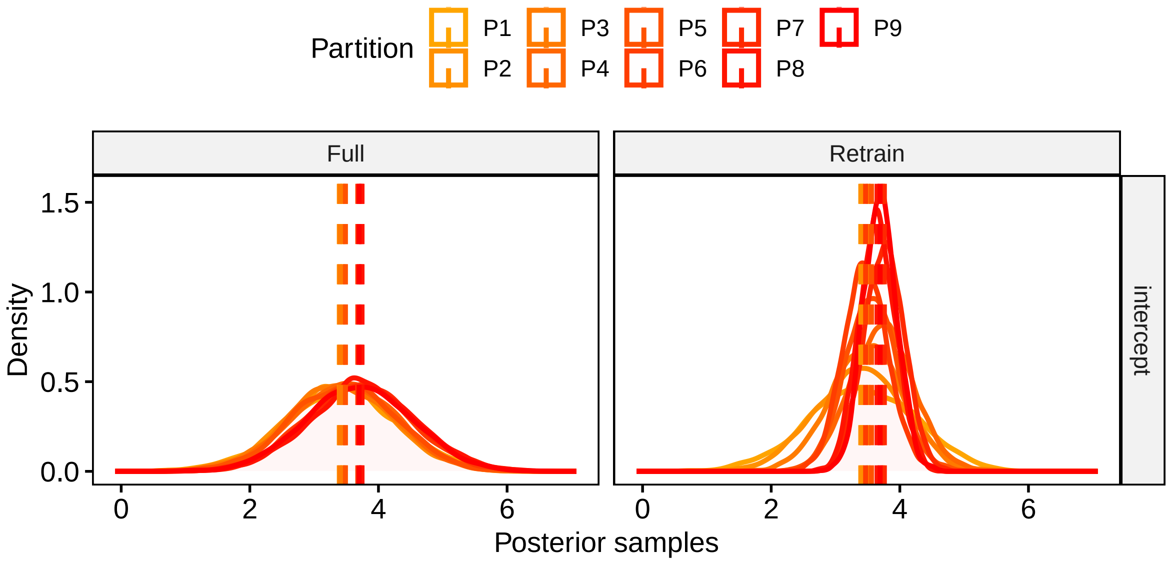

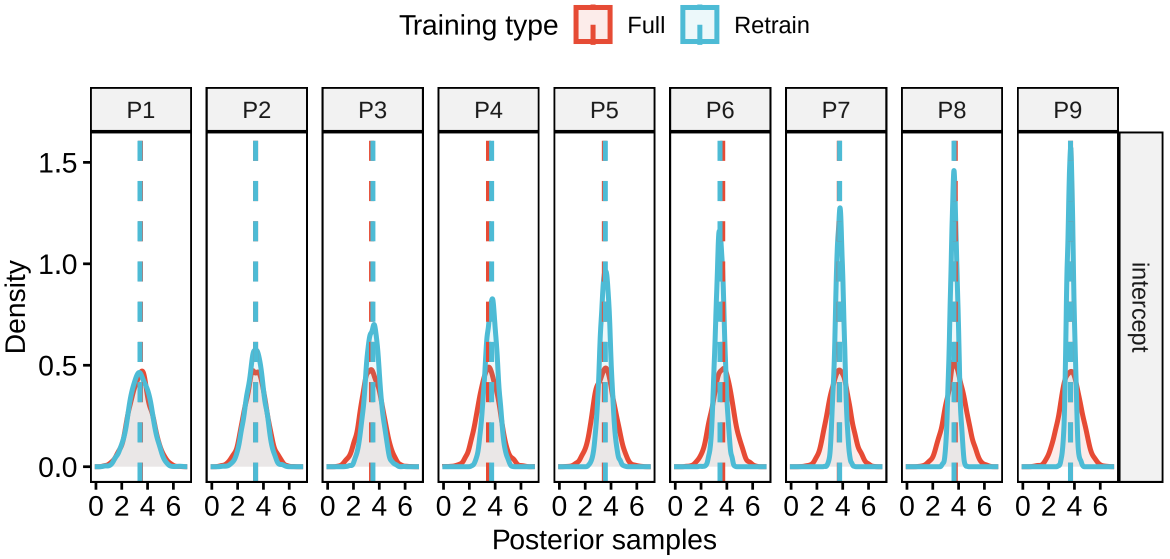
Training type
Full
Retrain
P1
P2
P3
P4
P5
P6
P7
P8
P9
intercept
Density
Posterior samples

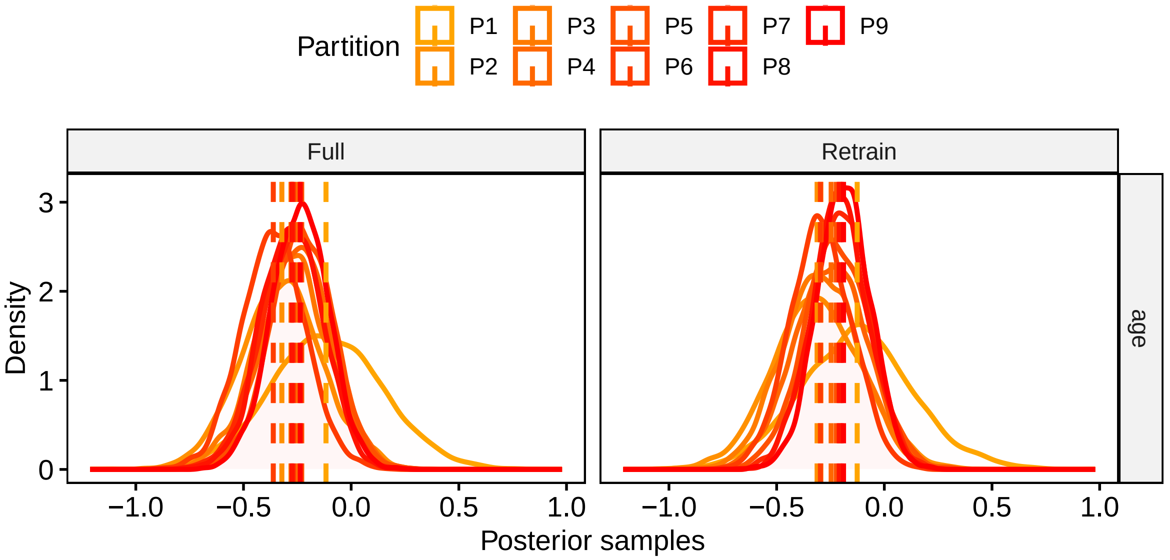
Partition
P1
P2
P3
P4
P5
P6
P7
P8
P9
Full
Retrain
age
Density
Posterior samples

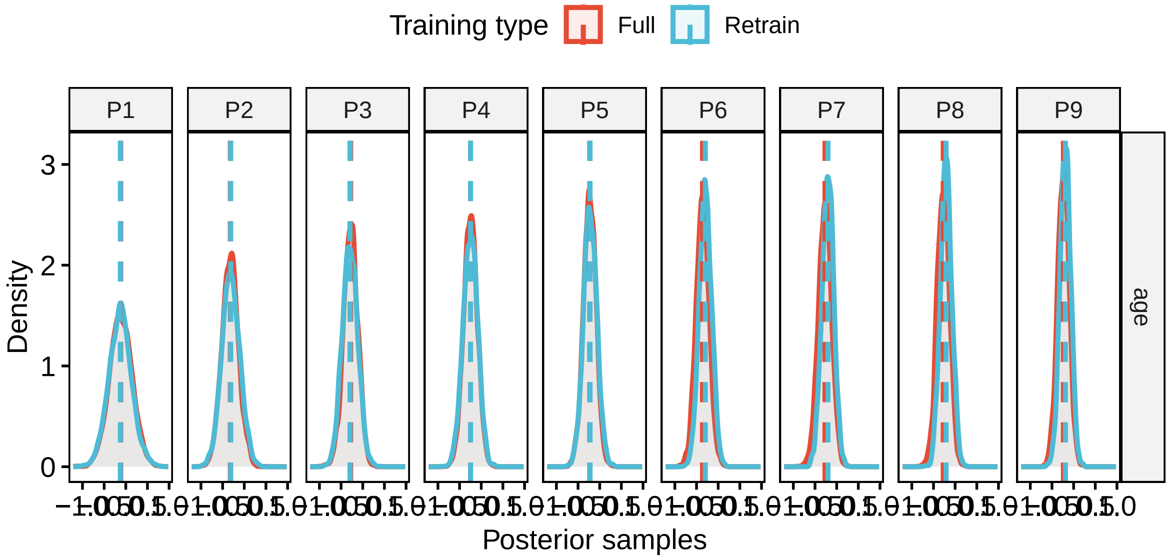
Training type
Full
Retrain
P1
P2
P3
P4
P5
P6
P7
P8
P9
age
Density
Posterior samples

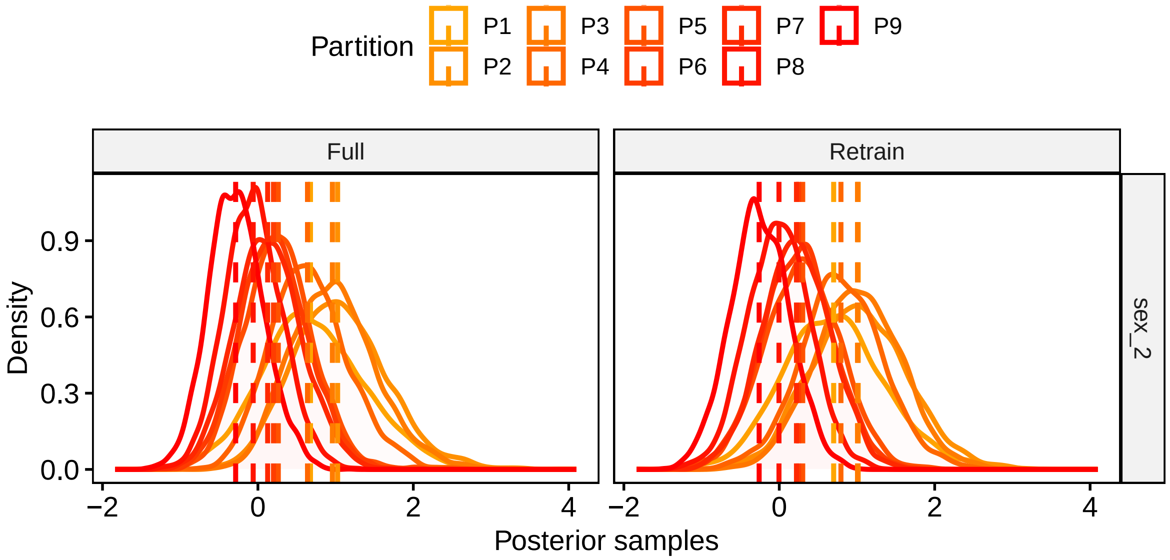
Partition
P1
P2
P3
P4
P5
P6
P7
P8
P9
Full
Retrain
sex_2
Density
Posterior samples

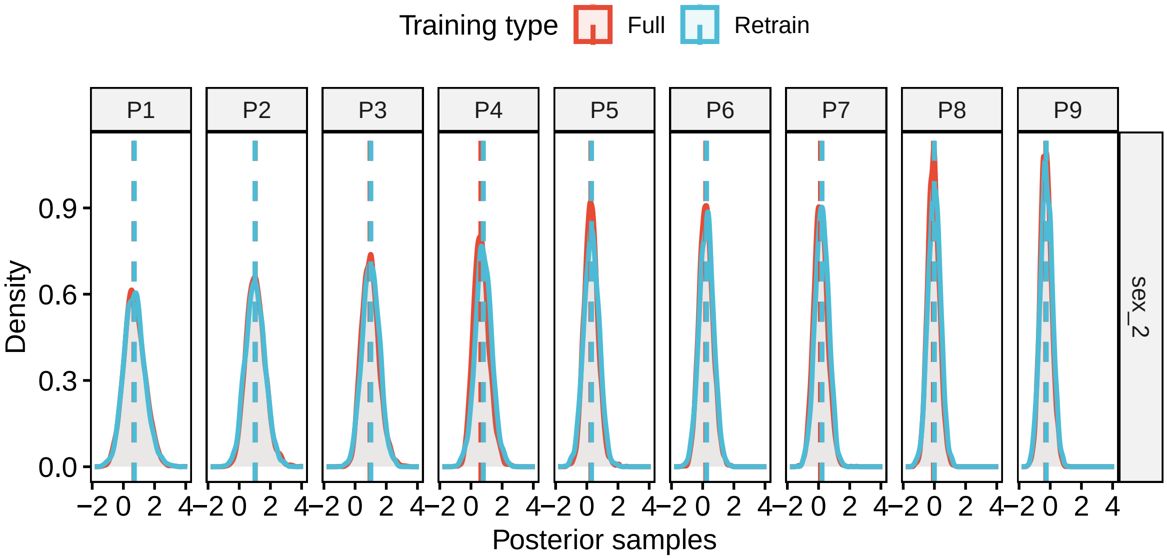
Training type
Full
Retrain
P1
P2
P3
P4
P5
P6
P7
P8
P9
sex_2
Density
0.9
0.6
0.3
0.0
−2 0 2 4
Posterior samples

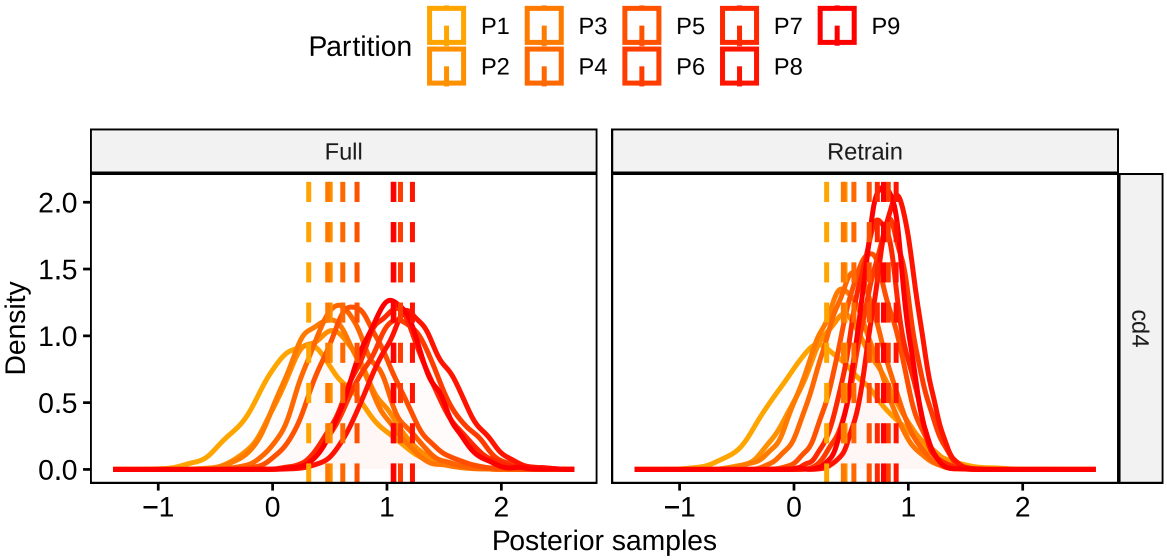
Partition
P1
P2
P3
P4
P5
P6
P7
P8
P9
Full
Retrain
cd4
Density
2.0
1.5
1.0
0.5
0.0
−1
0
1
2
Posterior samples

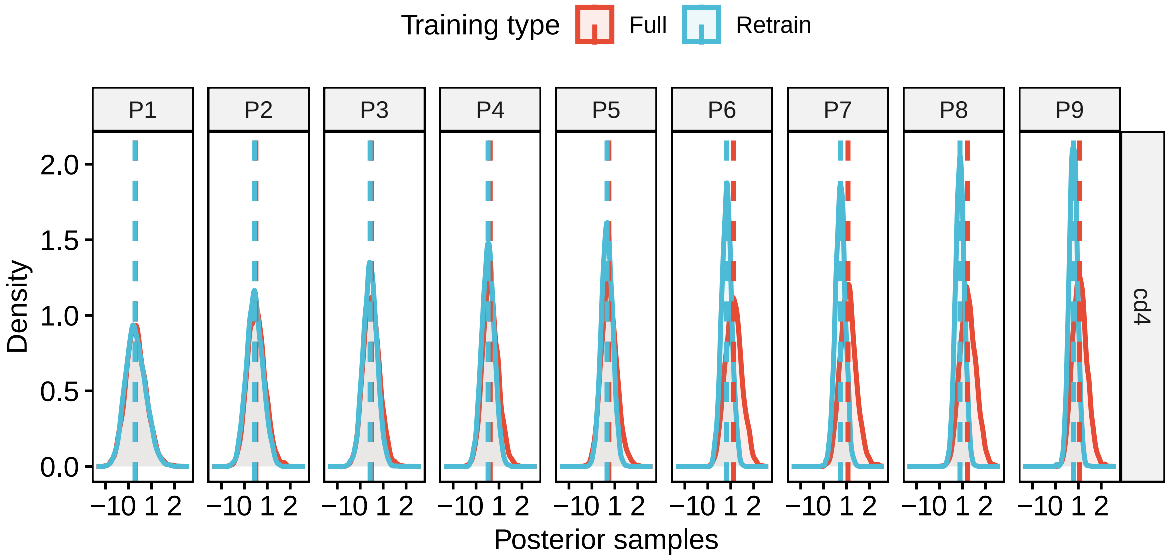
Training type
Full
Retrain
P1
P2
P3
P4
P5
P6
P7
P8
P9
Density
2.0
1.5
1.0
0.5
0.0
cd4
−10 1 2
Posterior samples

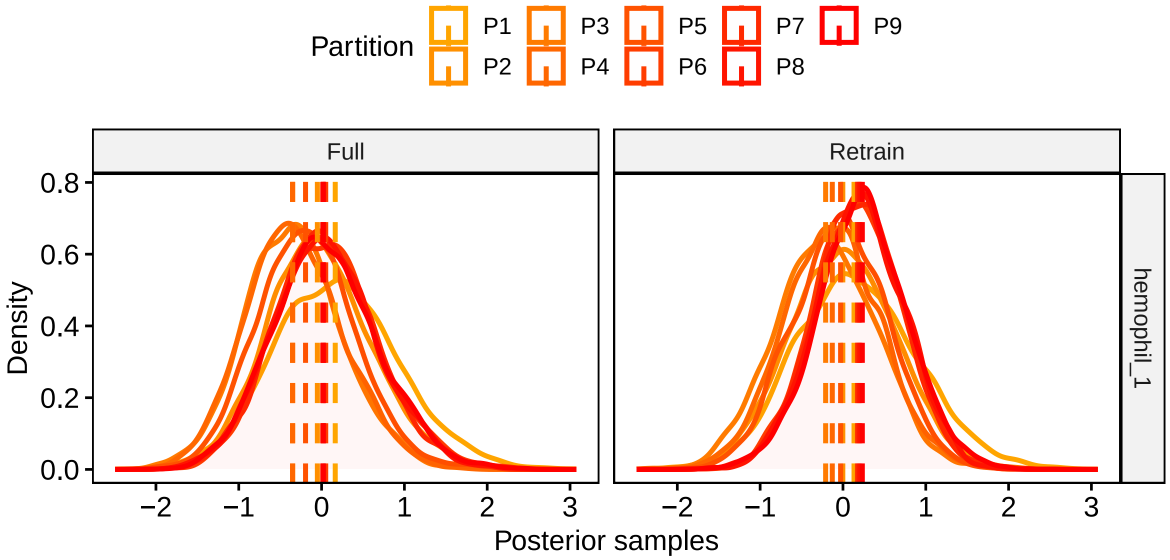
Partition
P1
P2
P3
P4
P5
P6
P7
P8
P9
Full
Retrain
Density
0.8
0.6
0.4
0.2
0.0
hemophil_1
−2
−1
0
1
2
3
Posterior samples

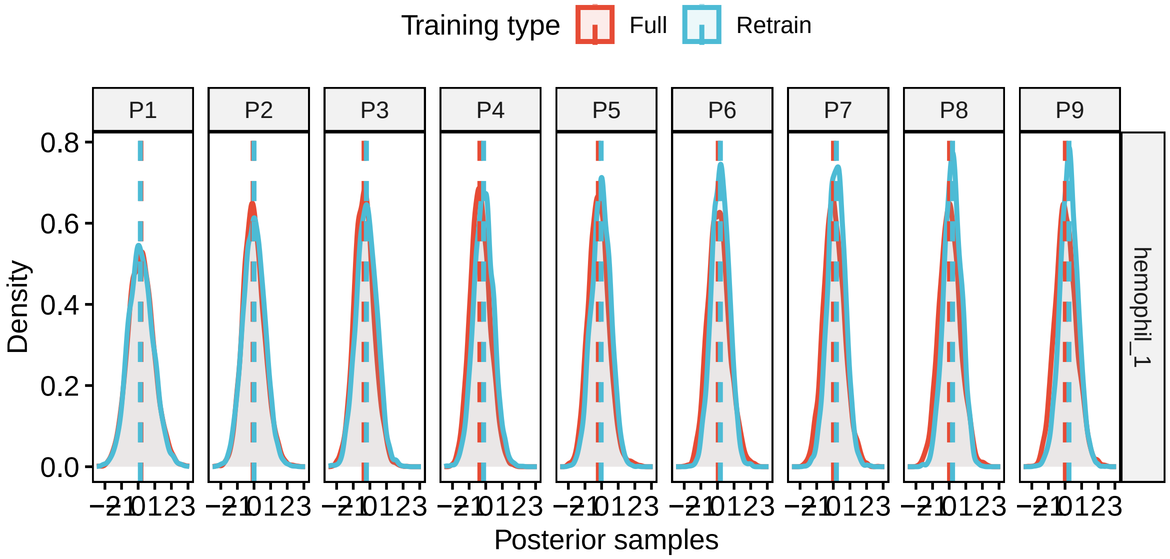
Training type
Full
Retrain
P1
P2
P3
P4
P5
P6
P7
P8
P9
hemophil_1
Density
Posterior samples

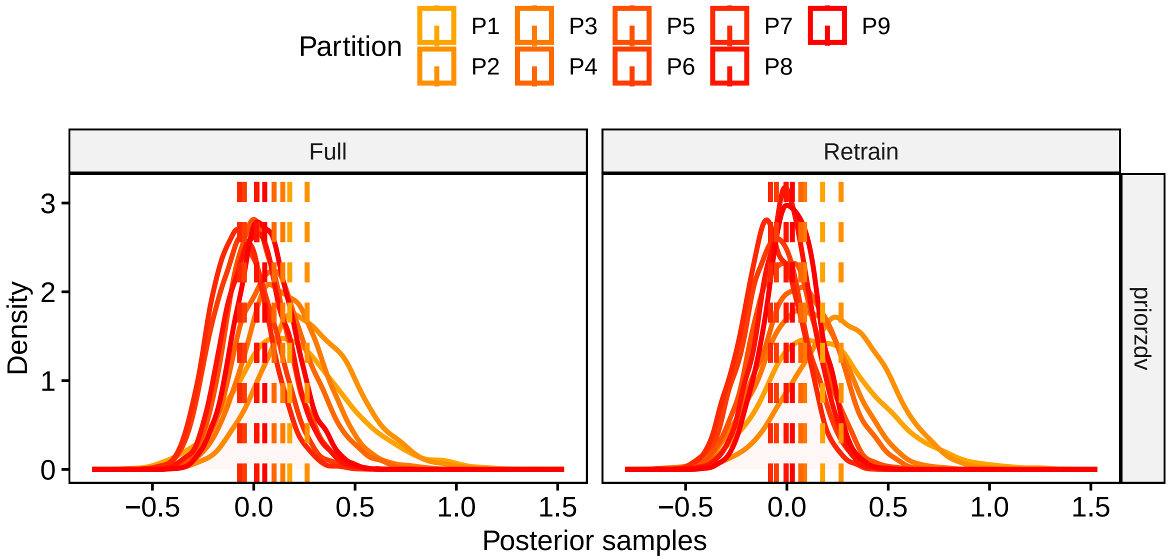
Partition
P1
P2
P3
P4
P5
P6
P7
P8
P9
Full
Retrain
priorzdv
Density
Posterior samples

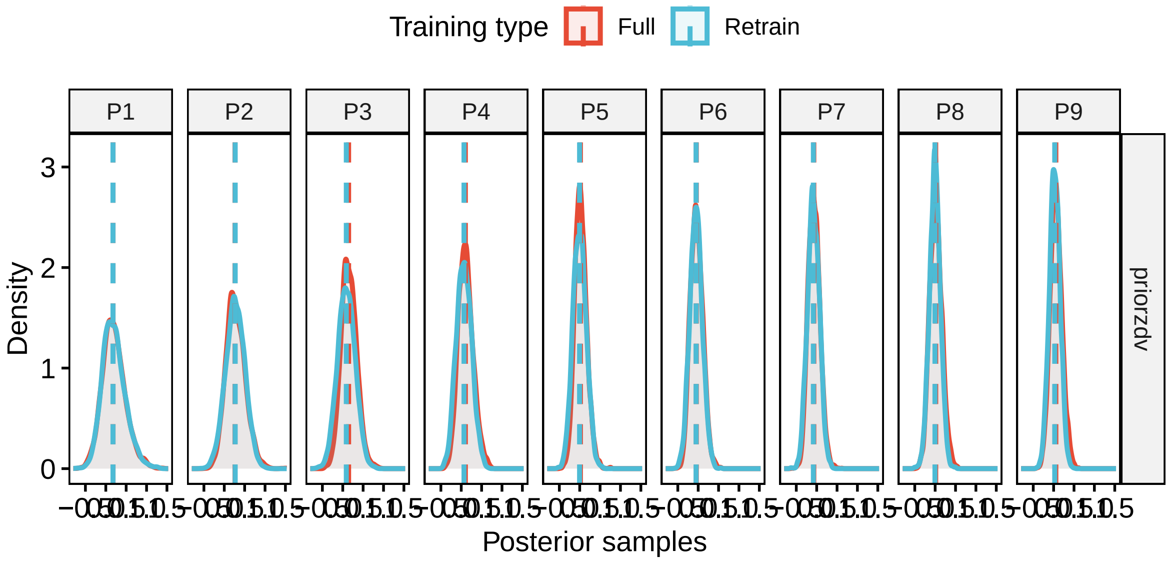
Training type
Full
Retrain
P1
P2
P3
P4
P5
P6
P7
P8
P9
priorzdv
Density
Posterior samples

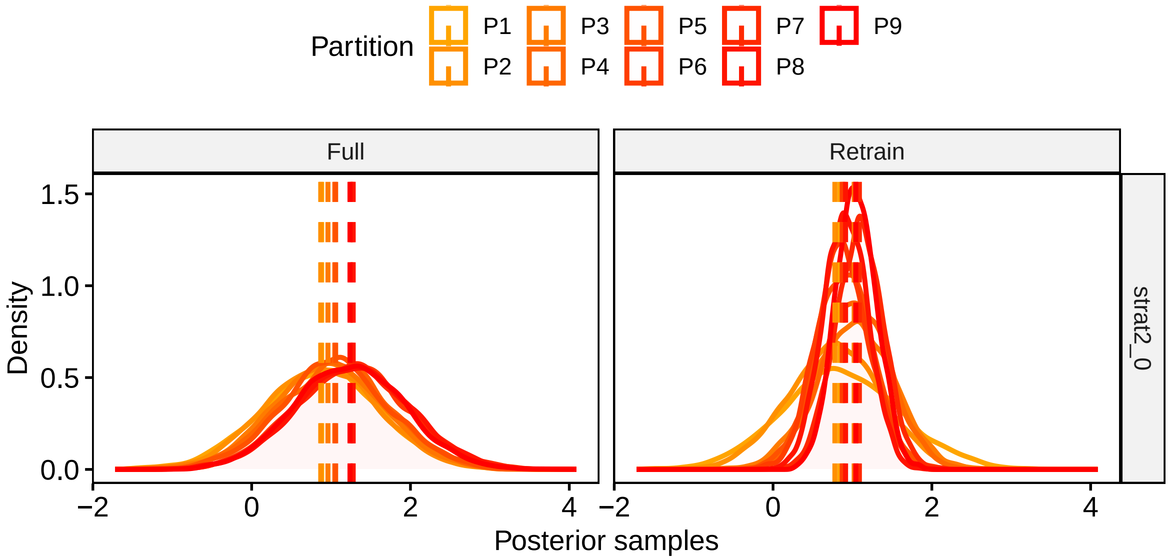
Partition
P1
P2
P3
P4
P5
P6
P7
P8
P9
Full
Retrain
strat2_0
Density
Posterior samples

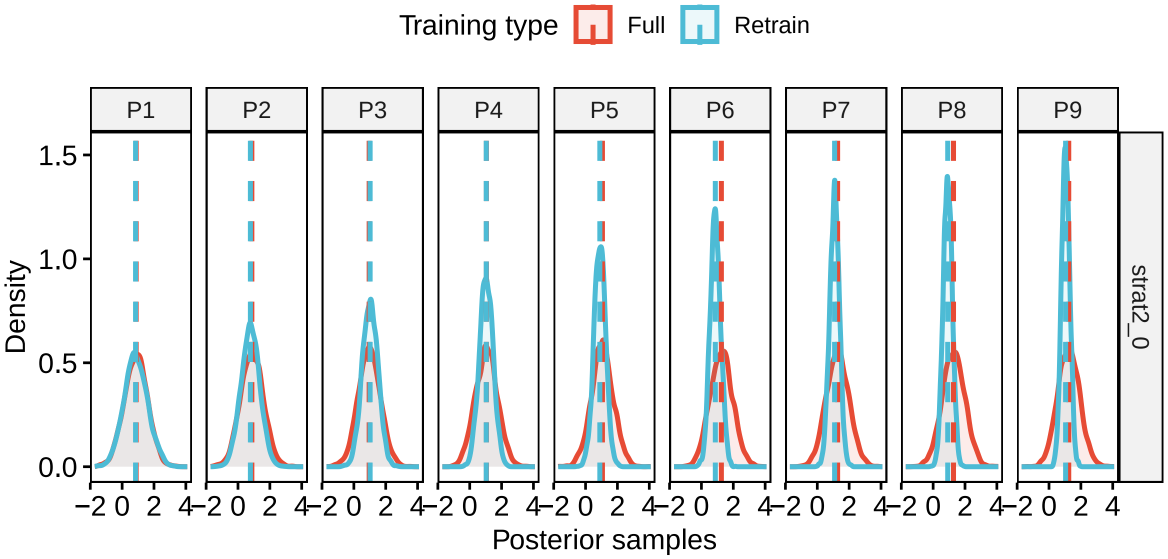
Training type
Full
Retrain
P1
P2
P3
P4
P5
P6
P7
P8
P9
strat2_0
Density
Posterior samples

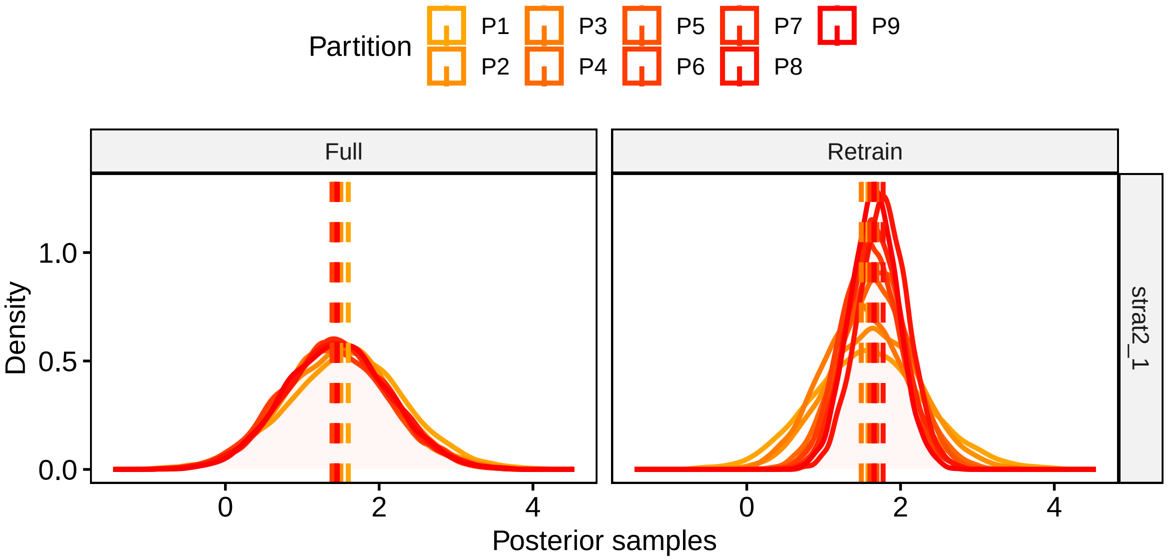
Partition
P1
P2
P3
P4
P5
P6
P7
P8
P9
Full
Retrain
strat2_1
Density
Posterior samples

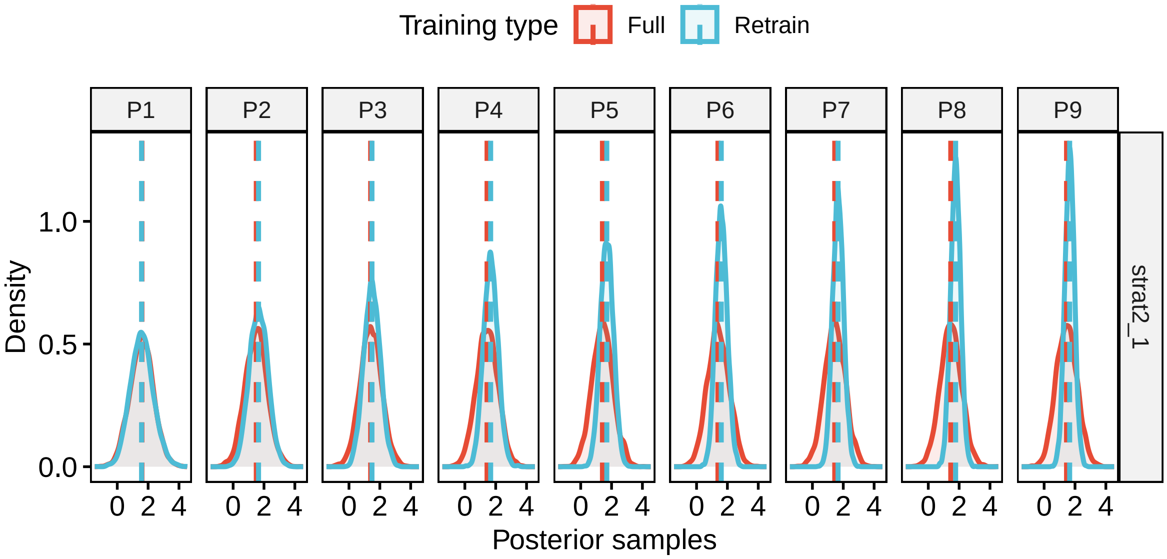
Training type
Full
Retrain
P1
P2
P3
P4
P5
P6
P7
P8
P9
Density
1.0
0.5
0.0
0 2 4
Posterior samples
strat2_1

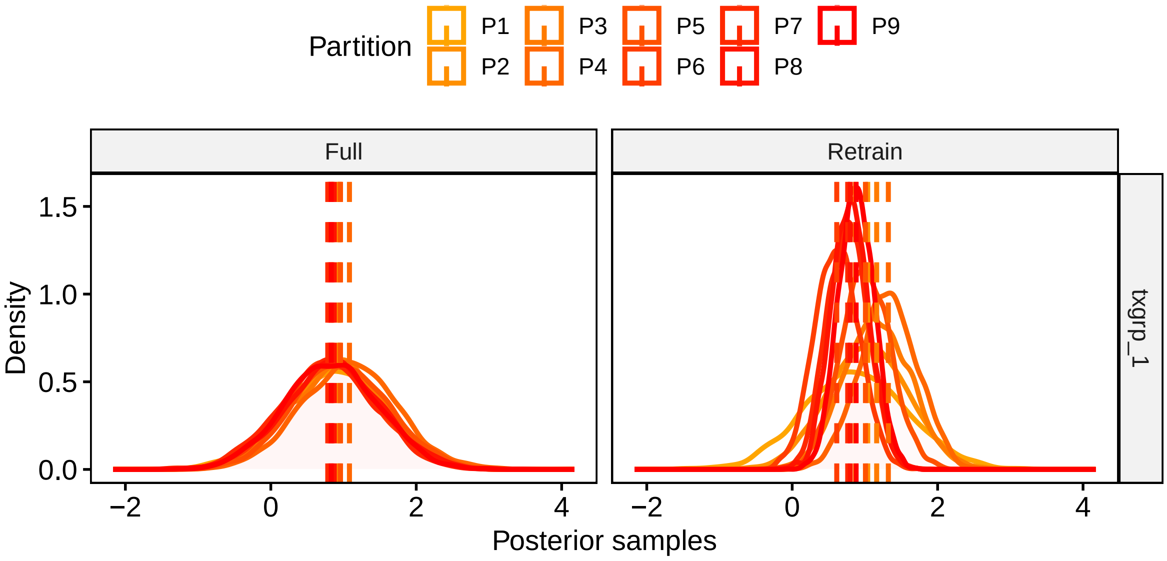
Partition
P1
P2
P3
P4
P5
P6
P7
P8
P9
Full
Retrain
Density
1.5
1.0
0.5
0.0
−2
0
2
4
Posterior samples
txgrp_1

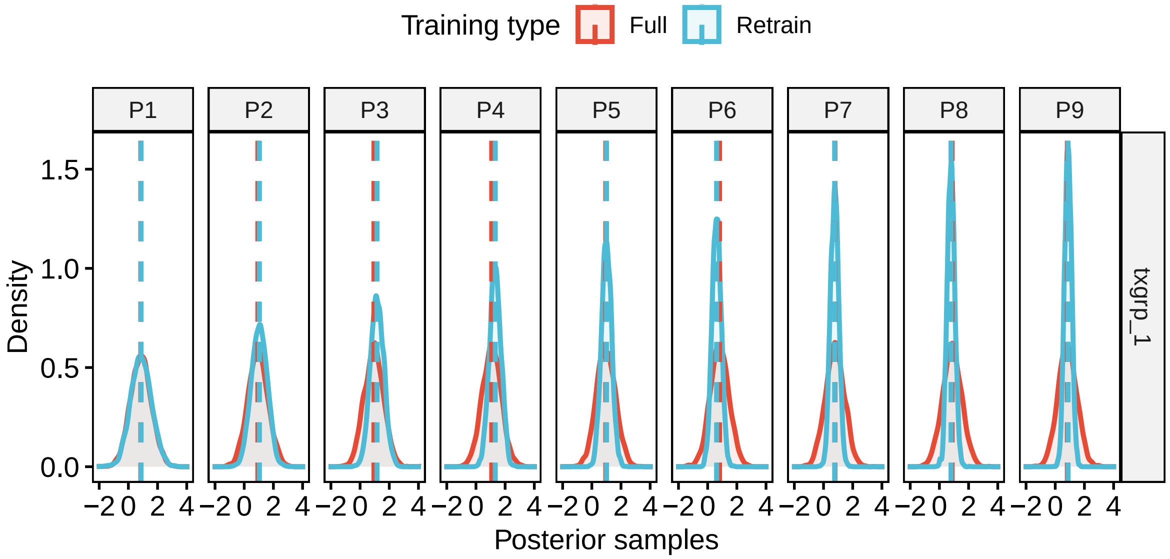
Training type
Full
Retrain
P1
P2
P3
P4
P5
P6
P7
P8
P9
txgrp_1
Density
Posterior samples

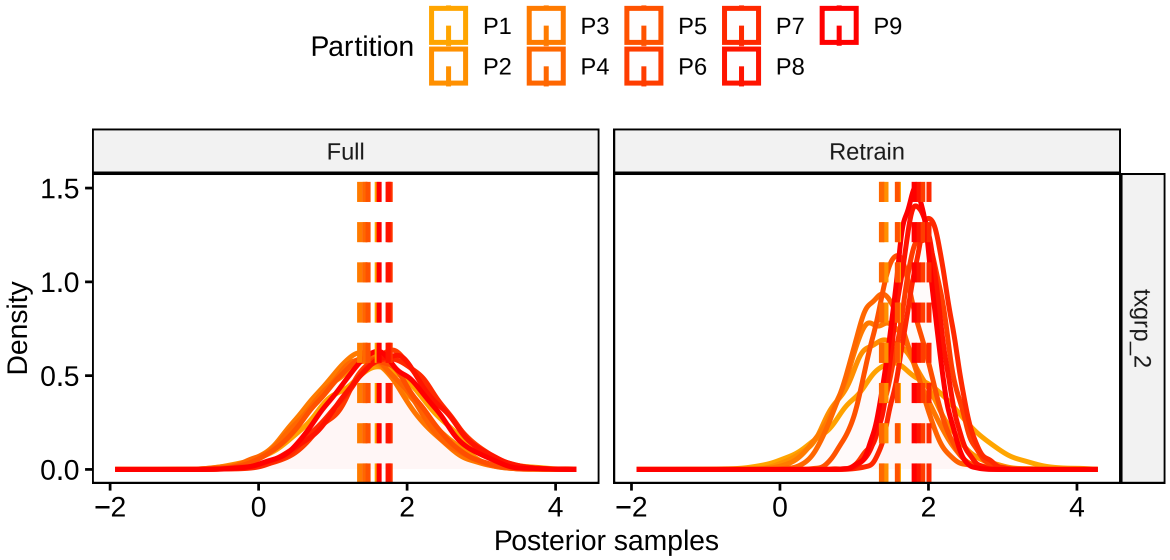
Partition
P1
P2
P3
P4
P5
P6
P7
P8
P9
Full
Retrain
txgrp_2
Density
Posterior samples

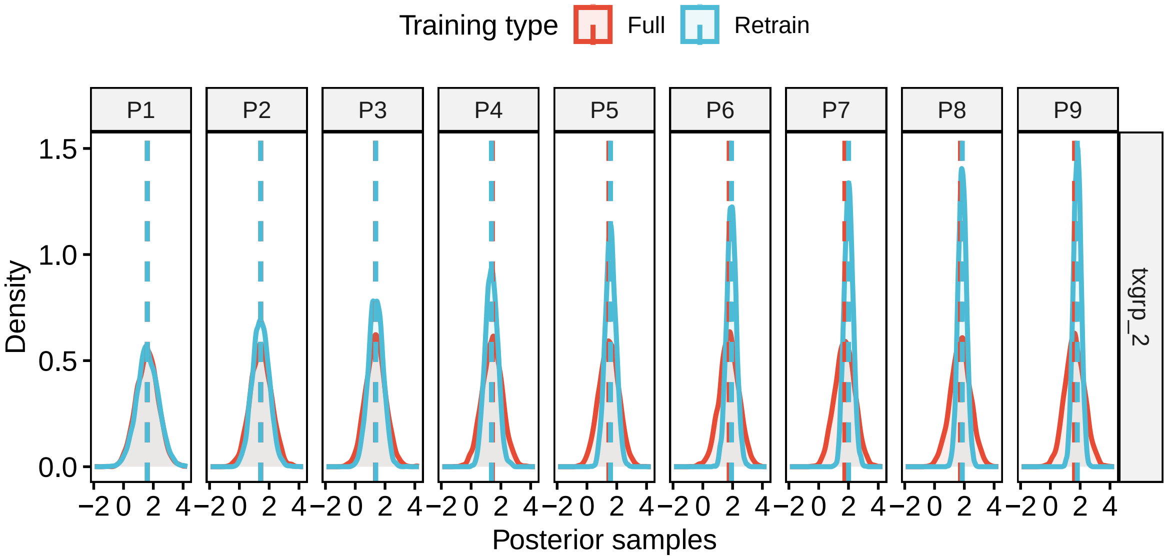
Training type
Full
Retrain
P1
P2
P3
P4
P5
P6
P7
P8
P9
txgrp_2
Density
Posterior samples

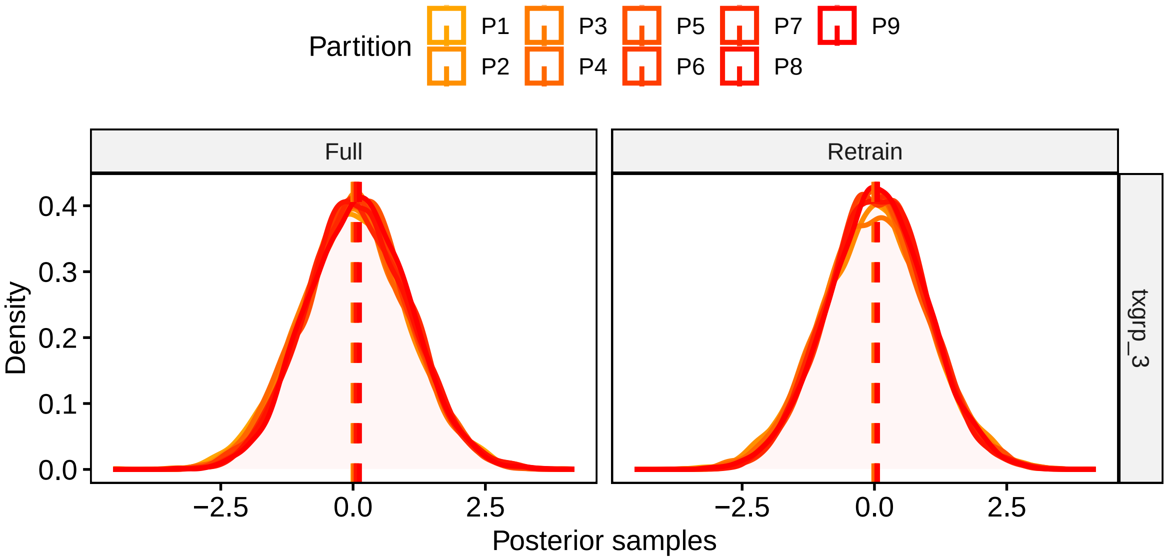
Partition
P1
P2
P3
P4
P5
P6
P7
P8
P9
Full
Retrain
txgrp_3
Density
Posterior samples

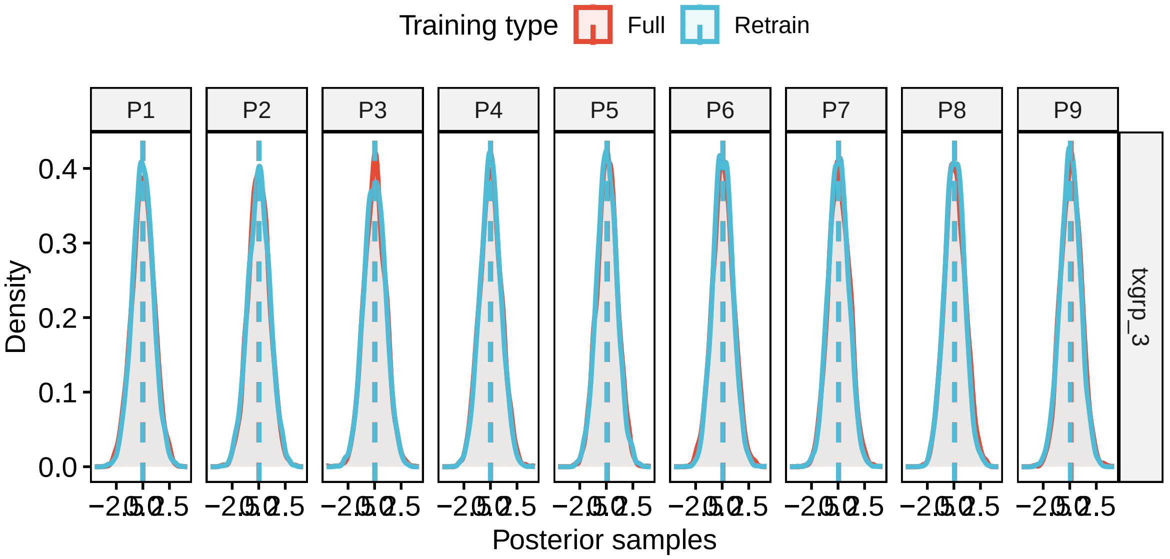
Training type
Full
Retrain
P1
P2
P3
P4
P5
P6
P7
P8
P9
txgrp_3
Density
Posterior samples

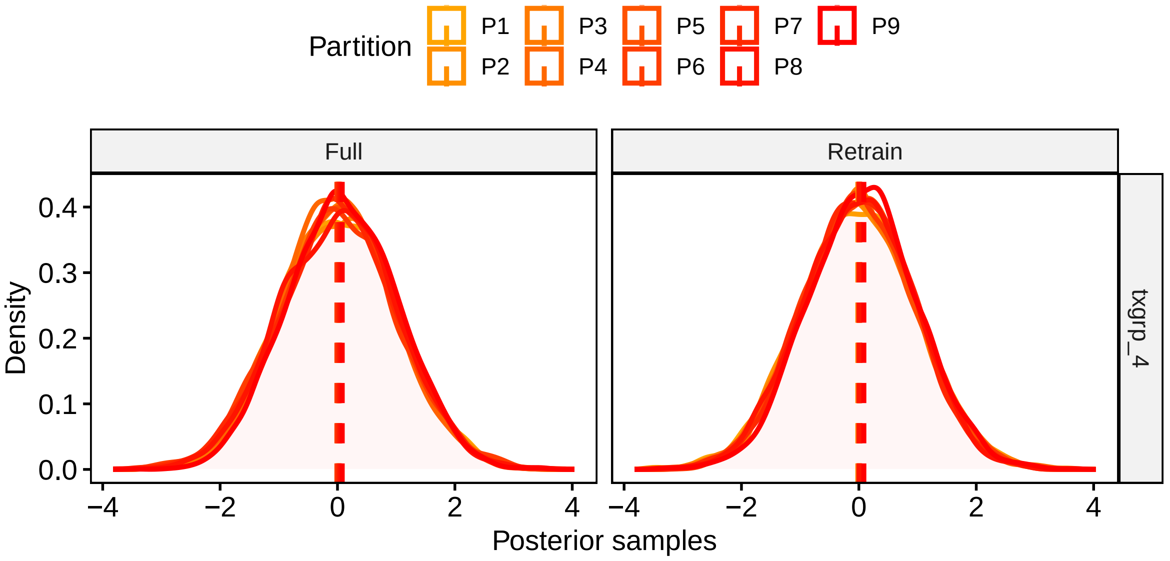
Partition
P1
P2
P3
P4
P5
P6
P7
P8
P9
Full
Retrain
txgrp_4
Density
Posterior samples

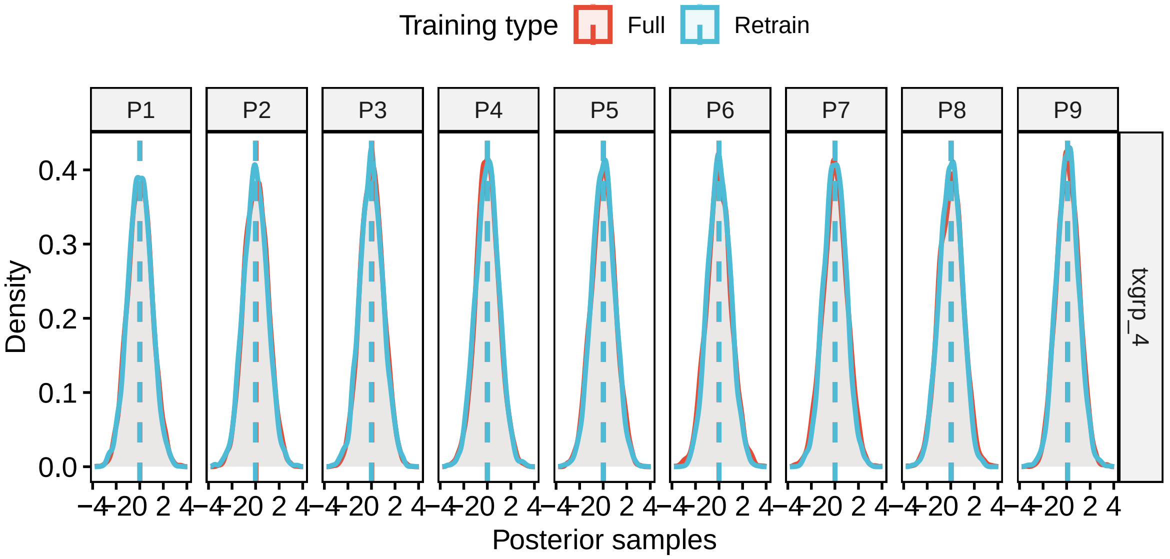

Training type
Full
Retrain
P1
P2
P3
P4
P5
P6
P7
P8
P9
txgrp_4
Density
Posterior samples

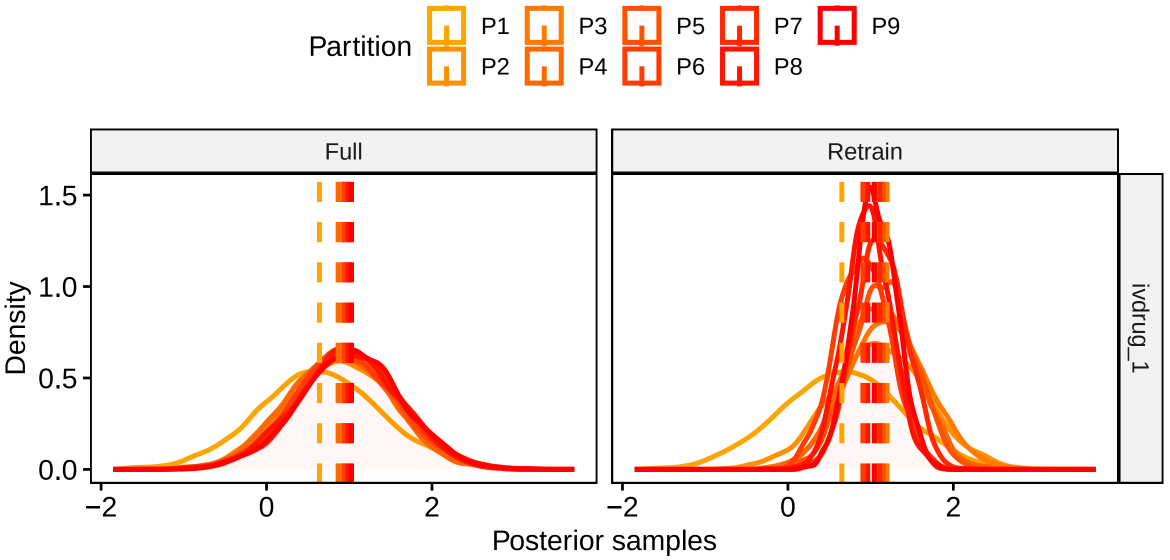

Partition
P1
P2
P3
P4
P5
P6
P7
P8
P9
Full
Retrain
ivdrug_1
Density
Posterior samples

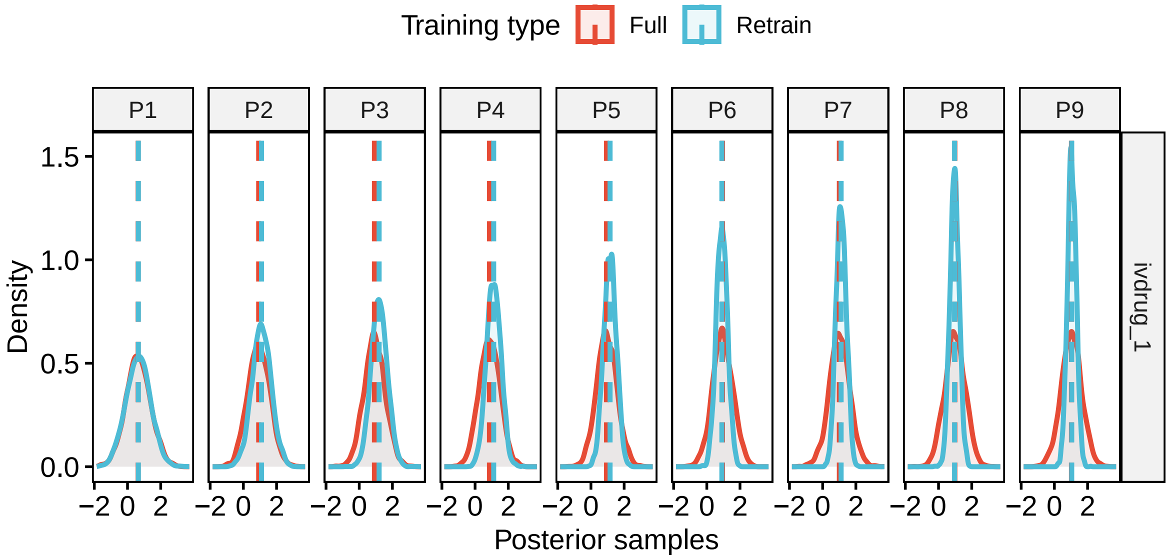
Training type
Full
Retrain
P1
P2
P3
P4
P5
P6
P7
P8
P9
ivdrug_1
Density
Posterior samples

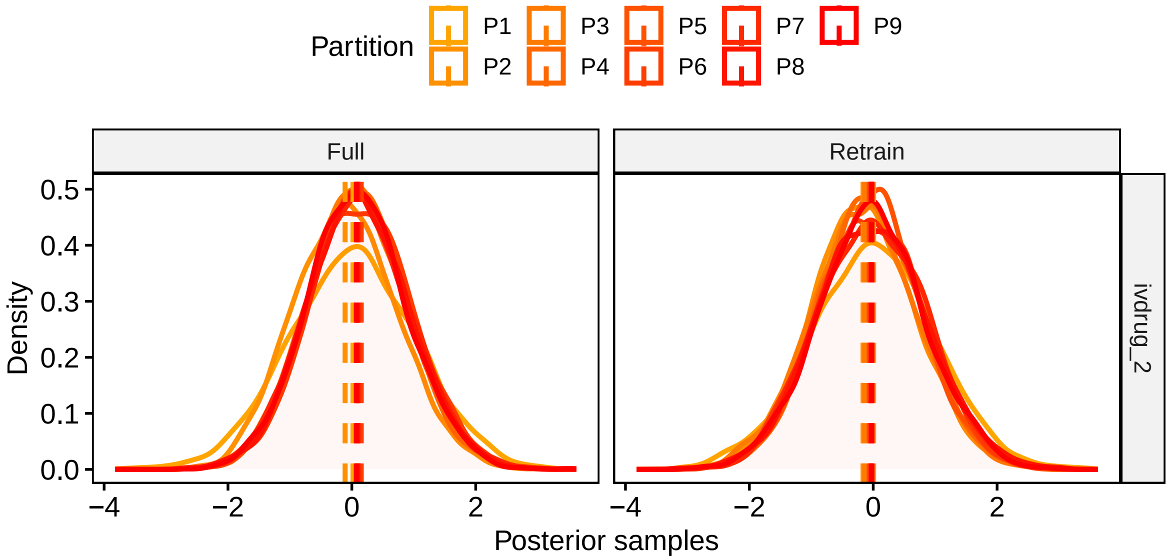
Partition
P1
P2
P3
P4
P5
P6
P7
P8
P9
Full
Retrain
ivdrug_2
Density
Posterior samples

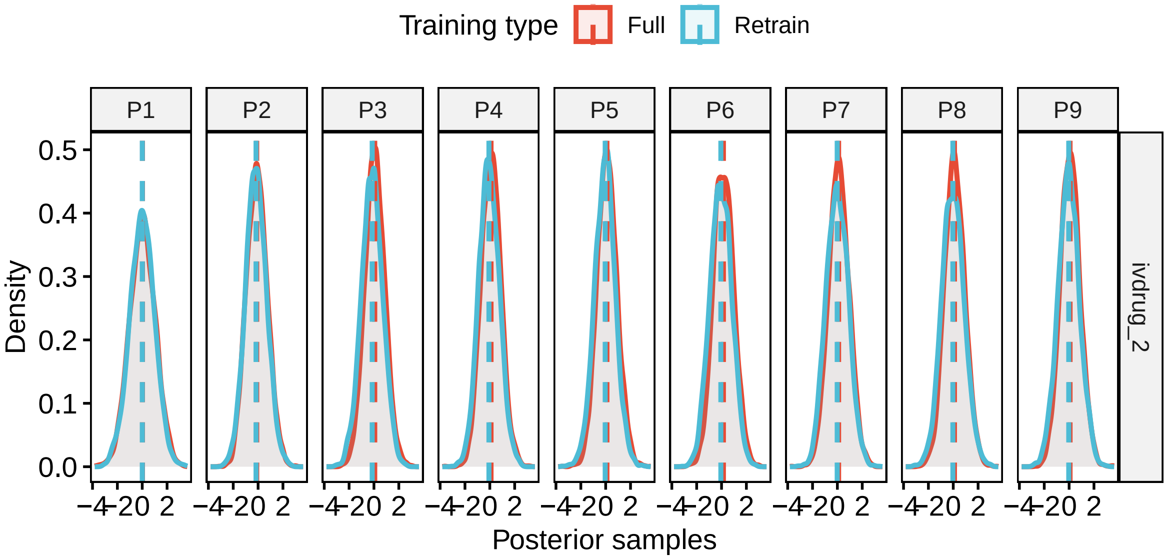
Training type
Full
Retrain
P1
P2
P3
P4
P5
P6
P7
P8
P9
ivdrug_2
Density
Posterior samples

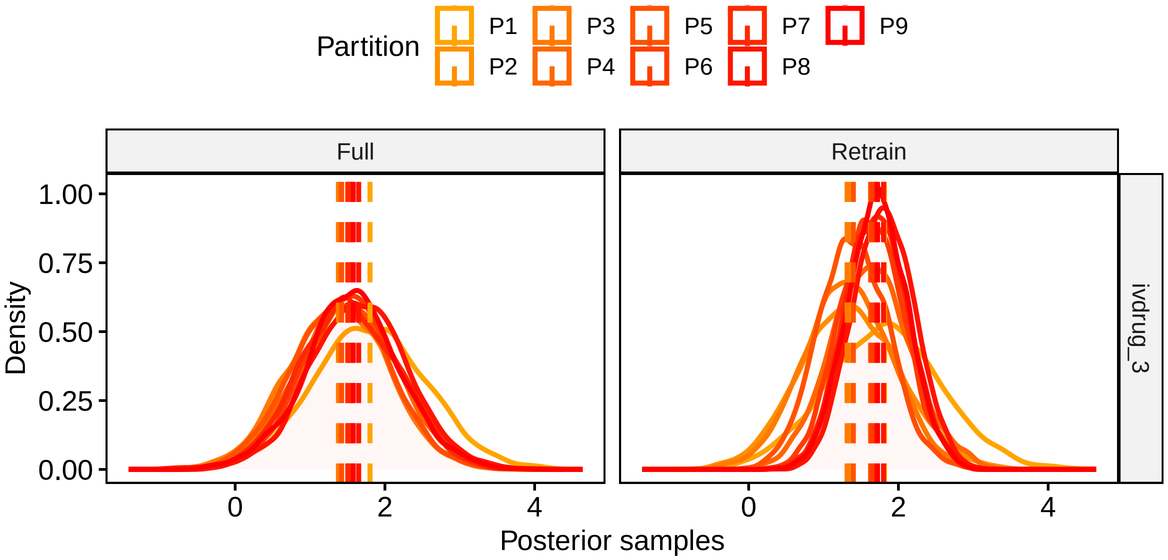
Partition
P1
P2
P3
P4
P5
P6
P7
P8
P9
Full
Retrain
ivdrug_3
Density
Posterior samples

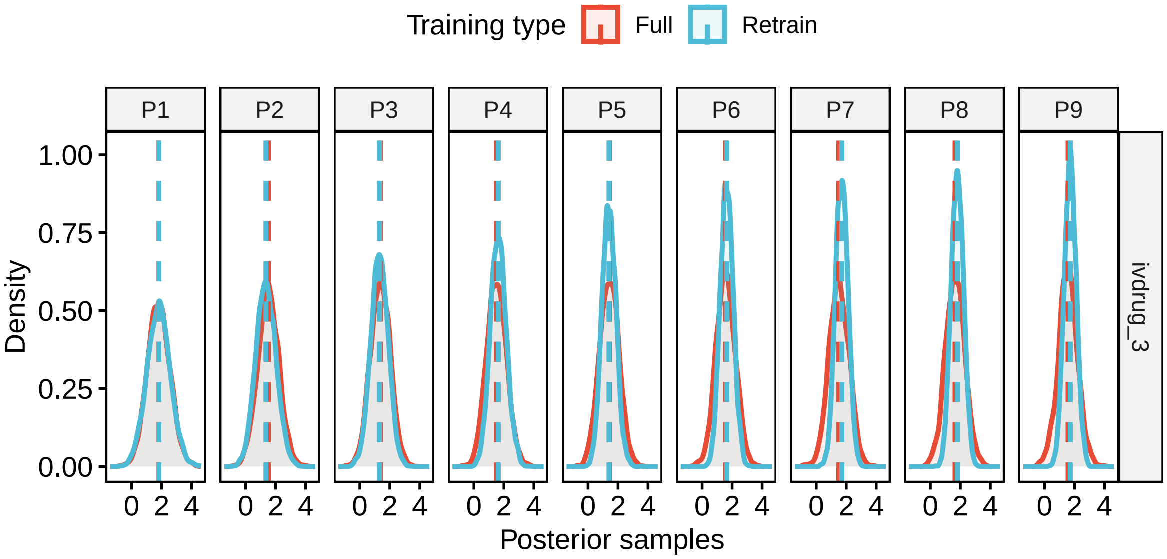
Training type
Full
Retrain
P1
P2
P3
P4
P5
P6
P7
P8
P9
ivdrug_3
Density
1.00
0.75
0.50
0.25
0.00
0 2 4
Posterior samples

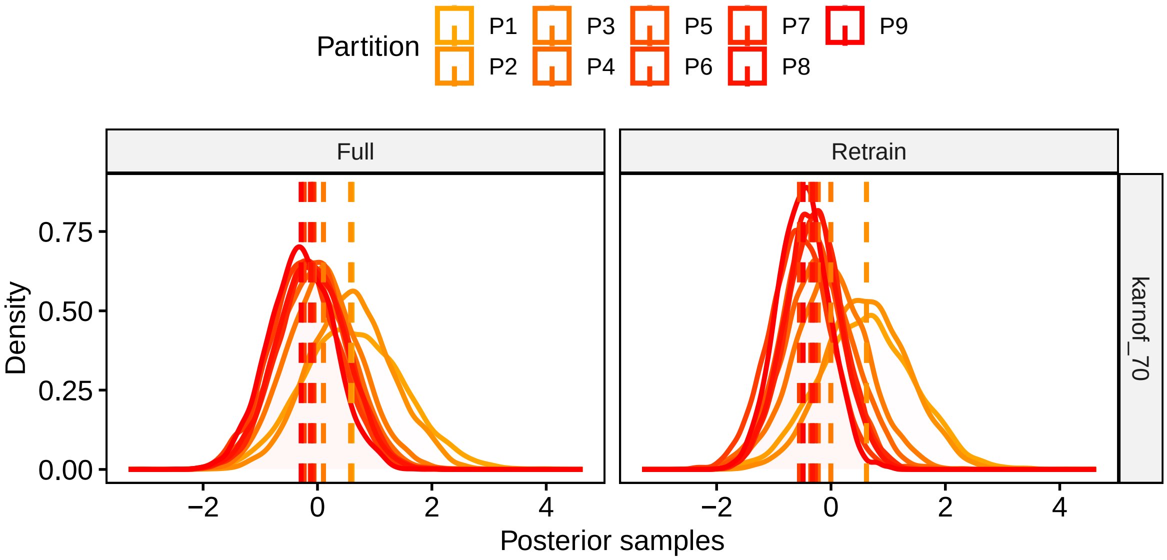
Partition
P1
P2
P3
P4
P5
P6
P7
P8
P9
Full
Retrain
karnof_70
Density
0.75
0.50
0.25
0.00
−2
0
2
4
Posterior samples

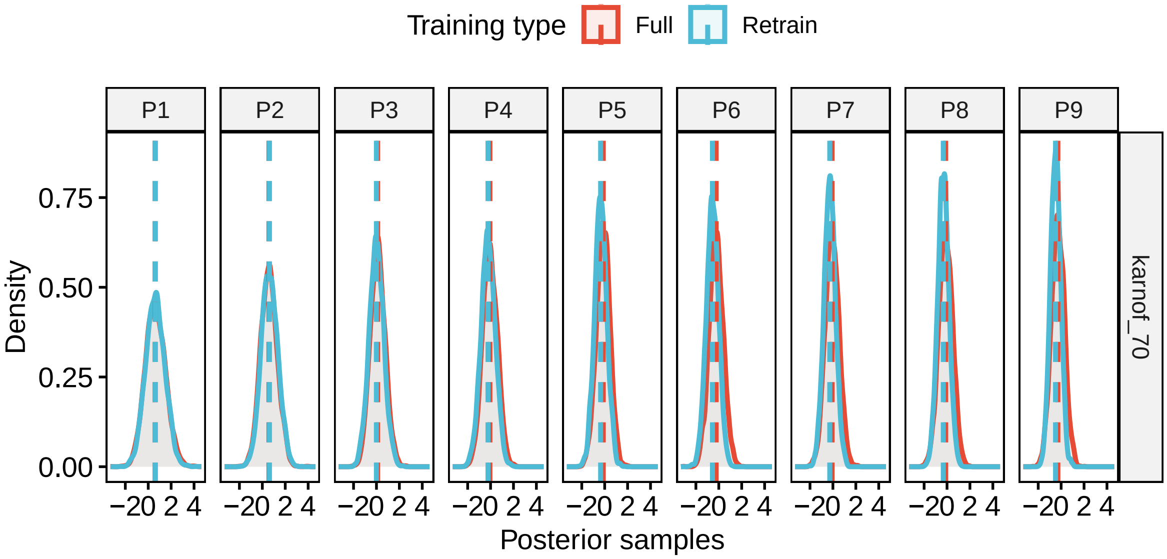
Training type
Full
Retrain
P1
P2
P3
P4
P5
P6
P7
P8
P9
Density
0.75
0.50
0.25
0.00
−20 2 4
Posterior samples
karnof_70

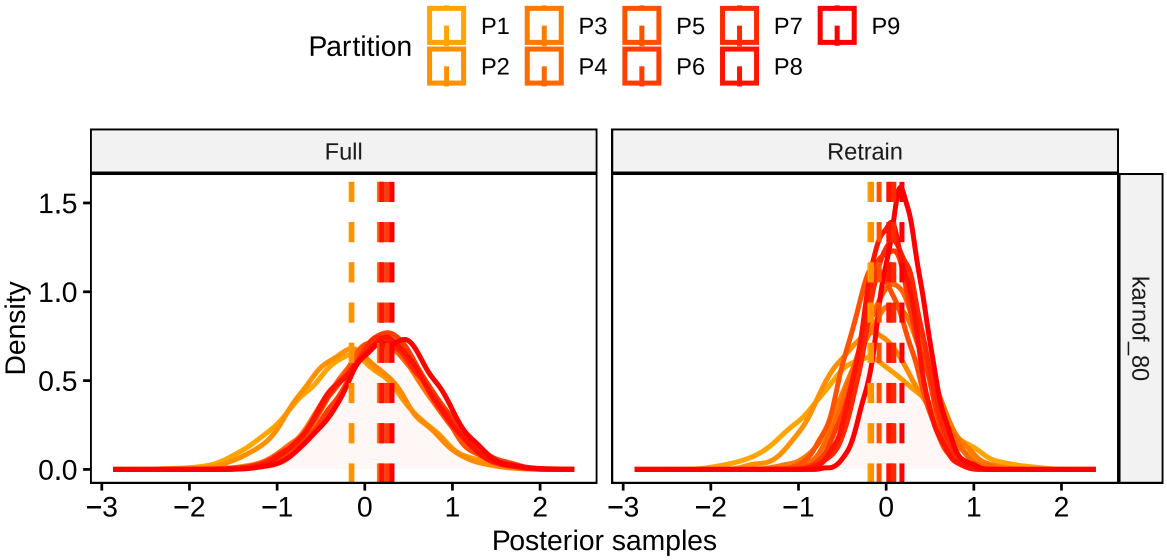
Partition
P1
P2
P3
P4
P5
P6
P7
P8
P9
Full
Retrain
Density
1.5
1.0
0.5
0.0
−3
−2
−1
0
1
2
Posterior samples
karnof_80

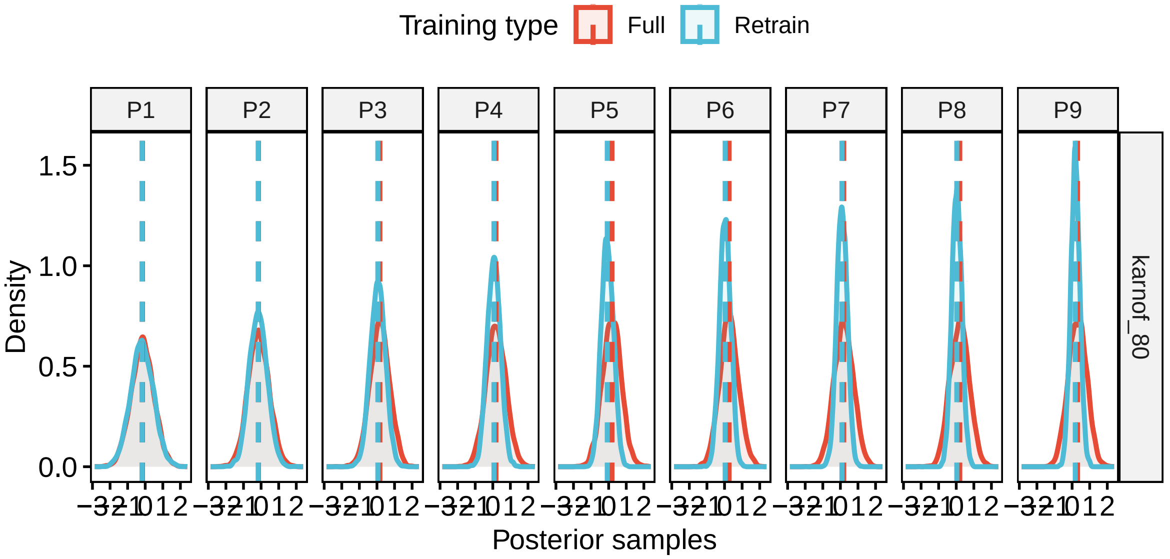

Training type
Full
Retrain
P1
P2
P3
P4
P5
P6
P7
P8
P9
karnof_80
Density
Posterior samples

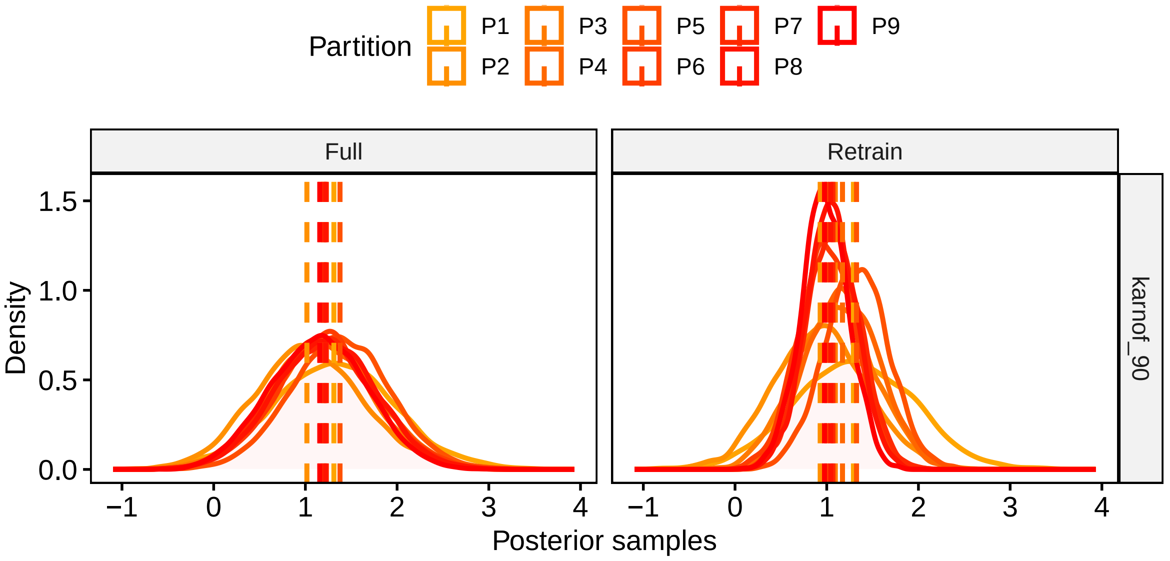

Partition
P1
P2
P3
P4
P5
P6
P7
P8
P9
Full
Retrain
karnof_90
Density
Posterior samples

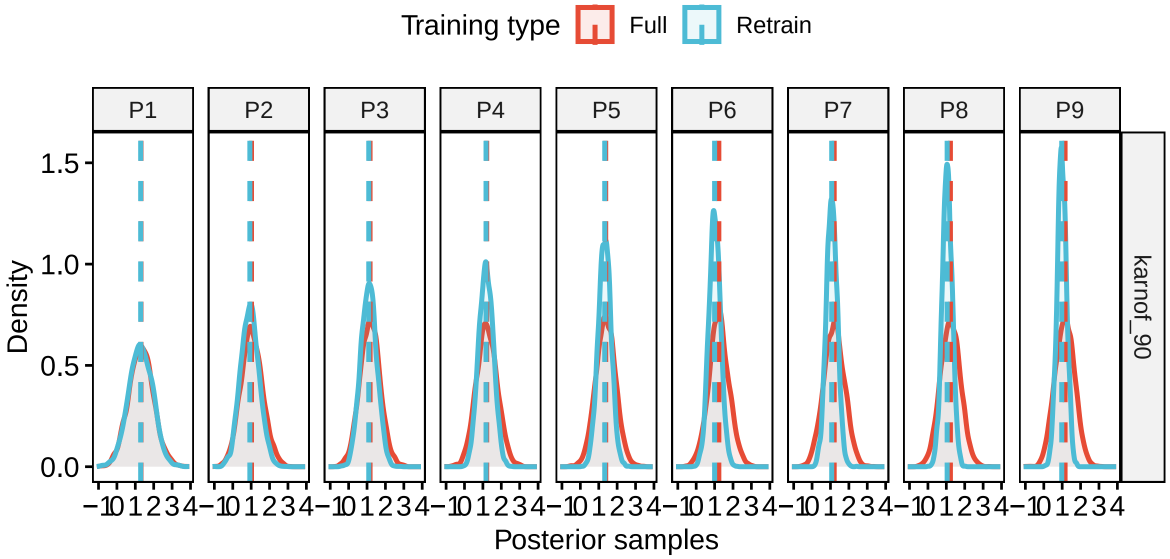
Training type
Full
Retrain
P1
P2
P3
P4
P5
P6
P7
P8
P9
karnof_90
Density
1.5
1.0
0.5
0.0
Posterior samples

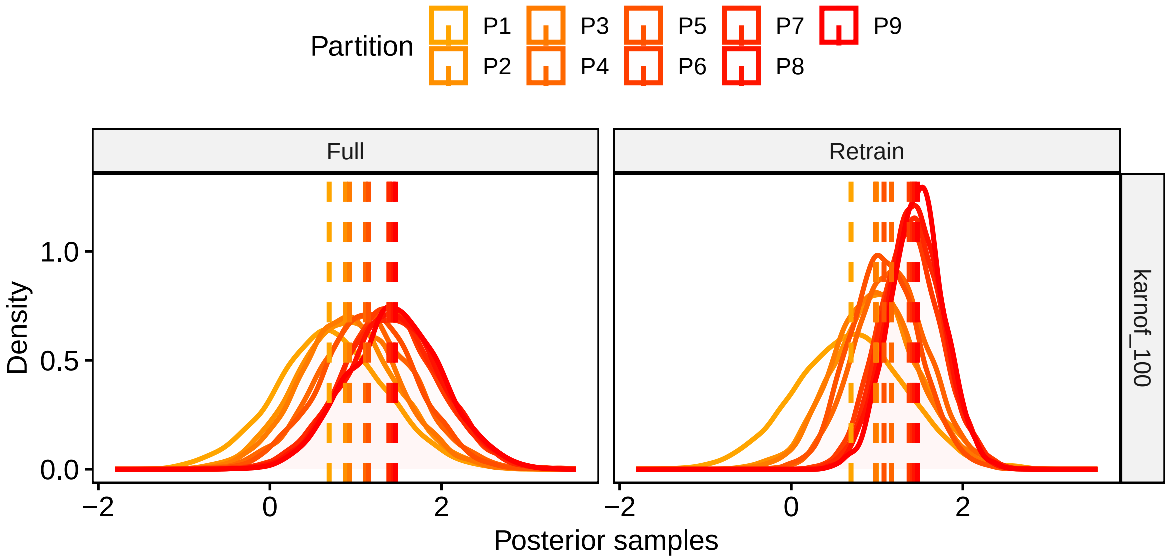
Partition
P1
P2
P3
P4
P5
P6
P7
P8
P9
Full
Retrain
karnof_100
Density
1.0
0.5
0.0
−2
0
2
Posterior samples

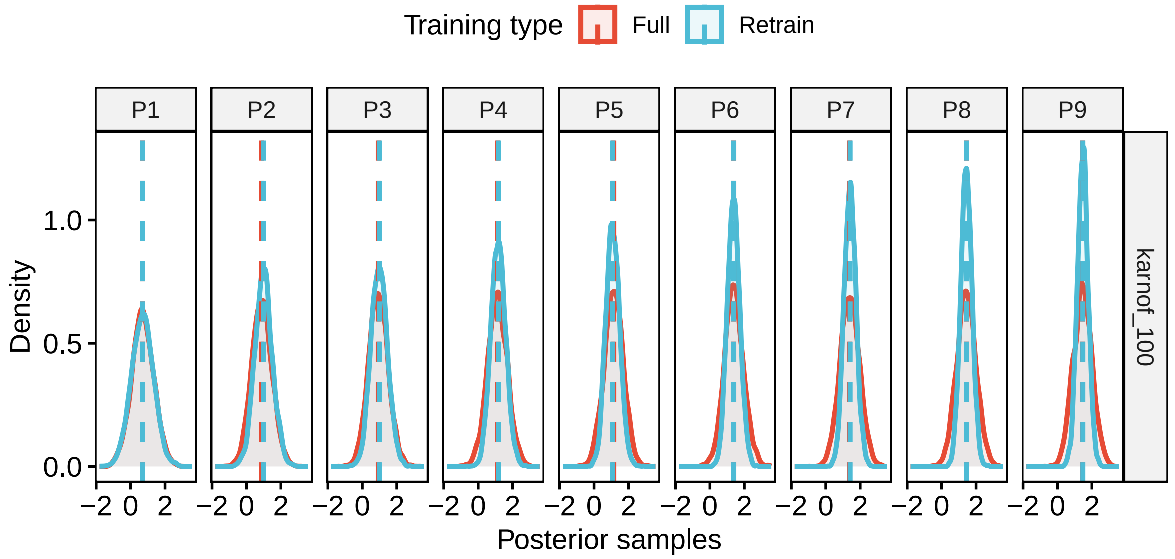
Training type
Full
Retrain
P1
P2
P3
P4
P5
P6
P7
P8
P9
karnof_100
Density
Posterior samples

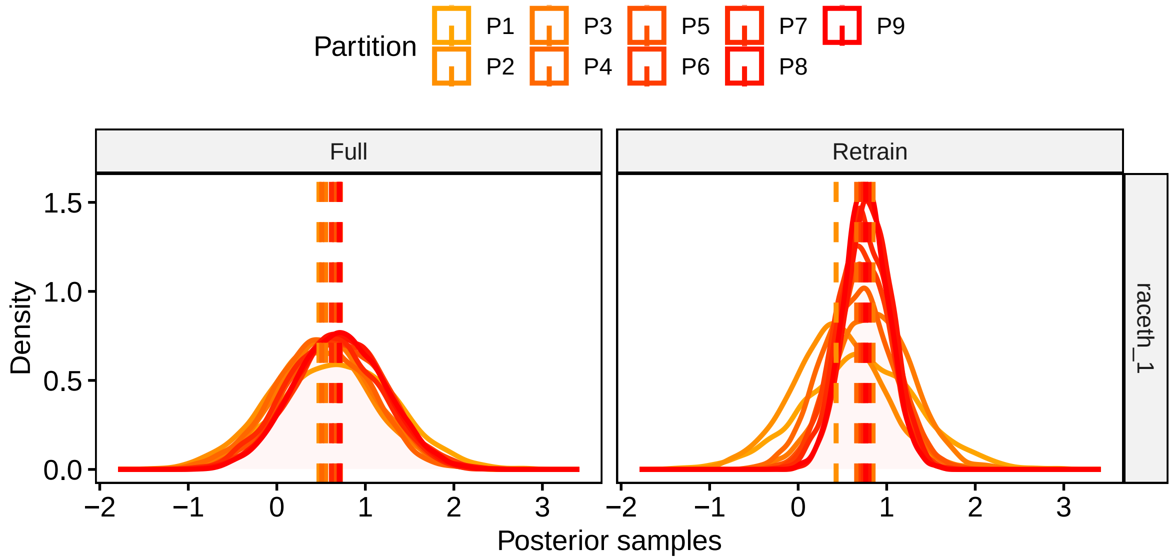
Partition
P1
P2
P3
P4
P5
P6
P7
P8
P9
Full
Retrain
raceth_1
Density
Posterior samples

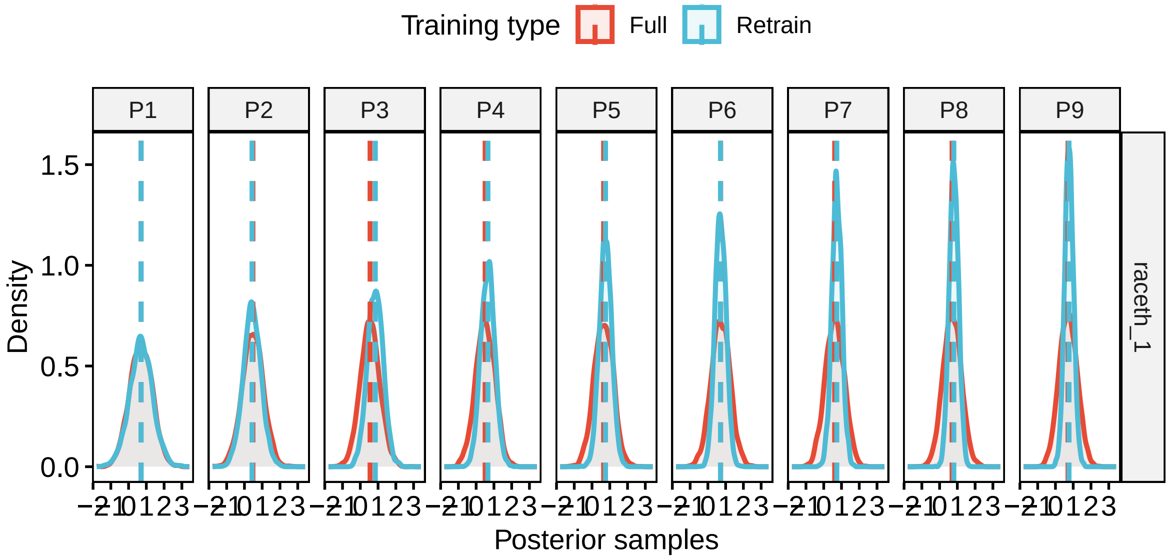
Training type
Full
Retrain
P1
P2
P3
P4
P5
P6
P7
P8
P9
Density
Posterior samples
raceth_1

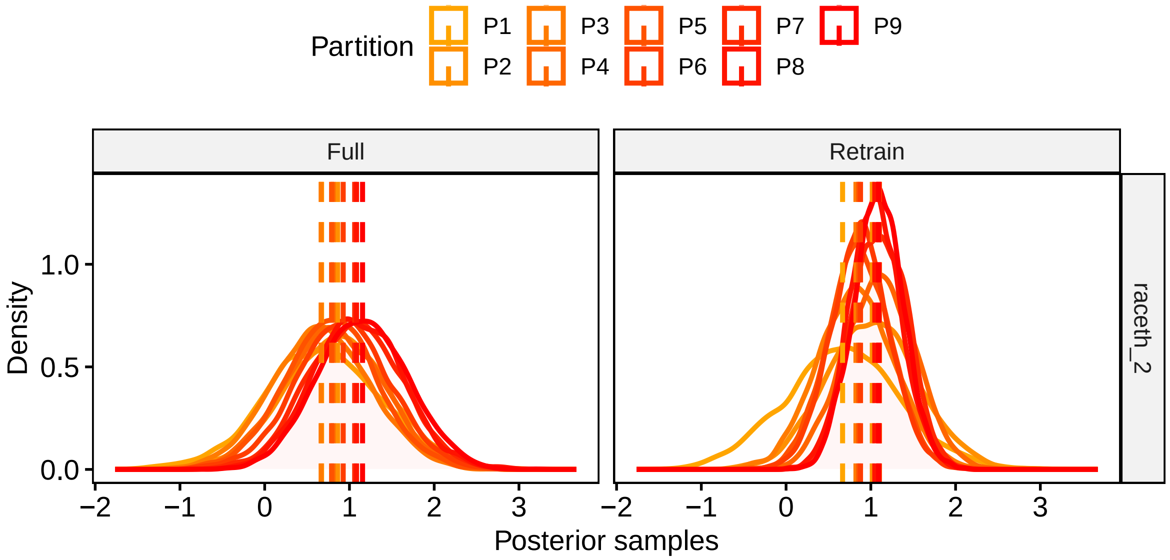
Partition
P1
P2
P3
P4
P5
P6
P7
P8
P9
Full
Retrain
Density
Posterior samples
raceth_2

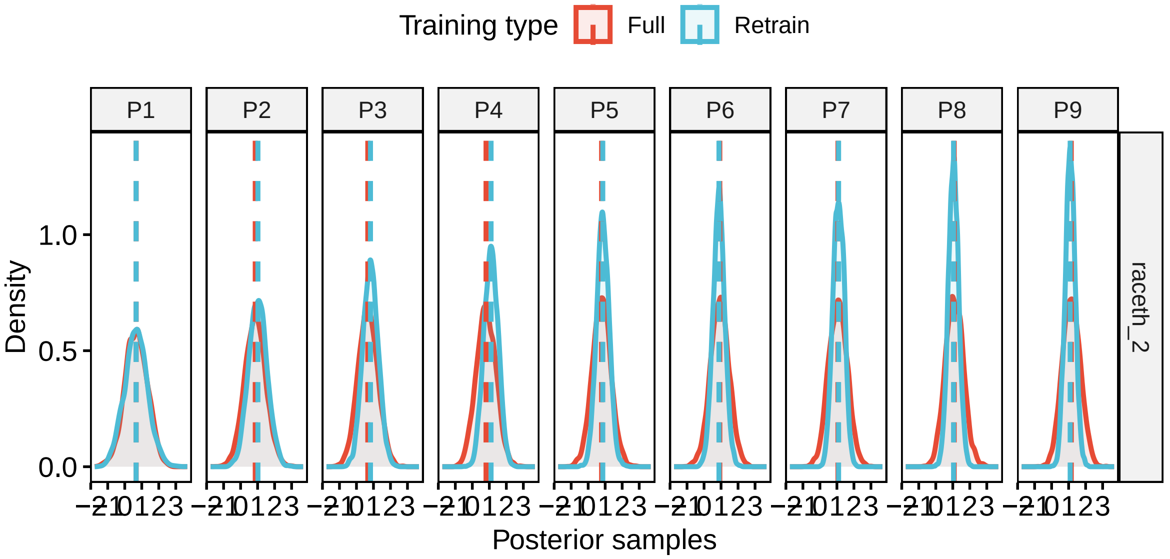

Training type
Full
Retrain
P1
P2
P3
P4
P5
P6
P7
P8
P9
raceth_2
Density
1.0
0.5
0.0
Posterior samples

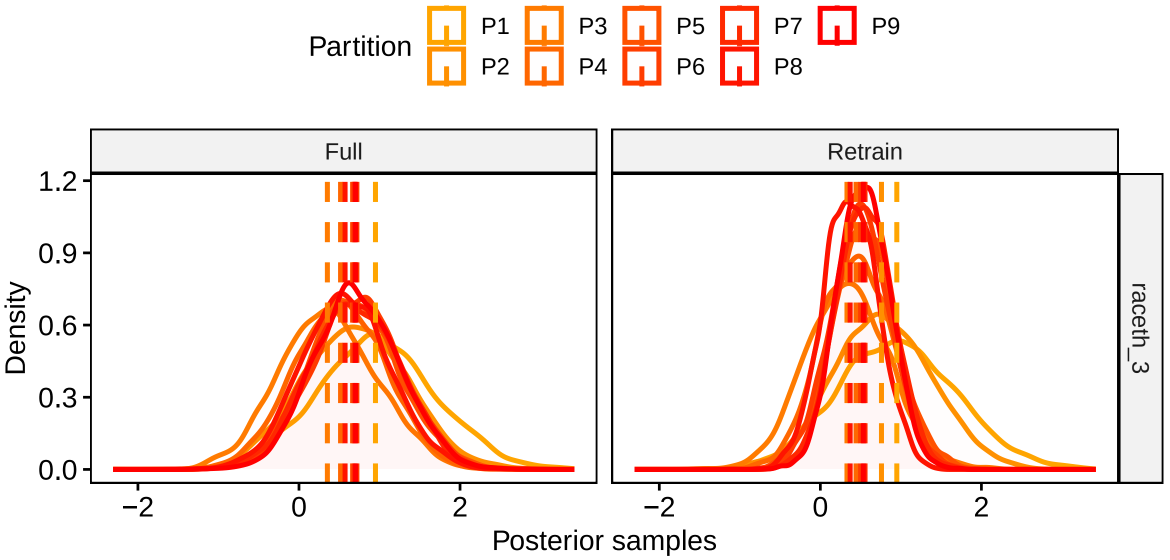

Partition
P1
P2
P3
P4
P5
P6
P7
P8
P9
Full
Retrain
raceth_3
Density
1.2
0.9
0.6
0.3
0.0
−2
0
2
Posterior samples

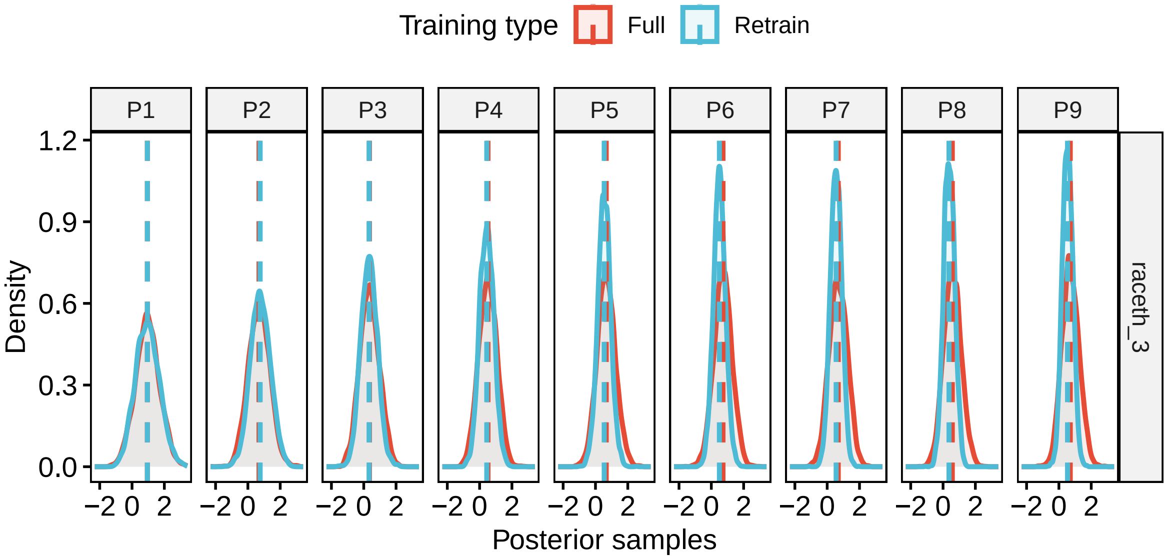

Training type
Full
Retrain
P1
P2
P3
P4
P5
P6
P7
P8
P9
raceth_3
Density
Posterior samples

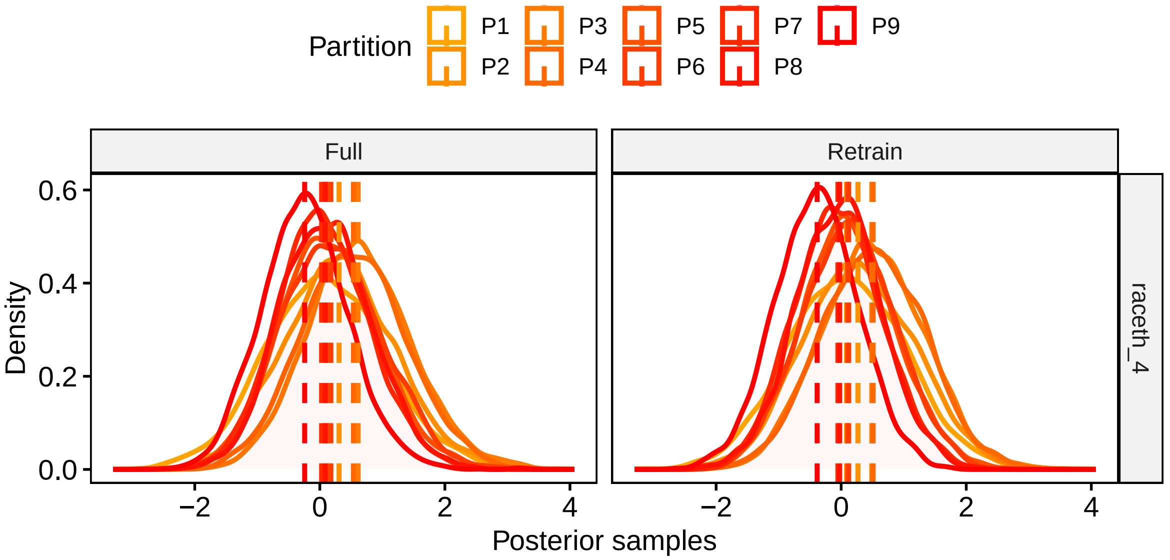

Partition
P1
P2
P3
P4
P5
P6
P7
P8
P9
Full
Retrain
raceth_4
Density
Posterior samples

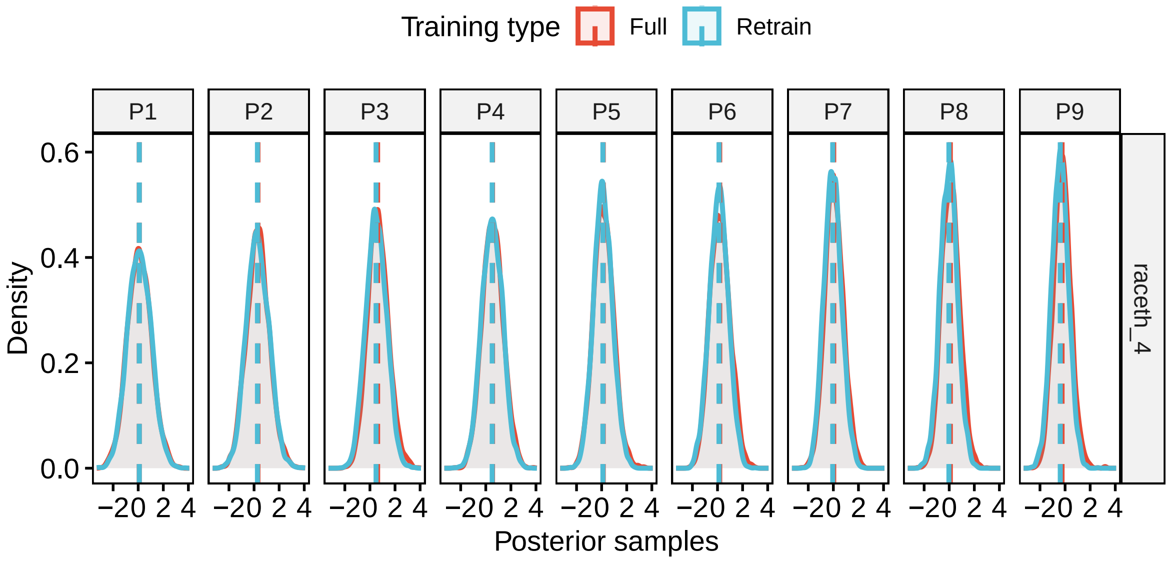
Training type
Full
Retrain
P1
P2
P3
P4
P5
P6
P7
P8
P9
raceth_4
Density
Posterior samples

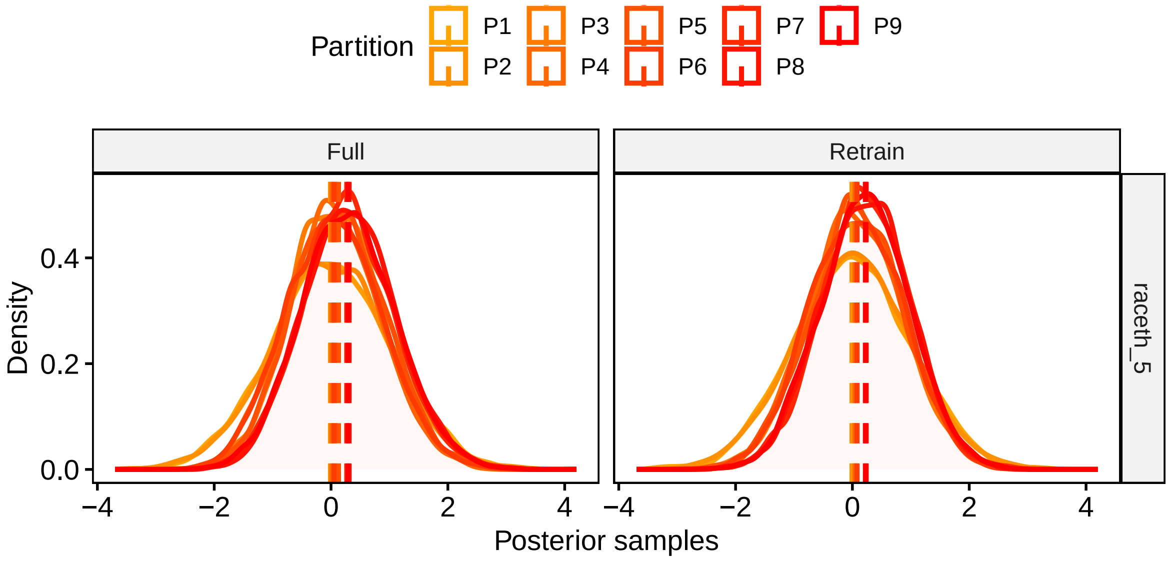
Partition
P1
P2
P3
P4
P5
P6
P7
P8
P9
Full
Retrain
raceth_5
Density
Posterior samples

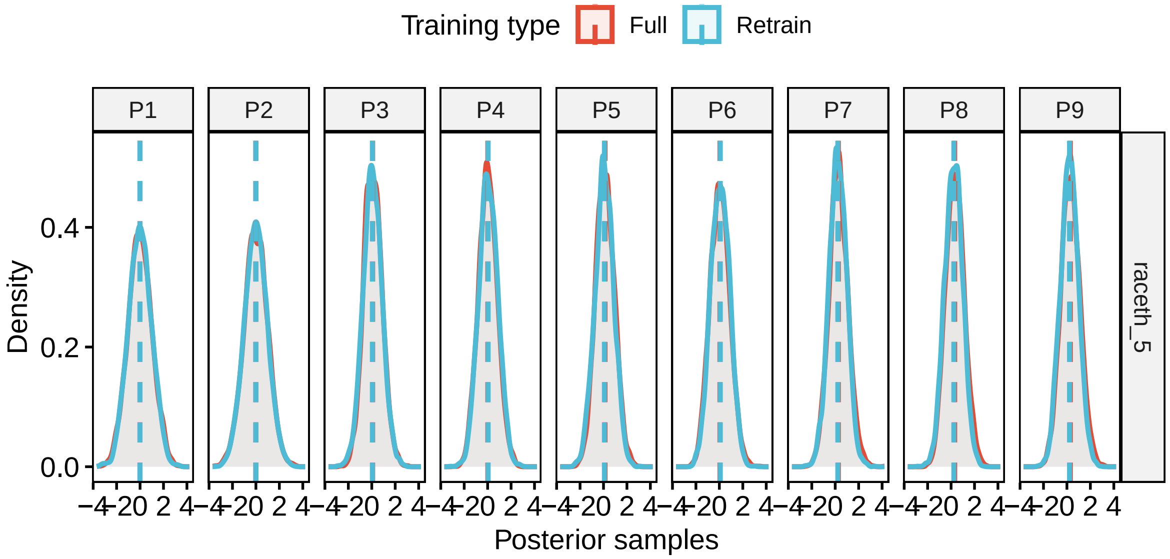
Training type
Full
Retrain
P1
P2
P3
P4
P5
P6
P7
P8
P9
raceth_5
Density
Posterior samples

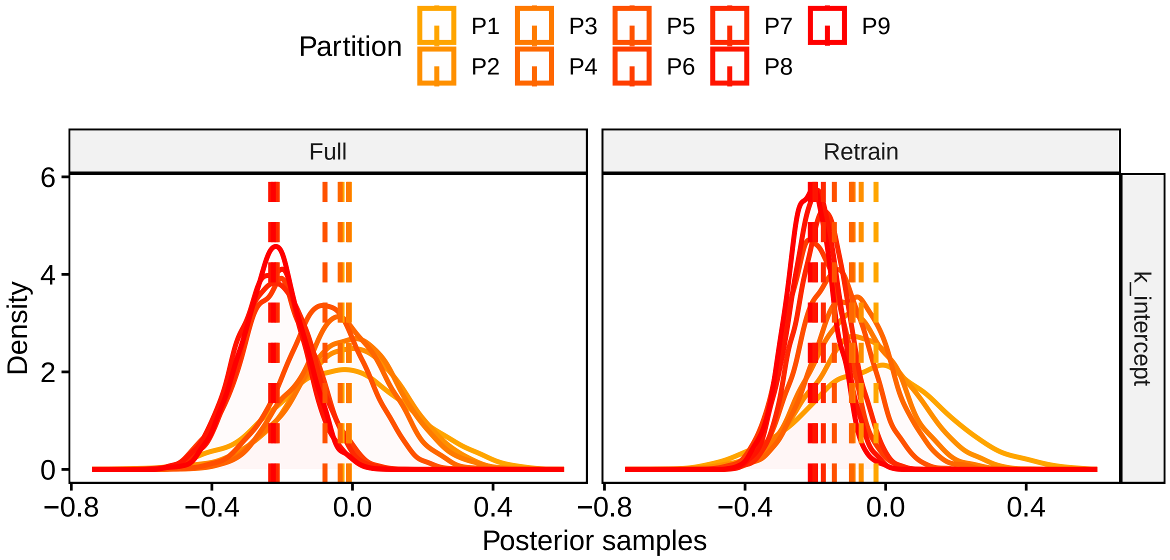
Partition
P1
P2
P3
P4
P5
P6
P7
P8
P9
Full
Retrain
k_intercept
Density
Posterior samples

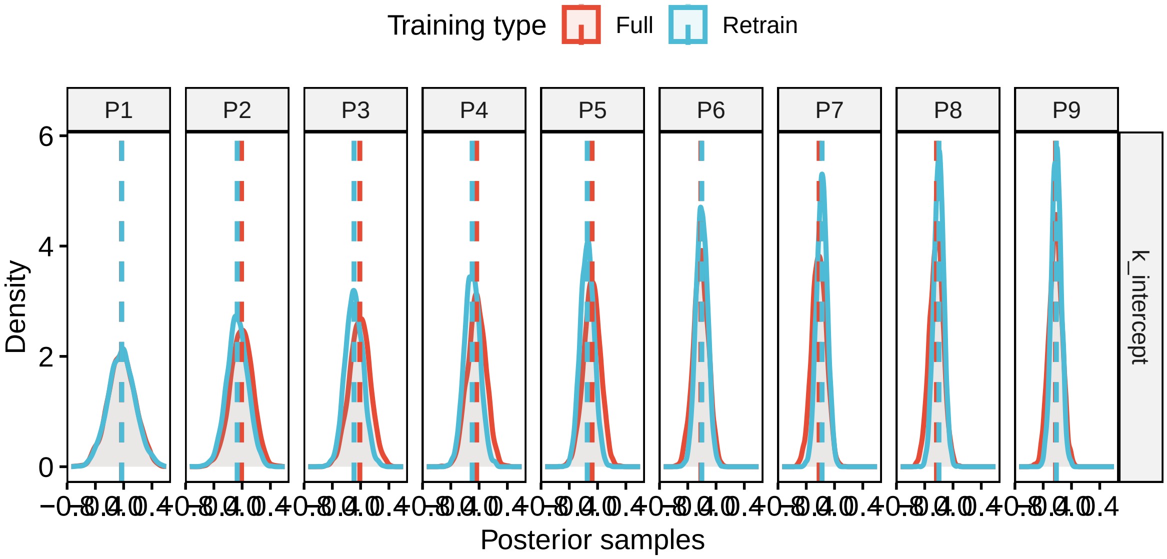
Training type
Full
Retrain
P1
P2
P3
P4
P5
P6
P7
P8
P9
k_intercept
Density
Posterior samples
0
2
4
6
