# Supplementary material for: Bayesian parametric models for survival prediction in medical applications
Source: BMC Med Res Methodol. 2023 Oct 26;23:250. doi: 10.1186/s12874-023-02059-4 (PMC10605790; doi:10.1186/s12874-023-02059-4)

Bayesian parametric models for survival prediction in medical applications

Retraining using Bayes rule

Iwan Paolucci, PhD

7/2/22

Table of contents

Aim 2

Setup 2

Load data 3

Preprocess data . . . 3

Results 5

Performence over time by experiment and model . . . 6

Difference between training types . . . 7

Test for equivalence . . . 9

Performance by model 12

ACTG . . . 12

GBCS . . . 13

PBC . . . 14

WHAS . . . 15

Aim

The aim of this experiment was to test whether continuous learning using Bayesian model updating performs as good as training on the full dataset each time. The DeepSurv algorithm is used as control which uses traditional transfer learning for Neural Networks.

Hypothesis: The difference in C-Index between full retraining and model updating is 0 with an equivalence margin of 0.01 [-0.01, 0.01].

Setup

```
library(ggplot2)
library(ggpubr)
library(gridExtra)
library(gtsummary)
library(dplyr)
library(gt)
library(bayestestR)
library(rstanarm)
library(stringr)
```

Load data

```
data <- read.csv('data/results_retraining.csv') %>%
  filter(run <= 75 )
```

Preprocess data

```
data.full <- data %>% filter(train_type == 'full')
data.retrain <- data %>% filter(train_type == 'retrain')

data.merge <- base::merge(data.full, data.retrain,
                          by.x = c('model', 'experiment', 'run', 'iter'),
                          by.y = c('model', 'experiment', 'run', 'iter'))
data.merge <- data.merge %>% rename(
    cindex.full = cindex.x,
    cindex.retrain = cindex.y
  ) %>%
  select(model, experiment, run, iter, cindex.full, cindex.retrain) %>%
  mutate(
    cindex.diff = cindex.full - cindex.retrain
  )
```

```
data <- data %>%
  mutate(experiment_lbl = factor(experiment,
                                 labels = c("ACTG", "GBCS", "PBC", "WHAS")),
         model_lbl = factor(model,
                            labels = c("DeepSurv", "BPS Exp", "BPS Wb")),
         train_type_lbl = factor(train_type,
                                 labels = c("Complete data", "Model updating")),
         run_iter = paste(run, iter, sep = '/'))


data.merge <- data.merge %>%
  mutate(experiment_lbl = factor(experiment,
                                 labels = c("ACTG", "GBCS", "PBC", "WHAS")),
         model_lbl = factor(model,
                            labels = c("DeepSurv", "BPS Exp", "BPS Wb")),
         run_iter = paste(run, iter, sep = '/'))
```

Show number of runs per dataset and model combination.

```
data %>%
  filter(iter == 0) %>%
  select(model_lbl, model, experiment_lbl, train_type_lbl, cindex) %>%
  group_by(model_lbl, experiment_lbl, train_type_lbl) %>%
  summarise(
    n = n(),
    lbl = first(model),
  )
```

```
# A tibble: 24 x 5
# Groups:   model_lbl, experiment_lbl [12]
   model_lbl experiment_lbl train_type_lbl     n lbl
   <fct>     <fct>          <fct>          <int> <chr>
 1 DeepSurv  ACTG           Complete data     75 deepsurv
 2 DeepSurv  ACTG           Model updating    75 deepsurv
 3 DeepSurv  GBCS           Complete data     75 deepsurv
 4 DeepSurv  GBCS           Model updating    75 deepsurv
 5 DeepSurv  PBC            Complete data     75 deepsurv
 6 DeepSurv  PBC            Model updating    75 deepsurv
 7 DeepSurv  WHAS           Complete data     75 deepsurv
 8 DeepSurv  WHAS           Model updating    75 deepsurv
 9 BPS Exp   ACTG           Complete data     75 pmsurv_exponential
10 BPS Exp   ACTG           Model updating    75 pmsurv_exponential
# ... with 14 more rows
```

Results

Group data for plotting first to enable median and CI 95%.

```
data.grouped <- data %>%
  group_by(experiment_lbl, model_lbl, train_type_lbl, iter) %>%
  summarise(
    n = n(),
    median = median(cindex),
    median_lower = wilcox.test(cindex, conf.int = TRUE, exact = FALSE)$conf.int[1],
    median_upper = wilcox.test(cindex, conf.int = TRUE, exact = FALSE)$conf.int[2]
  )

data.merged.grouped <- data.merge %>%
  group_by(experiment_lbl, model_lbl, iter) %>%
  summarise(
    n = n(),
    median = median(cindex.diff),
    median_lower = wilcox.test(cindex.diff, conf.int = TRUE, exact = FALSE)$conf.int[1],
    median_upper = wilcox.test(cindex.diff, conf.int = TRUE, exact = FALSE)$conf.int[2],
    p = wilcox.test(cindex.diff, exact = FALSE)$p.value
  )
```

Performence over time by experiment and model

```
plt.all <- ggline(data = data.grouped,
                  x = 'iter', y = 'median', color = 'train_type_lbl', group = 'train_type_
             facet.by = c('experiment_lbl', 'model_lbl'),
             add.params = list(color = "train_type_lbl", size = 1, width = 0.25),
             palette = 'lancet', size = 0.25) +
  geom_errorbar(data = data.grouped, aes(ymin = median_lower, ymax = median_upper,
                                         color = train_type_lbl), width = 0.25)


plt.all <- ggpar(plt.all, ylab = 'C-Index', xlab = 'Partition', legend.title = "Training t

plt.all
```

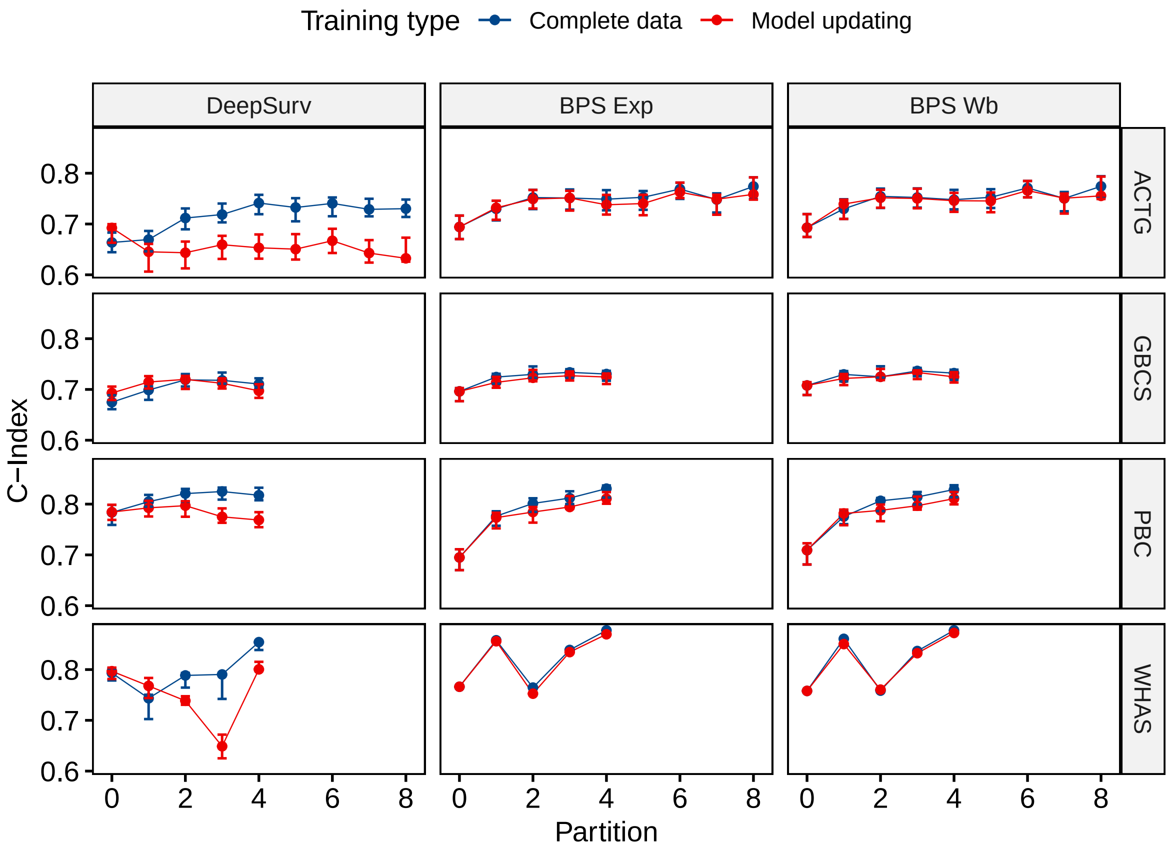

Difference between training types

```
plt.all <- ggline(data = data.merged.grouped,
                  x = 'iter', y = 'median', color='model_lbl',
             facet.by = c('experiment_lbl'),
             add.params = list(size = 1, width = 0.25),
             palette = 'lancet', size = 0.25) +
  geom_errorbar(data = data.merged.grouped,
                aes(color = model_lbl, ymin = median_lower, ymax = median_upper),
                width = 0.25) +
  geom_hline(yintercept = c(-0.01, 0.01), color = 'black', linetype = 'dashed')


plt.all <- ggpar(plt.all, ylab = 'Difference in C-Index', xlab = 'Partition', legend.title

plt.all
```

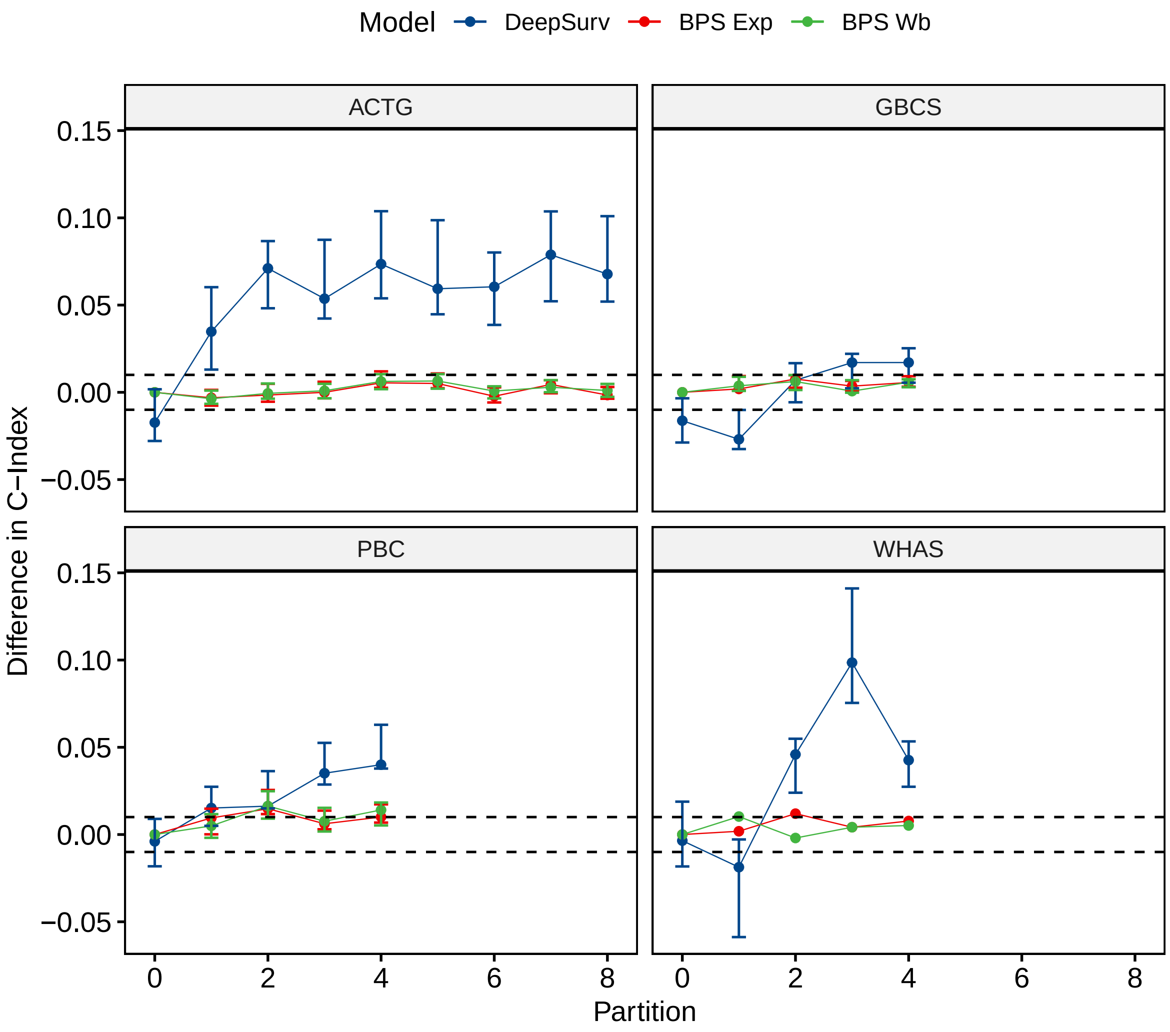

Model
DeepSurv
BPS Exp
BPS Wb
ACTG
GBCS
PBC
WHAS
0.15
0.10
0.05
0.00
−0.05
Difference in C−Index
0
2
4
6
8
Partition

Test for equivalence

```
comparisons <- data.frame('Model'=character(),
                          'Experiment'=character(),
                          'HDI_low'=double(),
                          'HDI_high'=double(),
                          'ROPE_percentage'=double(),
                          'ROPE_equivalence'=character())

for (idx_experiment in levels(data.merge$experiment_lbl)){
  for (idx_model in levels(data.merge$model_lbl)){
    model <- stan_glm(cindex.diff ~ 1 ,
                  data = data.merge %>%
                    filter(experiment_lbl == idx_experiment & model_lbl == idx_model),
                  refresh = 0,
                  algorithm = 'sampling')

    et <- equivalence_test(model,
                 range = c(-0.01, +0.01),
                 ci = 1 - 0.05 / 3
                 )
    comparisons <- rbind(comparisons, data.frame(
      'Model'=idx_model,
      'Experiment'=idx_experiment,
      'HDI_mean'=signif(model$coefficients['(Intercept)'], digits = 2),
      'HDI_low'=signif(et$HDI_low, digits = 2),
      'HDI_high'=signif(et$HDI_high, digits = 2),
      'ROPE_percentage'=round(et$ROPE_Percentage * 100, 1),
      'ROPE_equivalence'=et$ROPE_Equivalence
    ))
  }
}

comparisons %>% as_tibble()
```

```
# A tibble: 12 x 7
   Model    Experiment HDI_mean  HDI_low HDI_high ROPE_percentage ROPE_equival~1
   <chr>    <chr>         <dbl>    <dbl>    <dbl>           <dbl> <chr>
 1 DeepSurv ACTG        0.061    0.052    0.07                0   Rejected
 2 BPS Exp  ACTG        0.0026   0.00072  0.0045            100   Accepted
 3 BPS Wb   ACTG        0.0023   0.00055  0.0039            100   Accepted
```

```
 4 DeepSurv GBCS       -0.00043 -0.0072    0.0062           100   Accepted
 5 BPS Exp  GBCS        0.0046   0.003     0.0063           100   Accepted
 6 BPS Wb   GBCS        0.0042   0.0023    0.006            100   Accepted
 7 DeepSurv PBC         0.026    0.019     0.033              0   Rejected
 8 BPS Exp  PBC         0.0094   0.0063    0.013             68.1 Undecided
 9 BPS Wb   PBC         0.0088   0.0054    0.012             79.6 Undecided
10 DeepSurv WHAS        0.029    0.014     0.043              0   Rejected
11 BPS Exp  WHAS        0.0052   0.0046    0.0057           100   Accepted
12 BPS Wb   WHAS        0.0035   0.003     0.0041           100   Accepted
# ... with abbreviated variable name 1: ROPE_equivalence
```

```r
comparisons$cindex <- sprintf("%0.4f [%0.4f - %0.4f]", comparisons$HDI_mean, comparisons$H

comparisons %>% gt() %>% gtsave(filename = 'out/results_retrain.rtf')
```

```r
ggscatter(data = comparisons, x = 'Model', y = 'HDI_mean',
          facet.by = 'Experiment',
          color = 'Model', palette = 'lancet', ylab = 'Difference in C-Index') +
  geom_hline(yintercept = c(-0.01, 0.01), color = 'black', linetype = 'dashed') +
  geom_errorbar(data = comparisons,
                aes(color = Model, ymin = HDI_low, ymax = HDI_high),
                width = 0.25)
```

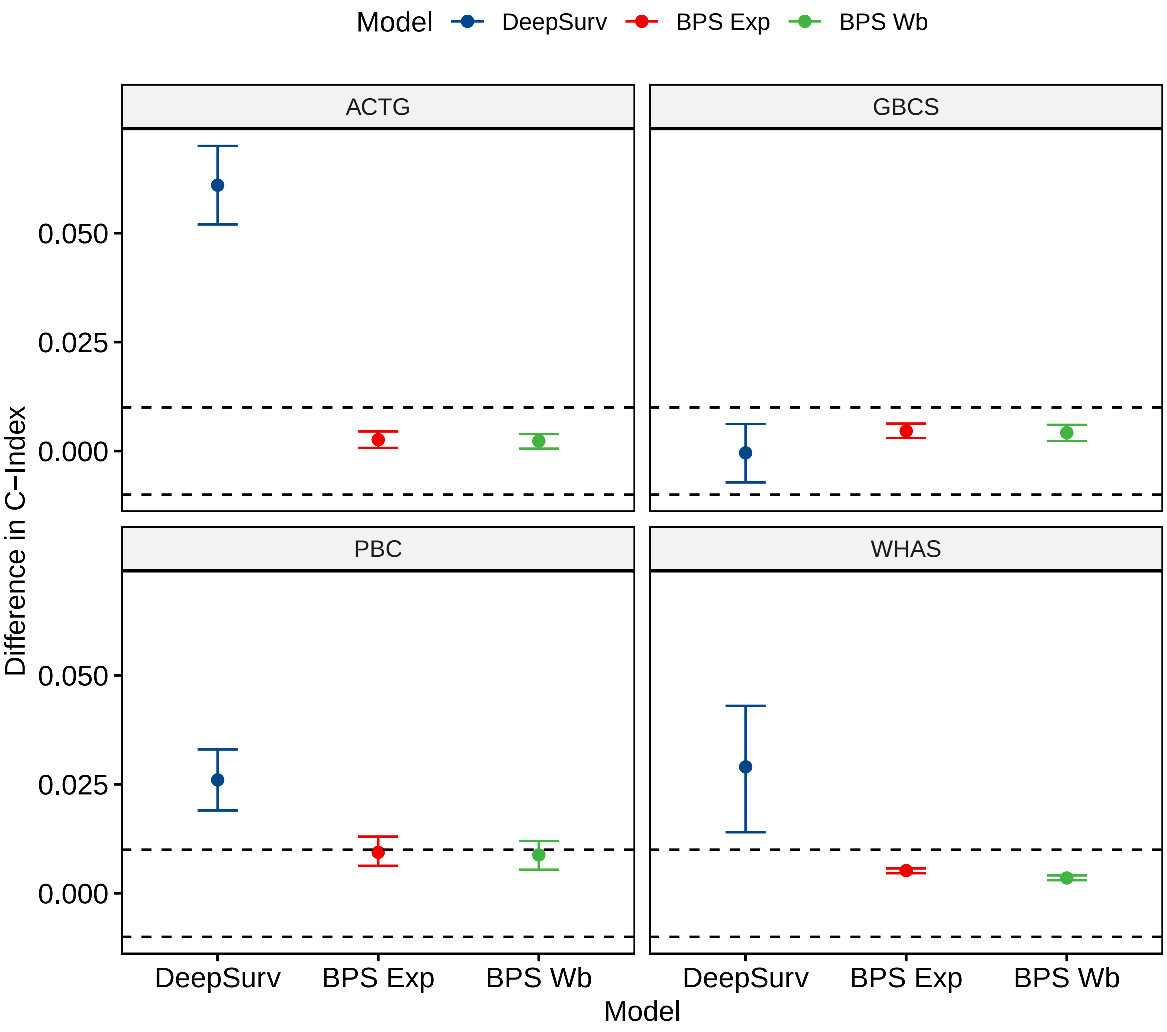

Model
DeepSurv
BPS Exp
BPS Wb
ACTG
GBCS
PBC
WHAS
Difference in C−Index
0.050
0.025
0.000
Model

Performance by model

ACTG

```
plt.actg <- data %>% filter(experiment_lbl == 'ACTG') %>%
  ggline(data = ., x = 'iter', y = 'cindex', color = 'train_type_lbl', group = 'train_type
       add = c("mean_ci"), facet.by = c('experiment_lbl', 'model_lbl'),
       add.params = list(color = "train_type_lbl", size = 1, width = 0.25),
       palette = 'lancet', size = 0.5)

plt.actg <- ggpar(plt.actg, ylab = 'C-Index', xlab = '', legend.title = "Training type")

plt.actg
```

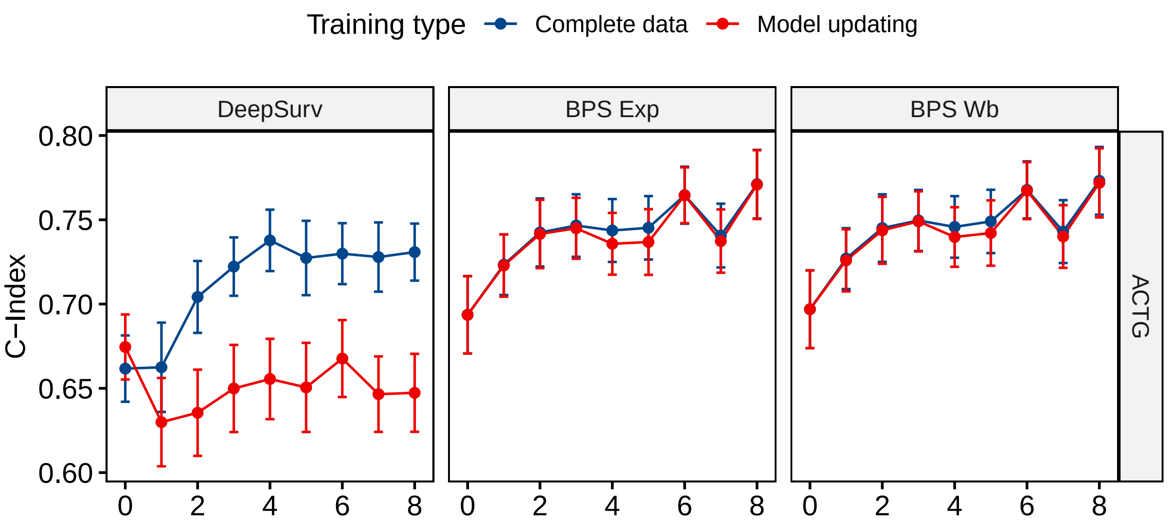

GBCS

```
plt.gbcs <- data %>% filter(experiment_lbl == 'GBCS') %>%
  ggline(data = ., x = 'iter', y = 'cindex', color = 'train_type_lbl', group = 'train_type
       add = c("mean_ci"), facet.by = c('experiment_lbl', 'model_lbl'),
       add.params = list(color = "train_type_lbl", size = 1, width = 0.25),
       palette = 'lancet', size = 0.5)

plt.gbcs <- ggpar(plt.gbcs, ylab = 'C-Index', xlab = '', legend.title = "Training type")

plt.gbcs
```

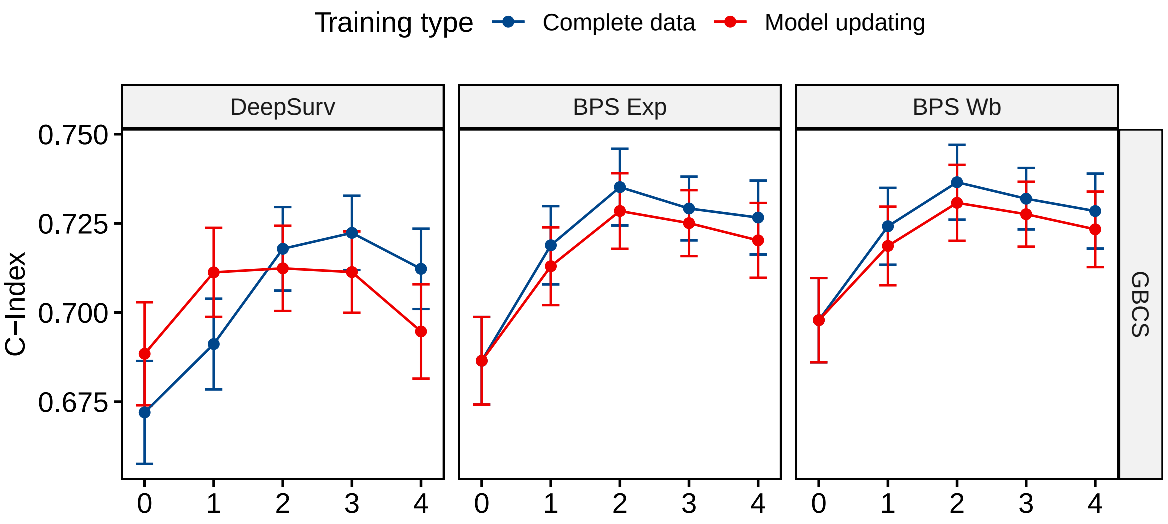

PBC

```
plt.pbc <- data %>% filter(experiment_lbl == 'PBC') %>%
  ggline(data = ., x = 'iter', y = 'cindex', color = 'train_type_lbl', group = 'train_type
       add = c("mean_ci"), facet.by = c('experiment_lbl', 'model_lbl'),
       add.params = list(color = "train_type_lbl", size = 1, width = 0.25),
       palette = 'lancet', size = 0.5)

plt.pbc <- ggpar(plt.pbc, ylab = 'C-Index', xlab = '', legend.title = "Training type")

plt.pbc
```

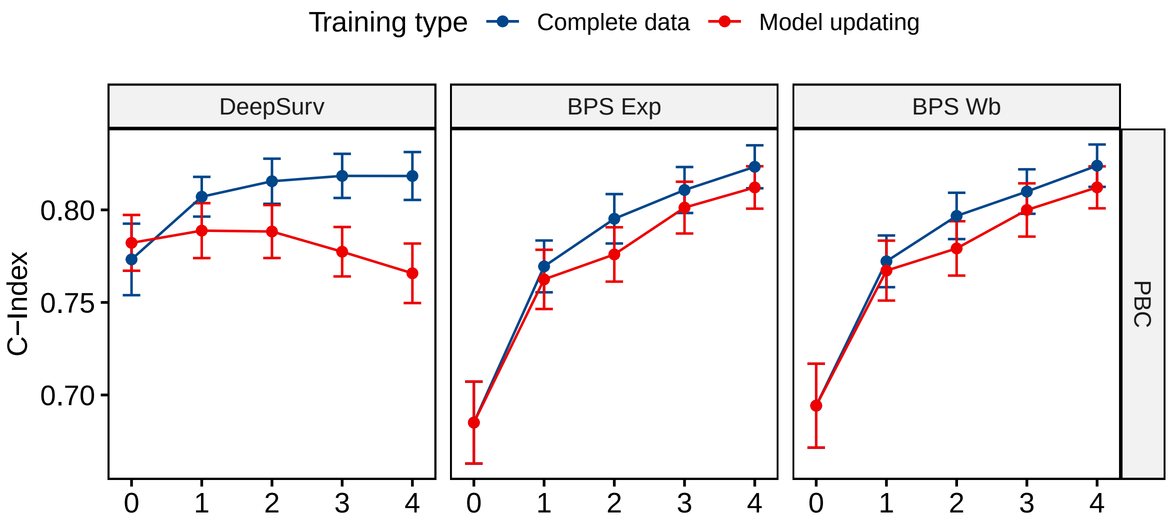

WHAS

```
plt.whas <- data %>% filter(experiment_lbl == 'WHAS') %>%
  ggline(data = ., x = 'iter', y = 'cindex', color = 'train_type_lbl', group = 'train_type
       add = c("mean_ci"), facet.by = c('experiment_lbl', 'model_lbl'),
       add.params = list(color = "train_type_lbl", size = 1, width = 0.25),
       palette = 'lancet', size = 0.5)

plt.whas <- ggpar(plt.whas, ylab = 'C-Index', xlab = '', legend.title = "Training type")

plt.whas
```

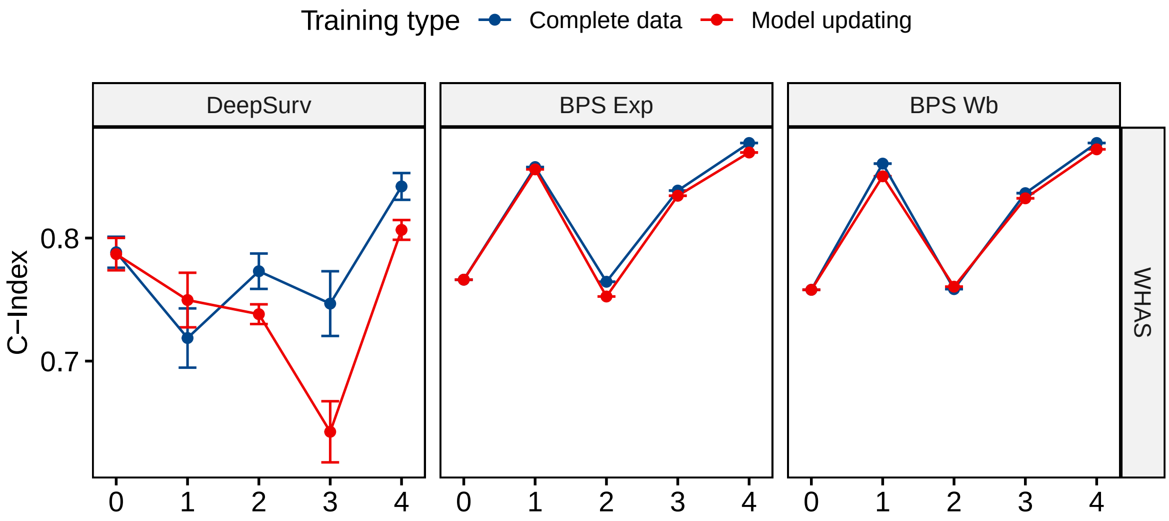

Supplement: Supplementary file 4 — Additional file 4. Retraining. [file 12874_2023_2059_MOESM4_ESM.pdf]
