# Supplementary material for: Bayesian parametric models for survival prediction in medical applications
Source: BMC Med Res Methodol. 2023 Oct 26;23:250. doi: 10.1186/s12874-023-02059-4 (PMC10605790; doi:10.1186/s12874-023-02059-4)
Supplement: Supplementary file 5 — Additional file 5. Sample size calculation. [file 12874_2023_2059_MOESM5_ESM.pdf]

Bayesian parametric models for survival prediction in medical applications

Sample size calculation

Iwan Paolucci, PhD

12-02-2022

Table of contents

Setup 1

Assumptions 1

Sample size for comparative experiments 2

Sample size for model re-training 3

Setup

```
library(pwr)
library(TOSTER)
```

Assumptions

- This study involves multiple testing of certain performance thresholds. Bonferroni correction is used to account for multiple testing.

Sample size for comparative experiments

```r
sample_sd <- 0.025
mu_diff <- 0.02
n_comparisons <- 15

pwr.calc <- pwr.t.test(
  d = mu_diff / sample_sd,
  power = 0.8,
  sig.level = 0.05 / n_comparisons,
  type = 'two.sample',
  alternative = 'two.sided'
)

pwr.calc$n_comparisons <- n_comparisons
pwr.calc$n_adjusted <- pwr.calc$n * 1.15

pwr.calc
```

```
     Two-sample t test power calculation

              n = 46.75793
              d = 0.8
      sig.level = 0.003333333
          power = 0.8
    alternative = two.sided
  n_comparisons = 15
     n_adjusted = 53.77161

NOTE: n is number in *each* group
```

Sample size for model re-training

```
sample_sd <- 0.025
n_comparisons <- 3

pwr.calc <- power_t_TOST(
  n = NULL,
  delta = 0,
  sd = sample_sd,
  low_eqbound = -0.01,
  high_eqbound = 0.01,
  alpha = 0.05 / n_comparisons ,
  power = 0.8,
  type = "two.sided"
)

pwr.calc$n_comparisions <- n_comparisons
pwr.calc$n_adjusted <- pwr.calc$n * 1.15

pwr.calc
```

```
     TOST power calculation 

          power = 0.8
           beta = 0.2
          alpha = 0.01666667
              n = 74.95133
          delta = 0
             sd = 0.025
         bounds = -0.01, 0.01
n_comparisions = 3
     n_adjusted = 86.19403
```
