# Supplementary material for: Bayesian parametric models for survival prediction in medical applications
Source: BMC Med Res Methodol. 2023 Oct 26;23:250. doi: 10.1186/s12874-023-02059-4 (PMC10605790; doi:10.1186/s12874-023-02059-4)
Supplement: Supplementary file 6 — Additional file 6. Overfitting. [file 12874_2023_2059_MOESM6_ESM.pdf]

Bayesian parametric models for survival prediction in medical applications

Assessing overfitting

Iwan Paolucci, PhD

7/14/23

Table of contents

Aim 1

Setup 2

Load data 3

Preprocess data . . . 3

Results 4

Graphical . . . 4

Bayesian . . . 5

Test for equivalence . . . 6

Aim

The aim of this experiment is to compare the amount of overfitting between Bayesian parametric survival models from the pymc-survival package against CoxPH, Random Survival Forest (RSF) and DeepSurv models.

Significant overfitting is defined when all other models are below the 95% CI of the best performing model.

Setup

```r
library(ggplot2)
library(ggpubr)
library(gtsummary)
library(dplyr)
library(gt)
library(rstanarm)
library(parameters)
library(stringr)
```

Load data

```
data.raw <- read.csv('data/results_overfitting.csv')
```

Preprocess data

```
data <- data.raw %>%
  mutate(experiment_name = experiment,
         experiment = str_sub(experiment_name, -3, -1),
         model = str_sub(experiment_name, 0, 3),
         experiment_lbl = factor(experiment,  ordered = FALSE,
                                levels = c("ids", "bcs", 'pbc', 'ran', 'has'),
                                labels = c("ACTG", "GBCS", "PBC", "Veteran", "WHAS")),
         model_lbl = factor(model, ordered = FALSE,
                           levels = c('cox', 'dee', 'exp', 'wb_', 'nnw', 'rsf'),
                           labels = c("CoxPH", 'DeepSurv', "BPS Exp", "BPS Wb", "BPS WbNN
```

```
data %>%
  select(model_lbl, model, experiment_lbl, cindex_diff) %>%
  group_by(model_lbl, experiment_lbl) %>%
  summarise(
    n = n(),
    lbl = first(model),
  ) %>% as.data.frame()
```

```
   model_lbl experiment_lbl  n lbl
1      CoxPH           ACTG 47 cox
2      CoxPH           GBCS 47 cox
3      CoxPH            PBC 47 cox
4      CoxPH        Veteran 47 cox
5      CoxPH           WHAS 47 cox
6   DeepSurv           ACTG 47 dee
7   DeepSurv           GBCS 47 dee
8   DeepSurv            PBC 47 dee
9   DeepSurv        Veteran 47 dee
10  DeepSurv           WHAS 47 dee
11   BPS Exp           ACTG 47 exp
12   BPS Exp           GBCS 47 exp
```

```
13   BPS Exp           PBC 47 exp
14   BPS Exp       Veteran 47 exp
15   BPS Exp          WHAS 47 exp
16    BPS Wb          ACTG 47 wb_
17    BPS Wb          GBCS 47 wb_
18    BPS Wb           PBC 47 wb_
19    BPS Wb       Veteran 47 wb_
20    BPS Wb          WHAS 47 wb_
21  BPS WbNN          ACTG 47 nnw
22  BPS WbNN          GBCS 47 nnw
23  BPS WbNN           PBC 47 nnw
24  BPS WbNN       Veteran 47 nnw
25  BPS WbNN          WHAS 47 nnw
26       RSF          ACTG 47 rsf
27       RSF          GBCS 47 rsf
28       RSF           PBC 47 rsf
29       RSF       Veteran 47 rsf
30       RSF          WHAS 47 rsf
```

Results

Graphical

```
ggboxplot(data = data, x = 'model_lbl', y = 'cindex_diff', fill = 'model_lbl', facet.by =
          ylab = 'C-Index', xlab = 'Model', palette = 'lancet') +
  theme(axis.text.x = element_text(angle = 90, vjust = 0.5))
```

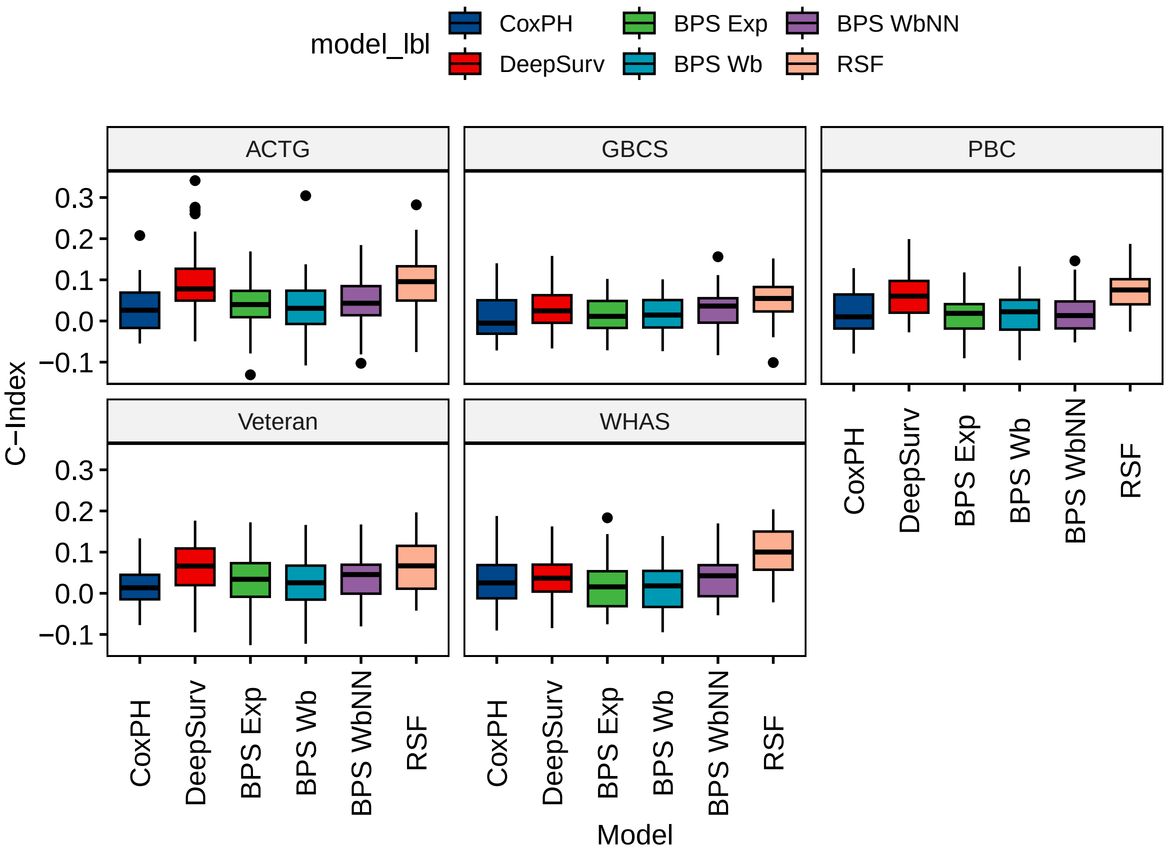

Bayesian

```
custom_mean_ci_bayes <- function(data, variable, ...) {
  model <- stan_glm(cindex_diff ~ 1 ,
                    data = data ,
                    refresh = 0,
                    algorithm = 'sampling')

  model_hdi <- model_parameters(model, ci = 1-0.05/15)

  dplyr::tibble(
    mean = model_hdi$Median[1],
    conf.low = model_hdi$CI_low[1],
    conf.high = model_hdi$CI_high[1]
  )
```

Table 1: **?(caption)**

```
}
```

```
res.tbl <- data %>%
  select(model_lbl, experiment_lbl, cindex_diff) %>%
  tbl_strata(strata = experiment_lbl,  ~.x %>%
    tbl_custom_summary(by = 'model_lbl',
                type = list(
                  cindex_diff ~ 'continuous'),
                label = list(
                  cindex_diff ~ 'C-Index'),
                digits = everything() ~ 3,
                stat_fns = ~ custom_mean_ci_bayes,
                statistic = ~ "{mean} [{conf.low} - {conf.high}]"
                ) ,
    .combine_with = 'tbl_stack'
  ) %>%
  modify_footnote(
    update = all_stat_cols() ~ "Mean [95% CI]"
  )

res.tbl
res.tbl %>% as_gt() %>% gtsave(filename = 'out/results_overfitting_bayes.rtf')
```

Test for equivalence

```
comparisons <- data.frame('Model'=character(),
                          'Experiment'=character(),
                          'HDI_low'=double(),
                          'HDI_high'=double(),
                          'ROPE_percentage'=double(),
                          'ROPE_equivalence'=character())

for (idx_experiment in levels(data$experiment_lbl)){
  for (idx_model in levels(data$model_lbl)){
    if (nrow(data %>% filter(experiment_lbl == idx_experiment & model_lbl == idx_model)) >
      model <- stan_glm(cindex_diff ~ 1 ,
                    data = data %>%
                      filter(experiment_lbl == idx_experiment & model_lbl == idx_model),
                    refresh = 0,
```

Table 2: **?(caption)**

Group	Characteristic	**CoxPH**, N = 47	**DeepSurv**, N = 47	**BPS Exp**, N = 47	**BPS Wb**, N = 47	**BPS WbNN**, N = 47	**RSF**, N = 47
ACTG	C-Index	0.031 [0.005 - 0.056]	0.096 [0.057 - 0.141]	0.038 [0.013 - 0.064]	0.037 [0.007 - 0.065]	0.043 [0.016 - 0.072]	0.090 [0.058 - 0.122]
GBCS	C-Index	0.007 [-0.014 - 0.029]	0.032 [0.010 - 0.056]	0.014 [-0.006 - 0.034]	0.016 [-0.005 - 0.038]	0.030 [0.008 - 0.052]	0.053 [0.034 - 0.074]
PBC	C-Index	0.018 [-0.004 - 0.040]	0.064 [0.042 - 0.089]	0.015 [-0.006 - 0.041]	0.018 [-0.008 - 0.041]	0.017 [-0.005 - 0.038]	0.074 [0.053 - 0.095]
Veteran	C-Index	0.022 [0.002 - 0.044]	0.063 [0.032 - 0.094]	0.029 [-0.004 - 0.060]	0.027 [-0.005 - 0.056]	0.037 [0.014 - 0.061]	0.066 [0.034 - 0.097]
WHAS	C-Index	0.028 [0.004 - 0.051]	0.039 [0.012 - 0.064]	0.020 [-0.007 - 0.046]	0.016 [-0.009 - 0.040]	0.034 [0.012 - 0.058]	0.098 [0.071 - 0.125]

```
                    algorithm = 'sampling')

        et <- equivalence_test(model,
                     range = c(-0.05, +0.05),
                     ci = 1 - 0.05 / 15
                     )
        comparisons <- rbind(comparisons, data.frame(
          'Model'=idx_model,
          'Experiment'=idx_experiment,
          'HDI_mean'=signif(model$coefficients['(Intercept)'], digits = 2),
          'HDI_low'=signif(et$HDI_low, digits = 2),
          'HDI_high'=signif(et$HDI_high, digits = 2),
          'ROPE_percentage'=round(et$ROPE_Percentage * 100, 1),
          'ROPE_equivalence'=et$ROPE_Equivalence
        ))
      }
    }
  }
```

```r
comparisons %>% as_tibble()
```

```
# A tibble: 30 x 7
   Model    Experiment HDI_mean HDI_low HDI_high ROPE_percentage ROPE_equivale~1
   <chr>    <chr>         <dbl>   <dbl>    <dbl>           <dbl> <chr>
 1 CoxPH    ACTG         0.031   0.0068    0.055            99.5 Undecided
 2 DeepSurv ACTG         0.096   0.057     0.13              0   Rejected
 3 BPS Exp  ACTG         0.038   0.011     0.063            92.8 Undecided
 4 BPS Wb   ACTG         0.037   0.0048    0.066            91.2 Undecided
 5 BPS WbNN ACTG         0.043   0.017     0.069            78.7 Undecided
 6 RSF      ACTG         0.09    0.058     0.12              0   Rejected
 7 CoxPH    GBCS         0.0074 -0.016     0.029           100   Accepted
 8 DeepSurv GBCS         0.032   0.011     0.055            99.1 Undecided
 9 BPS Exp  GBCS         0.014  -0.0053    0.034           100   Accepted
10 BPS Wb   GBCS         0.016  -0.0031    0.038           100   Accepted
# ... with 20 more rows, and abbreviated variable name 1: ROPE_equivalence
```

```r
comparisons$cindex <- sprintf("%0.4f [%0.4f - %0.4f]", comparisons$HDI_mean, comparisons$H

comparisons %>% gt() %>% gtsave(filename = 'out/results_overfit_compare.rtf')
```

```r
ggscatter(data = comparisons, x = 'Model', y = 'HDI_mean',
          facet.by = 'Experiment',
          color = 'Model', palette = 'lancet', ylab = 'Decrease in C-Index') +
  geom_hline(yintercept = c(0.01), color = 'black', linetype = 'dashed') +
  geom_errorbar(data = comparisons,
                aes(color = Model, ymin = HDI_low, ymax = HDI_high),
                width = 0.25) +
  ylim(-0.05, 0.15) +
  theme(axis.text.x = element_text(angle = 90, vjust = 0.5))
```

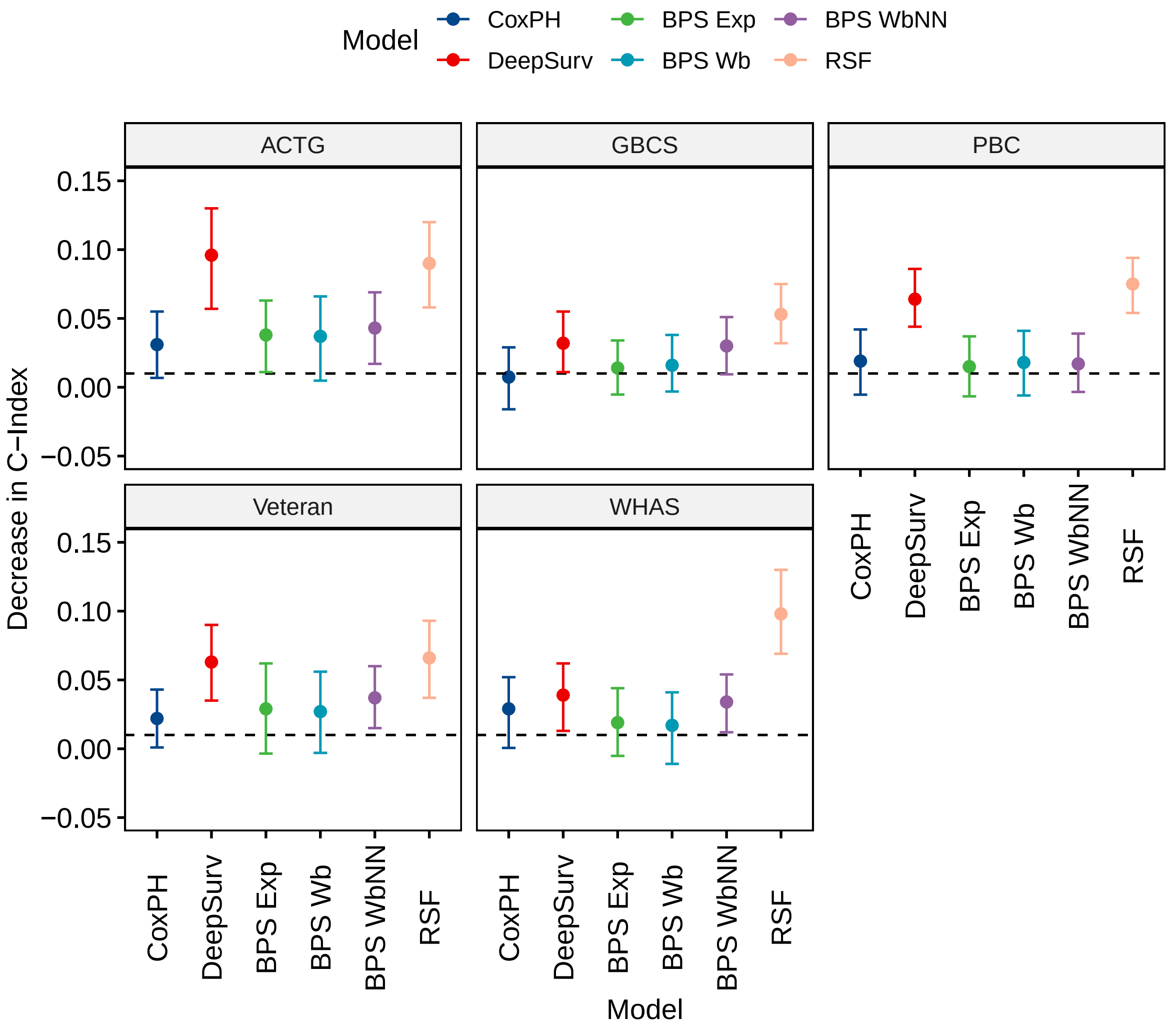
Model
CoxPH
DeepSurv
BPS Exp
BPS Wb
BPS WbNN
RSF
ACTG
GBCS
PBC
Veteran
WHAS
Decrease in C−Index
0.15
0.10
0.05
0.00
−0.05
Model
